# Non-decarboxylative C–H Fluorination of Carboxylic Acids by Iridium Photocatalysis

**DOI:** 10.1021/acs.orglett.6c01991

**Published:** 2026-06-10

**Authors:** Ji Won Lee, Thorsten Bach, Biki Ghosh

**Affiliations:** Department Chemie and Catalysis Research Center (CRC), TUM School of Natural Sciences, Technische Universität München, 85747 Garching, Germany

## Abstract

We have developed a photoredox-initiated non-decarboxylative C–H fluorination of carboxylic acids. Using Selectfluor as the fluorinating agent, a range of primary and secondary carboxylic acids were site-selectively fluorinated at their β- to ϵ-positions. Mechanistic studies suggest that the reaction proceeds by photocatalytic reduction of Selectfluor, generating an *N*-centered radical that mediates intermolecular hydrogen atom abstraction and propagates a radical chain fluorination process.

## Graphical abstract

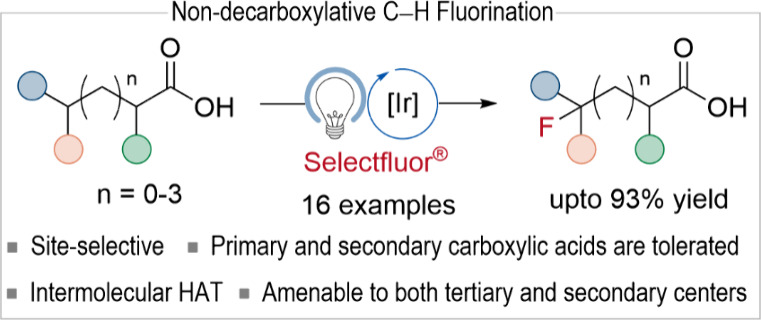

Owing to its small size and high electronegativity, fluorine exerts a profound influence on the chemical, physical, and biological properties of organic molecules, including solubility, lipophilicity, conformational flexibility, and metabolic stability.^1^ As a result, the use of partially fluorinated molecules as active pharmaceutical ingredients has markedly increased over the past decades. In addition, organofluorines have been widely utilized in synthetic chemistry as building blocks, reagents, and solvents.^2^ Consequently, the development of efficient, site-selective methods for C–F bond construction has garnered immense interest in the realm of organic synthesis.^3^

Carboxylic acids, being among the most abundant, inexpensive, and versatile feedstocks in organic synthesis,^4^ represent attractive radical precursors. They have therefore been extensively exploited in decarboxylative functionalization strategies,^5^ and decarboxylative fluorination has emerged as a convenient technique for the synthesis of organofluorines (Scheme 1A).^6,7^ Generally, the carboxylic acid is converted to a carbon-centered radical upon oxidation and decarboxylation, either by reacting with a metal^8^ or*via* photoredox catalysis.^9^ Subsequent interception of the generated radical by an electrophilic N–F reagent, such as Selectfluor or *N*-fluorobenzenesulfonimide (NFSI), results in the formation of C–F bonds. In recent years, several site-selective decarboxylative radical fluorination reactions of aliphatic carboxylic acids have been developed. Notably, the Li group^8a^ described the first decarboxylative fluorination of aliphatic carboxylic acid catalyzed by silver. Subsequently, the Sammis/Paquin,^9a,9^ MacMillan (Scheme 1B),^9d^ and Ye^9e^ groups successively reported decarboxylative fluorination strategies under photoredox catalysis. Although these methods^8–10^ are synthetically useful, the loss of a carboxyl functionality restricts their application when the retention of the carboxyl group is mandatory for further transformations.

**Scheme 1:**
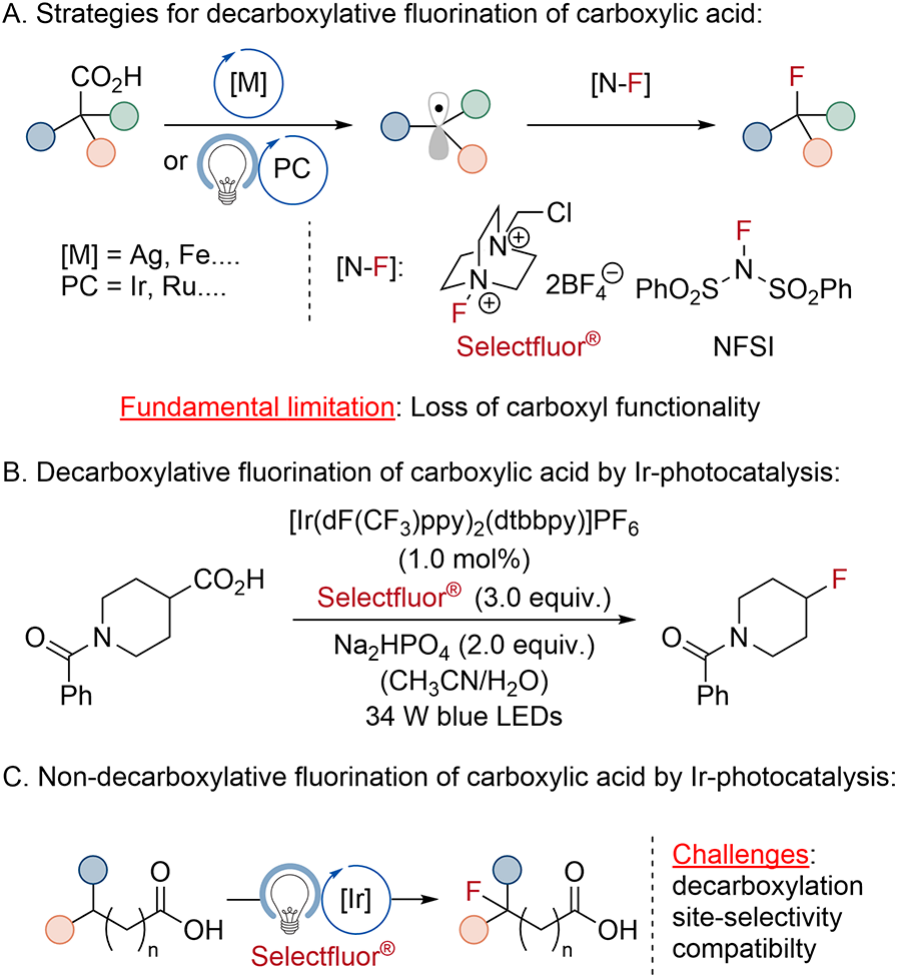
Background and This Work

In contrast, non-decarboxylative^11^ fluorination^12^ strategies that preserve the acid functionality have recently begun to emerge, including transition-metal-catalyzed directed C­(sp^3^)–H fluorination of carboxylic acids.^12b^ However, methods that enable predictable, remote C–H fluorination of unactivated carboxylic acids without the need for directing groups remain underdeveloped. Achieving site-selective remote C–H fluorination under such conditions presents significant challenges, including the controlled generation of reactive intermediates, suppression of competing decarboxylation pathways, and predictable site selectivity along aliphatic chains.

As part of our ongoing research program on developing a C–H functionalization reaction via hydrogen atom transfer (HAT),^13^ we became interested in developing a remote C–H fluorination of aliphatic carboxylic acids under photoredox catalysis. Although HAT-based fluorination^14^ methods using an electrophilic N–F reagent, such as Selectfluor, have been reported,^15^ they typically operate on simple hydrocarbons or require specific directing groups, and their application to unactivated carboxylic acids with predictable site-selectivity remains limited. Therefore, the development of a general strategy that enables remote, site-selective C–H fluorination while preserving the carboxylic acid functionality would provide a complementary approach to existing methods and expand the synthetic utility of abundant carboxylic acid feedstocks. Herein, we have developed a non-decarboxylative C–H fluorination of carboxylic acids under iridium photocatalysis using Selectfluor as a fluorinating agent (Scheme 1C).

The optimization studies commenced with the fluorination of commercially available 4-methylpentanoic acid **1a** using Selectfluor as an electrophilic fluorinating agent. The choice of [Ir­(dF­(CF_3_)­ppy)_2_(dtbbpy)]­PF_6_ (**I**) as the photocatalyst was inspired by prior work of the MacMillan group^9d^ and others^9^ in photofluorination reactions. Under irradiation in 1:1 CH_3_CN/H_2_O at 30 °C, using 2 mol % **I**, 5 equiv. of Selectfluor, and Na_2_HPO_4_ as the base, the desired γ-fluorinated acid **2a** was obtained in 53% yield (Table 1, entry 1). Along with the desired product, the corresponding lactone **3** was also detected in the crude reaction mixture by ^1^H NMR as a byproduct. Lower loading of either Selectfluor or catalyst led to lower yields (see the Supporting Information for details). Under identical conditions, organic photocatalyst 9-mesityl-10-methylacridinium tetrafluoroborate (**II**)^9e^ also afforded the desired product, albeit in a slightly lower yield of 48% (Table 1, entry 2). In the absence of photocatalyst, light, and Selectfluor, the starting material remained unreacted (Table 1, entries 3–5).

**Table 1:**
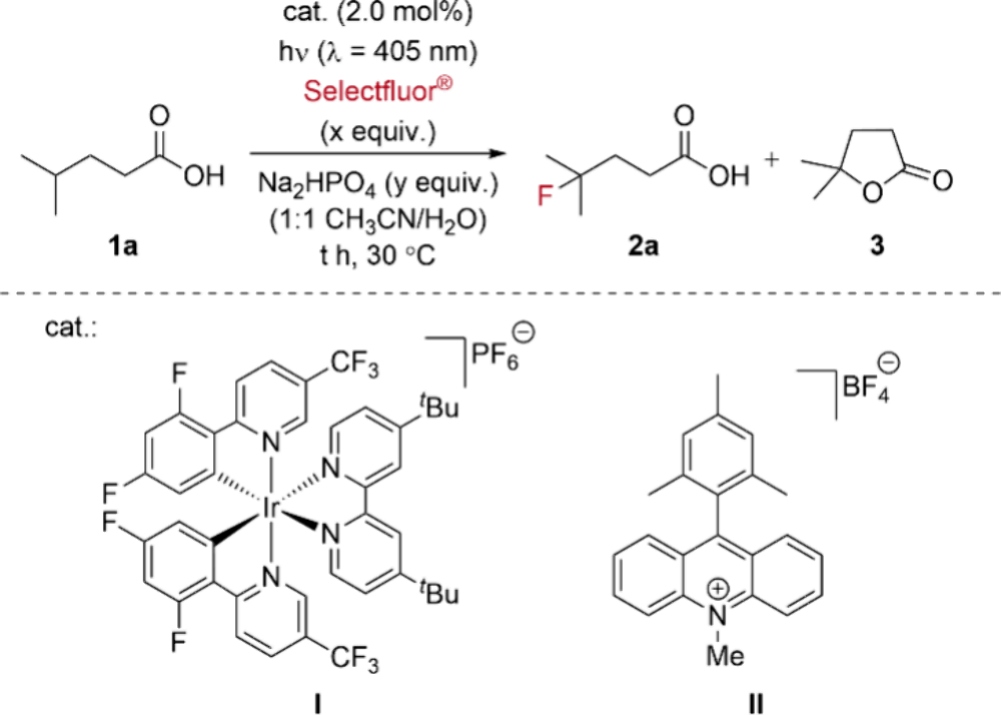
Optimization of Reaction Parameters for the Fluorination of 4-Methylpentanoic Acid **1a** ^a^

entry^a^	catalyst	*x*	*y*	*t* (h)	**2a**/**3**/**1a**	yield (%)
1	**I**	5.0	1.1	12	8.7:0.5:1	53
2	**II**	5.0	1.1	12	9:1:0.1	48
3		5.0	1.1	12	0:0:1	
4^b^	**I** or **II**	5.0	1.1	12	0:0:1	-
5	**I** or **II**	-	1.1	12	0:0:1	-
6	**I**	5.0	1.1	18	1:99:0	<2
7	**I**	5.0	-	12	50:6:1	77
8	**II**	5.0	-	12	14.5:3.9:0.1	73
9	**I**	5.0	-	6	43:2.2:1	93

a The reactions were performed on a 0.40 mmol scale using 4 mL of a 1:1 mixture of CH_3_CN/H_2_O. For catalyst **I**, 10 W 405 nm LEDs were used, and for catalyst **II**, 10 W 437 nm LEDs were used. The yield of **2a** corresponds to the ^1^H NMR yield using PhCF_3_ as an external standard.

b The reaction was carried out without light.

Interestingly, extending the irradiation time to 18 h resulted in a complete successive reaction of product **2a** (Table 1, entry 6), and corresponding lactone **3** was isolated exclusively. This observation suggested that fluorinated product **2a** is unstable under the reaction conditions. Subsequent NMR studies using both crude reaction mixtures and isolated **2a** confirmed that γ-fluorinated acid **2a** readily cyclizes to the corresponding lactone **3** under both reaction and ambient conditions. Nevertheless, the formation of lactone **3** within the catalytic cycle could not be ruled out. Strikingly, performing the reaction without base substantially improved the yields, delivering **2a** in 77 and 73% yields with catalysts **I** and **II**, respectively (Table 1, entries 7 and 8). To suppress the conversion of the product to corresponding lactone, we reduced the irradiation time. Optimization studies revealed that 6 h of irradiation provided the optimal balance, affording the desired product in 93% yield (Table 1, entry 9).

The pronounced base effect observed during optimization studies raised the question of whether the operative mechanism differs in the presence and absence of base. In the presence of base, we initially hypothesized, following the precedent of the MacMillan group,^9d^ that carboxylate of **1a** undergoes single-electron transfer (SET) to the photoexcited iridium catalyst to generate a carboxyl radical. If the propensity to undergo decarboxylation is relatively low, the carboxyl radical may undergo a [1,5]-HAT,^16^ leading to the formation of a carbon-centered radical at the γ-position.^17^ Trapping of this radical by Selectfluor would then furnish γ-fluorinated acid **2a**. While the proposed [1,5]-HAT from the carboxyl radical does constitute a theoretical possibility, we were cognizant of the potential competing pathways. In particular, the rapid decarboxylation of the carboxyl radical could outcompete the desired HAT process. On the other hand, in the absence of the base, intermolecular HAT mediated by the reduced form of Selectfluor can initiate a chain reaction^14i^ without the need of a carboxyl group.^14,15^

To distinguish between these pathways, a series of control experiments were conducted (Scheme 2). A competition experiment using a 1:1 mixture of acid **1a** and its corresponding methyl ester **4** afforded a 5:1 ratio of fluorinated acid **2a** over ester **5** (Scheme 2a), superficially suggesting preferential reactivity of the free acid. However, when ester **4** was employed as the only substrate, fluorinated acid **2a** was formed exclusively, indicating that the ester undergoes hydrolysis under the reaction conditions either before or after the reaction (Scheme 2b). The acid/ester selectivity observed in the competition experiment, therefore, likely reflects this hydrolysis rather than an intrinsic directing effect of the carboxyl group. Crucially, corresponding amide **6** also underwent smooth γ-fluorination, demonstrating that the carboxylic acid functionality is not required for the HAT event and ruling out the [1,5]-HAT pathway from the carboxyl radical (Scheme 2c). However, the irradiation of substrate **8**, lacking the γ-C–H bond, primarily led to substrate consumption, with traces of β-fluorinated acid **9** (Scheme 2d). This experiment is consistent with the unproductive decarboxylation pathways of carboxylates,^9d,18^ although no direct evidence for the decarboxylation was obtained. Stern–Volmer quenching experiments confirmed that the sodium carboxylate salt of **1a** could be oxidized by photocatalyst **II** (see the Supporting Information for details). Taken together, these control experiments indicate that the SET oxidation of carboxylates triggers rapid decarboxylation rather than productive HAT and that the carboxylic acid functionality serves no essential role in the HAT step.

**Scheme 2:**
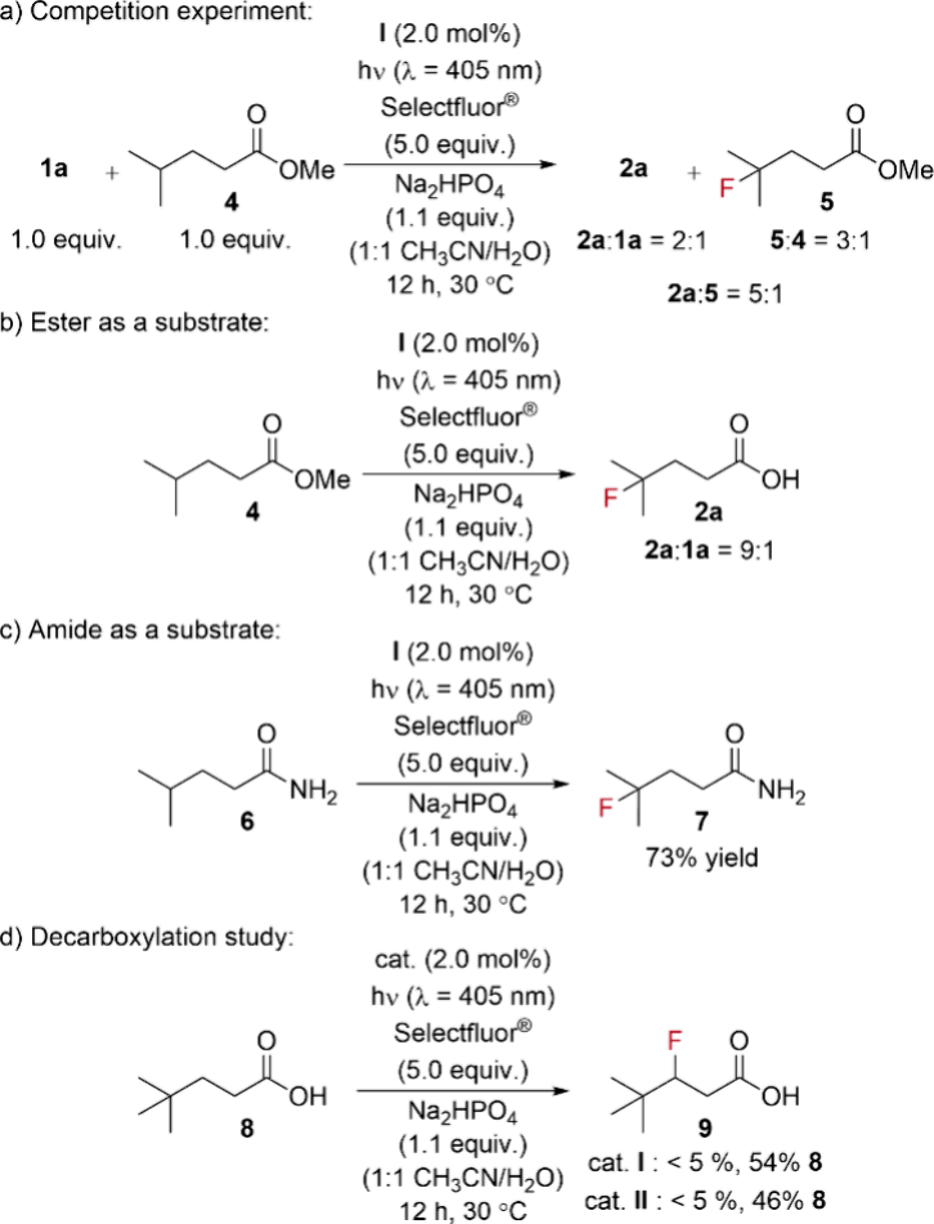
Control Experiments

Based on these experiments and relevant literature precedent,^14,15,19^ a plausible catalytic cycle involving a radical chain mechanism is proposed (Scheme 3). Under the optimized, base-free conditions, visible light irradiation generates photoexcited state PC* of the photocatalyst (**PC**), which reduces Selectfluor by an SET process to produce the *N*-centered radical dication and an equimolar amount of F^–^. The *N*-centered radical dication, known to be a potent hydrogen atom abstractor and radical chain carrier,^14,15^ engages in intermolecular HAT with substrate **1a**, preferentially at the activated and more hydridic C–H positions, thereby generating a carbon-centered radical (**10**). Subsequent interception of **10** by Selectfluor directly affords product **2a** and regenerates the *N*-centered radical dication, enabling propagation of a radical chain process. There might be an alternative oxidative termination pathway, in which **PC** ^•^ ^+^ could oxidize **10** to generate carbocation **11**, closing the catalytic cycle. The carbocation could be trapped by fluoride to produce **3**. While the formation of lactone **3** as the minor side product suggests the formation of a carbocation, it does not constitute direct evidence for the involvement of carbocation intermediates in the catalytic cycle due to the found instability of the product.

**Scheme 3:**
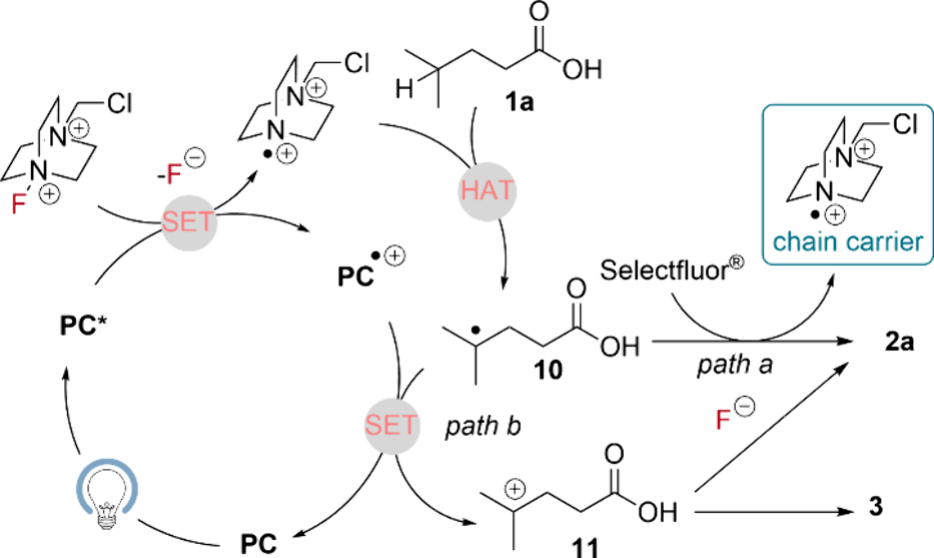
Proposed Catalytic Cycle

With the optimized conditions (Table 1, entry 9) for the photofluorination in hand, the scope of various carboxylic acids was explored (Scheme 4). To mitigate the product instability and volatility, along with the ease of isolation for reliable yields, the fluorinated acids were isolated as corresponding benzyl or methyl ester. Initially, we focused on moving the fluorination site along the chain for the primary carboxylic acids. The reactions of carboxylic acids **1b**–**1d** containing an alkyl side chain with varying lengths of *n* = 0–3 were found to be well-tolerated, giving β-, δ-, and ε-fluorinated products **12b**–**12d** in 46–71% yields with good site-selectivity. The effect of introducing a methyl group at the α-position of **1a** was studied next. We were pleased to see the exclusive incorporation of fluorine at the γ-position, and product **12e** was isolated as benzyl ester in 63% yield. Similarly, site-selective fluorination of secondary aliphatic carboxylic acids **1f** and **1g** was achieved with 70 and 42% yields, respectively. 2-Chloro-4-methylpentanoic acid **1h** was also a competent substrate. Pleasingly, this strategy could be extended to the late-stage fluorination of an amino acid. *N*-Protected amino acid **1i** afforded the desired fluorinated compound, albeit in modest yields, attributed to incomplete conversion (25%). An attempt to improve the conversion by prolonged irradiation was met with failure. When pentanoic acid **1j** was subjected to standard conditions, an inseparable mixture of γ- and β-fluorinated benzyl ester **12j** was obtained with a ratio of 6.5:1 in favor of γ. In addition, several benzoic acids bearing ethyl or isopropyl substituents at *ortho*, *meta*, and *para* positions (**1k**–**1p**) were successfully fluorinated in moderate to low yields. In these cases, fluorination occurs selectively at the benzylic C–H position, demonstrating that the method is not restricted to aliphatic substrates but extends to activated benzylic sites remote from the carboxylic acid functionality. The lower yields are related to competing side reactions and incomplete conversion under standard reaction conditions. Substrates with the possibility of generating a benzylic radical at the α-position, e.g., ibuprofen and fluorene-9-carboxylic acid, led to the formation of complex mixtures. Neither the desired γ-fluorinated product nor the decarboxylative fluorination product was detected by ^1^H or ^19^FNMR analysis of the crude mixture. Finally, 4-phenylbutanoic acid (**1q**) exclusively gave corresponding lactone **15**.

**Scheme 4:**
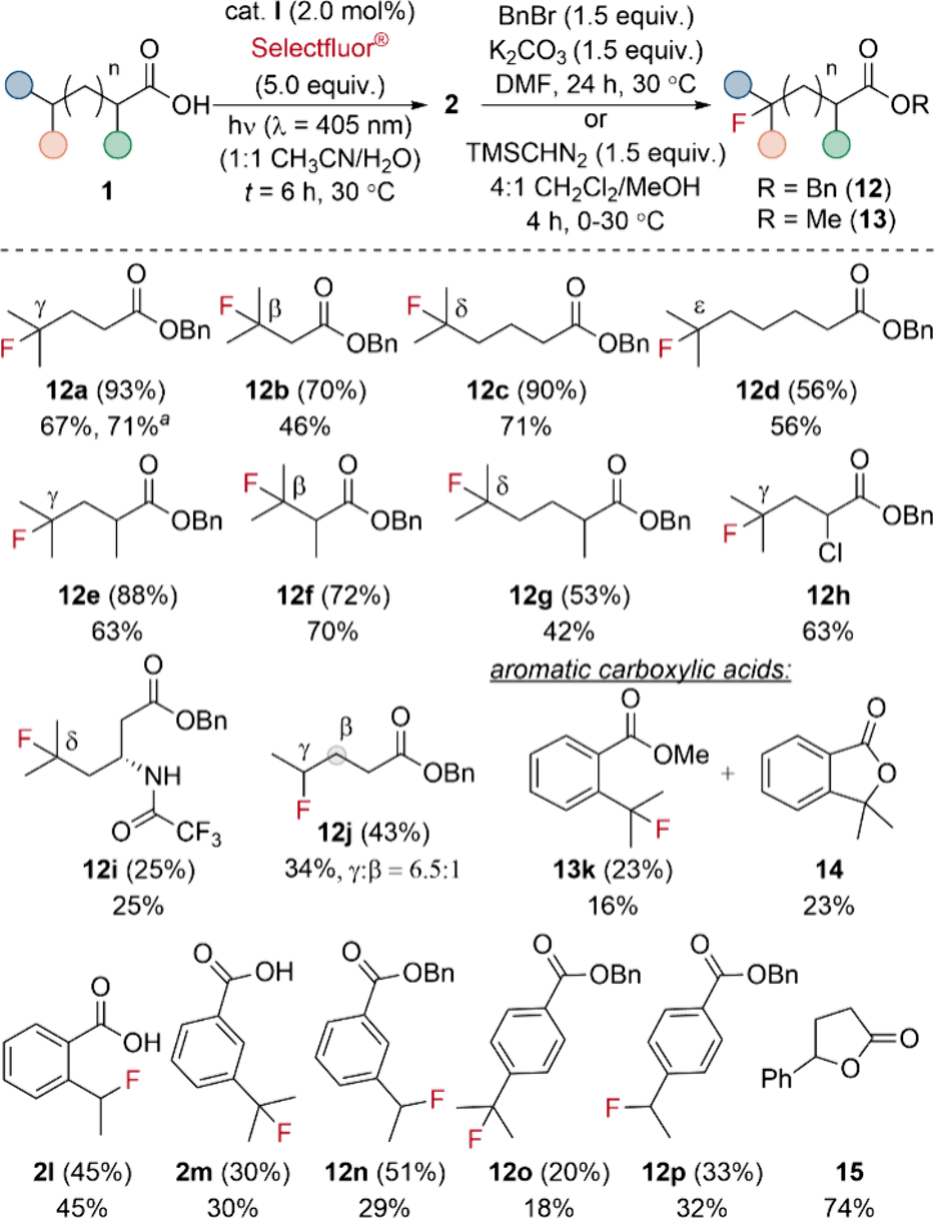
Scope of C–H Fluorination of Carboxylic Acids **1** ^a^

* The reactions were performed on a 0.40 mmol scale using 4 mL of a 1:1 mixture of CH_3_CN/H_2_O as the solvent. Yields correspond to isolated products over two steps. Yields in parentheses are ^1^H NMR yields of **2** determined using PhCF_3_ as an external standard after the photofluorination step.
a The reaction was performed on a 1.00 mmol scale (*t* = 12 h) with 6 mL of a 1:1 mixture of CH_3_CN/H_2_O as the solvent.

In summary, we have developed a non-decarboxylative C–H fluorination of carboxylic acid under photoredox catalysis. Under the established conditions, both iridium photocatalyst **I** and organic acridinium photocatalyst **II** can generate the *N*-centered radical dication from Selectfluor via SET, initiating an intermolecular HAT-based radical chain process. This protocol delivers a range of remote fluorinated primary and secondary carboxylic acids with good site selectivity, targeting both secondary and tertiary C–H bonds as fluorination sites.

## Supplementary Material



## Data Availability

The data underlying this study are available in the published article and its Supporting Information. Primary research data are openly available in the RADAR4Chem repository at 10.22000/ta5brgz3jjhquagw.

## Notes

Materials and methods, synthetic procedures and full characterization for all products (**2a**, **3**, **7**, **12a**–**12p**, **13k**, **14**, and **15**) and products of the synthesis route, spectroscopic data, and NMR spectra (PDF)
