# Supplementary material for: Non-decarboxylative C–H Fluorination of Carboxylic Acids by Iridium Photocatalysis
Source: Org Lett. 2026 Jun 10;28(24):7772–7. doi: 10.1021/acs.orglett.6c01991 (PMC13288877; doi:10.1021/acs.orglett.6c01991)
Supplement: Supplementary file 1 [file ol6c01991_si_001.pdf]

Supporting Information

Non-decarboxylative C–H Fluorination of Carboxylic Acids by Iridium Photocatalysis

Ji Won Lee,[a] Thorsten Bach,*[a] and Biki Ghosh*[a]

[a]Technische Universität München, TUM School of Natural Sciences, Department Chemie and Catalysis Research Center (CRC), 85747 Garching, Germany

*Corresponding author: Email: thorsten.bach@ch.tum.de and biki.ghosh@tum.de

Table of Contents

S1. General Information 3
S2. Analytical Methods 7
S3. General Synthetic Procedures...9
S4. Optimization of the Reaction Conditions 12
S5. Control Experiments...14
S6. Synthesis and Characterization of Carboxylic acids **1**...29
S7. Photofluorination Reactions of Carboxylic Acids **1** 31
S8. NMR Spectra 51
S9. List of References...99

S1. General Information

All reactions sensitive to air or moisture were carried out in flame-dried glassware under positive pressure of argon using standard Schlenk techniques.

Commercially available chemicals were used without further purification, if not further mentioned.

For moisture sensitive reactions, dichloromethane (CH_2Cl_2), diethyl ether (Et_2O) and tetrahydrofuran (THF) were purified using a MBSPS 800 *MBraun* solvent purification system. The following columns were used:

CH_2Cl_2: 2 × MB-KOL-A type (aluminum oxide)

Et_2O: 1 × MB-KOL-A type 2 (aluminum oxide), 1 × MB-KOL-M type 2 (3 Å molecular sieve)

THF: 2 × MB-KOL-M type 2 (3 Å molecular sieve)

Technical solvents for column chromatography [dichloromethane (CH_2Cl_2), ethyl acetate (EtOAc), methanol (MeOH), hexanes, *n*-pentane (pentane)] were used after simple distillation.

Normal-phase flash column chromatography (FCC) was performed on silica 60 (*Merck*, 230-400 mesh) with the indicated eluent mixture.

Commercially available starting materials were purchased either from *Sigma Aldrich (Merck)*, *TCI Chemicals*, *ABCR* or *BLDpharm*.

^{n}Butyl lithium (^{n}BuLi) is an extremely reactive, corrosive, and pyrophoric organometallic reagent. It ignites spontaneously upon contact with air and reacts violently (and exothermically) with water. Strict handling protocols were used to prevent devastating fires and severe chemical burns.

Unless water was used, round bottom flasks, vials and phototubes were dried with a heat gun at approximately 600 °C under vacuum.

Prior to the start of a photoreaction, each reaction mixture was degassed by being sparged with argon under ultrasonication for 10 min.

Datasheet LED074 405 nm / 10 W

Basic Information

Type	High-Power-LED
Description	Single Colour Ultraviolet 10 Watt 400nm - 405nm
Manufacturer / Supplier	ams OSRAM / Mouser
Order number / Date of purch.	LZ4-40UA00-00U7 / 03/2016
Internal lot / serial number	2016-03 / LED074

Specification Manufacturer

Type / size	single emitter / ca. 7 x 7 mm
Mechanical specification	
Electrical specification	700 mA, UF 13.76 - 18.56 V
Wavelength (range, typ.)	400 - 405 nm
Spectral width (FWHM)	n/a
Datasheet	n/a

Characterization

Description of measurement	Measured with Ocean-optics USB4000 spectrometer using a calibrated setup (cosine corrector/fibre). The distance between the emitting surface and the surface of the cosine corrector was 20 mm. The LED was operated at 700 mA on an active heat sink at approx. 20 °C	
Measured dominant wavelength / Int.	405 nm	21326 µW/mm²nm
Measured spectral width (FWHM)	17 nm	
Integral Reference intensity / range	378857 µW/cm²	350-500 nm

Spectrum

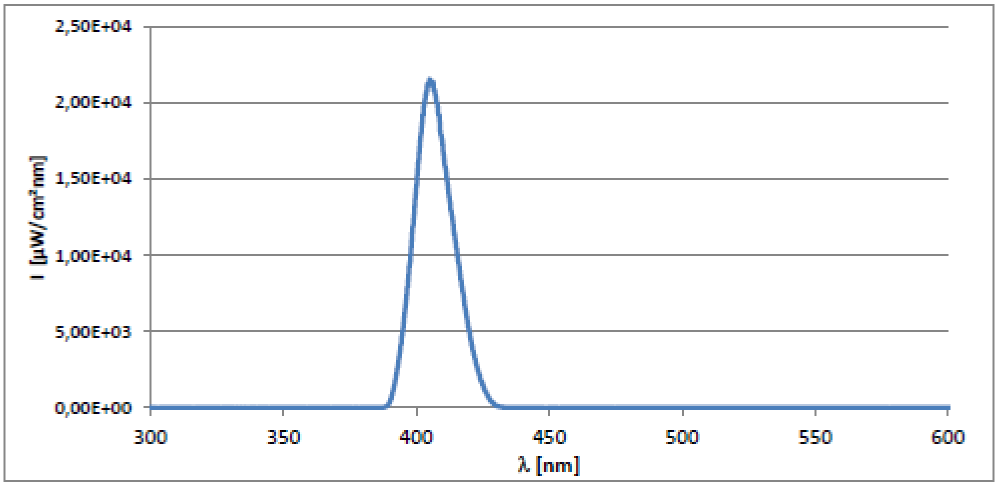

Lehrstuhl OC 1 - TUM

200 nm | 250 nm | 300 nm | 350 nm | 400 nm | 450 nm | 500 nm | 550 nm | 600 nm | 650 nm

Datasheet LED036 Av-440-10W

Basic Information

Type	High-Power-LED
Description	Avonec 440-450 nm / 10 W
Manufacturer / Supplier	n/a / Avonec
Order number / Date of purch.	n/a / 01/2016
Internal lot / serial number	2016-01 / LED036

Specification Manufacturer

Type / size	10 emitter / ca. 1 x 1 mm
Mechanical specification	module, dye-area ca. 7.5 x 4 mm
Electrical specification	700 mA, UF 16 V
Wavelength (range, typ.)	440-450 nm, typ. n/a
Spectral width (FWHM)	n/a
Datasheet	n/a

Characterization

Description of measurement	Measured with Ocean-optics USB4000 spectrometer using a calibrated setup (cosine corrector/fibre). The distance between the emitting surface and the surface of the cosine corrector was 20 mm. The LED was operated at 700 mA on a passive heat-sink at approx. 20 °C
Measured wavelength	437 nm
Measured spectral width	17 nm
Integral Reference intensity	220760 µW/cm² (380-530 nm @ 20 mm distance, 4 mm cosine corr.)

Spectrum

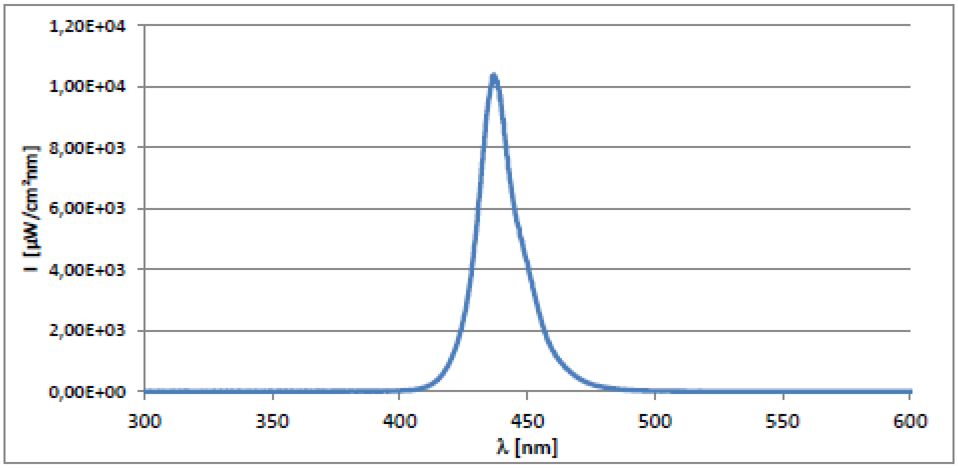

Fig. S1: Emission spectrum of the LEDs.

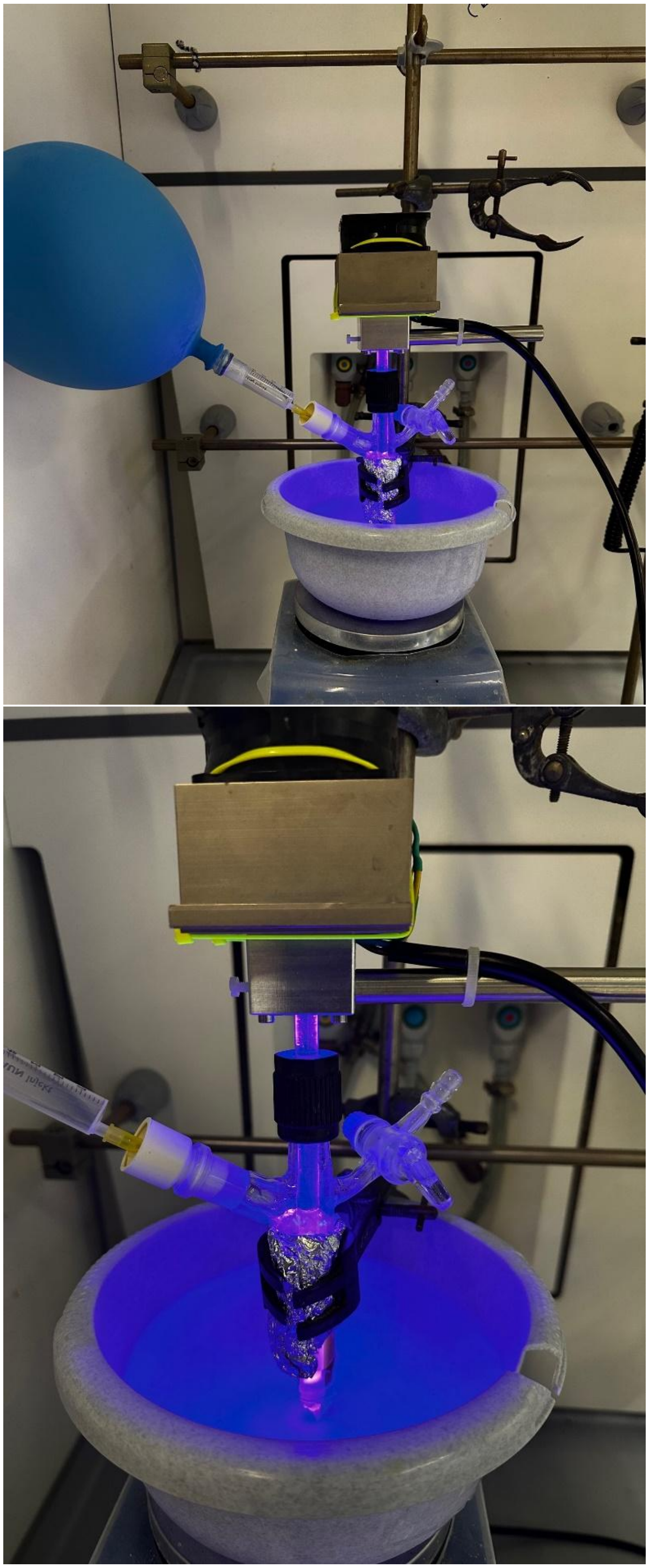

Fig. S2: Typical setup for a photochemical fluorination reaction.

S2. Analytical Methods

Thin layer chromatography (TLC) was performed on silica coated glass plates (silica gel 60 F_{254}) with detection by UV-light (λ = 254 nm) and potassium permanganate stain [$KMnO_4$].

Infrared spectra (IR) were recorded with *MicroLab* on an Agilent Technologies Cary 630 FTIR spectrometer by ATR technique. The signal intensity is assigned using the following abbreviations: s (strong), m (medium), w (weak). The following abbreviations were used: aliph = aliphatic, arom = aromatic.

$KMnO_4$-staining solution: Potassium permanganate (3.00 g), potassium carbonate (20.0 g), aqueous NaOH solution (5%, 5.0 mL), water (300 mL).

2,4-Dinitrophenylhydrazine (DNP)-staining solution: 2,4-Dinitrophenylhydrazine (12.0 g), H_2SO_4 (98%, 80 mL), aqueous NaOH solution (5%, 5.00 mL), EtOH (200 mL).

Melting points (M.p.) were determined using a Kofler ("Thermopan", Fs *Reichert*, Wien) apparatus.

Nuclear magnetic resonance (NMR) (^{1}H, ^{13}C and ^{19}F-NMR) spectra were recorded at room temperature (r.t.) on either a *Bruker* AVHD-400, AVHD-500, or a *Bruker* AV-II-500 equipped with cryo probe head. Chemical shifts of the NMR spectra are reported relative to $CHCl_3$ (^{1}H-NMR: δ = 7.26 ppm, ^{13}C-NMR: δ = 77.16 ppm), MeOH (^{1}H-NMR: δ = 3.31 ppm, ^{13}C-NMR: δ = 49.00 ppm) or DMSO (^{1}H-NMR: δ = 2.50 ppm, ^{13}C-NMR: δ = 39.52 ppm). The data are reported as follows: chemical shift (δ) [multiplicity, coupling constant *J* (Hz), relative integral, number of protons] where multiplicity is defined as: m = multiplet, s = singlet, d = doublet, t = triplet, q = quartet, br = broad, bs = broad singlet. Apparent multiplets which occur as a result of coupling constant equality between magnetically non-equivalent protons are marked as virtual (*virt.*). COSY methods were used to establish atom connectivities.

Mass spectrometry (MS) and **high-resolution mass spectrometry (HRMS)** were measured on a *Thermo Scientific* LTQ-FT Ultra (ESI). Thermo Scientific LTQ-FT Ultra is a hybrid instrument that combines two distinct mass analyzer types: a Linear Ion Trap (LTQ XL) front-end and a Fourier Transform Ion Cyclotron Resonance (FT-ICR) mass analyzer.

Specific Rotation was determined using an ADP440+ polarimeter (Fa *Bellingham+Stanley*) and is reported as follows: $[\alpha]_D^T$ (c in g per 100 mL solvent). The polarimeter has a variance of ± 0.001 which translates to a variance of ± 2 of the measured rotation for c = 2.0 and a cuvette path length of 0.5 cm.

Luminescence spectroscopy was performed on a Horiba Scientific FluoroMax-4P spectrofluorometer equipped with a continuous Xe source for steady state spectra and a Xe flashlight source for the observation of phosphorescence spectra. Spectra were recorded in Horiba quartz tubes (inner ⌀ = 4 mm) in a small quartz Dewar vessel.

S3. General Synthetic Procedures

General Procedure A (GP A): Synthesis of Aliphatic Carboxylic Acids 1

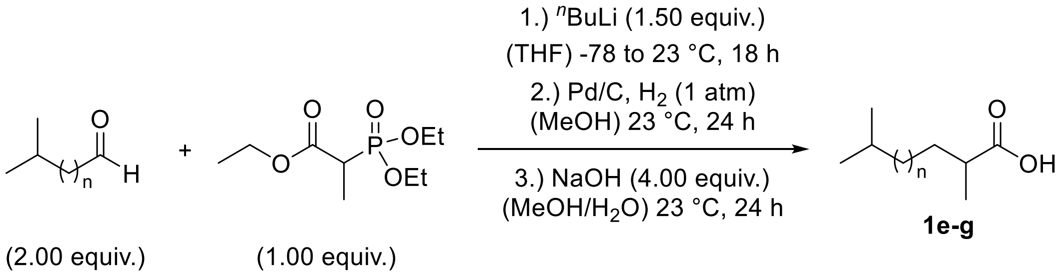

*n*Butyl lithium (*n*BuLi) (2.61 mL, 6.00 mmol, 2.30 M in hexane, 1.50 equiv.) was added dropwise to a solution of ethyl 2-(diethoxyphosphoryl) propanoate (858 µL, 4.00 mmol, 1.00 equiv.) in THF (16 mL, 250 mM) at −78 °C under *Schlenk* conditions. The acetone/dry ice bath was replaced by an ice bath and aldehyde (8.00 mmol, 2.00 equiv.) was added dropwise to the solution. Subsequently, the ice bath was removed, and the reaction mixture was stirred for 18 h at 23 °C. The reaction mixture was filtered through a Silica plug and concentrated under reduced pressure.

Without further purification, the crude material was subjected to a suspension of palladium on charcoal (Pd/C) (42.6 mg, 400 µmol, 0.10 equiv.) in MeOH (35 mL, 114 mM). H_2 gas was bubbled through the solution for 15 minutes and the reaction mixture was stirred for 24 h at 23 °C under H_2 atmosphere (balloon). Then, the reaction mixture was filtered through Celite and concentrated under reduced pressure.

A solution of NaOH (16 mmol, 4.00 equiv.) in water (4 mL, 4.0 M) was added to a solution of the crude material in MeOH (16 mL, 250 mM) and stirred for 24 h at 23 °C. The reaction mixture was concentrated under reduced pressure, diluted with water (15 mL), extracted with EtOAc (3 × 15 mL), dried over Na_2SO_4, filtered and concentrated under reduced pressure to yield the desired carboxylic acid **1e-g** as colorless oils.

General Procedure B (GP B): Photofluorination of carboxylic acids 1

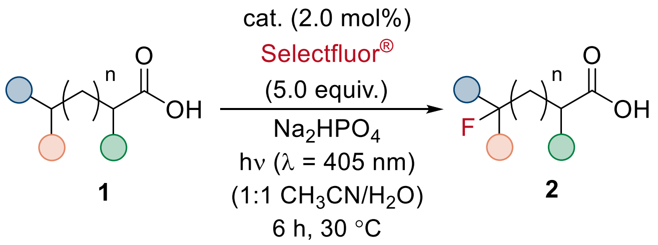

A solution of photocatalyst (8.00 µmol, 2 mol%), carboxylic acid **1** (400 µmol, 1.00 equiv.), Selectfluor® (709 mg, 2.00 mmol, 5.00 equiv.) and Na_2HPO_4 (62.5 mg, 440 µmol, 1.10 equiv.) in a mixture of acetonitrile/water (4 mL, 1:1 v/v) was degassed in an LED tube by sparging argon for 10 minutes, then irradiated with a 10 W blue LED (λ = 405 nm) for 12 h. After the irradiation, the reaction mixture was acidified with 1 (N) HCl until pH 1 and diluted with Et_2O (10 mL). The organic layer was separated, and the aqueous layer was extracted with Et_2O (3 × 15 mL). The combined organic phase was washed with brine, dried over Na_2SO_4, and concentrated under reduced pressure at 30 °C and the products were subjected to flash column chromatography (FCC) (SiO_2) to afford **2**.

General Procedure C (GP C): Photofluorination of carboxylic acids 1

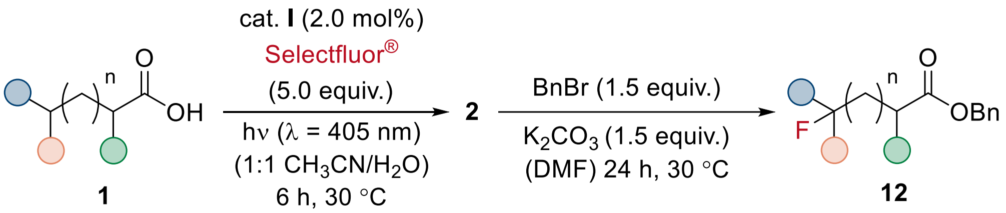

A solution of photocatalyst (8.00 µmol, 2 mol%), carboxylic acid **1** (400 µmol, c = 100 mM, 1.00 equiv.) and Selectfluor® (709 mg, 2.00 mmol, 5.00 equiv.) in a mixture of acetonitrile/water (4 mL, 1:1 v/v) was degassed in an LED tube by sparging argon for 10 minutes, then irradiated with a 10 W blue LED (λ = 405 nm) for 6 h. After the reaction completion, the crude reaction mixture was extracted with Et_2O (3 × 15 mL). The combined organic layers were washed with brine, dried over Na_2SO_4, and concentrated under reduced pressure.

If not stated otherwise, K_2CO_3 (82.9 mg, 600 µmol, 1.50 equiv.) and benzyl bromide (BnBr) (71.3 µL, 600 µmol, 1.50 equiv.) were added to a solution of the resulting crude mixture in dimethyl formamide (DMF) (5 mL, 0.08 M), and the reaction mixture was stirred at 23 °C for 24 h.

The suspension was diluted with Et_2O and washed with H_2O (3 × 15 mL), the organic layer washed with brine, dried over Na_2SO_4, and concentrated under reduced pressure and the products were subjected to FCC (SiO_2, EtOAc/pentane) to afford the fluorinated esters **12** as colorless oil.

General Procedure D (GP D): Photofluorination of carboxylic acids 1

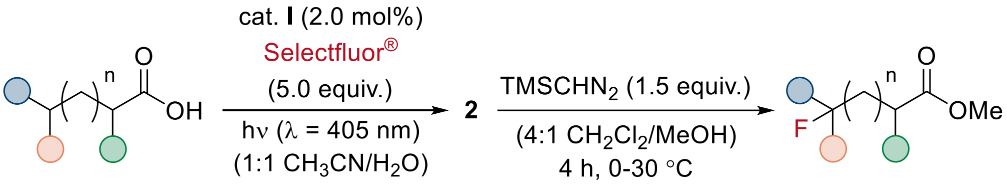

1 **13**

A solution of photocatalyst (8.00 µmol, 2 mol%), carboxylic acid **1** (400 µmol, c = 100 mM, 1.00 equiv.) and Selectfluor® (709 mg, 2.00 mmol, 5.00 equiv.) in a mixture of acetonitrile/water (4 mL, 1:1 v/v) was degassed in an LED tube by sparging argon for 10 minutes, then irradiated with a 10 W blue LED (λ = 405 nm) for 6 h. After the reaction completion, the crude reaction mixture was extracted with Et_2O (3 × 15 mL). The combined organic layers were washed with brine, dried over Na_2SO_4, and concentrated under reduced pressure.

Subsequently, (trimethylsilyl)diazomethane (300 µL, 600 µmol, 1.50 equiv.) was added dropwise to a solution of the crude reaction mixture in DCM/MeOH (5 mL, 4:1, 0.08 M) at 0 °C. After the reaction mixture was stirred for 4 h at 30 °C, unreacted excess of (trimethylsilyl)diazomethane was quenched with AcOH. The reaction mixture was concentrated under reduced pressure and the obtained crude product was then purified by FCC (SiO_2) to afford **13**.

S4. Optimization of the Reaction Conditions

Table S1: Initial Optimization of the reaction conditions for the fluorination of 4-methylpentanoic acid (**1a**). All reactions were performed on a 0.40 mmol scale.

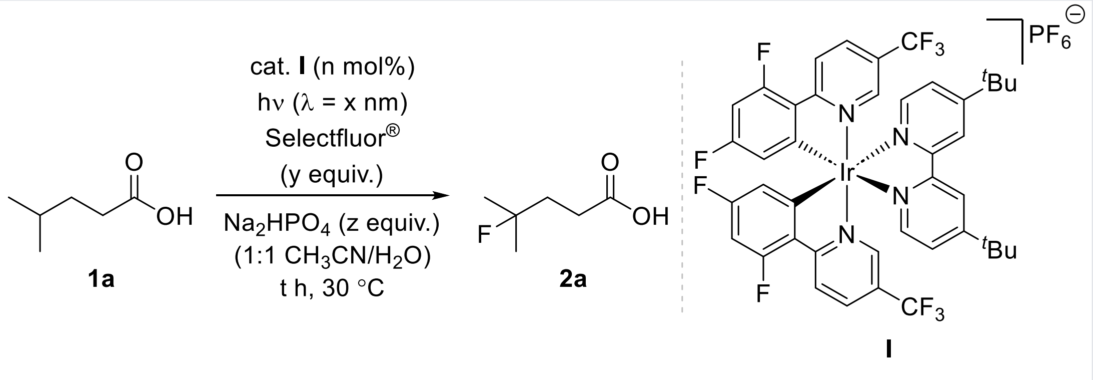

Entry	n (mol%)	x (nm)	y (equiv.)	z (equiv.)	t (h)	**2a**:**1a**
1	1.0	415	2.0	1.5	6	9:91
2	**0.5**	415	2.0	1.5	6	3:97
3	**2.0**	415	2.0	1.5	6	15:85
4	1.0	415	2.0	1.5	**12**	33:67
5	1.0	415	2.0	**1.1**	6	17:83
6	1.0	**405**	2.0	1.5	6	29:71
7	1.0	**425**	2.0	1.5	6	9:91
8	1.0	**398**	2.0	1.5	6	2:98
9	1.0	415	**3.0**	1.5	6	20:80
10	1.0	415	**4.0**	1.5	6	53:47
11	**2.0**	**405**	**4.0**	**1.1**	**12**	62:38
12	2.0	405	**5.0**	1.1	12	91:9
13	**-**	405	5.0	1.1	12	0:100
14	2.0	**-**	5.0	1.1	12	0:100
15	2.0	405	**-**	1.1	12	0:100
16	2.0	405	5.0	**-**	12	98:2

Table S2: Optimization of the reaction conditions for the fluorination of 4-methylpentanoic acid (**1a**). All reactions were performed on a 0.40 mmol scale.

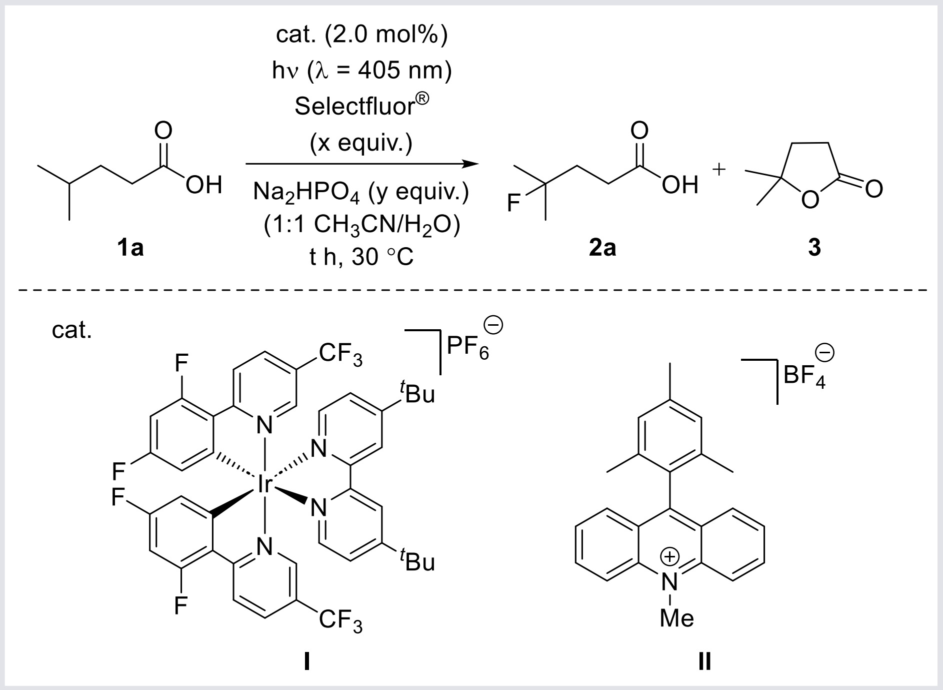

Entry	cat.	x (equiv.)	y (equiv.)	t (h)	**2a**:**3**:**1a**	yield (%)
1	**I**	5.0	1.1	12	8.7:0.5:1	53
2	**II**	5.0	1.1	12	9:1:0.1	48
3	-	5.0	1.1	12	0:0:1	-
4[b]	**I** or **II**	5.0	1.1	12	0:0:1	-
5	**I** or **II**	-	1.1	12	0:0:1	-
6	**I**	5.0	1.1	18	1:99:0	<2%
7	**I**	5.0	-	12	50:6:1	77
8	**II**	5.0	-	12	14.5:3.9:0.1	73
9	**I**	5.0	-	6	43:2.2:1	93

[a]The reactions were performed on a 0.40 mmol scale using 4 mL 1:1 mixture of CH_3CN/H_2O. For cat. **I** 10 W 405 nm and for cat. **II** 10 W 437 nm blue LEDs were used. Yields correspond to the ^{1}H-NMR yields using $PhCF_3$ as an external standard. [b]The reaction was carried out without light.

S5. Control Experiments

Lactonization study:

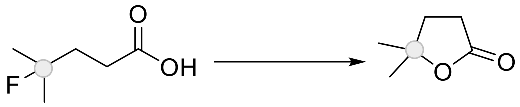

2a **3**

During our optimization studies along with the desired product 4-fluoro-4-methylpentanoic acid **2a**, the corresponding lactone 5,5-dimethyldihydrofuran-2(3*H*)-one (**3**) was also detected in the crude reaction mixture by ^{1}H-NMR as a byproduct. This observation suggested that the fluorinated product **2a** might be unstable under the reaction conditions. To substantiate this suspicion, we irradiated the reaction mixture for 18 h instead of 12 h and **3** was formed exclusively. The lactonization of the **2a** was also observed after the workup of the reaction mixture. Moreover, pure **2a** was also found to be prone towards lactonization under ambient conditions. A neat sample of pure **2a** underwent lactonization within 72 h of isolation under the ambient conditions. These observations led us to believe that the γ-fluorinated acid **2a** readily cyclizes to the corresponding lactone **3** under both reaction and ambient conditions.[1,2]

¹H-NMR immediately after purification.

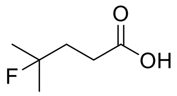

2a

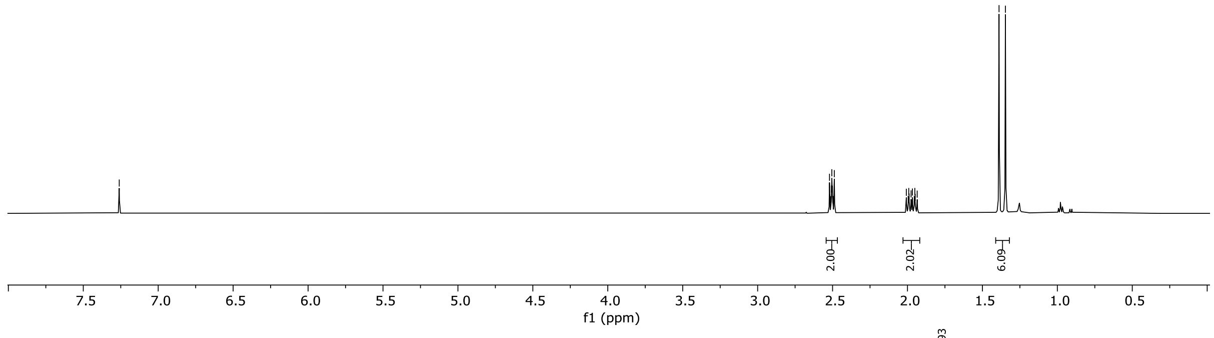

¹⁹F-NMR immediately after purification.

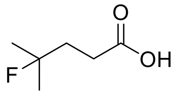

2a

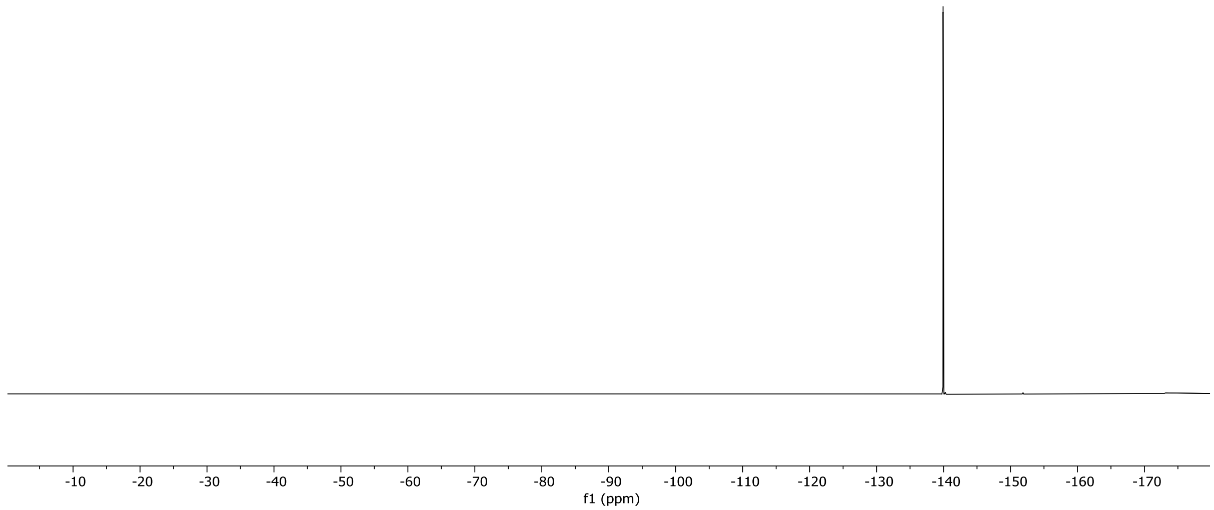

^{1}H-NMR after 72 h of purification.

3

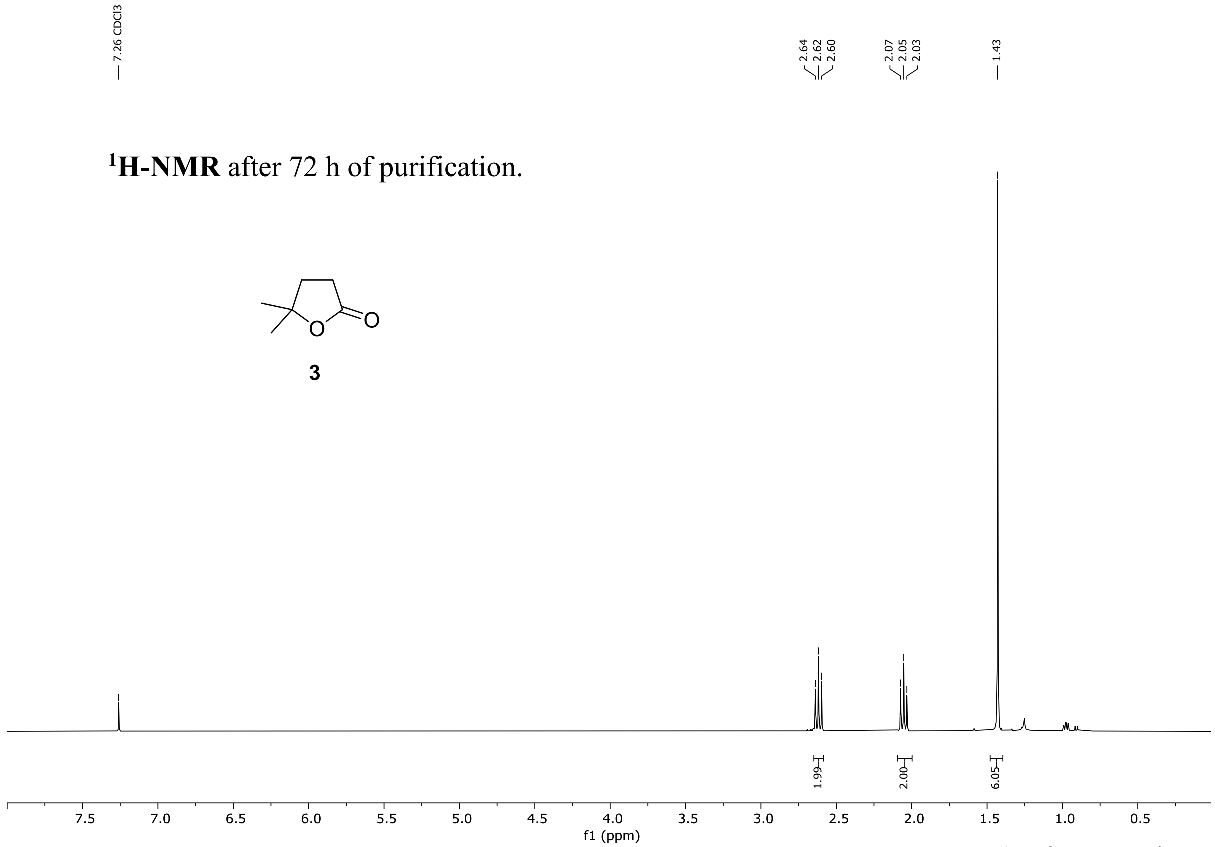

^{19}F-NMR after 72 h of purification.

3

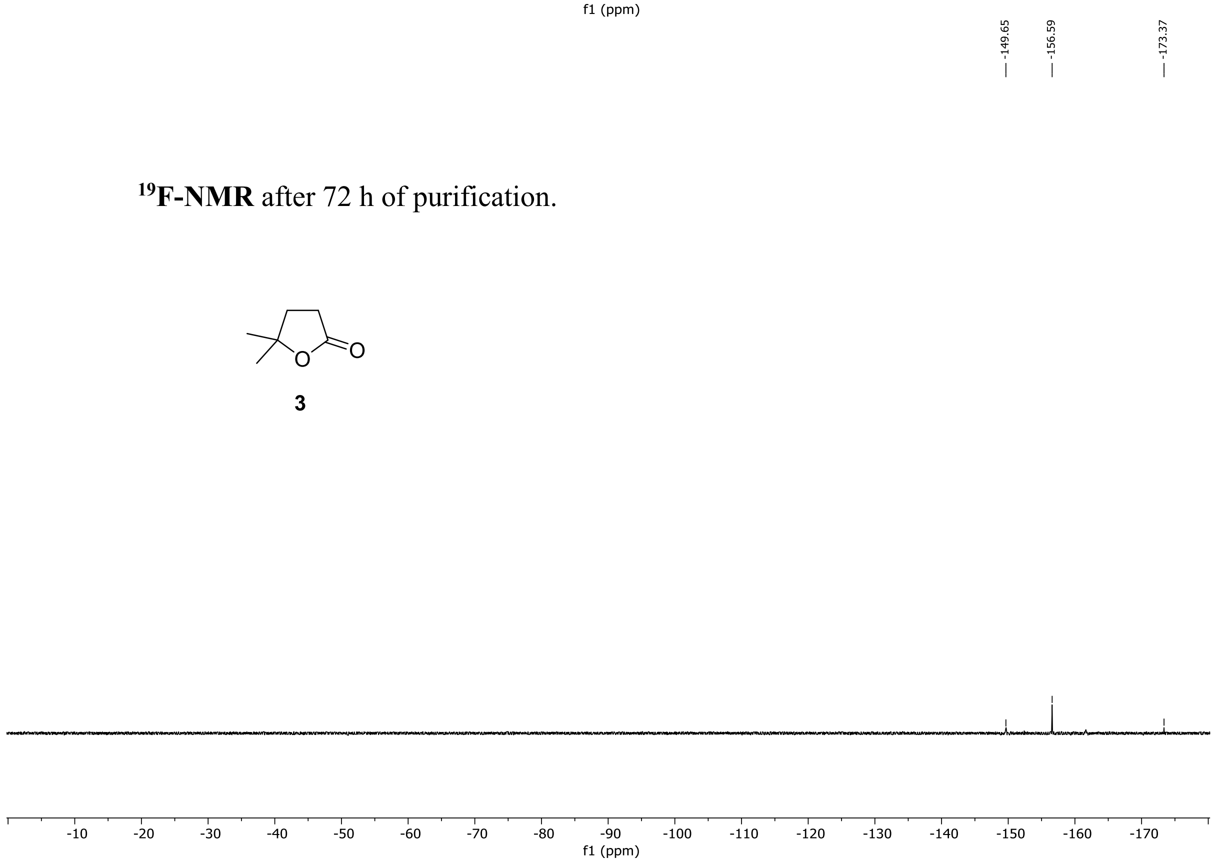

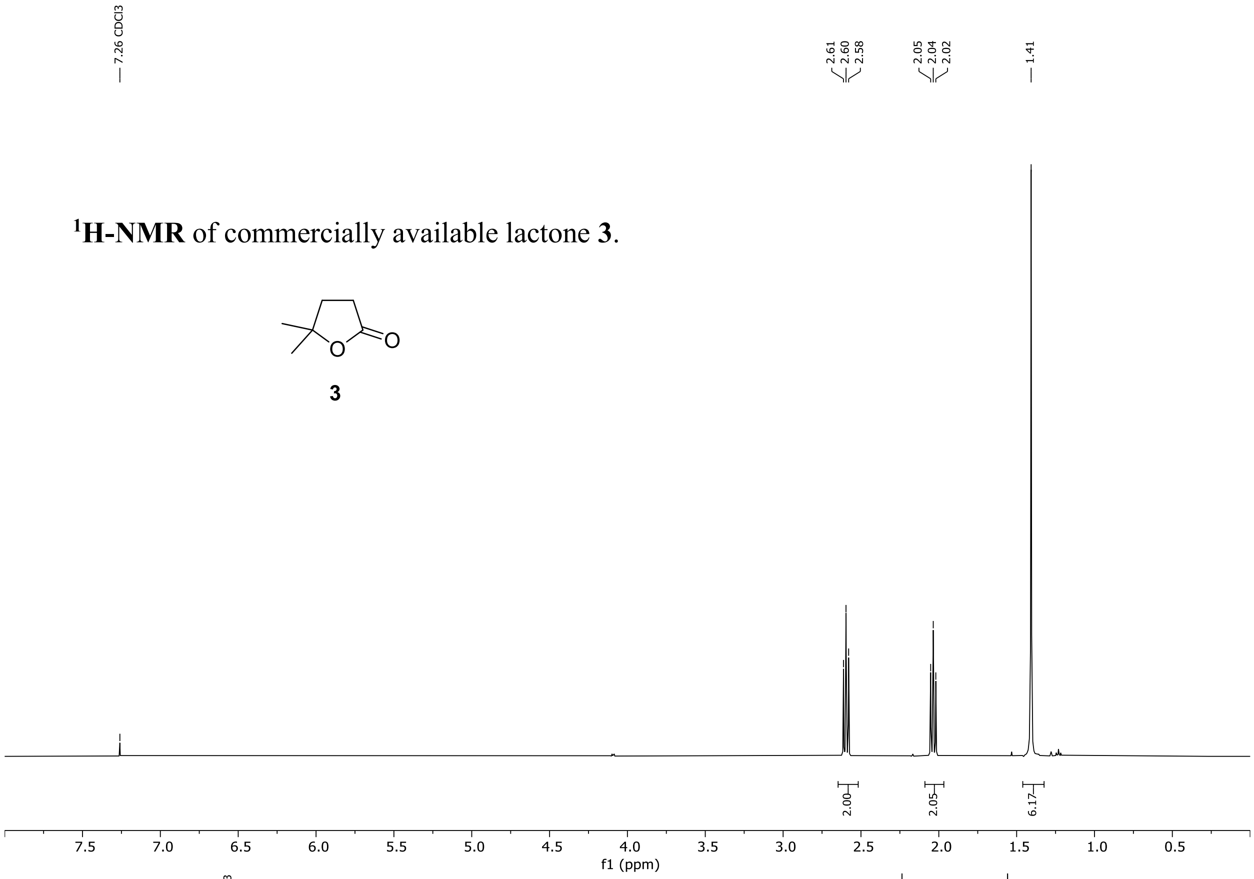

^{1}H-NMR of commercially available lactone **3**.

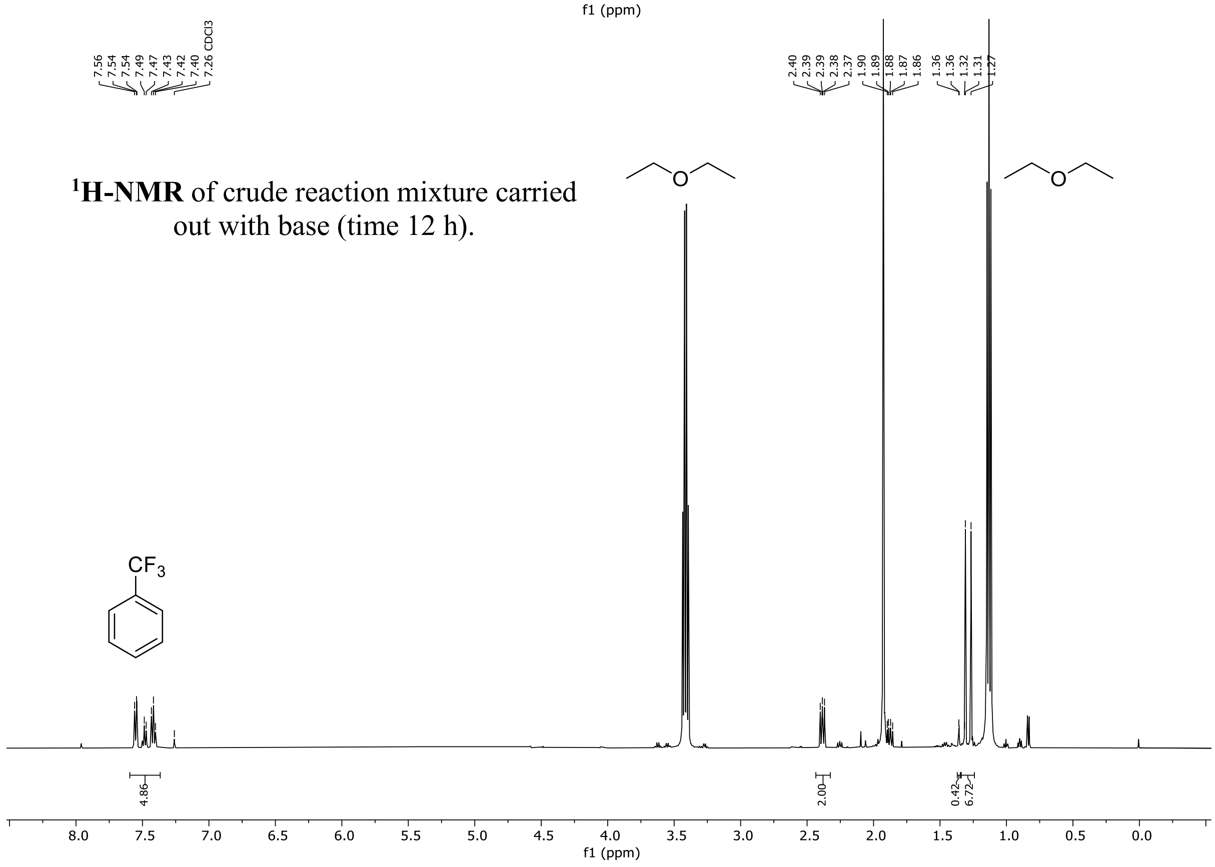

^{1}H-NMR of crude reaction mixture carried out with base (time 12 h).

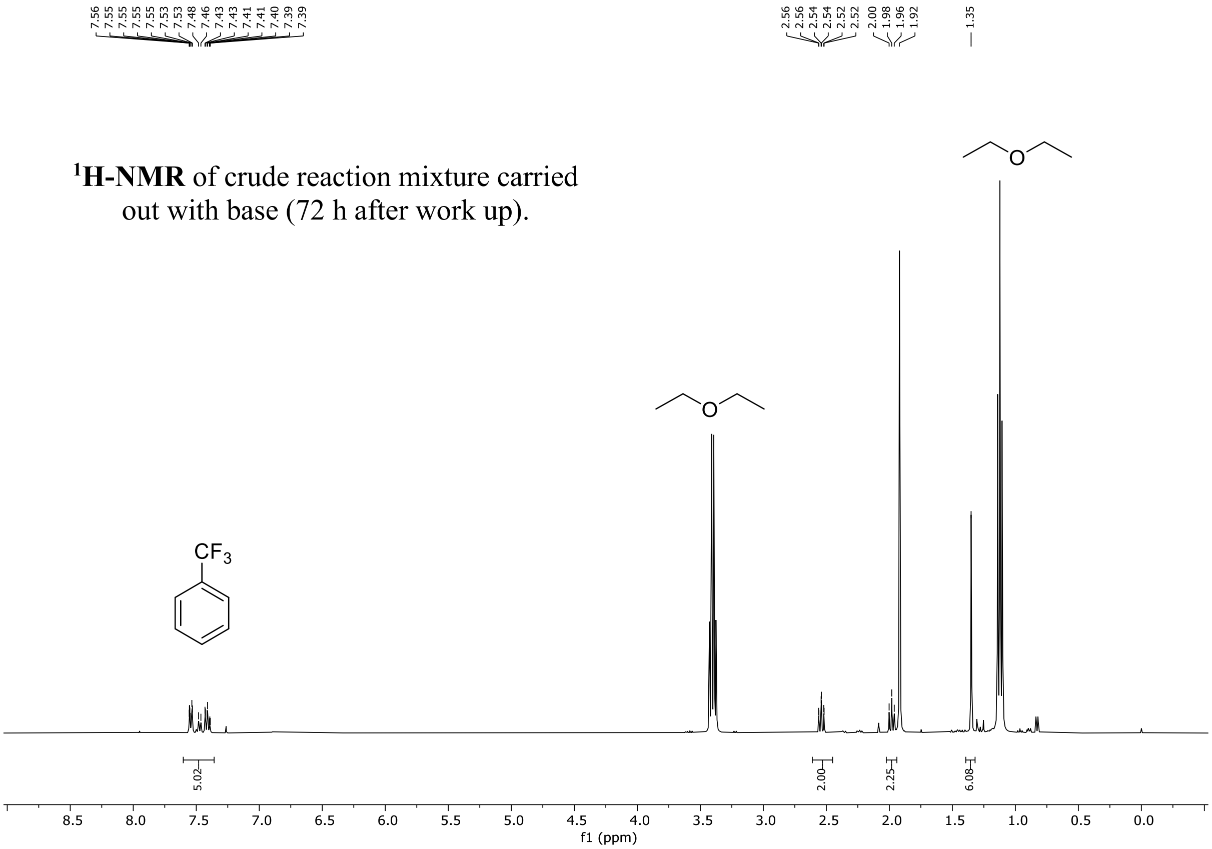

7.56
7.55
7.55
7.55
7.55
7.53
7.53
7.48
7.46
7.43
7.43
7.41
7.40
7.39
7.39
2.56
2.56
2.54
2.54
2.52
2.52
2.00
1.98
1.96
1.92
1.35
^{1}H-NMR of crude reaction mixture carried out with base (72 h after work up).
O
CF_3
5.02
2.00
2.25
6.08
8.5 8.0 7.5 7.0 6.5 6.0 5.5 5.0 4.5 4.0 3.5 3.0 2.5 2.0 1.5 1.0 0.5 0.0
f1 (ppm)

Competition experiment:

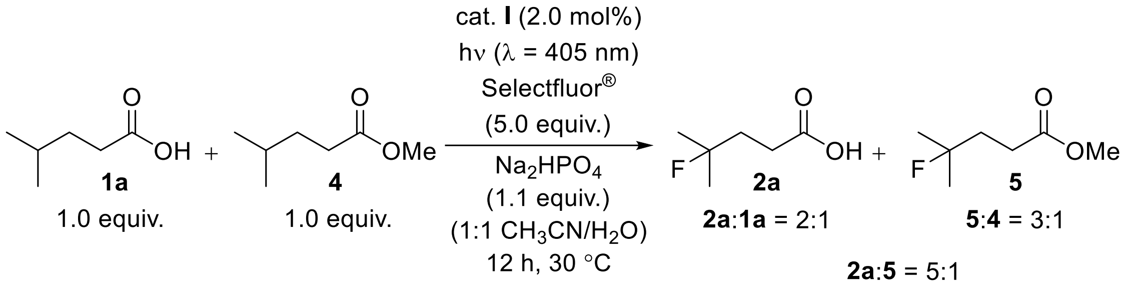

According to the GP B, a solution of photocatalyst Ir[dF(CF$_3$)ppy]$_2$(dtbbpy)PF$_6$ **I** (8.98 mg, 8.00 µmol, 2 mol%), 4-methylpentanoic acid (**1a**) (46.5 mg, 400 µmol, 1.00 equiv.), methyl 4-methylpentanoate (**4**) (52.7 mg, 400 µmol, 1.00 equiv.), Selectfluor® (709 mg, 2.00 mmol, 5.00 equiv.) and Na$_2$HPO$_4$ (62.5 mg, 440 µmol, 1.10 equiv.) in a mixture of acetonitrile/water (4 mL, 1:1 v/v) was degassed in an LED tube by sparging argon for 10 minutes, then irradiated with a 10 W blue LED (λ = 405 nm) for 12 h. After the irradiation, the reaction mixture was acidified with 1 (N) HCl until pH 1 and diluted with Et$_2$O (10 mL). The organic layer was separated, and the aqueous layer was extracted with Et$_2$O (3 × 15 mL). The combined organic phase was washed with brine, dried over Na$_2$SO$_4$, and concentrated under reduced pressure at 30 ºC.[3]

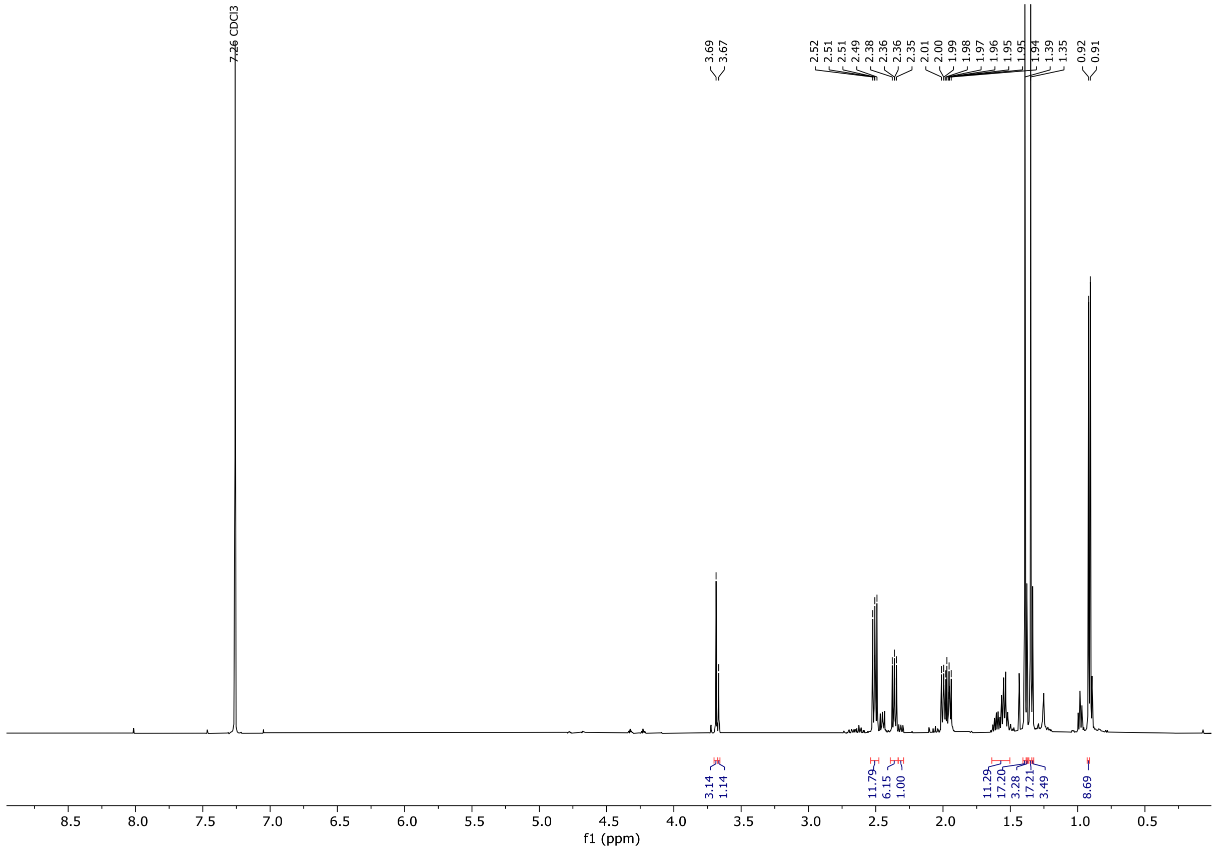
7.26 CDCl3
f1 (ppm)

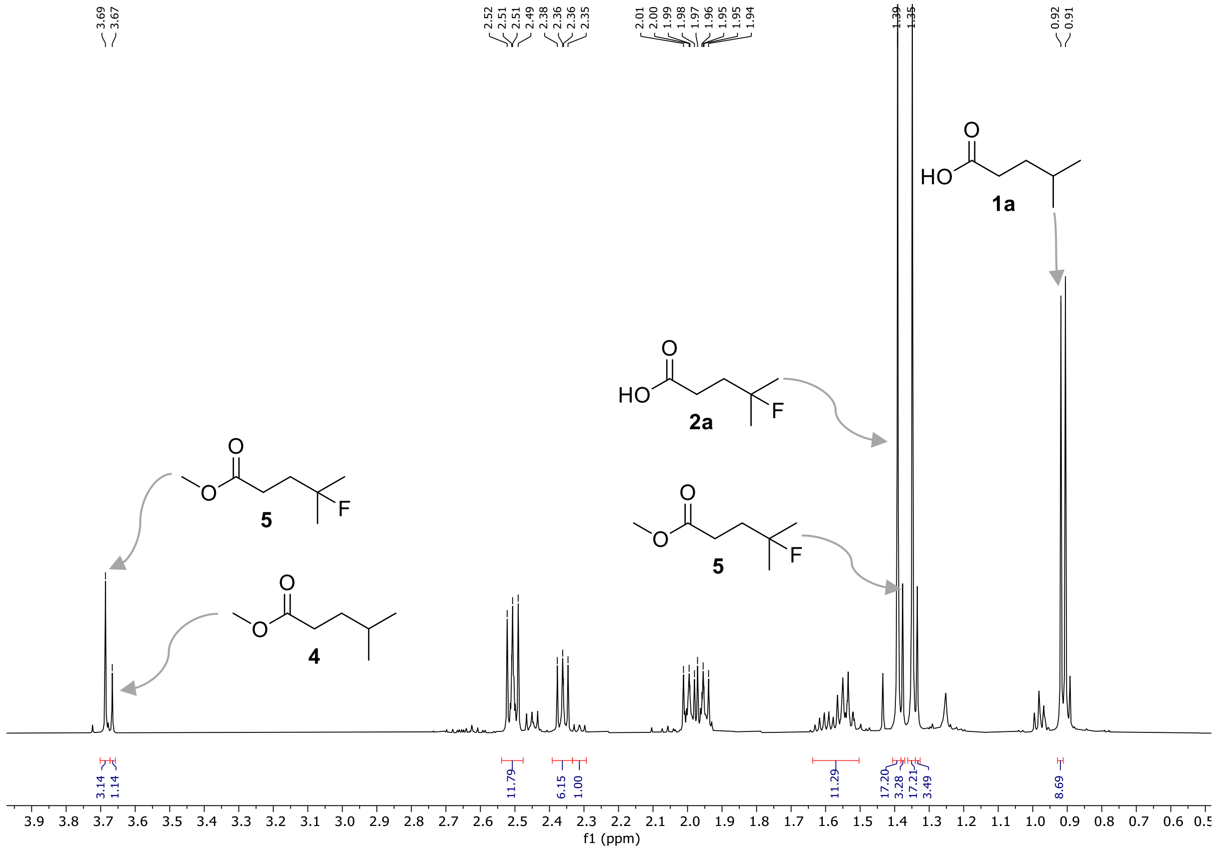
HO
1a
HO
F
2a
O
F
5
O
F
5
O
4
f1 (ppm)

Decarboxylation Study:

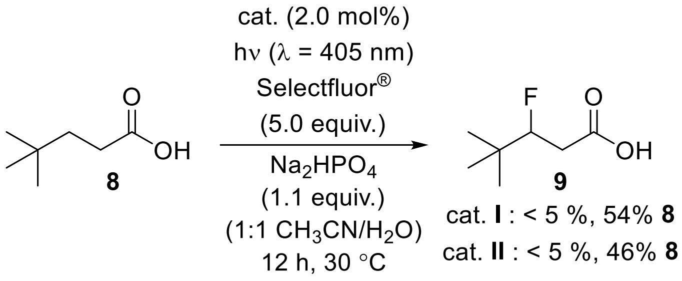

According to the GP B, A solution of photocatalyst Ir[dF(CF_3)ppy]$_2$(dtbbpy)PF_6 **I** or 9-mesityl-10-methylacridinium tetrafluoroborate (**II**) (8.00 µmol, 2 mol%), methyl 4,4-dimethylpentanoate (**8**) (52.7 mg, 400 µmol, 1.00 equiv.), Selectfluor® (709 mg, 2.00 mmol, 5.00 equiv.) and Na_2HPO_4 (62.5 mg, 440 µmol, 1.10 equiv.) in a mixture of acetonitrile/water (4 mL, 1:1 v/v) was degassed in an LED tube by sparging argon for 10 minutes, then irradiated with a 10 W blue LED (λ = 405 nm for cat. **I** and 437 nm for cat. **II**) for 12 h. After the irradiation, the reaction mixture was acidified with 1 N HCl until pH 1 and diluted with Et_2O (10 mL). The organic layer was separated, and the aqueous layer was extracted with Et_2O (3 × 15 mL). The combined organic phase was washed with brine, dried over Na_2SO_4, and concentrated under reduced pressure at 30 ºC followed by the addition of 0.50 equiv. of $PhCF_3$. The mixture was subjected to NMR using $CDCl_3$ as the solvent.

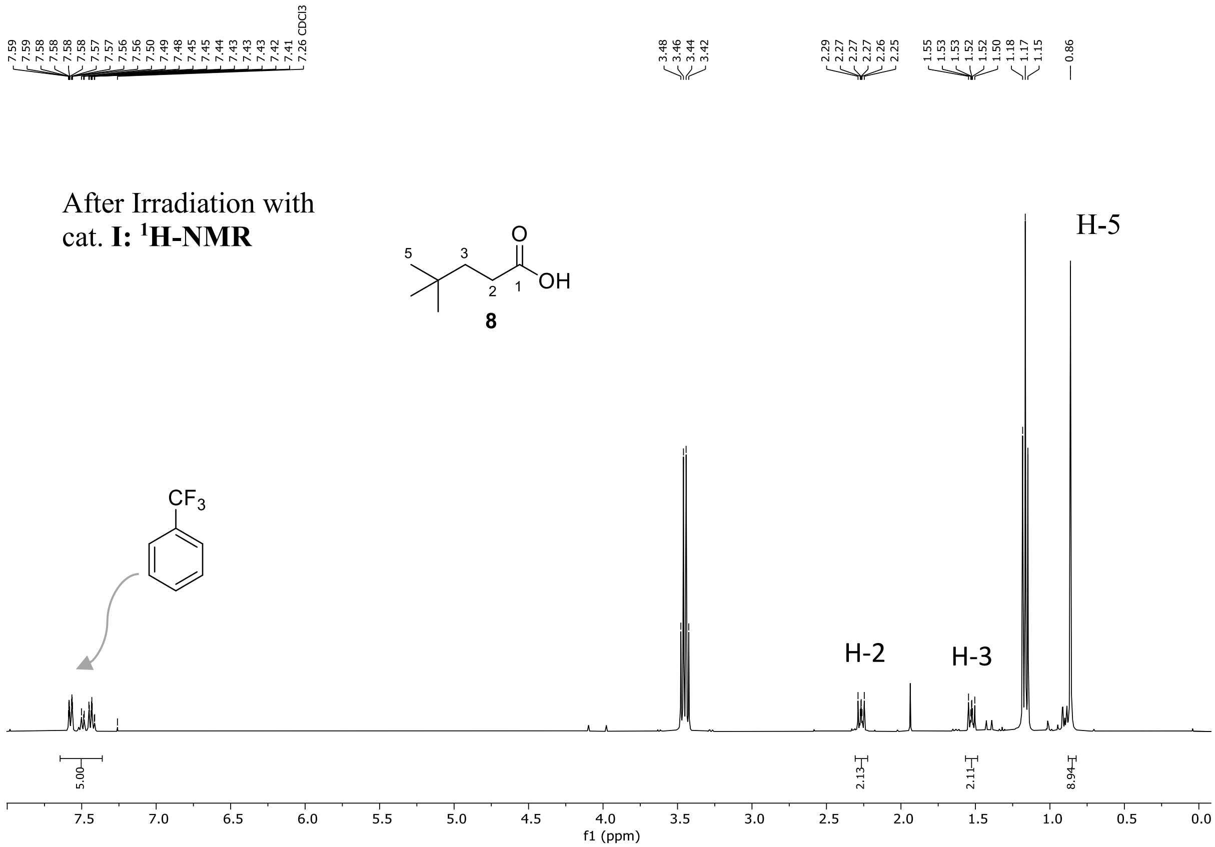
After Irradiation with
cat. I: 1H-NMR
8
H-2
H-3
H-5
CF3
f1 (ppm)

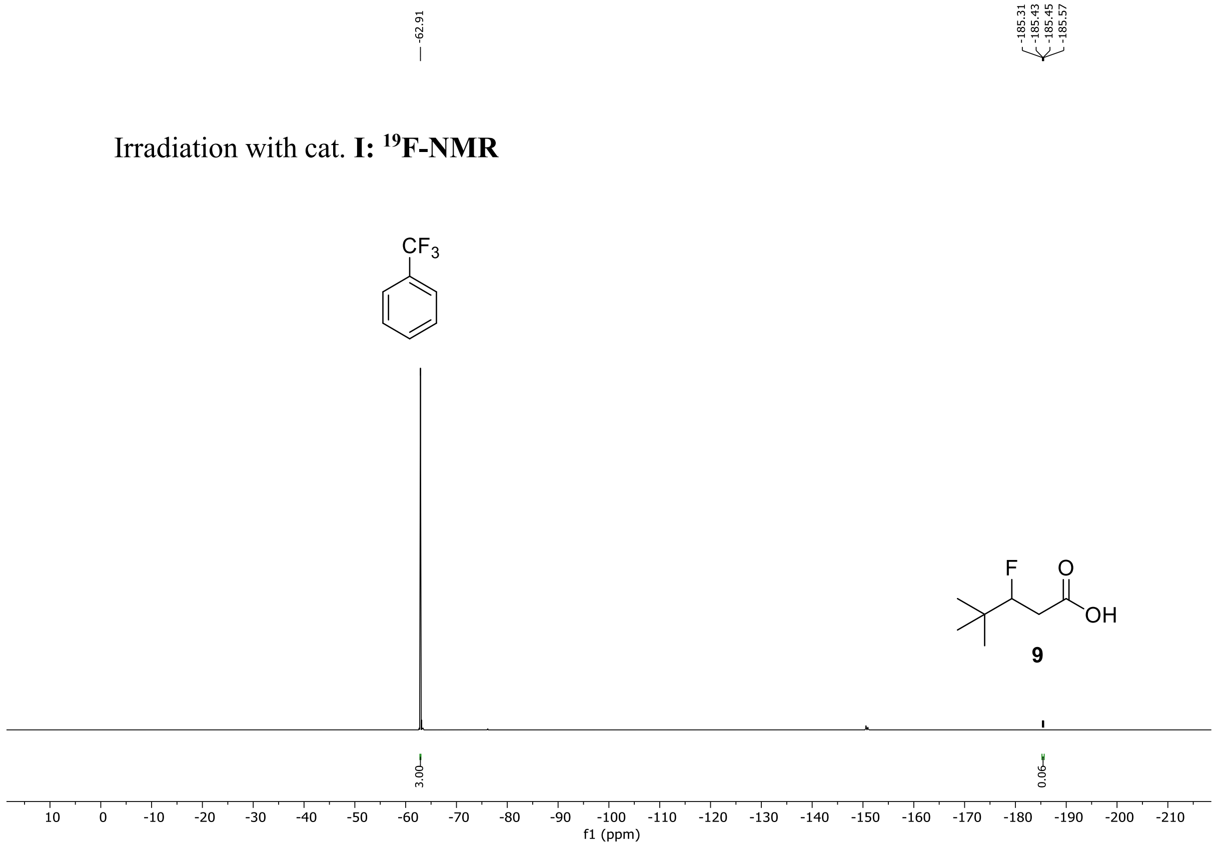
Irradiation with cat. I: 19F-NMR
CF3
9
f1 (ppm)

Irradiation with cat. **II:** [1]**H-NMR**

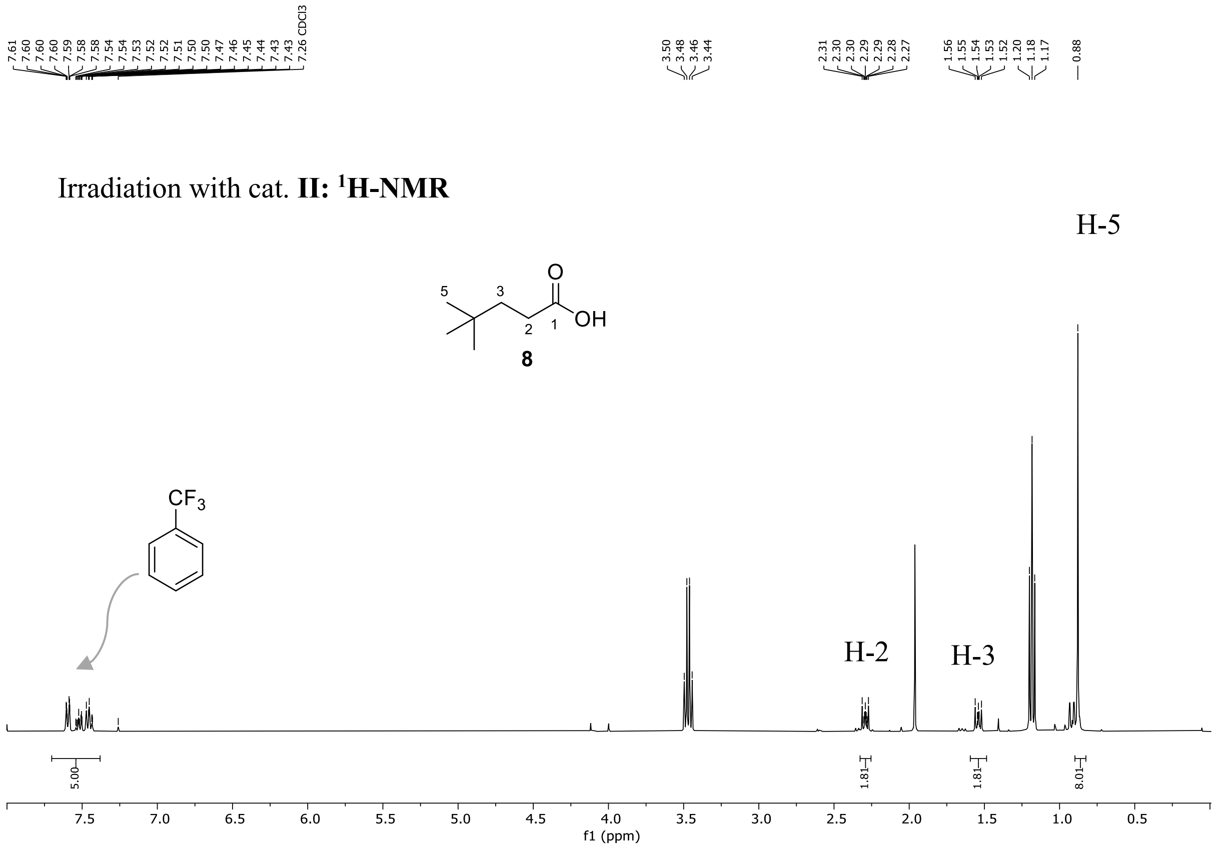

Irradiation with cat. **II:** [19]**F-NMR**

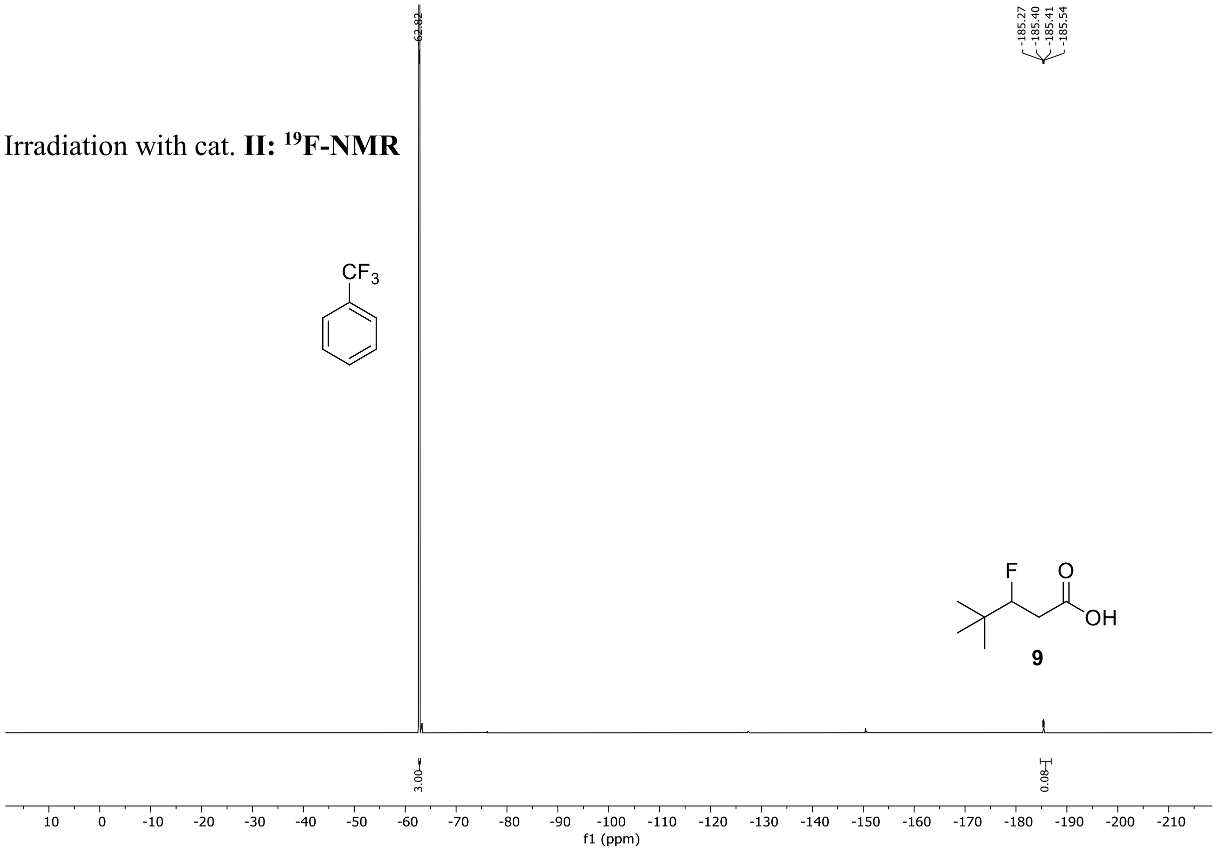

Stern-Volmer quenching experiment:

As the yield of the reaction was similar with both photocatalyst Ir[dF(CF_3)ppy]$_2$(dtbbpy)PF_6 **I** or 9-mesityl-10-methylacridinium tetrafluoroborate (**II**), we decided to perform a semi-quantitative Stern-Volmer quenching experiment with the stronger photo oxidant (cat. **II**).

Procedure:

A screw-top quartz cuvette was charged with a 1.15×10^{-4} M solution solution of **II** in MeCN (1.5 mL) and the initial fluorescence emission was measured at ambient temperature (25 °C). Subsequently, the appropriate amount of the quencher in MeCN was added. The sample was sonicated for 5 min and then the emission of the sample was measured.[4]

The solutions were excited at 425 nm, and the emission intensity was collected at 507 nm.

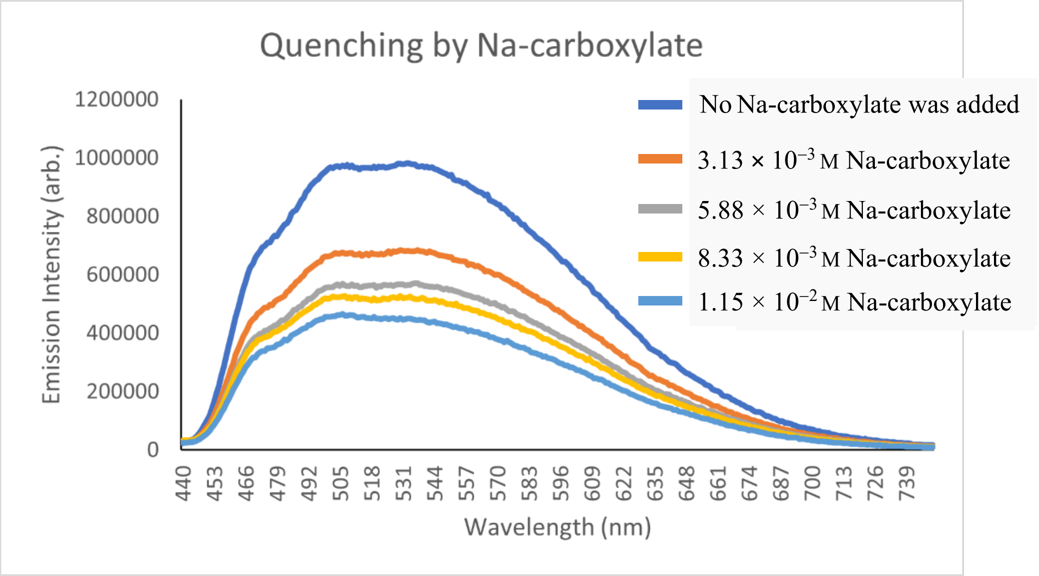

Fig. S1: Fluorescence quenching of cat. **II** by Na-carboxylate solution

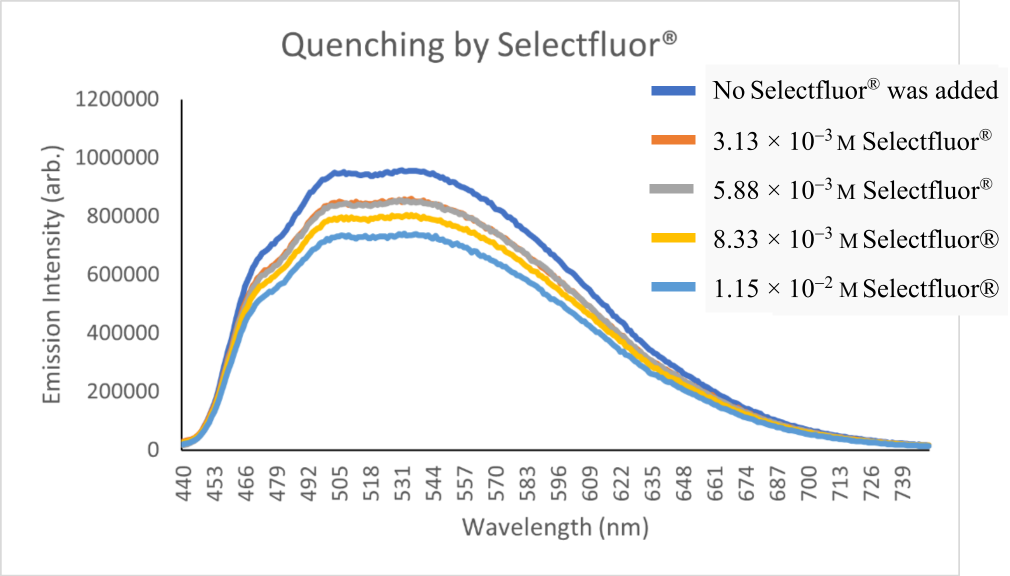

Fig. S2: Fluorescence quenching of cat. **II** by Selectfluor®

Table S3: Data for the Stern-Volmer quenching study with cat. **II**

Entry	Concentration of quencher [M]	I_0/I Na-carboxylate	I_0/I Selectfluor®
1	0	1	1
2	0.003125	1.441	1.119
3	0.00588	1.708	1.13
4	0.00833	1.845	1.194
5	0.011538	2.09	1.295

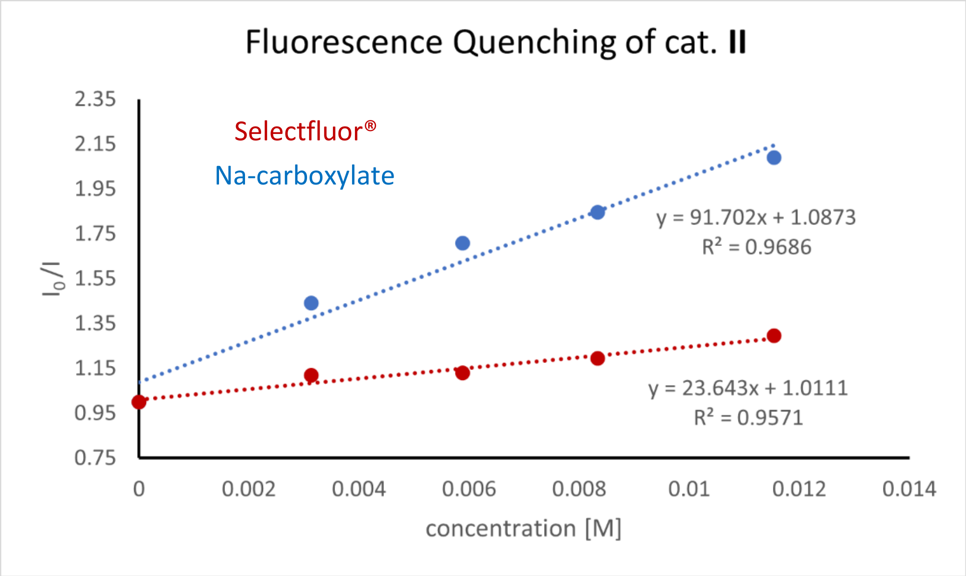

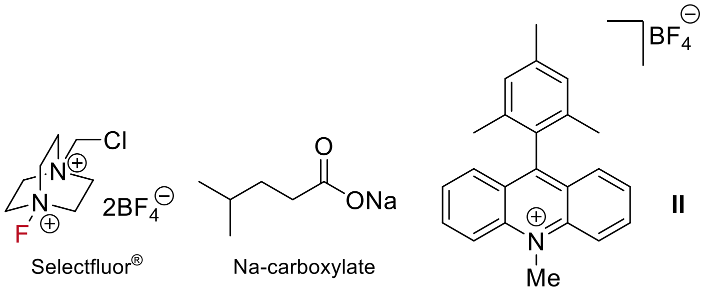

Selectfluor® Na-carboxylate **II**

Quenching rate constants were obtained using the Stern-Volmer relationship assuming dynamic quenching of the fluorescence emission:

$$I_0/I=1+k_q\tau_0[\text{Quencher}]$$

The intrinsic fluorescence lifetime τ_0 of the photocatalyst was taken as = 7.3×10^{-9} s in acetonitrile solution as reported previously by Romero and Nicewicz.[5] The quenching rate constant k_q was calculated from the Stern-Volmer constant as given in Table S3.

Cat.	quencher	k_q ($M^{-1}s^{-1}$)
**II**	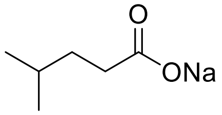 Na-carboxylate	1.26×10^{10}
**II**	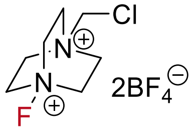 Selectfluor®	3.24×10^{9}

From the quenching rate constant (k_q) of Na-carboxylate, it was clear that the oxidation of carboxylate is possible by cat. **II**. However, we also see the quenching by Selectfluor®. In addition, the rate of quenching was 3.89-fold faster by Na-carboxylate than Selectfluor®. However, as we are using 5.00 equiv. of Selectfluor® in the reaction, we hypothesize that the quenching/oxidation of the Cat. **II** by Selectfluor® will outcompete the oxidation of Na-carboxylate by cat. **II**.

Failed substrates:

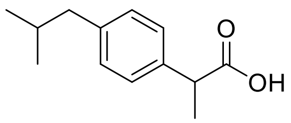

starting material consumed
complex reaction mixture formed

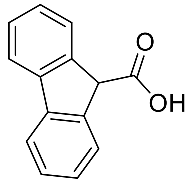

starting material consumed
complex reaction mixture formed

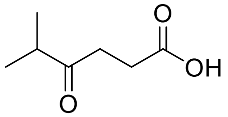

conversion <5%

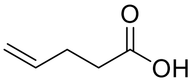

conversion <5%

S6. Synthesis and Characterization of Carboxylic acids 1

2,4-Dimethylpentanoic acid (1e)

According to GP A, 2-methylpropanal (730 µL, 8.00 mmol, 2.00 equiv.) was converted to yield 2,4-dimethylpentanoic acid (**1e**) (104 mg, 799 µmol, 20%) as a colorless oil over three steps.

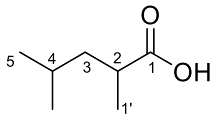

1e

$C_7H_{14}O_2$

MW: 130.19 gmol^{-1}

^{1}H-NMR (400 MHz, $CDCl_3$, 300 K): δ [ppm] = 2.60 – 2.47 (m, 1H, H2), 1.72 – 1.56 (m, 2H, H3), 1.31 – 1.19 (m, 1H, H4), 1.17 (d, $^3J_{H\text{-}H}$ = 7.0 Hz, 3H, H1'), 0.92 (d, $^3J_{H\text{-}H}$ = 6.3 Hz, 3H, H5), 0.89 (d, $^3J_{H\text{-}H}$ = 6.3 Hz, 3H, H5).

^{13}C-NMR (101 MHz, $CDCl_3$, 300 K): δ [ppm] = 183.8 (C1), 42.9 (C3), 37.6 (C2), 26.0 (C4), 22.6 (C5), 22.6 (C5), 17.4 (C1').

HRMS (ESI) *m/z* [M−H]$^-$ calculated for $[C_7H_{13}O_2]^-$: 129.0921; found: 129.0908.

IR (ATR): $\tilde{\nu}$ [cm^{-1}] = 3032 (m, O–H), 2956, 2935, 2872 (m, C_{aliph}–H), 1702 (s, C=O), 1467 (m, C–H), 1237, 1206 (s, C–O).

2,5-Dimethylhexanoic acid (1g)

According to GP A, 3-methylbutanal (878 µL, 8.00 mmol, 2.00 equiv.) was converted to yield the desired 2,5-dimethylhexanoic acid (**1g**) (118 mg, 818 µmol, 23%) as a colorless oil over three steps.

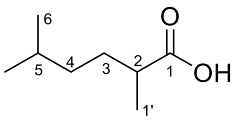

1g

$C_8H_{16}O_2$

MW: 144.21 gmol^{-1}

^{1}H-NMR (400 MHz, $CDCl_3$, 300 K): δ [ppm] = 2.43 (*virt.* sextet, $^3J_{H\text{-}H} \cong {}^3J_{H\text{-}H}$ = 7.0 Hz, 1H, H2), 1.77 – 1.63 (m, 1H), 1.60 – 1.36 (m, 2H, H3), 1.25 – 1.19 (m, 2H, H4), 1.18 (d, $^3J_{H\text{-}H}$ = 7.0 Hz, 3H, H1'), 0.88 (d, $^3J_{H\text{-}H}$ = 6.6 Hz, 6H, H6).

^{13}C-NMR (101 MHz, $CDCl_3$, 300 K): δ [ppm] = 183.7 (C1), 39.8 (C2), 36.4 (C4), 31.5 (C3), 28.2 (C5), 22.7 (C6), 22.6 (C6), 17.0 (C1').

HRMS (ESI) *m/z* [M−H]$^-$ calculated for $[C_8H_{15}O_2]^-$: 143.1078; found: 143.1065.

IR (ATR): $\tilde{\nu}$ [cm^{-1}] = 2500–3300 (m, O–H), 2956, 2927, 2872 (m, C_{aliph}–H), 1702 (s, C=O), 1466, 1414, 1385 (m, C_{aliph}–H), 1290, 1232 (s, C–O).

(*S*)-5-Methyl-3-(2,2,2-trifluoroacetamido)hexanoic acid (1i)

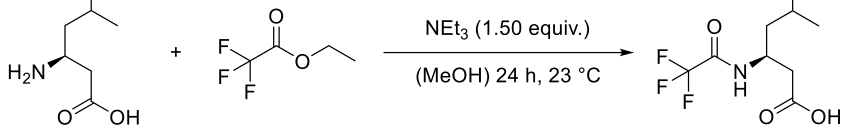

(1.00 equiv.) (1.30 equiv.) **1i**

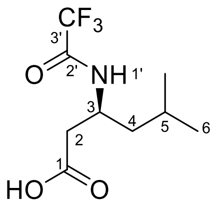

1i
$C_9H_{14}F_3NO_3$
MW: 241.21 gmol^{-1}

Ethyl trifluoroacetate (320 µL, 2.69 mmol, 1.30 equiv.) was added dropwise to a solution of triethylamine (432 µL, 3.10 mmol, 1.50 equiv.) and 3-amino-5-methylhexanoic acid (300 mg, 2.07 mmol, 1.00 equiv.) in MeOH (5 mL, 534 mM). The reaction mixture was stirred for 24 h at 23 °C. The solvent was concentrated under reduced pressure, upon which the crude was dissolved in H_2O (15 mL) and acidified with concentrated HCl to pH = 2. Subsequently, the mixture was extracted with EtOAc (3 × 25 mL), and the combined organic layers were washed with brine (20 mL) and dried over Na_2SO_4. After removing the solvent under reduced pressure, the desired product (*S*)-5-methyl-3-(2,2,2-trifluoroacetamido)hexanoic acid (**1i**) (455 mg, 1.89 mmol) was afforded in 91% yield as a white solid.

^{1}H-NMR (400 MHz, $CDCl_3$, 300 K): δ [ppm] = 9.51 (s, 1H, COO–H), 7.02 (d, $^3J_{H\text{-}H}$ = 9.6 Hz, 1H, N–H), 4.38 (tq, $^3J_{H\text{-}H}$ = 9.6, 5.1 Hz, 1H, H3), 2.76 – 2.57 (m, 2H, H2), 1.68 – 1.51 (m, 2H, H4), 1.48 – 1.35 (m, 1H, H5), 0.94 (d, $^3J_{H\text{-}H}$ = 6.5 Hz, 3H, H6), 0.94 (d, $^3J_{H\text{-}H}$ = 6.5 Hz, 3H, H6).

^{13}C-NMR (101 MHz, $CDCl_3$, 300 K): δ [ppm] = 176.8 (C1), 157.1 (q, $^2J_{C\text{-}F}$ = 37.1 Hz, C2'), 115.9 (q, $^1J_{C\text{-}F}$ = 287.7 Hz, C3'), 45.2 (C3), 42.7 (C2), 38.0 (C4), 25.1 (C5), 22.8 (C6), 22.0 (C6).

^{19}F NMR (376 MHz, $CDCl_3$, 300 K): δ [ppm] = −76.07.

HRMS (ESI) *m/z* [M−H]$^-$ calculated for $[C_9H_{13}F_3NO_3]^-$: 240.0853; found: 240.0847.

IR (ATR): $\tilde{\nu}$ [cm^{-1}] = 3309 (s, N–H), 2500–3100 (m, O–H), 2962, 2875 (m, C_{aliph}–H) , 1712 (s, C=O acid/amide stacked), 1215, 1157 (s, C–F), 1184 (s, C–O).

S7. Photofluorination Reactions of Carboxylic Acids 1

4-Fluoro-4-methylpentanoic acid (2a)

According to GP C, a solution of Ir[dF(CF_3)ppy]$_2$(dtbbpy)PF_6 **I** (8.98 mg, 8.00 µmol, 2 mol%), 4-methylpentanoic acid (**1a**) (50.3 µL , 400 µmol, 1.00 equiv.) and Selectfluor® (709 mg, 2.00 mmol, 5.00 equiv.) in a mixture of acetonitrile/water (4 mL, 1:1 v/v) was degassed in an LED tube by sparging argon for 10 minutes, then irradiated with a 10 W blue LED (λ = 405 nm) for 6 h. After the irradiation, the reaction mixture was diluted with Et_2O (10 mL). The organic layer was separated, and the aqueous layer was extracted with Et_2O (3 × 15 mL). The combined organic phase was washed with brine, dried over Na_2SO_4, and concentrated under reduced pressure at 30 ºC. The obtained crude product was then purified by flash column chromatography FCC (SiO_2, pentane/EtOAc = 98:2, 2% AcOH) to yield 4-fluoro-4-methylpentanoate (**2a**) as a colorless oil (44 mg, 328 µmol, 83%).

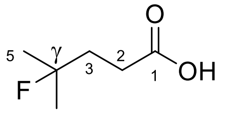

2a
$C_6H_{11}FO_2$
MW: 134,15 gmol^{-1}

TLC (pentane/EtOAc = 98:2, 2% AcOH): R_f = 0.16 [UV, $KMnO_4$].

^{1}H-NMR (400 MHz, CD_2Cl_2, 300 K): δ [ppm] = 2.54 – 2.46 (m, 2H, H2), 2.03 – 1.89 (m, 2H, H3), 1.36 (d, $^3J_{H\text{-}F}$ = 21.4 Hz, 6H, H5).

^{13}C-NMR (101 MHz, CD_2Cl_2, 300 K): δ [ppm] = 178.7 (C1), 94.9 (d, $^1J_{C\text{-}F}$ = 166.0 Hz, C4), 36.2 (d, $^2J_{C\text{-}F}$ = 23.0 Hz, C3), 29.0 (d, $^3J_{C\text{-}F}$ = 4.8 Hz, C2), 26.7 (d, $^2J_{C\text{-}F}$ = 24.7 Hz, C5).

^{19}F NMR (376 MHz, CD_2Cl_2, 300 K): δ [ppm] = −141.05.

The spectroscopic data matches the values reported in the literature.[1]

5,5-Dimethyldihydrofuran-2(3*H*)-one (3)

According to GP B, a solution of Ir[dF(CF_3)ppy]$_2$(dtbbpy)PF_6 **I** (8.98 mg, 8.00 µmol, 2 mol%), 4-methylpentanoic acid (**1a**) (50.3 µL, 400 µmol, 1.00 equiv.), Selectfluor® (709 mg, 2.00 mmol, 5.00 equiv.) and Na_2HPO_4 (62.5 mg, 440 µmol, 1.10 equiv.) in a mixture of acetonitrile/water (4 mL, 1:1 v/v) was degassed in an LED tube by sparging argon for 10 minutes, then irradiated with a 10 W blue LED (λ = 405 nm) for 18 h. After the irradiation, the reaction mixture was acidified with 1 (N) HCl until pH 1 and diluted with Et_2O (10 mL). The organic layer was separated, and the aqueous layer was extracted with Et_2O (3 × 15 mL). The combined organic phase was washed with brine, dried over Na_2SO_4, and concentrated under reduced pressure at

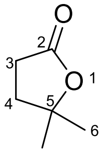

3
$C_6H_{10}O_2$
MW: 114.14 gmol^{-1}

30 ºC. The obtained crude product was then purified by flash column chromatography FCC (SiO_2, hexane/EtOAc = 92:8) to yield 5,5-dimethyldihydrofuran-2(3*H*)-one (**3**) as a colorless oil (28 mg, 245 µmol, 61%).

TLC (hexane/EtOAc = 90:10): R_f = 0.2 [$KMnO_4$].

^{1}H-NMR (400 MHz, $CDCl_3$, 300 K): δ [ppm] = 2.62 (t, $^3J_{H\text{-}H}$ = 8.3 Hz, 2H, H3), 2.05 (t, $^3J_{H\text{-}H}$ = 8.3 Hz, 2H, H4), 1.43 (s, 6H, H6).

^{13}C-NMR (101 MHz, $CDCl_3$, 300 K): δ [ppm] = 176.9 (C2), 84.8 (C5), 34.9 (C4), 29.5 (C3), 27.9 (C6).

The spectroscopic data matches the values reported in the literature.[2]

4-Fluoro-4-methylpentanamide (7)

According to GP B, a solution of Ir[dF(CF_3)ppy]$_2$(dtbbpy)PF_6 **I** (8.98 mg, 8.00 µmol, 2 mol%), 4-methylpentanamide (50.1 µL, 400 µmol, 1.00 equiv.), Selectfluor® (709 mg, 2.00 mmol, 5.00 equiv.) and Na_2HPO_4 (62.5 mg, 440 µmol, 1.10 equiv.) in a mixture of acetonitrile/water (4 mL, 1:1 v/v) was degassed in an LED tube by sparging argon for 10 minutes, then irradiated with a 10 W blue LED (λ = 405 nm) for 12 h. After the irradiation, the reaction mixture was acidified with 1 (N) HCl till pH 1 and diluted with Et_2O (10 mL). The organic layer was separated, and the aqueous layer was extracted with Et_2O (3 × 15 mL). The combined organic phase was washed with brine, dried over Na_2SO_4, and concentrated under reduced pressure at 30 ºC. The obtained crude product was then purified by flash column chromatography FCC (SiO_2, hexane/EtOAc = 50:50 → 30:70) to yield 4-fluoro-4-methylpentanamide (**7**) as a colorless sticky oil (39 mg, 293 µmol, 73%).

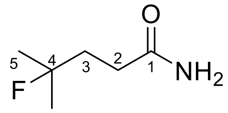

7
$C_6H_{12}FNO$
MW: 133.17 gmol^{-1}

TLC (hexane/EtOAc = 50:50): R_f = 0.29 [UV, $KMnO_4$].

^{1}H-NMR (400 MHz, $CDCl_3$, 300 K): δ [ppm] = 5.49 (bs, 2H, N−H), 2.41 – 2.32 (m, 2H, H2), 2.05 – 1.87 (m, 2H, H3), 1.37 (d, $^3J_{H\text{-}F}$ = 21.4 Hz, 6H, H5).

^{13}C-NMR (101 MHz, $CDCl_3$, 300 K): δ [ppm] = 174.9 (C1), 94.9 (d, $^1J_{C\text{-}F}$ = 166.5 Hz, C4), 36.3 (d, $^2J_{C\text{-}F}$ = 23.0 Hz, C3), 30.1 (C2), 26.6 (d, $^2J_{C\text{-}F}$ = 24.7 Hz, C5).

^{19}F NMR (376 MHz, CD_2Cl_2, 300 K): δ [ppm] = −140.97 (dp, $^2J_{H\text{-}F}$ = 42.2 Hz, $^3J_{H\text{-}F}$ = 21.1 Hz).

The spectroscopic data matches the values reported in the literature.[4]

Benzyl 4-fluoro-4-methylpentanoate (12a)

According to GP C, a solution of Ir[dF(CF_3)ppy]$_2$(dtbbpy)PF_6 **I** (8.98 mg, 8.00 µmol, 2 mol%), 4-methylpentanoic acid (**1a**) (50.3 µL , 400 µmol, 1.00 equiv.) and Selectfluor® (709 mg, 2.00 mmol, 5.00 equiv.) in a mixture of acetonitrile/water (4 mL, 1:1 v/v) was degassed in an LED tube by sparging argon for 10 minutes, then irradiated with a 10 W blue LED (λ = 405 nm) for 6 h. After the irradiation, the reaction mixture was diluted with Et_2O (10 mL). The organic layer was separated, and the aqueous layer was extracted with Et_2O (3 × 15 mL). The combined organic phase was washed with brine, dried over Na_2SO_4, and concentrated under reduced pressure at 30 ºC. Subsequently, the crude reaction mixture was dissolved in dimethyl formamide (DMF) (5 mL, 0.08 M) followed by the addition of K_2CO_3 (82.9 mg, 600 µmol, 1.50 equiv.) and benzyl bromide (BnBr) (71.3 µL, 600 µmol, 1.50 equiv.). The resulting mixture was stirred at 30 °C for 24 h. The suspension was diluted with Et_2O (15 mL) and washed with H_2O (3 × 10 mL). The organic layer washed with brine, dried over Na_2SO_4 and concentrated under reduced pressure. The obtained crude product was then purified by flash column chromatography FCC (SiO_2, pentane → pentane/EtOAc = 98:2) to yield benzyl 4-fluoro-4-methylpentanoate (**12a**) as a colorless oil (60.1 mg, 268 µmol, 67%).

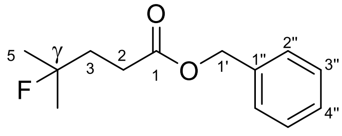

12a
$C_{13}H_{17}FO_2$
MW: 224.28 gmol^{-1}

TLC (pentane/EtOAc = 98:2): R_f = 0.23 [UV, $KMnO_4$].

^{1}H-NMR (400 MHz, $CDCl_3$, 300 K): δ [ppm] = 7.38 – 7.31 (m, 5H, H2”, H3”, H4”), 5.13 (s, 2H, H1’), 2.54 – 2.46 (m, 2H, H2), 2.07 – 1.92 (m, 2H, H3), 1.35 (d, $^3J_{H\text{-}F}$ = 21.3 Hz, 6H, H5).

^{13}C-NMR (101 MHz, $CDCl_3$, 300 K): δ [ppm] = 173.3 (C1), 136.1 (C1”), 128.7 (C3”), 128.4 (C2”, C4”), 94.7 (d, $^1J_{C\text{-}F}$ = 166.5 Hz, C4), 66.5 (C1’), 36.1 (d, $^2J_{C\text{-}F}$ = 23.0 Hz, C3), 29.1 (d, $^3J_{C\text{-}F}$ = 4.8 Hz, C2), 26.6 (d, $^2J_{C\text{-}F}$ = 24.7 Hz, C5).

^{19}F NMR (376 MHz, $CDCl_3$, 300 K): δ [ppm] = −140.96.

HRMS (ESI) *m/z* [M+H]$^+$ calculated for [$C_{13}H_{18}FO_2$]$^+$: 225.1285; found: 225.1279.

IR (ATR): $\tilde{\nu}$ [cm^{-1}] = 3066, 3034 (m, C_{ar}–H), 2980, 2932 (m, C_{aliph}–H), 1735 (s, C=O), 1608, 1586, 1498, 1456 (m, C_{ar}=C_{ar}), 1167 (s, C–O), 1126 (s, C–F).

On a 1.00 mmol scale:

According to GP C, a solution of Ir[dF(CF_3)ppy]$_2$(dtbbpy)PF_6 **I** (22.5 mg, 20.0 µmol, 2 mol%), 4-methylpentanoic acid (**1a**) (126 µL , 1.0 mmol, 1.00 equiv.) and Selectfluor® (1.77 g, 5.00 mmol,

5.00 equiv.) in a mixture of acetonitrile/water (6 mL, 1:1 v/v) was degassed in an LED tube by sparging argon for 20 minutes, then irradiated with a 10 W blue LED (λ = 405 nm) for 12 h. After the irradiation, the reaction mixture was diluted with Et_2O (15 mL). The organic layer was separated, and the aqueous layer was extracted with Et_2O (3 × 30 mL). The combined organic phase was washed with brine, dried over Na_2SO_4, and concentrated under reduced pressure at 30 ºC. Subsequently, the crude reaction mixture (for ^{1}H NMR, see below) was dissolved in dimethyl formamide (DMF) (12.5 mL, 0.08 M) followed by the addition of K_2CO_3 (207 mg, 1.50 mmol, 1.50 equiv.) and benzyl bromide (BnBr) (178 µL, 1.50 mmol, 1.50 equiv.). The resulting mixture was stirred at 30 °C for 24 h. The suspension was diluted with Et_2O (30 mL) and washed with H_2O (3 × 15 mL). The organic layer washed with brine, dried over Na_2SO_4 and concentrated under reduced pressure. The obtained crude product was then purified by flash column chromatography FCC (SiO_2, pentane → pentane/EtOAc = 98:2) to yield benzyl 4-fluoro-4-methylpentanoate (**12a**) as a colorless oil (160 mg, 714 µmol, 71%).

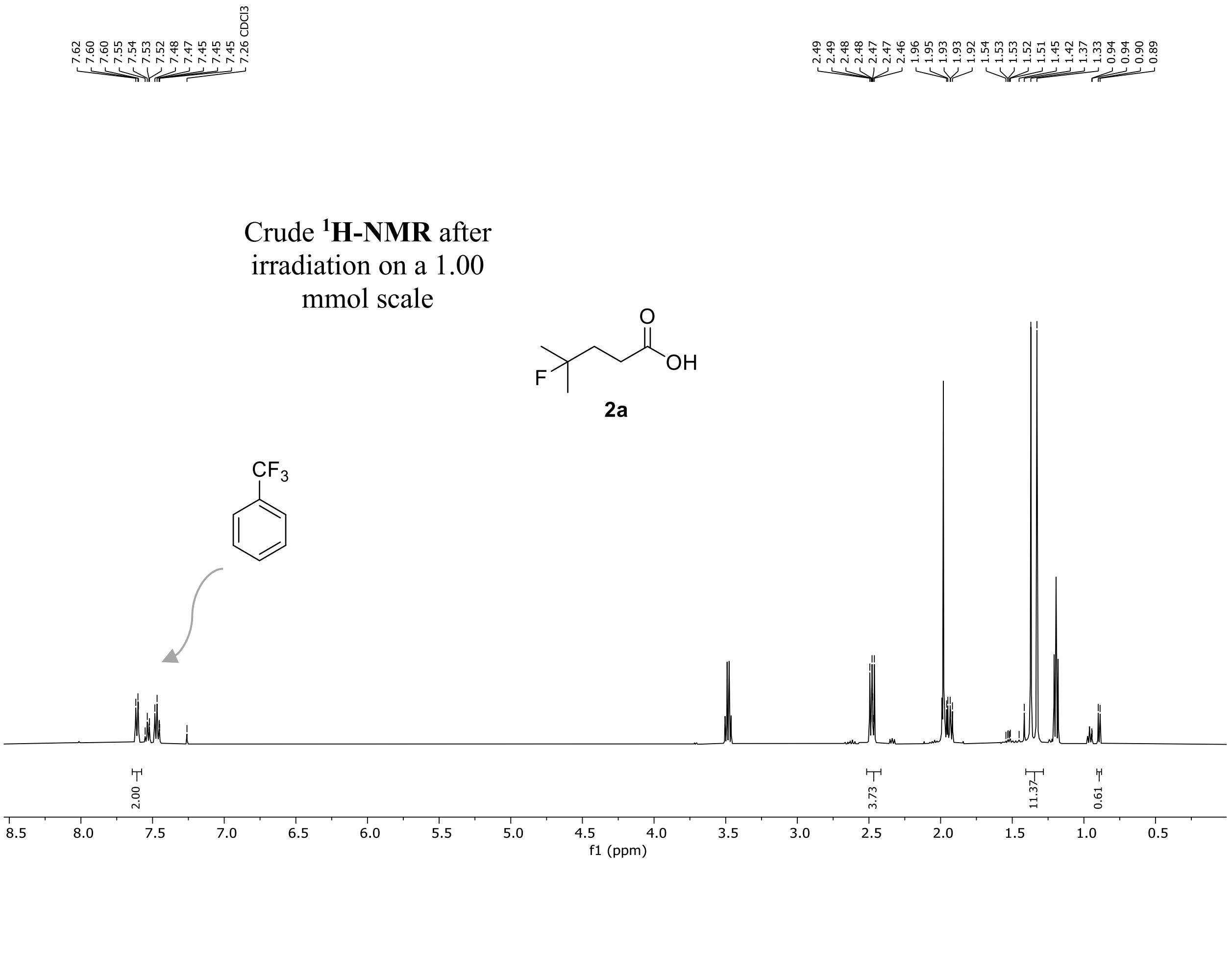

Benzyl 3-fluoro-3-methylbutanoate (12b)

According to GP C, a solution of Ir[dF(CF_3)ppy]$_2$(dtbbpy)PF_6 **I** (8.98 mg, 8.00 µmol, 2 mol%), 3-methylbutanoic acid (**1b**) (43.9 µL, 400 µmol, 1.00 equiv.) and Selectfluor® (709 mg, 2.00 mmol, 5.00 equiv.) in a mixture of acetonitrile/water (4 mL, 1:1 v/v) was degassed in an LED tube by sparging argon for 10 minutes, then irradiated with a 10 W blue LED (λ = 405 nm) for 6 h. After the irradiation, the reaction mixture was diluted with Et_2O (10 mL). The organic layer was separated, and the aqueous layer was extracted with Et_2O (3 × 15 mL). The combined organic phase was washed with brine, dried over Na_2SO_4, and concentrated under reduced pressure at 30 ºC. Subsequently, the crude reaction mixture was dissolved in dimethyl formamide (DMF) (5 mL, 0.08 M) followed by the addition of K_2CO_3 (82.9 mg, 600 µmol, 1.50 equiv.) and benzyl bromide (BnBr) (71.3 µL, 600 µmol, 1.50 equiv.). The resulting mixture was stirred at 30 °C for 24 h. The suspension was diluted with Et_2O (15 mL) and washed with H_2O (3 × 10 mL). The organic layer washed with brine, dried over Na_2SO_4 and concentrated under reduced pressure. The obtained crude product was then purified by flash column chromatography FCC (SiO_2, pentane → pentane/EtOAc = 98:2) to yield benzyl 3-fluoro-3-methylbutanoate (**12b**) as a colorless oil (38.5 mg, 183 µmol, 46%).

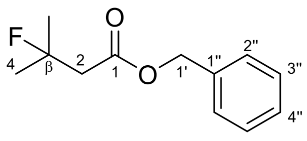

12b

$C_{12}H_{15}FO_2$

MW: 210.25 gmol^{-1}

TLC (pentane/EtOAc = 98:2): R_f = 0.23 [UV, $KMnO_4$].

^{1}H-NMR (400 MHz, $CDCl_3$, 300 K): δ [ppm] = 7.43 – 7.29 (m, 5H, H2”, H3”, H4”), 5.15 (s, 2H, H1’), 2.72 (d, $^3J_{H\text{-}F}$ = 16.0 Hz, 2H, H2), 1.49 (d, $^3J_{H\text{-}F}$ = 21.7 Hz, 6H, H4).

^{13}C-NMR (101 MHz, $CDCl_3$, 300 K): δ [ppm] = 169.7 (d, $^3J_{C\text{-}F}$ = 10.4 Hz, C1), 135.9 (C1”), 128.7 (C4”), 128.4 (C2”), 128.4 (C3”), 93.4 (d, $^1J_{C\text{-}F}$ = 169.5 Hz, C3), 66.6 (C1’), 46.2 (d, $^2J_{C\text{-}F}$ = 26.0 Hz, C2), 27.0 (d, $^2J_{C\text{-}F}$ = 23.8 Hz, C4).

^{19}F NMR (376 MHz, $CDCl_3$, 300 K): δ [ppm] = −133.62.

HRMS (ESI) *m/z* [M+H]$^+$ calculated for [$C_{12}H_{16}FO_2$]$^+$: 211.1129; found: 211.1117.

IR (ATR): $\tilde{\nu}$ [cm^{-1}] = 3090, 3066, 3034 (m, C_{ar}–H), 2983, 2942 (m, C_{aliph}–H), 1739 (s, C=O), 1456 (s, C_{ar}=C_{ar}), 1167 (s, C–O), 1133 (s, C–F).

Benzyl 5-fluoro-5-methylhexanoate (12c)

According to GP C, a solution of $Ir[dF(CF_3)ppy]_2(dtbbpy)PF_6$ **I** (8.98 mg, 8.00 µmol, 2 mol%), 5-methylhexanoic acid (**1c**) (57.2 µL, 400 µmol, 1.00 equiv.) and Selectfluor® (709 mg, 2.00 mmol, 5.00 equiv.) in a mixture of acetonitrile/water (4 mL, 1:1 v/v) was degassed in an LED tube by sparging argon for 10 minutes, then irradiated with a 10 W blue LED (λ = 405 nm) for 6 h. After the irradiation, the reaction mixture was diluted with Et_2O (10 mL). The organic layer was separated, and the aqueous layer was extracted with Et_2O (3 × 15 mL). The combined organic phase was washed with brine, dried over Na_2SO_4, and concentrated under reduced pressure at 30 ºC. Subsequently, the crude reaction mixture was dissolved in dimethyl formamide (DMF) (5 mL, 0.08 M) followed by the addition of K_2CO_3 (82.9 mg, 600 µmol, 1.50 equiv.) and benzyl bromide (BnBr) (71.3 µL, 600 µmol, 1.50 equiv.). The resulting mixture was stirred at 30 °C for 24 h. The suspension was diluted with Et_2O (15 mL) and washed with H_2O (3 × 10 mL). The organic layer washed with brine, dried over Na_2SO_4 and concentrated under reduced pressure. The obtained crude product was then purified by flash column chromatography FCC (SiO_2, pentane → pentane/EtOAc = 98:2) to yield benzyl 5-fluoro-5-methylhexanoate (**12c**) as a colorless oil (67.7 mg, 284 µmol, 71%).

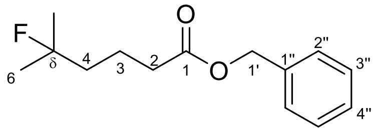

12c
$C_{14}H_{19}FO_2$
MW: 238.30 gmol^{-1}

TLC (pentane/EtOAc = 98:2): R_f = 0.24 [UV, $KMnO_4$].

^{1}H-NMR (400 MHz, $CDCl_3$, 300 K): δ [ppm] = 7.42 – 7.33 (m, 5H, H2", H3", H4"), 5.13 (s, 2H, H1'), 2.39 (t, $^3J_{H\text{-}H}$ = 7.3 Hz, 2H, H2), 1.81 – 1.69 (m, 2H, H3), 1.69 – 1.56 (m, 2H, H4), 1.34 (d, $^3J_{H\text{-}F}$ = 21.4 Hz, 6H, H6).

^{13}C-NMR (101 MHz, $CDCl_3$, 300 K): δ [ppm] = 173.4 (C1), 136.2 (C1"), 128.7 (C2", C4"), 128.4 (C3"), 95.5 (d, $^1J_{C\text{-}F}$ = 165.2 Hz, C5), 66.3 (C1'), 40.8 (d, $^2J_{C\text{-}F}$ = 23.0 Hz, C4), 34.5 (C2), 26.7 (d, $^2J_{C\text{-}F}$ = 25.1 Hz, C6), 19.6 (C3).

^{19}F NMR (376 MHz, $CDCl_3$, 300 K): δ [ppm] = −138.31.

HRMS (ESI) *m/z* $[M+H]^+$ calculated for $[C_{14}H_{20}FO_2]^+$: 239.1442; found: 239.1428.

IR (ATR): $\tilde{\nu}$ [cm^{-1}] = 3066, 3034 (m, C_{ar}–H), 2978, 2935 (m, C_{aliph}–H), 1736 (s, C=O), 1456 (s, C_{ar}=C_{ar}), 1165 (s, C–O), 1050 (s, C–F).

Benzyl 6-fluoro-6-methylheptanoate (12d)

According to GP C, a solution of Ir[dF(CF_3)ppy]$_2$(dtbbpy)PF_6 **I** (8.98 mg, 8.00 µmol, 2 mol%), 6-methylheptanoic acid (**1d**) (64.1 µL, 400 µmol, 1.00 equiv.) and Selectfluor® (709 mg, 2.00 mmol, 5.00 equiv.) in a mixture of acetonitrile/water (4 mL, 1:1 v/v) was degassed in an LED tube by sparging argon for 10 minutes, then irradiated with a 10 W blue LED (λ = 405 nm) for 6 h. After the irradiation, the reaction mixture was diluted with Et_2O (10 mL). The organic layer was separated, and the aqueous layer was extracted with Et_2O (3 × 15 mL). The combined organic phase was washed with brine, dried over Na_2SO_4, and concentrated under reduced pressure at 30 ºC. Subsequently, the crude reaction mixture was dissolved in dimethyl formamide (DMF) (5 mL, 0.08 M) followed by the addition of K_2CO_3 (82.9 mg, 600 µmol, 1.50 equiv.) and benzyl bromide (BnBr) (71.3 µL, 600 µmol, 1.50 equiv.). The resulting mixture was stirred at 30 °C for 24 h. The suspension was diluted with Et_2O (15 mL) and washed with H_2O (3 × 10 mL). The organic layer washed with brine, dried over Na_2SO_4 and concentrated under reduced pressure. The obtained crude product was then purified by flash column chromatography FCC (SiO_2, pentane → pentane/EtOAc = 98:2) to yield benzyl 6-fluoro-6-methylheptanoate (**12d**) as a colorless oil (57.0 mg, 226 µmol, 56%).

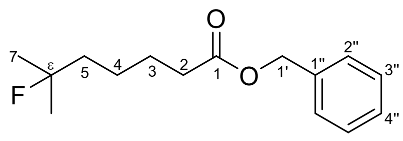

12d
$C_{15}H_{21}FO_2$
MW: 252.33 gmol^{-1}

TLC (pentane/EtOAc = 98:2): R_f = 0.26 [UV, $KMnO_4$].

^{1}H-NMR (400 MHz, $CDCl_3$, 300 K): δ [ppm] = 7.38 – 7.30 (m, 5H, H2", H3", H4"), 5.12 (s, 2H, H1'), 2.38 (t, $^3J_{H\text{-}H}$ = 7.4 Hz, 2H, H2), 1.73 – 1.53 (m, 4H, H3, H4), 1.48 – 1.39 (m, 2H, H5), 1.31 (d, $^3J_{H\text{-}F}$ = 21.5 Hz, 6H, H7).

^{13}C-NMR (101 MHz, $CDCl_3$, 300 K): δ [ppm] = 173.5 (C1), 136.2 (C1"), 128.7 (C3"), 128.3 (C2", C4"), 95.6 (d, $^1J_{C\text{-}F}$ = 164.7 Hz, C6), 66.3 (C1'), 41.2 (d, $^2J_{C\text{-}F}$ = 23.0 Hz, C5), 34.4 (C2), 26.8 (d, $^2J_{C\text{-}F}$ = 24.7 Hz, C7), 25.4 (C3), 23.7 (C4).

^{19}F NMR (376 MHz, $CDCl_3$, 300 K): δ [ppm] = −137.72.

HRMS (ESI) *m/z* [M+H]$^+$ calculated for [$C_{15}H_{22}FO_2$]$^+$: 253.1598; found: 253.1587.

IR (ATR): $\tilde{\nu}$ [cm^{-1}] = 3066, 3035 (m, C_{ar}–H), 2969, 2938, 2866 (m, C_{aliph}–H), 1736 (s, C=O), 1456 (s, C_{ar}=C_{ar}), 1164 (s, C–O), 1139 (s, C–F).

Benzyl 4-fluoro-2,4-dimethylpentanoate (12e)

According to GP C, a solution of Ir[dF(CF_3)ppy]$_2$(dtbbpy)PF_6 **I** (8.98 mg, 8.00 µmol, 2 mol%), 2,4-dimethylpentanoic acid (**1e**) (57.9 µL, 400 µmol, 1.00 equiv.) and Selectfluor® (709 mg, 2.00 mmol, 5.00 equiv.) in a mixture of acetonitrile/water (4 mL, 1:1 v/v) was degassed in an LED tube by sparging argon for 10 minutes, then irradiated with a 10 W blue LED (λ = 405 nm) for 6 h. After the irradiation, the reaction mixture was diluted with Et_2O (10 mL). The organic layer was separated, and the aqueous layer was extracted with Et_2O (3 × 15 mL). The combined organic phase was washed with brine, dried over Na_2SO_4, and concentrated under reduced pressure at 30 ºC. Subsequently, the crude reaction mixture was dissolved in dimethyl formamide (DMF) (5 mL, 0.08 M) followed by the addition of K_2CO_3 (82.9 mg, 600 µmol, 1.50 equiv.) and benzyl bromide (BnBr) (71.3 µL, 600 µmol, 1.50 equiv.). The resulting mixture was stirred at 30 °C for 24 h. The suspension was diluted with Et_2O (15 mL) and washed with H_2O (3 × 10 mL). The organic layer washed with brine, dried over Na_2SO_4 and concentrated under reduced pressure. The obtained crude product was then purified by flash column chromatography FCC (SiO_2, pentane → pentane/EtOAc = 98:2) to yield benzyl 4-fluoro-2,4-dimethylpentanoate (**12e**) as a colorless oil (60.0 mg, 252 µmol, 63%).

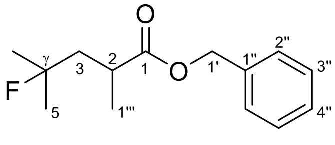

12e

$C_{14}H_{19}FO_2$

MW: 238.30 gmol^{-1}

TLC (pentane/EtOAc = 98:2): R_f = 0.24 [UV, $KMnO_4$].

^{1}H-NMR (400 MHz, $CDCl_3$, 300 K): δ [ppm] = 7.38 – 7.30 (m, 5H, H2'', H3'', H4''), 5.13 (s, 2H, H1'), 2.54 – 2.43 (m, 1H, H2), 1.83 – 1.68 (m, 1H, H3), 1.57 – 1.54 (m, 1H, H3), 1.31 (d, $^3J_{H\text{-}F}$ = 21.4 Hz, 6H, H5), 1.20 (d, $^3J_{H\text{-}H}$ = 7.1 Hz, 3H, H1''').

^{13}C-NMR (101 MHz, $CDCl_3$, 300 K): δ [ppm] = 176.4 (C1), 136.3 (C1''), 128.7 (C3''), 128.3 (C2''), 128.3 (C4''), 95.4 (d, $^1J_{C\text{-}F}$ = 165.3 Hz, C4), 66.2 (C1'), 38.8 (d, $^2J_{C\text{-}F}$ = 23.1 Hz, C3), 28.1 (d, $^3J_{C\text{-}F}$ = 5.2 Hz, C2), 26.9 (d, $^2J_{C\text{-}F}$ = 24.8 Hz, C5), 26.5 (d, $^2J_{C\text{-}F}$ = 24.8 Hz, C5), 17.2 (C1''').

^{19}F NMR (376 MHz, $CDCl_3$, 300 K): δ [ppm] = −138.56.

HRMS (ESI) *m/z* [M+H]$^+$ calculated for $[C_{14}H_{20}FO_2]^+$: 239.1442; found: 239.1431.

IR (ATR): $\tilde{\nu}$ [cm^{-1}] = 3066, 3034 (m, C_{ar}–H), 2978, 2937 (m, C_{aliph}–H), 1731 (s, C=O), 1456 (s, C_{ar}=C_{ar}), 1169 (s, C–O), 1139 (s, C–F).

Benzyl 3-fluoro-2,3-dimethylbutanoate (12f)

According to GP C, a solution of Ir[dF(CF_3)ppy]$_2$(dtbbpy)PF_6 **I** (8.98 mg, 8.00 µmol, 2 mol%), 2,3-dimethylbutanoic acid (**1f**) (50.1 µL, 400 µmol, 1.00 equiv.) and Selectfluor® (709 mg, 2.00 mmol, 5.00 equiv.) in a mixture of acetonitrile/water (4 mL, 1:1 v/v) was degassed in an LED tube by sparging argon for 10 minutes, then irradiated with a 10 W blue LED (λ = 405 nm) for 6 h. After the irradiation, the reaction mixture was diluted with Et_2O (10 mL). The organic layer was separated, and the aqueous layer was extracted with Et_2O (3 × 15 mL). The combined organic phase was washed with brine, dried over Na_2SO_4, and concentrated under reduced pressure at 30 ºC. Subsequently, the crude reaction mixture was dissolved in dimethyl formamide (DMF) (5 mL, 0.08 M) followed by the addition of K_2CO_3 (82.9 mg, 600 µmol, 1.50 equiv.) and benzyl bromide (BnBr) (71.3 µL, 600 µmol, 1.50 equiv.). The resulting mixture was stirred at 30 °C for 24 h. The suspension was diluted with Et_2O (15 mL) and washed with H_2O (3 × 10 mL). The organic layer washed with brine, dried over Na_2SO_4 and concentrated under reduced pressure. The obtained crude product was then purified by flash column chromatography FCC (SiO_2, pentane → pentane/EtOAc = 98:2) to yield benzyl 3-fluoro-2,3-dimethylbutanoate (**12f**) as a colorless oil (63.0 mg, 281 µmol, 70%).

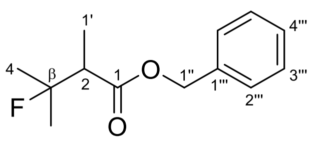

12f

$C_{13}H_{17}FO_2$

MW: 224.28 gmol^{-1}

TLC (pentane/EtOAc = 98:2): R_f = 0.27 [UV, $KMnO_4$].

^{1}H-NMR (500 MHz, $CDCl_3$, 300 K): δ [ppm] = 7.41 – 7.28 (m, 5H, H2", H3", H4"), 5.14 (dd, $^2J_{H\text{-}H}$ = 15.2, 12.4 Hz, 2H, H1"), 2.84 (dq, $^3J_{H\text{-}F}$ = 10.7 Hz, $^3J_{H\text{-}H}$ = 7.1 Hz, 1H, H2), 1.40 (d, $^3J_{H\text{-}F}$ = 21.8 Hz, 3H, H4), 1.39 (d, $^3J_{H\text{-}F}$ = 22.1 Hz, 3H, H4), 1.23 (d, $^3J_{H\text{-}H}$ = 7.1 Hz, 3H, H1').

^{13}C-NMR (101 MHz, $CDCl_3$, 300 K): δ [ppm] = 173.4 (d, $^3J_{C\text{-}F}$ = 9.4 Hz, C1), 136.0 (C1"), 128.7 (C3"), 128.4 (C4"), 128.3 (C2"), 95.6 (d, $^1J_{C\text{-}F}$ = 171.5 Hz, C3), 66.5 (C1"), 49.2 (d, $^2J_{C\text{-}F}$ = 24.6 Hz, C2), 25.2 (d, $^2J_{C\text{-}F}$ = 24.4 Hz, C4), 24.4 (d, $^2J_{C\text{-}F}$ = 24.2 Hz, C4), 12.7 (d, $^3J_{C\text{-}F}$ = 5.6 Hz, C1').

^{19}F NMR (376 MHz, $CDCl_3$, 300 K): δ [ppm] = −137.57.

HRMS (ESI) *m/z* [M+H]$^+$ calculated for $[C_{13}H_{18}FO_2]^+$: 225.1285; found: 225.1285.

IR (ATR): $\tilde{\nu}$ [cm^{-1}] = 3066, 3034 (m, C_{ar}–H), 2981, 2942 (m, C_{aliph}–H), 1736 (s, C=O), 1456 (s, C_{ar}=C_{ar}), 1386 (m, C_{aliph}–H), 1165 (s, C–O), 1144 (s, C–F).

Benzyl 5-fluoro-2,5-dimethylhexanoate (12g)

According to GP C, a solution of Ir[dF(CF_3)ppy]$_2$(dtbbpy)PF_6 **I** (8.98 mg, 8.00 µmol, 2 mol%), 2,5-dimethylhexanoic acid (**1g**) (57.7 mg, 400 µmol, 1.00 equiv.) and Selectfluor® (709 mg, 2.00 mmol, 5.00 equiv.) in a mixture of acetonitrile/water (4 mL, 1:1 v/v) was degassed in an LED tube by sparging argon for 10 minutes, then irradiated with a 10 W blue LED (λ = 405 nm) for 6 h. After the irradiation, the reaction mixture was diluted with Et_2O (10 mL). The organic layer was separated, and the aqueous layer was extracted with Et_2O (3 × 15 mL). The combined organic phase was washed with brine, dried over Na_2SO_4, and concentrated under reduced pressure at 30 ºC. Subsequently, the crude reaction mixture was dissolved in dimethyl formamide (DMF) (5 mL, 0.08 M) followed by the addition of K_2CO_3 (82.9 mg, 600 µmol, 1.50 equiv.) and benzyl bromide (BnBr) (71.3 µL, 600 µmol, 1.50 equiv.). The resulting mixture was stirred at 30 °C for 24 h. The suspension was diluted with Et_2O (15 mL) and washed with H_2O (3 × 10 mL). The organic layer washed with brine, dried over Na_2SO_4 and concentrated under reduced pressure. The obtained crude product was then purified by flash column chromatography FCC (SiO_2, pentane → pentane/EtOAc = 98:2) to yield benzyl 5-fluoro-2,5-dimethylhexanoate (**12g**) as a colorless oil (42.0 mg, 167 µmol, 42%).

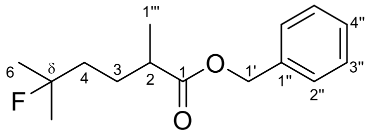

12g
$C_{15}H_{21}FO_2$
MW: 252.33 gmol^{-1}

TLC (pentane/EtOAc = 98:2): R_f = 0.26 [UV, $KMnO_4$].

^{1}H-NMR (400 MHz, $CDCl_3$, 300 K): δ [ppm] = 7.41 – 7.29 (m, 5H, H2”, H3”, H 4”), 5.11 (s, 2H, H1’), 2.81 – 2.68 (m, 1H, H2), 2.21 (ddd, $^3J_{H\text{-}F}$ = 22.6 Hz, $^2J_{H\text{-}H}$ = 14.7 Hz, $^3J_{H\text{-}H}$ = 8.9 Hz, 1H, H4), 1.64 (ddd, $^3J_{H\text{-}F}$ = 20.5 Hz, $^2J_{H\text{-}H}$ = 14.7 Hz, $^2J_{H\text{-}H}$ = 4.2 Hz, 1H, H4), 1.59 (m, 1H, H3) 1.36 (d, $^3J_{H\text{-}F}$ = 19.6 Hz, 3H, H6), 1.30 (d, $^3J_{H\text{-}F}$ = 19.7 Hz, 3H, H6), 1.23 (d, $^3J_{H\text{-}H}$ = 6.6 Hz, 3H, H1’”), 1.19 (m, 1H, H3). H3 signals overlap with the H1” and H4 signals.

^{13}C-NMR (101 MHz, $CDCl_3$, 300 K): δ [ppm] = 176.8 (C1), 136.2 (C1”), 128.7 (C3”), 128.3 (C2”), 128.3 (C4”), 95.1 (d, $^1J_{C\text{-}F}$ = 166.1 Hz, C5), 66.5 (C1’”), 44.9 (d, $^2J_{C\text{-}F}$ = 22.2 Hz, C4), 35.7 (d, $^3J_{C\text{-}F}$ = 2.9 Hz, C3), 29.9 (C2), 27.3 (d, $^2J_{C\text{-}F}$ = 24.7 Hz, C6), 26.7 (d, $^2J_{C\text{-}F}$ = 24.7 Hz, C6), 19.3 (C1’”).

^{19}F NMR (376 MHz, $CDCl_3$, 300 K): δ [ppm] = −138.80.

HRMS (ESI) *m/z* $[M+H]^+$ calculated for $[C_{15}H_{22}FO_2]^+$: 253.1598; found: 253.1586.

IR (ATR): $\tilde{\nu}$ [cm^{-1}] = 3090, 3066, 3034 (m, C_{ar}–H), 2977, 2939, 2879 (m, C_{aliph}–H), 1735 (s, C=O), 1456 (s, C_{ar}=C_{ar}), 1161 (s, C–O), 1142 (s, C–F).

Benzyl-2-chloro-4-fluoro-4-methylpentanoate (12h)

According to GP C, a solution of Ir[dF(CF_3)ppy]$_2$(dtbbpy)PF_6 **I** (8.98 mg, 8.00 µmol, 2 mol%), 2-chloro-4-methylpentanoic acid (**1h**) (60.2 µL, 400 µmol, 1.00 equiv.) and Selectfluor® (709 mg, 2.00 mmol, 5.00 equiv.) in a mixture of acetonitrile/water (4 mL, 1:1 v/v) was degassed in an LED tube by sparging argon for 10 minutes, then irradiated with a 10 W blue LED (λ = 405 nm) for 6 h. After the irradiation, the reaction mixture was diluted with Et_2O (10 mL). The organic layer was separated, and the aqueous layer was extracted with Et_2O (3 × 15 mL). The combined organic phase was washed with brine, dried over Na_2SO_4, and concentrated under reduced pressure at 30 ºC. Subsequently, the crude reaction mixture was dissolved in dimethyl formamide (DMF) (5 mL, 0.08 M) followed by the addition of K_2CO_3 (82.9 mg, 600 µmol, 1.50 equiv.) and benzyl bromide (BnBr) (71.3 µL, 600 µmol, 1.50 equiv.). The resulting mixture was stirred at 30 °C for 24 h. The suspension was diluted with Et_2O (15 mL) and washed with H_2O (3 × 10 mL). The organic layer washed with brine, dried over Na_2SO_4 and concentrated under reduced pressure. The obtained crude product was then purified by flash column chromatography FCC (SiO_2, pentane → pentane/EtOAc = 98:2) to yield benzyl-2-chloro-4-fluoro-4-methylpentanoate (**12h**) as a colorless oil (65.0 mg, 251 µmol, 63%).

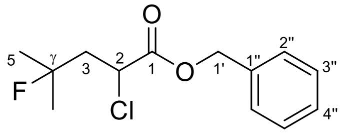

12h
$C_{13}H_{16}ClFO_2$
MW: 258.72 gmol^{-1}

TLC (pentane/EtOAc = 98:2): R_f = 0.22 [UV, $KMnO_4$].

^{1}H-NMR (400 MHz, $CDCl_3$, 300 K): δ [ppm] = 7.40 – 7.31 (m, 5H, H2”, H3”, H4”), 5.21 (s, 2H, H1’), 4.48 (dd, $^3J_{H\text{-}H}$ = 8.2 Hz, 5.3 Hz, 1H, H2), 2.62 (ddd, $^3J_{H\text{-}F}$ = 16.5 Hz, $^2J_{H\text{-}H}$ = 14.9 Hz, $^3J_{H\text{-}H}$ = 8.2 Hz, 1H, H3), 2.19 (ddd, $^3J_{H\text{-}F}$ = 23.8 Hz, $^2J_{H\text{-}H}$ = 14.9 Hz, $^3J_{H\text{-}H}$ = 5.3 Hz, 1H, H3), 1.43 (d, $^3J_{H\text{-}F}$ = 21.5 Hz, 3H, H5), 1.35 (d, $^3J_{H\text{-}F}$ = 21.5 Hz, 3H, H5).

^{13}C-NMR (101 MHz, $CDCl_3$, 300 K): δ [ppm] = 169.7 (C1), 135.2 (C1”), 128.8 (C3”), 128.7 (C4”), 128.5 (C2”), 94.1 (d, $^1J_{C\text{-}F}$ = 168.2 Hz, C4), 68.0 (C1’), 52.3 (d, $^3J_{C\text{-}F}$ = 4.1 Hz, C2), 46.2 (d, $^2J_{C\text{-}F}$ = 22.5 Hz, C3), 27.6 (d, $^2J_{C\text{-}F}$ = 24.3 Hz, C5), 26.6 (d, $^2J_{C\text{-}F}$ = 24.7 Hz, C5).

^{19}F NMR (376 MHz, $CDCl_3$, 300 K): δ [ppm] = −139.26.

HRMS (ESI) *m/z* $[M]^+$ calculated for $[C_{13}H_{16}ClFO_2]^+$: 258.0817; found: 258.0832.

IR (ATR): $\tilde{\nu}$ [cm^{-1}] = 3090, 3066, 3034 (m, C_{ar}–H), 2983, 2939 (m, C_{aliph}–H), 1746 (s, C=O), 1456 (s, C_{ar}=C_{ar}), 1161 (s, C–O), 1140 (s, C–F).

Benzyl-5-fluoro-5-methyl-3-(2,2,2-trifluoroacetamido)hexanoate (12i)

According to GP C, a solution of Ir[dF(CF_3)ppy]$_2$(dtbbpy)PF_6 **I** (8.98 mg, 8.00 µmol, 2 mol%), (*S*)-5-methyl-3-(2,2,2-trifluoroacetamido)hexanoic acid (**1i**) (96.5 mg, 400 µmol, 1.00 equiv.) and Selectfluor® (709 mg, 2.00 mmol, 5.00 equiv.) in a mixture of acetonitrile/water (4 mL, 1:1 v/v) was degassed in an LED tube by sparging argon for 10 minutes, then irradiated with a 10 W blue LED (λ = 405 nm) for 6 h. After the irradiation, the reaction mixture was diluted with Et_2O (10 mL). The organic layer was separated, and the aqueous layer was extracted with Et_2O (3 × 15 mL). The combined organic phase was washed with brine, dried over Na_2SO_4, and concentrated under reduced pressure at 30 ºC. Subsequently, the crude reaction mixture was dissolved in dimethyl formamide (DMF) (5 mL, 0.08 M) followed by the addition of K_2CO_3 (82.9 mg, 600 µmol, 1.50 equiv.) and benzyl bromide (BnBr) (71.3 µL, 600 µmol, 1.50 equiv.). The resulting mixture was stirred at 30 °C for 24 h. The suspension was diluted with Et_2O (15 mL) and washed with H_2O (3 × 10 mL). The organic layer washed with brine, dried over Na_2SO_4 and concentrated under reduced pressure. The obtained crude product was then purified by flash column chromatography FCC (SiO_2, pentane → pentane/EtOAc = 98:2) to yield benzyl (*S*)-5-fluoro-5-methyl-3-(2,2,2-trifluoroacetamido)hexanoate (**12i**) as a colorless oil (35.0 mg, 100 µmol, 25%).

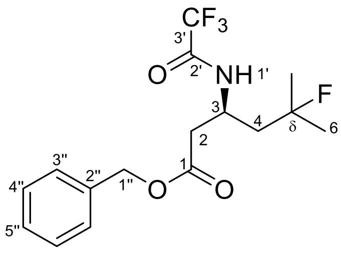

12i

$C_{16}H_{19}F_4NO_3$

MW: 349.33 gmol^{-1}

TLC (pentane/EtOAc = 98:2): R_f = 0.21 [UV, $KMnO_4$].

^{1}H-NMR (400 MHz, $CDCl_3$, 300 K): δ [ppm] = 7.40 – 7.30 (m, 5H, H3”, H4”, H5”), 7.14 – 7.12 (m, 1H, N–H), 5.16 (dd, $^2J_{H\text{-}H}$ = 17.9, 12.3 Hz, 2H, H1”), 4.46 – 4.38 (m, 1H, H3), 2.86 – 2.64 (m, 2H, H2), 2.06 – 1.82 (m, 2H, H4), 1.42 (d, $^3J_{H\text{-}F}$ = 21.4 Hz, 3H, H6), 1.40 (d, $^3J_{H\text{-}F}$ = 21.4 Hz, 3H, H6).

^{13}C-NMR (101 MHz, $CDCl_3$, 300 K): δ [ppm] = 171.4 (C1), 156.6 (q, $^2J_{C\text{-}F3}$ = 37.1 Hz, C2’), 135.3 (C2”), 128.8 (C4”), 128.7 (C5”), 128.5 (C3”), 115.8 (q, $^1J_{C\text{-}F3}$ = 287.6 Hz, C3’), 95.1 (d, $^1J_{C\text{-}F}$ = 165.1 Hz, C5), 67.0 (C1”), 43.9 (C2), 43.6 (d, $^2J_{C\text{-}F}$ = 21.1 Hz, C4), 38.1 (d, $^3J_{C\text{-}F}$ = 2.5 Hz, C3), 27.0 (d, $^2J_{C\text{-}F}$ = 24.6 Hz, C6), 26.8 (d, $^2J_{C\text{-}F}$ = 24.6 Hz, C6).

^{19}F NMR (376 MHz, $CDCl_3$, 300 K): δ [ppm] = −76.31 (CF_3), −138.23 (F5).

HRMS (ESI) *m/z* $[M+H]^+$ calculated for $[C_{16}H_{20}F_4NO_3]^+$: 350.1374; found: 350.1371.

IR (ATR): $\tilde{\nu}$ [cm^{-1}] = 3308 (s, N–H), 3066 (m, C_{ar}–H), 2983, 2940 (m, C_{aliph}–H), 1732 (s, C_{ester}=O), 1707 (s, C_{amide}=O), 1164 (m, C-F_3), 1023 (s, C-F).
Specific Rotation: $[\alpha]_D^{25}$: −33 (c = 2.0, $CHCl_3$).

Benzyl 4-fluoropentanoate (12j)

According to GP C, a solution of Ir[dF(CF_3)ppy]$_2$(dtbbpy)PF_6 **I** (8.98 mg, 8.00 µmol, 2 mol%), valeric acid (**1j**) (43.5 µL, 400 µmol, 1.00 equiv.) and Selectfluor® (709 mg, 2.00 mmol, 5.00 equiv.) in a mixture of acetonitrile/water (4 mL, 1:1 v/v) was degassed in an LED tube by sparging argon for 10 minutes, then irradiated with a 10 W blue LED (λ = 405 nm) for 6 h. After the irradiation, the reaction mixture was diluted with Et_2O (10 mL). The organic layer was separated, and the aqueous layer was extracted with Et_2O (3 × 15 mL). The combined organic phase was washed with brine, dried over Na_2SO_4, and concentrated under reduced pressure at 30 ºC. Subsequently, the crude reaction mixture was dissolved in dimethyl formamide (DMF) (5 mL, 0.08 M) followed by the addition of K_2CO_3 (82.9 mg, 600 µmol, 1.50 equiv.) and benzyl bromide (BnBr) (71.3 µL, 600 µmol, 1.50 equiv.). The resulting mixture was stirred at 30 °C for 24 h. The suspension was diluted with Et_2O (15 mL) and washed with H_2O (3 × 10 mL). The organic layer washed with brine, dried over Na_2SO_4 and concentrated under reduced pressure. The obtained crude product was then purified by flash column chromatography FCC (SiO_2, pentane → pentane/EtOAc = 98:2) to yield benzyl 4-fluoropentanoate (**12j**) as a colorless oil (29.0 mg, 138 µmol, 34%).

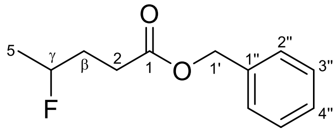

12j
$C_{12}H_{15}FO_2$
MW: 210.25 gmol^{-1}

TLC (pentane/EtOAc = 98:2): R_f = 0.25 [UV, $KMnO_4$].
^{1}H-NMR (400 MHz, $CDCl_3$, 300 K): δ [ppm] = 7.39 – 7.32 (m, 5H, H2”, H3”, H4”), 5.13 (s, 2H, H1’), 4.69 (m, 1H, H4), 2.62 – 2.42 (m, 2H, H2), 2.03 – 1.84 (m, 2H, H3), 1.34 (dd, $^3J_{H-F}$ = 23.8 Hz, $^3J_{H-H}$ = 6.2 Hz, 3H, H5).
^{13}C-NMR (101 MHz, $CDCl_3$, 300 K): δ [ppm] = 173.1 (C1), 136.1 (C1”), 128.7 (C3”), 128.4 (C4”), 128.3 (C2”), 90.0 (d, $^1J_{C-F}$ = 165.6 Hz, C4), 66.5 (C1’), 32.1 (d, $^2J_{C-F}$ = 21.1 Hz, C3), 30.0 (d, $^3J_{C-F}$ = 4.4 Hz, C2), 21.0 (d, $^2J_{C-F}$ = 22.5 Hz, C5).
^{19}F NMR (376 MHz, $CDCl_3$, 300 K): δ [ppm] = −175.48 (major, γ), −180.75 (minor, β).
HRMS (ESI) m/z [M+H]$^+$ calculated for $[C_{12}H_{16}FO_2]^+$: 211.1129; found: 211.1129.

IR (ATR): $\tilde{\nu}$ [cm^{-1}] = 3090, 3066, 3034 (m, C_{ar}–H), 2982, 2939 (m, C_{aliph}–H), 1736 (s, C=O), 1456 (s, C_{ar}=C_{ar}), 1164 (s, C–O), 1112 (s, C–F).

Methyl 2-(2-fluoropropan-2-yl)benzoate (13k)

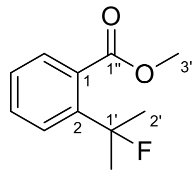

13k

$C_{11}H_{13}FO_2$

MW: 196.22 gmol^{-1}

According to GP D, a solution of Ir[dF(CF_3)ppy]$_2$(dtbbpy)PF_6 **I** (8.98 mg, 8.00 µmol, 2 mol%), 2-isopropylbenzoic acid (**1k**) (65.7 mg, 400 µmol, 1.00 equiv.) and Selectfluor® (709 mg, 2.00 mmol, 5.00 equiv.) in a mixture of acetonitrile/water (4 mL, 1:1 v/v) was degassed in an LED tube by sparging argon for 10 minutes, then irradiated with a 10 W blue LED (λ = 405 nm) for 6 h. After the irradiation, the reaction mixture was diluted with Et_2O (10 mL). The organic layer was separated, and the aqueous layer was extracted with Et_2O (3 × 15 mL). The combined organic phase was washed with brine, dried over Na_2SO_4, and concentrated under reduced pressure at 30 ºC. Subsequently, (trimethylsilyl)diazomethane (300 µL, 600 µmol, 1.50 equiv.) was added dropwise to a solution of the crude reaction mixture in DCM/MeOH (5 mL, 4:1, 0.08 M) at 0 °C. After the reaction mixture was stirred for 4h at 30 °C, unreacted excess of (trimethylsilyl)diazomethane was quenched with AcOH. The reaction mixture was concentrated under reduced pressure, and the obtained crude product was then purified by flash column chromatography FCC (SiO_2, pentane/EtOAc = 95:5 → pentane/EtOAc = 9:1) to yield methyl 2-(2-fluoropropan-2-yl)benzoate (**13k**) as a colorless oil (12.6 mg, 64.2 µmol, 16%).

TLC (pentane/EtOAc = 9:1): R_f = 0.33 [UV, $KMnO_4$].

^{1}H-NMR (400 MHz, $CDCl_3$, 300 K): δ [ppm] = 7.45 – 7.39 (m, 2H, H4, H6), 7.36 – 7.28 (m, 2H, H3, H5), 3.89 (s, 3H, H3''), 1.78 (d, $^3J_{H-F}$ = 22.6 Hz, 6H, H2').

^{13}C-NMR (101 MHz, $CDCl_3$, 300 K): δ [ppm] = 171.0 (C1''), 143.6 (d, $^2J_{C-F}$ = 21.2 Hz, C2), 130.7 (d, $^4J_{C-F}$ = 5.6 Hz, C4), 130.1 (d, $^4J_{C-F}$ = 2.2 Hz, C6), 128.4 (C1), 127.3 (C5), 125.2 (d, $^3J_{C-F}$ = 10.0 Hz, C3), 96.8 (d, $^1J_{C-F}$ = 169.5 Hz, C1'), 52.5 (C3''), 29.2 (d, $^2J_{C-F}$ = 25.6 Hz, C2'').

^{19}F NMR (376 MHz, $CDCl_3$, 300 K): δ [ppm] = −132.33.

HRMS (ESI) *m/z* [M+H]$^+$ calculated for [$C_{11}H_{14}FO_2$]$^+$: 197.0972, found: 197.0972.

IR (ATR): $\tilde{\nu}$ [cm^{-1}] = 3069, 3033 (m, C_{ar}–H), 2988, 2953 (m, C_{aliph}–H) , 1729 (s, C=O), 1599, 1577 (m, C_{ar}=C_{ar}), 1293, 1256 (s, C–O), 1139 (s, C–F).

3,3-Dimethylisobenzofuran-1(3*H*)-one (14)

Along with desired fluorinated ester **13k** the corresponding lactone 3,3-dimethylisobenzofuran-1(3*H*)-one (**14**) was also isolated (15 mg, 92.6 µmol, 23%).

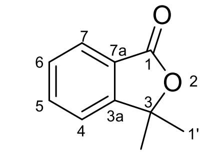

14
$C_{10}H_{10}O_2$
MW: 162.19 gmol^{-1}

TLC (pentane/EtOAc = 9:1): R_f = 0.24 [UV, $KMnO_4$].

^{1}H-NMR (400 MHz, $CDCl_3$, 300 K): δ [ppm] = 7.87 (dt, $^3J_{H\text{-}H}$ = 7.7 Hz, $^4J_{H\text{-}H}$ = 1.0 Hz, 1H, H7), 7.66 (*virt*. td, $^3J_{H\text{-}H}$ = 7.6 Hz, $^4J_{H\text{-}H} \cong {}^4J_{H\text{-}H}$ = 1.2 Hz, 1H, H5), 7.50 (*virt*. td, $^3J_{H\text{-}H}$ = 7.5 Hz, $^4J_{H\text{-}H} \cong {}^4J_{H\text{-}H}$ = 1.0 Hz, 1H, H6), 7.40 (dt, $^3J_{H\text{-}H}$ = 7.7 Hz, $^4J_{H\text{-}H}$ = 1.0 Hz, 1H, H4), 1.66 (s, 6H, H1').

^{13}C-NMR (101 MHz, $CDCl_3$, 300 K): δ [ppm] = 169.9 (C1), 155.1 (C7a), 134.2 (C5), 129.1 (C7), 128.4 (C3a), 125.9 (C6), 120.8 (C4), 85.5 (C3), 27.5 (C1').

The spectroscopic data matches the values reported in the literature.[2]

2-(1-Fluoroethyl)benzoic acid (2l)

According to GP C, a solution of $Ir[dF(CF_3)ppy]_2(dtbbpy)PF_6$ **I** (8.98 mg, 8.00 µmol, 2 mol%), 2-ethylbenzoic acid (**1l**) (60.1 mg, 400 µmol, 1.00 equiv.) and Selectfluor® (709 mg, 2.00 mmol, 5.00 equiv.) in a mixture of acetonitrile/water (4 mL, 1:1 v/v) was degassed in an LED tube by sparging argon for 10 minutes, then irradiated with a 10 W blue LED (λ = 405 nm) for 6 h. After the irradiation, the reaction mixture was diluted with Et_2O (10 mL). The organic layer was separated, and the aqueous layer was extracted with Et_2O (3 × 15 mL). The combined organic phase was washed with brine, dried over Na_2SO_4, and concentrated under reduced pressure at 30 ºC. The obtained crude product was then purified by flash column chromatography FCC (SiO_2, pentane/EtOAc = 8:2, 1% AcOH) to yield 2-(1-fluoroethyl)benzoic acid (**2l**) as a white solid (30.0 mg, 178 µmol, 45%).

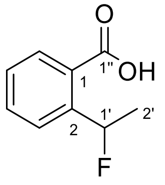

2l
$C_9H_9FO_2$
MW: 168.17 gmol^{-1}

M.p.: 68-69 °C.

TLC (pentane/EtOAc = 8:2, 1% AcOH): R_f = 0.12 [UV, $KMnO_4$].

^{1}H-NMR (400 MHz, $CDCl_3$, 300 K): δ [ppm] = 8.12 (dt, $^3J_{H\text{-}H}$ = 7.9 Hz, $^4J_{H\text{-}H}$ = 1.5 Hz, 1H, H6), 7.77 (dd, $^3J_{H\text{-}H}$ = 7.9 Hz, $^4J_{H\text{-}H}$ = 1.2 Hz, 1H, H5), 7.68 (ddd, $^3J_{H\text{-}H}$ = 8.9, 7.9 Hz, $^4J_{H\text{-}H}$ = 1.5 Hz, 1H, H4), 7.49 – 7.40 (m, 1H, H3), 6.55 (dq, $^2J_{H\text{-}F}$ = 48.7 Hz, $^3J_{H\text{-}H}$ = 6.2 Hz, 1H, H1'), 1.69 (dd, $^3J_{H\text{-}F}$ = 24.3 Hz, $^3J_{H\text{-}H}$ = 6.2 Hz, 3H, H2').

^{13}C-NMR (101 MHz, $CDCl_3$, 300 K): δ [ppm] = 171.3 (C1''), 145.9 (d, $^2J_{C\text{-}F}$ = 19.5 Hz, C2), 134.0 (C1), 131.5 (C4), 127.8 (C3), 125.8 (C5), 125.6 (C6), 88.7 (d, $^1J_{C\text{-}F}$ = 166.5 Hz, C1'), 23.8 (d, $^2J_{C\text{-}F}$ = 25.1 Hz. C2').

^{19}F NMR (376 MHz, $CDCl_3$, 300 K): δ [ppm] = −172.31.

HRMS (ESI) *m/z* $[M-H]^-$ calculated for $[C_9H_8FO_2]^-$: 167.0514; found: 167.0502.

IR (ATR): $\tilde{\nu}$ [cm^{-1}] = 2500–3100 (s, O–H), 2988, 2940 (m, C_{aliph}–H), 1683 (s, C=O), 1603, 1581 (s, C_{ar}=C_{ar}), 1261 (s, C–O), 1082 (s, C–F).

3-(2-Fluoropropan-2-yl)benzoic acid (2m)

According to GP C, a solution of Ir[dF(CF_3)ppy]$_2$(dtbbpy)PF_6 **I** (8.98 mg, 8.00 μmol, 2 mol%), 3-isopropylbenzoic acid (**1m**) (65.7 mg, 400 μmol, 1.00 equiv.) and Selectfluor® (709 mg, 2.00 mmol, 5.00 equiv.) in a mixture of acetonitrile/water (4 mL, 1:1 v/v) was degassed in an LED tube by sparging argon for 10 minutes, then irradiated with a 10 W blue LED (λ = 405 nm) for 6 h. After the irradiation, the reaction mixture was diluted with Et_2O (10 mL). The organic layer was separated, and the aqueous layer was extracted with Et_2O (3 × 15 mL). The combined organic phase was washed with brine, dried over Na_2SO_4, and concentrated under reduced pressure at 30 ºC. The obtained crude product was then purified by flash column chromatography FCC (SiO_2, pentane/EtOAc = 8:2, 1% AcOH) to yield 3-(2-fluoropropan-2-yl)benzoic acid (**2m**) as a white solid (21.5 mg, 118 μmol, 30%).

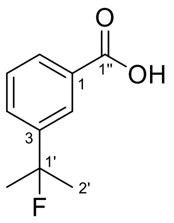

2m
$C_{10}H_{11}FO_2$
MW: 182.19 gmol^{-1}

M.p.: 58-59 °C.

TLC (pentane/EtOAc = 8:2, 1% AcOH): R_f = 0.13 [UV, $KMnO_4$].

^{1}H-NMR (400 MHz, $CDCl_3$, 300 K): δ [ppm] = 8.15 (d, $^4J_{H\text{-}H}$ = 1.8 Hz, 1H, H2), 8.08 (dd, $^3J_{H\text{-}H}$ = 7.7 Hz, $^4J_{H\text{-}H}$ = 1.8 Hz, 1H, H6), 7.75 – 7.67 (m, 1H, H5), 7.51 (*virt.* td, $^3J_{H\text{-}H}$ = 7.7 Hz, $^4J_{H\text{-}H} \cong {}^4J_{H\text{-}H}$ = 2.7 Hz, 1H, H4), 1.75 (d, $^3J_{H\text{-}F}$ = 21.9 Hz, 6H, H2').

^{13}C-NMR (101 MHz, $CDCl_3$, 300 K): δ [ppm] = 172.3 (C1''), 146.7 (d, $^2J_{C\text{-}F}$ = 22.5 Hz, C3), 129.5 (d, $^3J_{C\text{-}F}$ = 9.1 Hz, C4), 129.4 (C1), 129.3 (C5), 128.8 (C6), 125.8 (d, $^3J_{C\text{-}F}$ = 8.7 Hz, C2), 95.5 (d, $^1J_{C\text{-}F}$ = 170.4 Hz, C1'), 29.4 (d, $^2J_{C\text{-}F}$ = 25.6 Hz, C2').

^{19}F NMR (376 MHz, $CDCl_3$, 300 K): δ [ppm] = −137.88.

HRMS (ESI) *m/z* $[M-H]^-$ calculated for $[C_{10}H_{10}FO_2]^-$: 181.0670; found: 181.0661.

IR (ATR): $\tilde{\nu}$ [cm^{-1}] = 2500–3300 (s, O–H), 2984, 2960 (m, C_{aliph}–H), 1690 (s, C=O), 1608, 1588 (s, C_{ar}=C_{ar}), 1306, 1265 (s, C–O / δ O–H), 1139 (s, C–F).

Benzyl 3-(1-fluoroethyl)benzoate (12n)

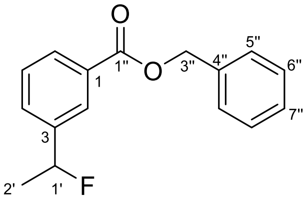

12n

$C_{16}H_{15}FO_2$

MW: 258.29 $gmol^{-1}$

According to GP C, a solution of Ir[dF(CF_3)ppy]$_2$(dtbbpy)PF_6 **I** (8.98 mg, 8.00 µmol, 2 mol%), 3-ethylbenzoic acid (**1n**) (60.1 mg, 400 µmol, 1.00 equiv.) and Selectfluor® (709 mg, 2.00 mmol, 5.00 equiv.) in a mixture of acetonitrile/water (4 mL, 1:1 v/v) was degassed in an LED tube by sparging argon for 10 minutes, then irradiated with a 10 W blue LED (λ = 405 nm) for 6 h. After the irradiation, the reaction mixture was diluted with Et_2O (10 mL). The organic layer was separated, and the aqueous layer was extracted with Et_2O (3 × 15 mL). The combined organic phase was washed with brine, dried over Na_2SO_4, and concentrated under reduced pressure at 30 ºC. Subsequently, the crude reaction mixture was dissolved in dimethyl formamide (DMF) (5 mL, 0.08 M) followed by the addition of K_2CO_3 (82.9 mg, 600 µmol, 1.50 equiv.) and benzyl bromide (BnBr) (71.3 µL, 600 µmol, 1.50 equiv.). The resulting mixture was stirred at 30 °C for 24 h. The suspension was diluted with Et_2O (15 mL) and washed with H_2O (3 × 10 mL). The organic layer washed with brine, dried over Na_2SO_4 and concentrated under reduced pressure. The obtained crude product was then purified by flash column chromatography FCC (SiO_2, pentane → pentane/EtOAc = 98:2) to yield benzyl 3-(1-fluoroethyl)benzoate (**12n**) as a colorless oil (45.0 mg, 174 µmol, 29%).

TLC (pentane/EtOAc = 98:2): R_f = 0.19 [UV, $KMnO_4$].

^{1}H-NMR (400 MHz, $CDCl_3$, 300 K): δ [ppm] = 8.04 (m, 2H, H2, H6), 7.57 (dtd, $^3J_{H\text{-}H}$ = 7.7 Hz, $^4J_{H\text{-}H}$ = 1.8, 0.9 Hz, 1H, H4), 7.49 – 7.43 (m, 3H, H5, H6''), 7.43 – 7.32 (m, 3H, H5'', H7''), 5.67 (dq, $^2J_{H\text{-}F}$ = 47.4 Hz, $^3J_{H\text{-}H}$ = 6.5 Hz, 1H, H1'), 5.38 (s, 2H, H3''), 1.66 (dd, $^3J_{H\text{-}F}$ = 23.8 Hz, $^3J_{H\text{-}H}$ = 6.5 Hz, 3H, H2').

^{13}C-NMR (101 MHz, $CDCl_3$, 300 K): δ [ppm] = 166.3 (C1''), 142.1 (d, $^{2}J_{C\text{-}F}$ = 19.9 Hz, C3), 136.1 (C4''), 130.6 (C6), 129.9 (d, $^{3}J_{C\text{-}F}$ = 6.5 Hz, C4), 129.7 (d, $^{4}J_{C\text{-}F}$ = 1.7 Hz, C1), 128.8 (C5), 128.8 (C6''), 128.5 (C7''), 128.4 (C5''), 126.6 (d, $^{3}J_{C\text{-}F}$ = 7.4 Hz, C2), 90.5 (d, $^{1}J_{C\text{-}F}$ = 168.6 Hz, C1'), 67.0 (C3''), 23.1 (d, $^{2}J_{C\text{-}F}$ = 24.7 Hz, C2').

^{19}F NMR (376 MHz, $CDCl_3$, 300 K): δ [ppm] = −168.64.

HRMS (ESI) *m/z* $[M+H]^+$ calculated for $[C_{16}H_{16}FO_2]^+$: 259.1129; found: 259.1153.

IR (ATR): $\tilde{\nu}$ [cm^{-1}] = 3090, 3066, 3034 (m, C_{ar}–H), 2984, 2938 (m, C_{aliph}–H), 1720 (s, C=O), 1609, 1589 (s, C_{ar}=C_{ar}), 1270 (s, C–O), 1082 (s, C–F).

Benzyl 4-(2-fluoropropan-2-yl)benzoate (12o)

According to GP C, a solution of Ir[dF(CF_3)ppy]$_2$(dtbbpy)PF_6 **I** (8.98 mg, 8.00 µmol, 2 mol%), 4-isopropylbenzoic acid (**1o**) (65.7 mg, 400 µmol, 1.00 equiv.) and Selectfluor® (709 mg, 2.00 mmol, 5.00 equiv.) in a mixture of acetonitrile/water (4 mL, 1:1 v/v) was degassed in an LED tube by sparging argon for 10 minutes, then irradiated with a 10 W blue LED (λ = 405 nm) for 6 h. After the irradiation, the reaction mixture was diluted with Et_2O (10 mL). The organic layer was separated, and the aqueous layer was extracted with Et_2O (3 × 15 mL). The combined organic phase was washed with brine, dried over Na_2SO_4, and concentrated under reduced pressure at 30 ºC. Subsequently, the crude reaction mixture was dissolved in dimethyl formamide (DMF) (5 mL, 0.08 M) followed by the addition of K_2CO_3 (82.9 mg, 600 µmol, 1.50 equiv.) and benzyl bromide (BnBr) (71.3 µL, 600 µmol, 1.50 equiv.). The resulting mixture was stirred at 30 °C for 24 h. The suspension was diluted with Et_2O (15 mL) and washed with H_2O (3 × 10 mL). The organic layer washed with brine, dried over Na_2SO_4 and concentrated under reduced pressure. The obtained crude product was then purified by flash column chromatography FCC (SiO_2, pentane → pentane/EtOAc = 98:2) to yield benzyl 4-(2-fluoropropan-2-yl)benzoate (**12o**) as a colorless oil (20.0 mg, 73 µmol, 18%).

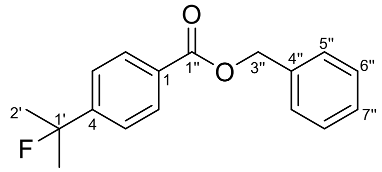

12o
$C_{17}H_{17}FO_2$
MW: 272.32 $gmol^{-1}$

TLC (pentane/EtOAc = 98:2): R_f = 0.19 [UV, $KMnO_4$].

^{1}H-NMR (400 MHz, $CDCl_3$, 300 K): δ [ppm] = 8.07 (d, $^{3}J_{H\text{-}H}$ = 8.1 Hz, 2H, H2), 7.49 – 7.43 (m, 4H, H3, H6''), 7.42 – 7.32 (m, 3H, H5'', H7''), 5.37 (s, 2H, H3''), 1.69 (d, $^{3}J_{H\text{-}F}$ = 21.9 Hz, 6H, H2').

^{13}C-NMR (101 MHz, $CDCl_3$, 300 K): δ [ppm] = 166.3 (C1”), 151.1 (d, $^2J_{C\text{-}F}$ = 21.7 Hz, C4), 136.2 (C4”), 130.0 (d, $^4J_{C\text{-}F}$ = 1.7 Hz, C2), 129.3 (C1), 128.8 (C6”), 128.4 (C7”), 128.3 (C5”), 124.0 (d, $^3J_{C\text{-}F}$ = 9.5 Hz, C3), 95.6 (d, $^1J_{C\text{-}F}$ = 170.8 Hz, C1’), 66.8 (C3”), 29.3 (d, $^2J_{C\text{-}F}$ = 25.6 Hz, C2’).

^{19}F NMR (376 MHz, $CDCl_3$, 300 K): δ [ppm] = −138.88.

HRMS (ESI) *m/z* $[M]^+$ calculated for $[C_{17}H_{17}FO_2]^+$: 272.1207; found: 272.1184.

IR (ATR): $\tilde{\nu}$ [cm^{-1}] = 3066, 3034 (m, C_{ar}–H), 2983, 2938 (m, C_{aliph}–H), 1719 (s, C=O), 1612, 1577 (s, C_{ar}=C_{ar}), 1271 (s, C–O), 1137 (s, C–F).

Benzyl 4-(1-fluoroethyl)benzoate (12p)

According to GP B, a solution of $Ir[dF(CF_3)ppy]_2(dtbbpy)PF_6$ **I** (8.98 mg, 8.00 µmol, 2 mol%), 4-ethylbenzoic acid (**1p**) (60.1 mg, 400 µmol, 1.00 equiv.) and Selectfluor® (709 mg, 2.00 mmol, 5.00 equiv.) in a mixture of acetonitrile/water (4 mL, 1:1 v/v) was degassed in an LED tube by sparging argon for 10 minutes, then irradiated with a 10 W blue LED (λ = 405 nm) for 6 h. After the irradiation, the reaction mixture was diluted with Et_2O (10 mL). The organic layer was separated, and the aqueous layer was extracted with Et_2O (3 × 15 mL). The combined organic phase was washed with brine, dried over Na_2SO_4, and concentrated under reduced pressure at 30 ºC. Subsequently, the crude reaction mixture was dissolved in dimethyl formamide (DMF) (5 mL, 0.08 M) followed by the addition of K_2CO_3 (82.9 mg, 600 µmol, 1.50 equiv.) and benzyl bromide (BnBr) (71.3 µL, 600 µmol, 1.50 equiv.). The resulting mixture was stirred at 30 °C for 24 h. The suspension was diluted with Et_2O (15 mL) and washed with H_2O (3 × 10 mL). The organic layer washed with brine, dried over Na_2SO_4 and concentrated under reduced pressure. The obtained crude product was then purified by flash column chromatography FCC (SiO_2, pentane → pentane/EtOAc = 98:2) to yield benzyl 4-(1-fluoroethyl)benzoate (**12p**) as a colorless oil (33.0 mg, 128 µmol, 32%).

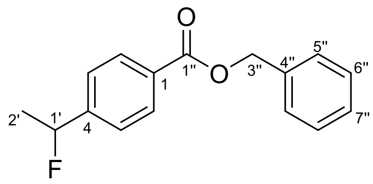

12p
$C_{16}H_{15}FO_2$
MW: 258.29 $gmol^{-1}$

TLC (pentane/EtOAc = 98:2): R_f = 0.18 [UV, $KMnO_4$].

^{1}H-NMR (400 MHz, $CDCl_3$, 300 K): δ [ppm] = 8.13 – 8.05 (m, 2H, H2, H6), 7.49 – 7.30 (m, 7H, H3, H5, H5”, H6”, H7”), 5.68 (dq, $^2J_{H\text{-}F}$ = 47.7 Hz, $^3J_{H\text{-}H}$ = 6.5 Hz, 1H, H1’), 5.38 (s, 2H, H3”), 1.64 (dd, $^2J_{H\text{-}F}$ = 24.0 Hz, $^3J_{H\text{-}H}$ = 6.5 Hz, 3H, H2’).

^{13}C-NMR (101 MHz, $CDCl_3$, 300 K): δ [ppm] = 166.2 (C1''), 146.8 (d, $^{2}J_{C\text{-}F}$ = 19.5 Hz, C4), 136.2 (C4''), 130.1 (C1), 130.0 (d, $^{3}J_{C\text{-}F}$ = 2.2 Hz, C2, C6), 128.8 (C6''), 128.4 (C7''), 128.3 (C5''), 125.1 (d, $^{3}J_{C\text{-}F}$ = 7.4 Hz, C3, C5), 90.5 (d, $^{1}J_{C\text{-}F}$ = 169.5 Hz, C1'), 66.9, 23.2 (d, $^{2}J_{C\text{-}F}$ = 24.7 Hz, C2').
^{19}F NMR (376 MHz, $CDCl_3$, 300 K): δ [ppm] = −171.20.
HRMS (ESI) *m/z* $[M+H]^+$ calculated for $[C_{16}H_{16}FO_2]^+$: 259.1129; found: 259.1154.
IR (ATR): $\tilde{\nu}$ [cm^{-1}] = 3090, 3066, 3034 (m, C_{ar}–H), 2984, 2936 (m, C_{aliph}–H), 1716 (s, C=O), 1613, 1579 (s, C_{ar}=C_{ar}), 1269 (s, C-O), 1082 (s, C–F).

5-Phenyldihydrofuran-2(3*H*)-one (15)

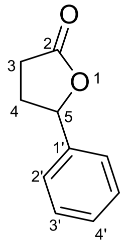

15
$C_{10}H_{10}O_2$
MW: 162,19 $gmol^{-1}$

According to GP B, a solution of $Ir[dF(CF_3)ppy]_2(dtbbpy)PF_6$ **I** (8.98 mg, 8.00 µmol, 2 mol%), 4-phenylbutanoic acid (**1q**) (67.9 µL , 400 µmol, 1.00 equiv.) and Selectfluor® (709 mg, 2.00 mmol, 5.00 equiv.) in a mixture of acetonitrile/water (4 mL, 1:1 v/v) was degassed in an LED tube by sparging argon for 10 minutes, then irradiated with a 10 W blue LED (λ = 405 nm) for 6 h. After the irradiation, the reaction mixture was diluted with Et_2O (10 mL). The organic layer was separated, and the aqueous layer was extracted with Et_2O (3 × 15 mL). The combined organic phase was washed with brine, dried over Na_2SO_4, and concentrated under reduced pressure at 30 ºC. The obtained crude product was then purified by flash column chromatography FCC (SiO_2, hexane/EtOAc = 95:5) to yield the 5-phenyldihydrofuran-2(3*H*)-one (**15**) as a colorless oil (48 mg, 296 µmol, 74%).
TLC (hexane /EtOAc = 98:2): R_f = 0.22 [UV, $KMnO_4$].
^{1}H-NMR (400 MHz, $CDCl_3$, 300 K): δ [ppm] = 7.45 – 7.29 (m, 5H, H2'-H4'), 5.57 – 5.47 (m, 1H, H5), 2.73 – 2.59 (m, 3H, H3, H4), 2.28 – 2.11 (m, 1H, H4).
^{13}C-NMR (101 MHz, $CDCl_3$, 300 K): δ [ppm] = 177.0 (C2), 139.5 (C1'), 128.9 (C2'), 128.6 (C4'), 125.4 (C3'), 81.3 (C5), 31.1 (C3), 29.1 (C4).
The spectroscopic data matches the one reported in the literature.[2]

S8. NMR Spectra

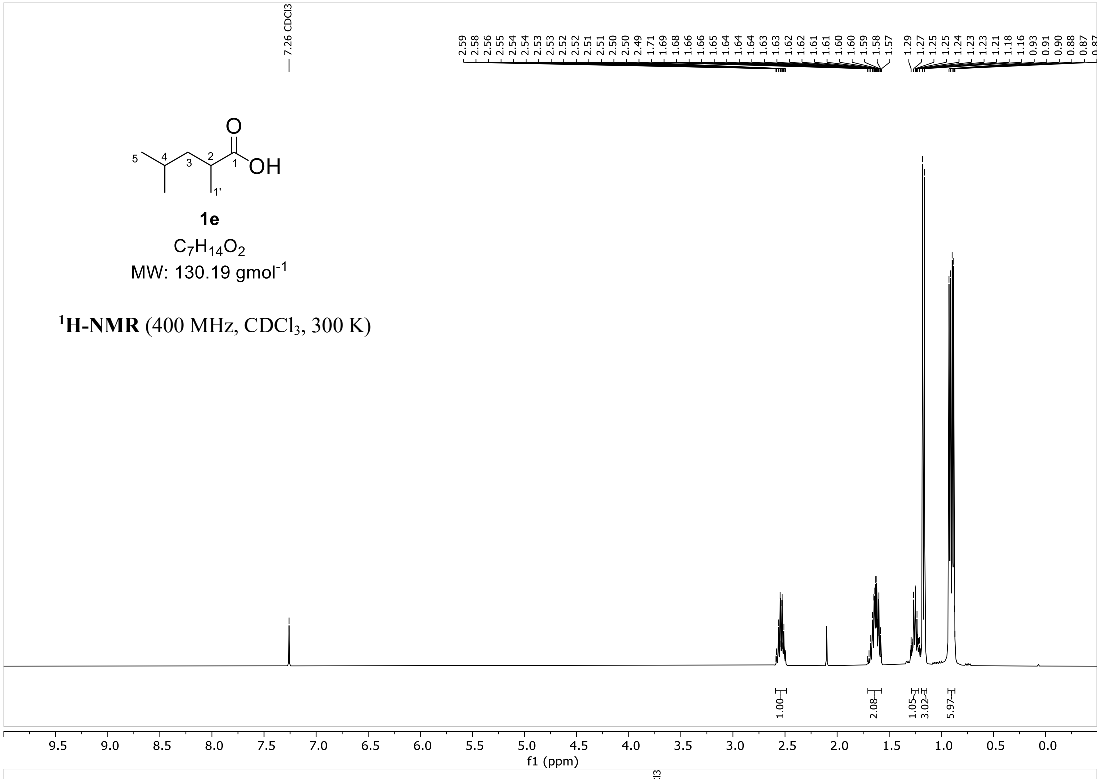

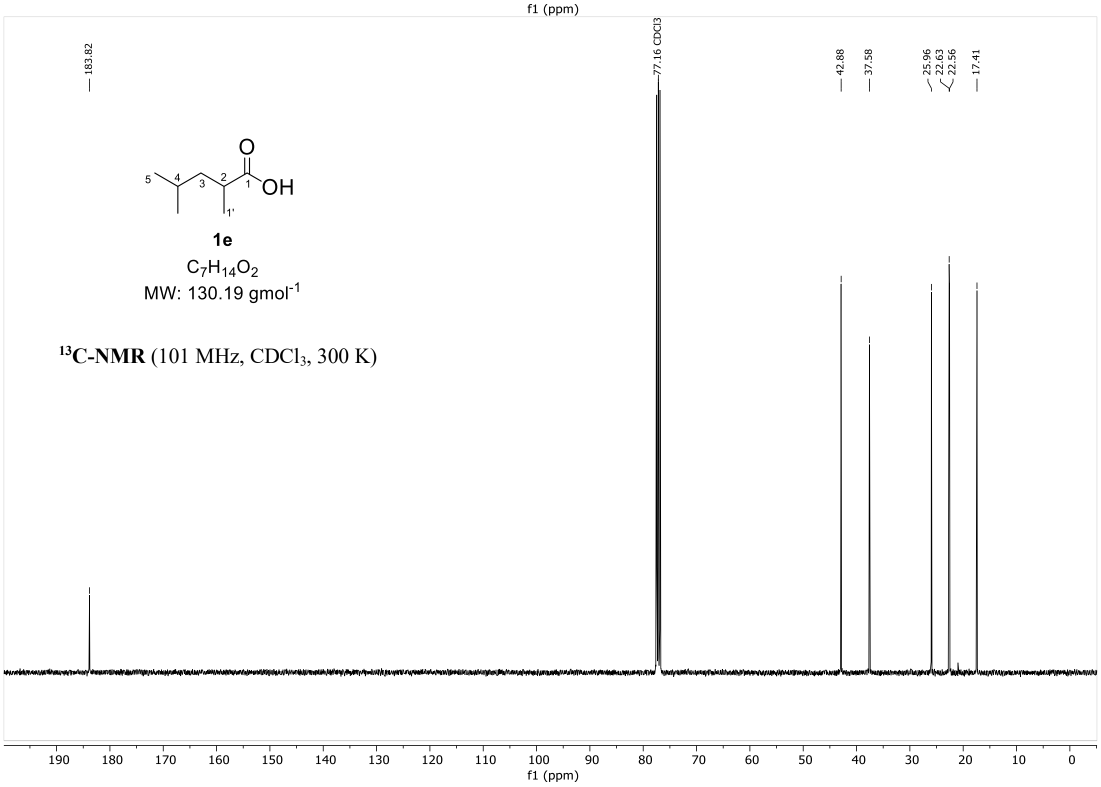

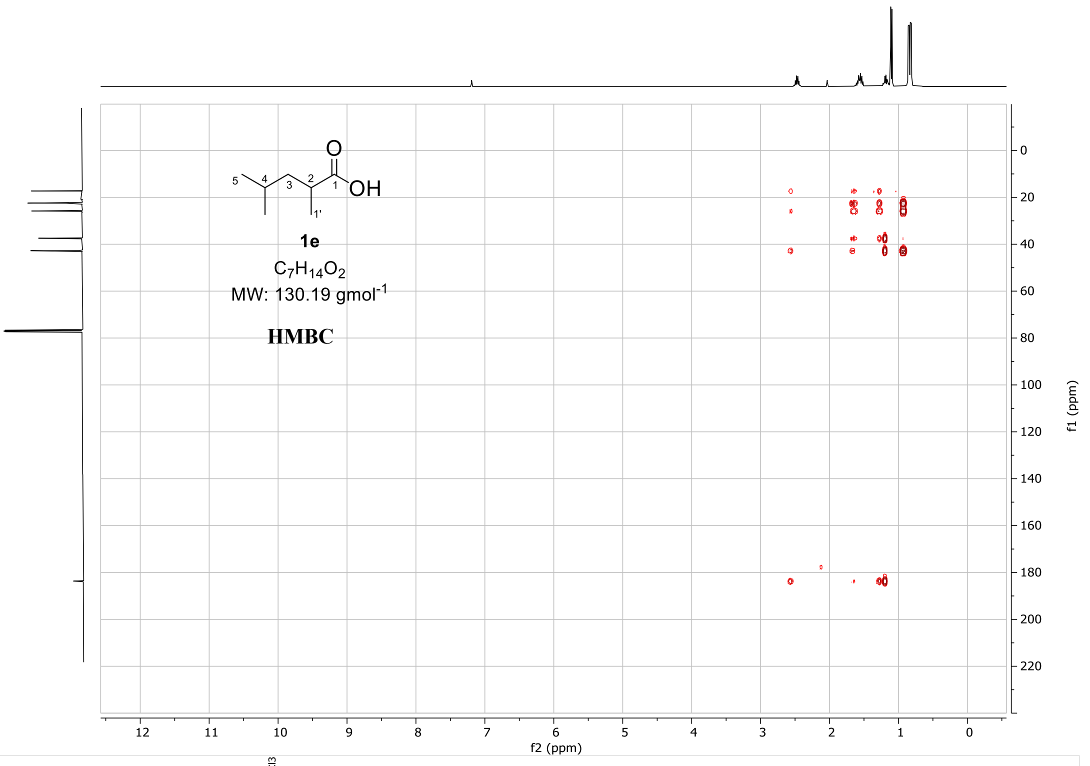
1e
C7H14O2
MW: 130.19 gmol-1
HMBC
f2 (ppm)
f1 (ppm)

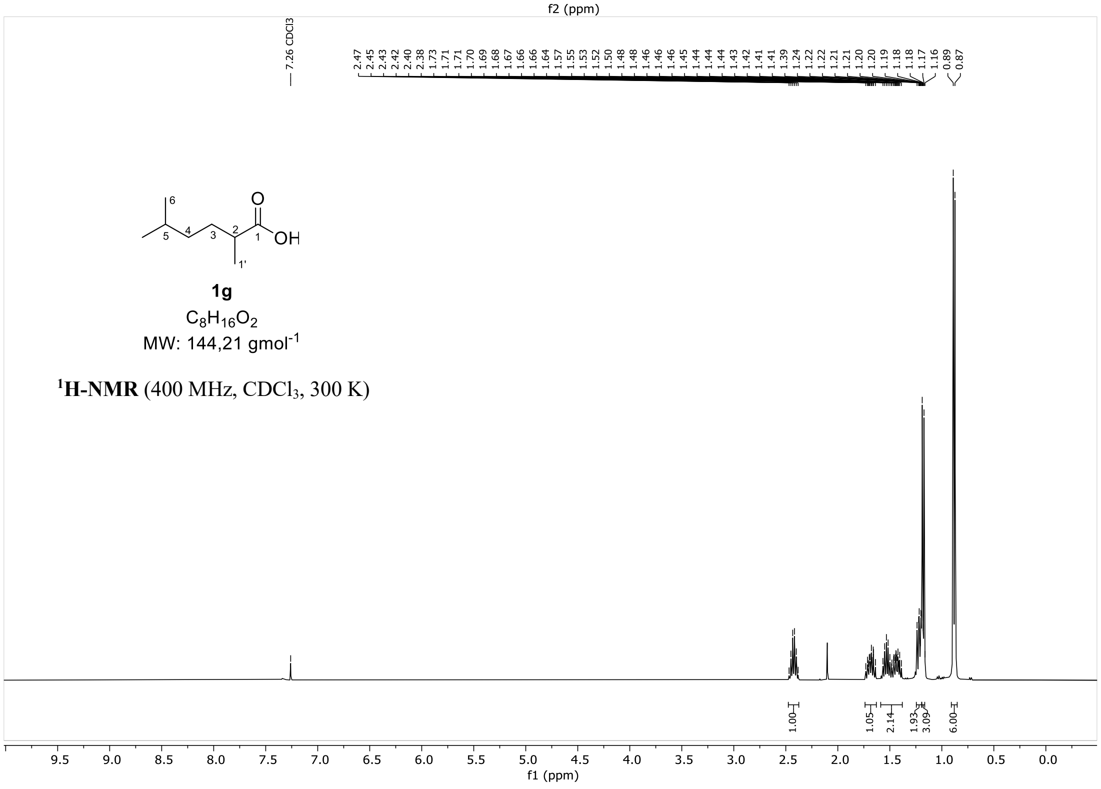
7.26 CDCl3
2.47 2.45 2.43 2.42 2.40 2.38 1.73 1.71 1.71 1.70 1.69 1.68 1.67 1.66 1.66 1.64 1.57 1.55 1.53 1.52 1.50 1.48 1.48 1.46 1.46 1.45 1.44 1.44 1.43 1.42 1.41 1.41 1.39 1.24 1.22 1.22 1.21 1.21 1.20 1.20 1.19 1.18 1.18 1.17 1.16 0.89 0.87
1g
C8H16O2
MW: 144,21 gmol-1
1H-NMR (400 MHz, CDCl3, 300 K)
1.00 1.05 2.14 1.93 3.09 6.00
f1 (ppm)

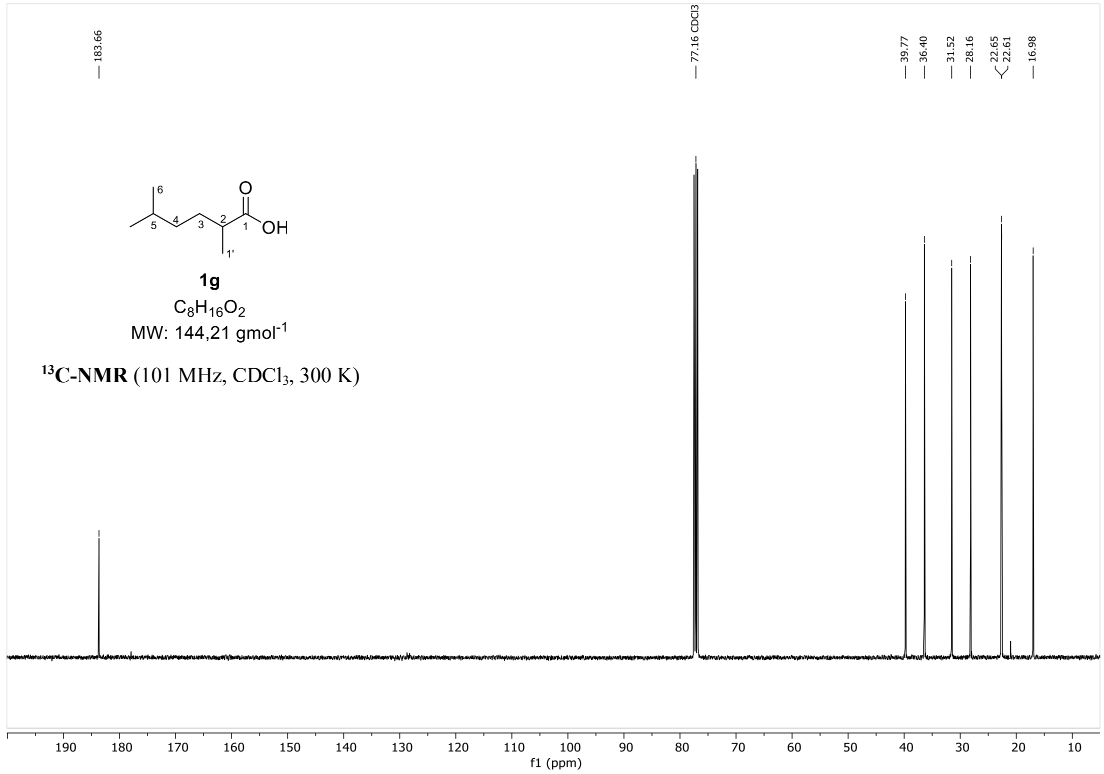
183.66
77.16 CDCl3
39.77
36.40
31.52
28.16
22.65
22.61
16.98
1g
$C_8H_{16}O_2$
MW: 144,21 gmol^{-1}
^{13}C-NMR (101 MHz, CDCl$_3$, 300 K)
f1 (ppm)

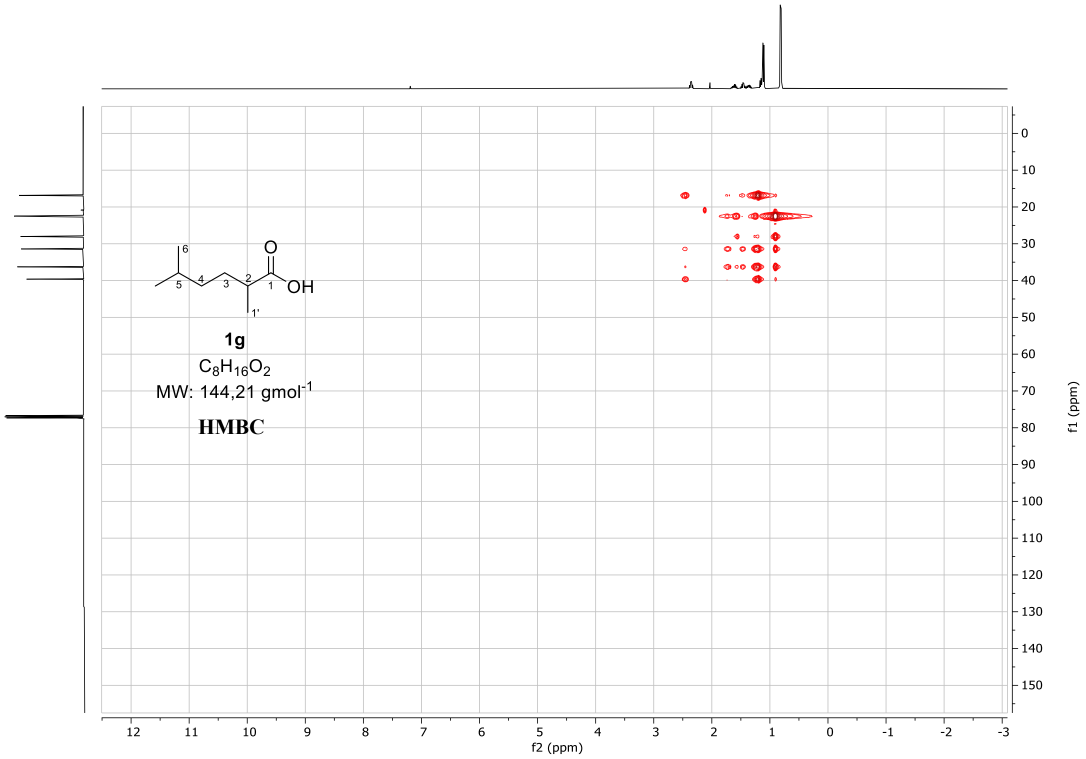
1g
$C_8H_{16}O_2$
MW: 144,21 gmol^{-1}
HMBC
f2 (ppm)
f1 (ppm)

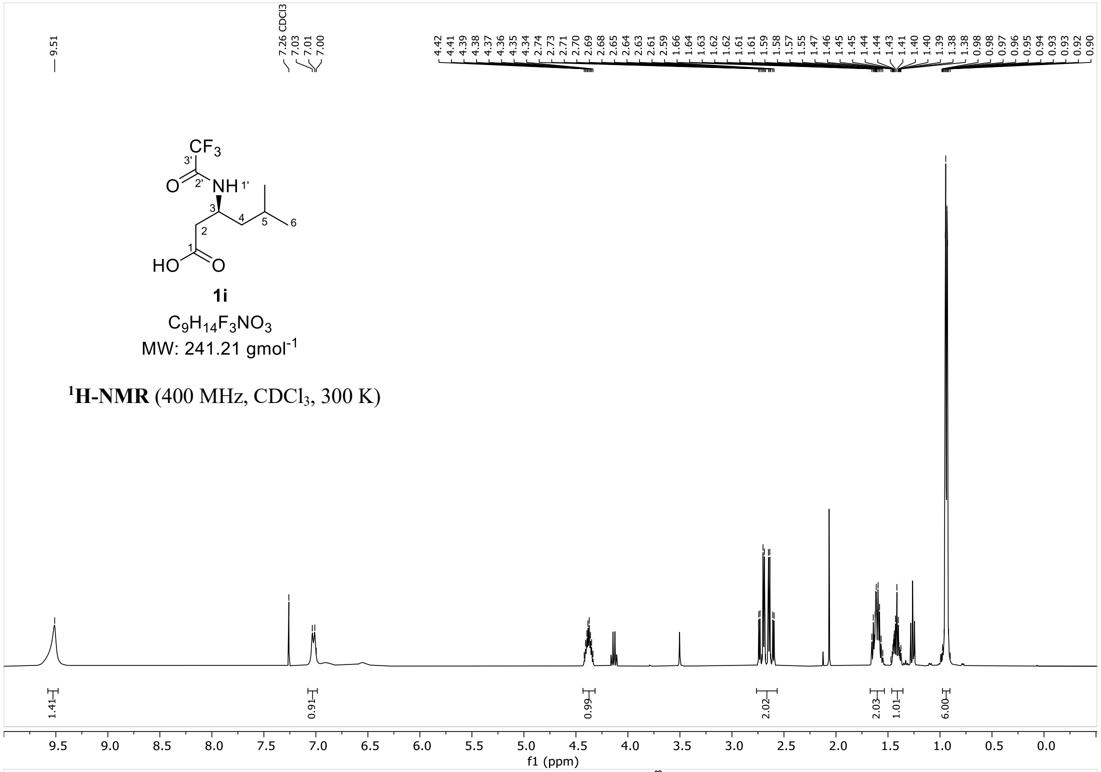

1i
$C_9H_{14}F_3NO_3$
MW: 241.21 gmol^{-1}
^{1}H-NMR (400 MHz, $CDCl_3$, 300 K)
9.51
7.26 CDCl3
7.03
7.01
7.00
4.42 4.41 4.39 4.38 4.37 4.36 4.35 4.34 2.74 2.73 2.71 2.70 2.69 2.68 2.65 2.64 2.63 2.61 2.59 1.66 1.64 1.63 1.63 1.62 1.62 1.61 1.61 1.59 1.58 1.57 1.55 1.47 1.46 1.45 1.45 1.44 1.44 1.43 1.41 1.40 1.40 1.39 1.38 1.38 0.98 0.98 0.97 0.96 0.96 0.95 0.94 0.93 0.93 0.92 0.90
1.41
0.91
0.99
2.02
2.03
1.01
6.00
f1 (ppm)

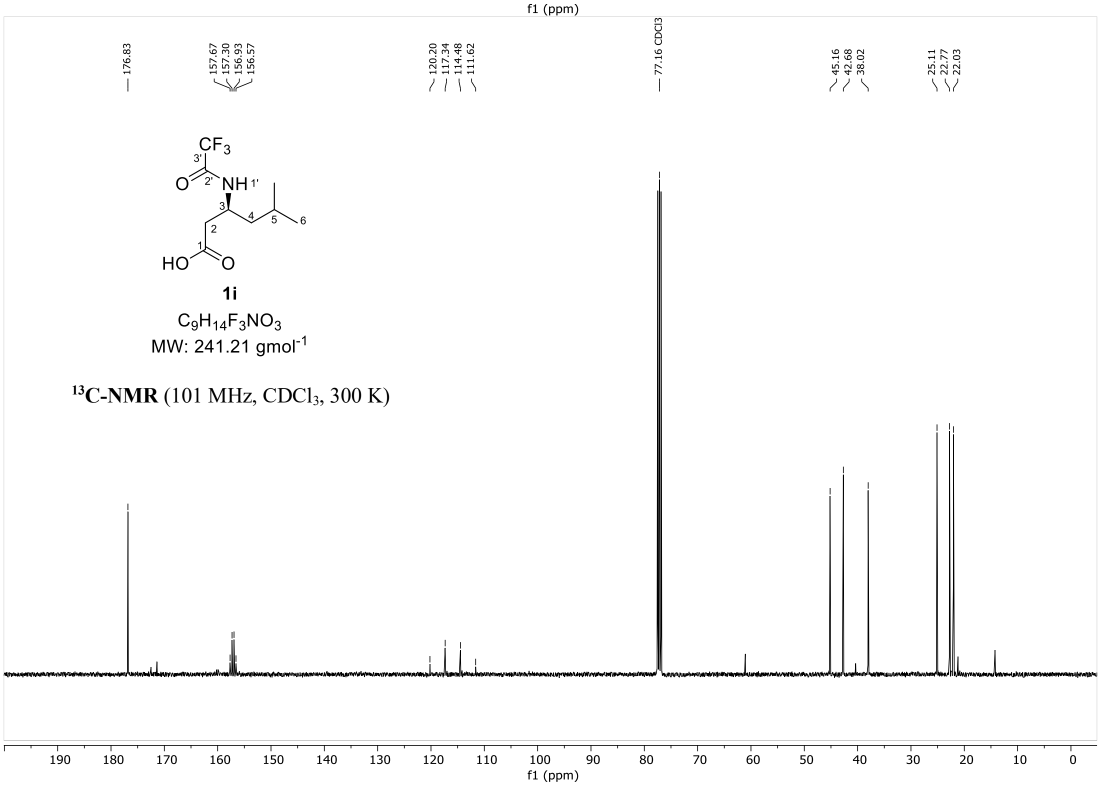

1i
$C_9H_{14}F_3NO_3$
MW: 241.21 gmol^{-1}
^{13}C-NMR (101 MHz, $CDCl_3$, 300 K)
176.83
157.67 157.30 156.93 156.57
120.20 117.34 114.48 111.62
77.16 CDCl3
45.16 42.68 38.02
25.11 22.77 22.03
f1 (ppm)

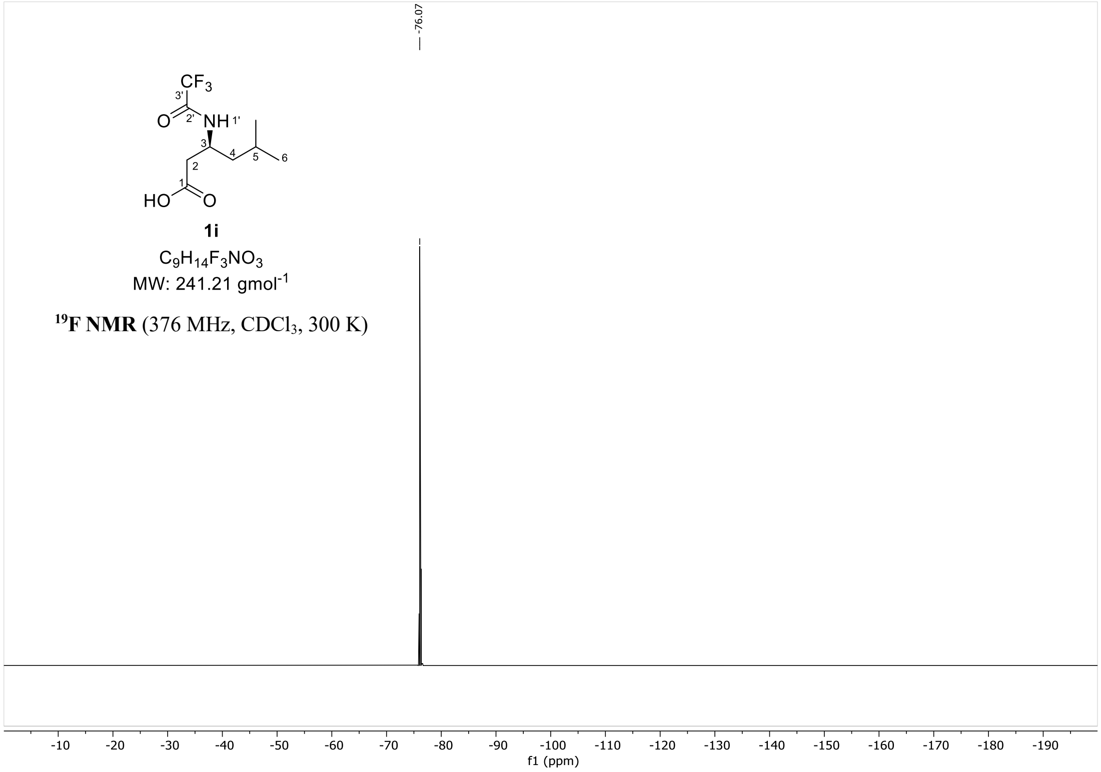
-76.07
CF3
3'
2'
O
NH 1'
3
4
5
6
2
1
HO
O
1i
C9H14F3NO3
MW: 241.21 gmol-1
19F NMR (376 MHz, CDCl3, 300 K)
-10 -20 -30 -40 -50 -60 -70 -80 -90 -100 -110 -120 -130 -140 -150 -160 -170 -180 -190
f1 (ppm)

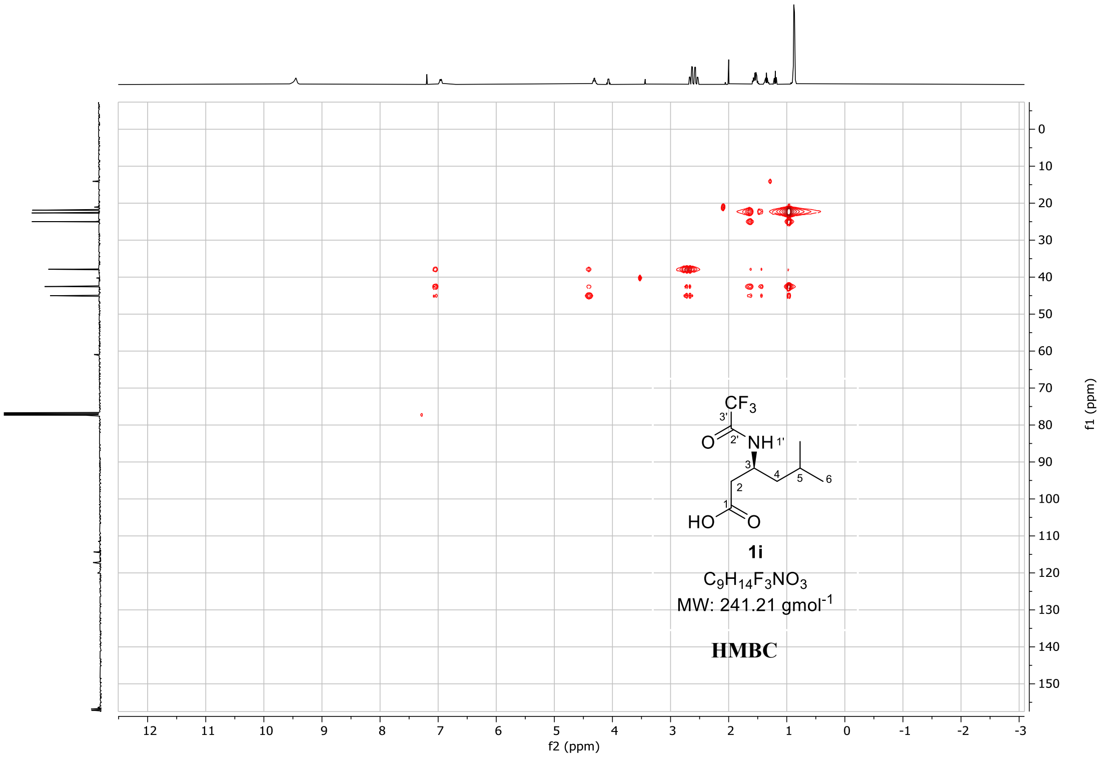
CF3
3'
2'
O
NH 1'
3
4
5
6
2
1
HO
O
1i
C9H14F3NO3
MW: 241.21 gmol-1
HMBC
0 10 20 30 40 50 60 70 80 90 100 110 120 130 140 150
f1 (ppm)
12 11 10 9 8 7 6 5 4 3 2 1 0 -1 -2 -3
f2 (ppm)

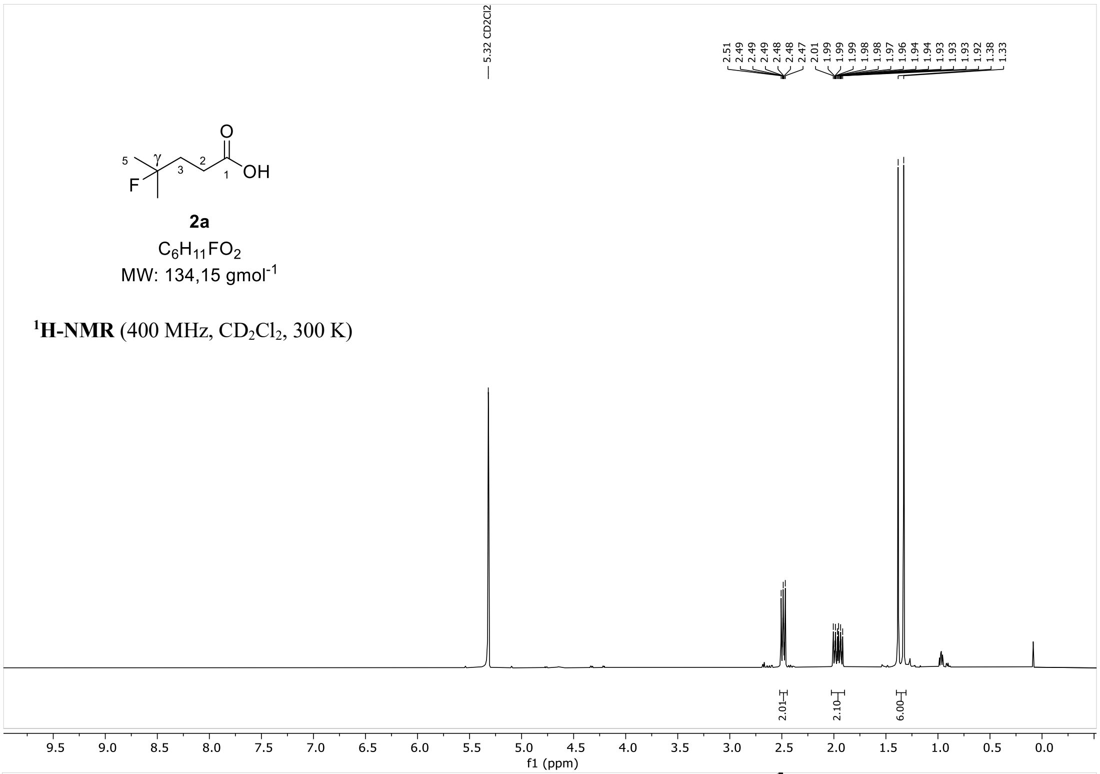
5.32 CD2Cl2
2.51
2.49
2.49
2.49
2.48
2.48
2.47
2.01
1.99
1.99
1.99
1.98
1.98
1.97
1.96
1.94
1.94
1.93
1.93
1.93
1.92
1.38
1.33
5
γ
2
3
1
O
OH
F
2a
C6H11FO2
MW: 134,15 gmol-1
1H-NMR (400 MHz, CD2Cl2, 300 K)
2.01
2.10
6.00
9.5 9.0 8.5 8.0 7.5 7.0 6.5 6.0 5.5 5.0 4.5 4.0 3.5 3.0 2.5 2.0 1.5 1.0 0.5 0.0
f1 (ppm)

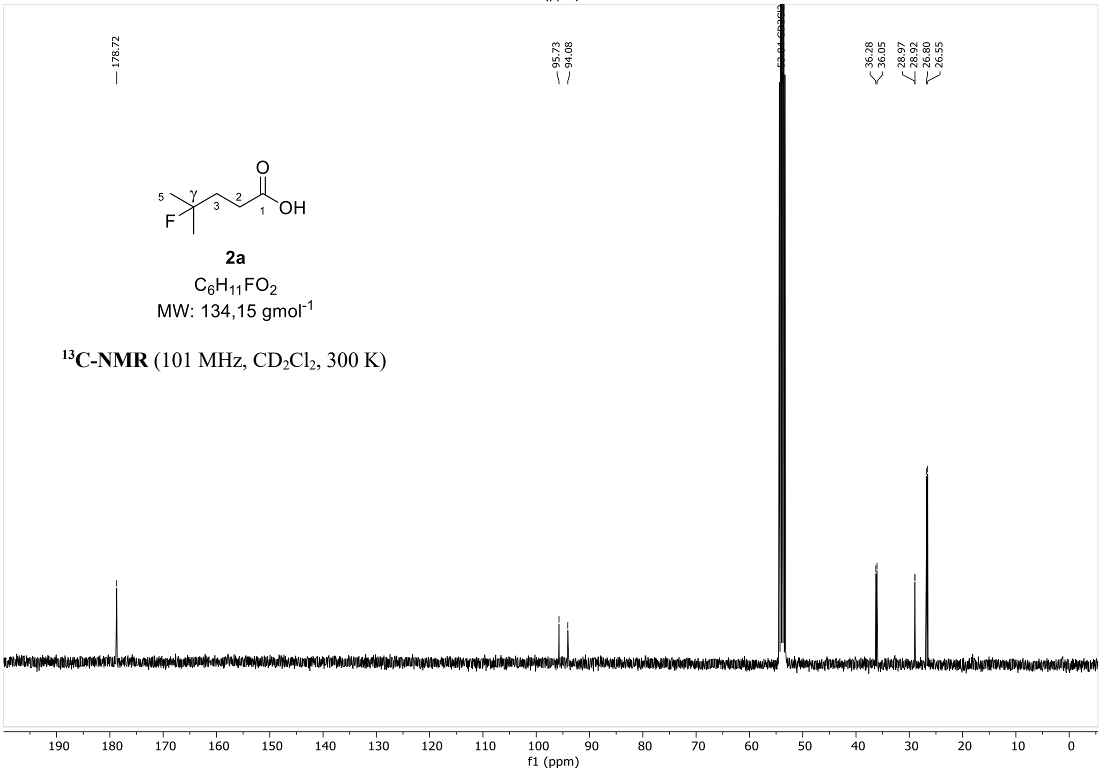
178.72
95.73
94.08
36.28
36.05
28.97
28.92
26.80
26.55
5
γ
2
3
1
O
OH
F
2a
C6H11FO2
MW: 134,15 gmol-1
13C-NMR (101 MHz, CD2Cl2, 300 K)
190 180 170 160 150 140 130 120 110 100 90 80 70 60 50 40 30 20 10 0
f1 (ppm)

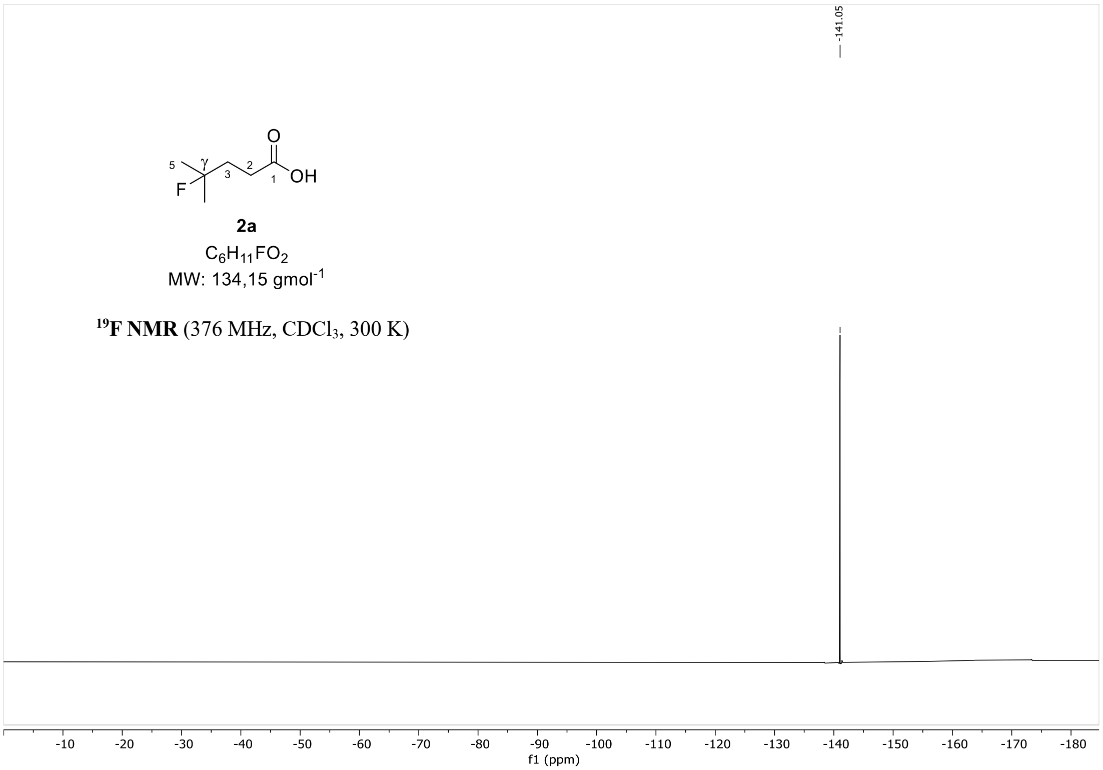

-141.05
O
OH
5
γ
3
2
1
F
2a
C6H11FO2
MW: 134,15 gmol-1
19F NMR (376 MHz, CDCl3, 300 K)
f1 (ppm)

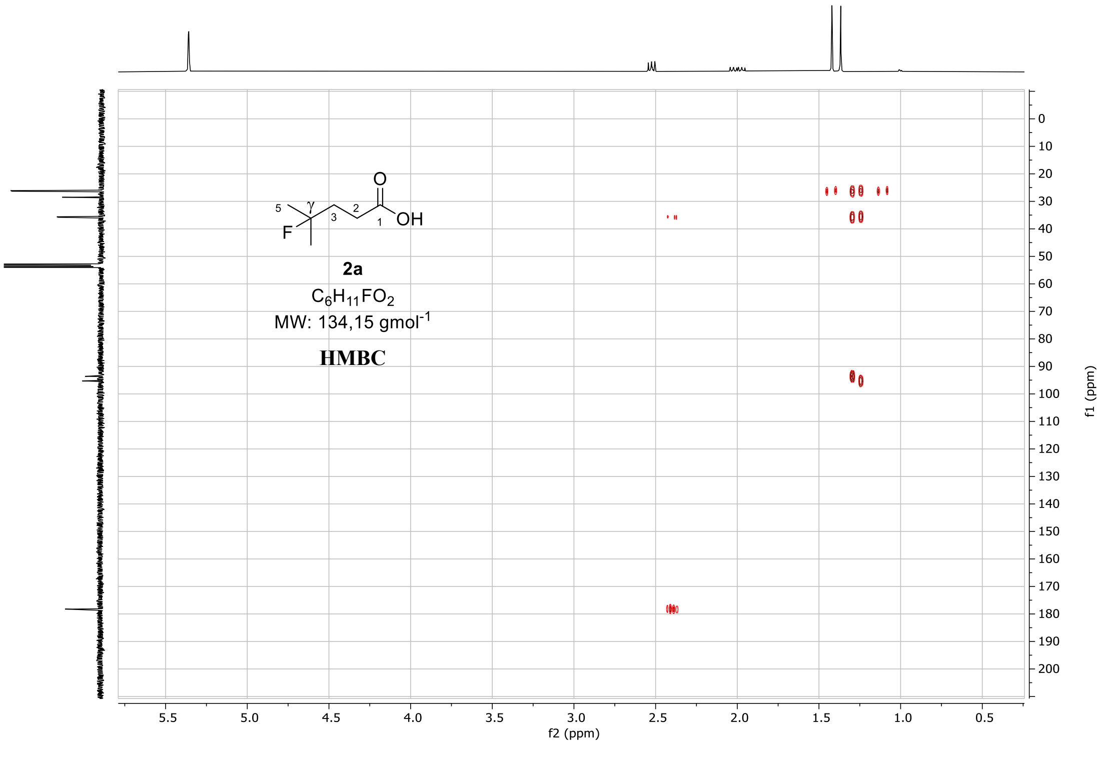

O
OH
5
γ
3
2
1
F
2a
C6H11FO2
MW: 134,15 gmol-1
HMBC
f2 (ppm)
f1 (ppm)

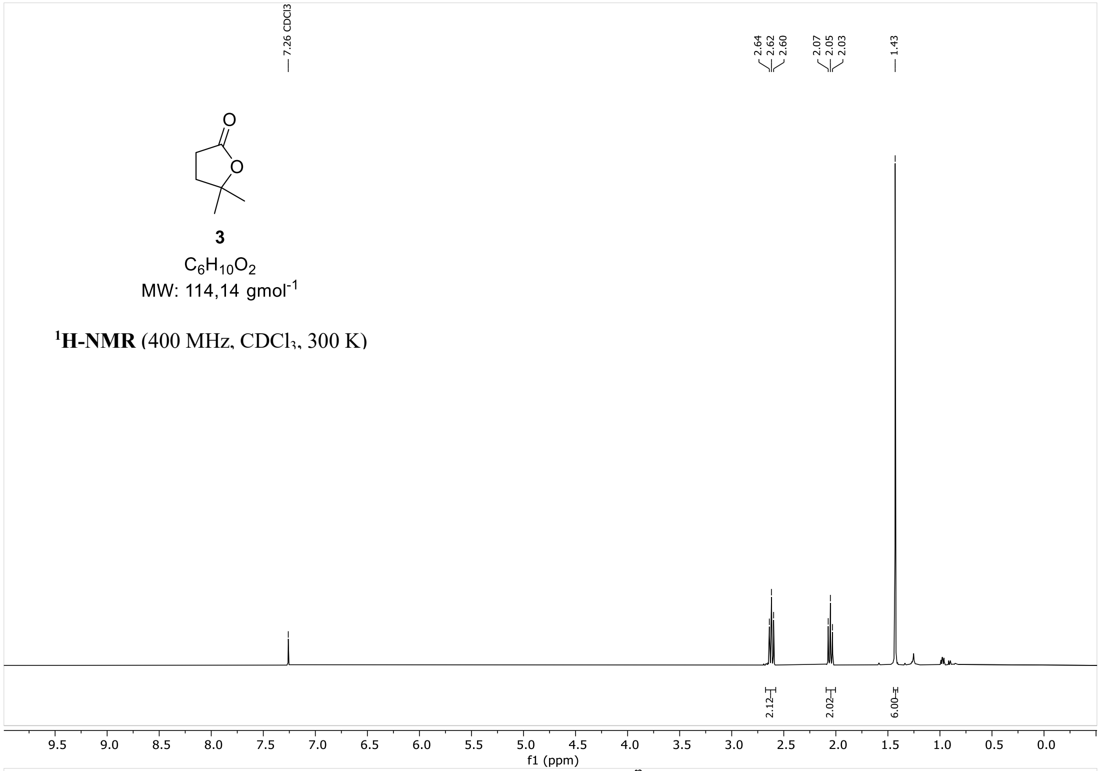
7.26 CDCl3
2.64
2.62
2.60
2.07
2.05
2.03
1.43
3
C6H10O2
MW: 114,14 gmol-1
1H-NMR (400 MHz, CDCl3, 300 K)
2.12
2.02
6.00
9.5 9.0 8.5 8.0 7.5 7.0 6.5 6.0 5.5 5.0 4.5 4.0 3.5 3.0 2.5 2.0 1.5 1.0 0.5 0.0
f1 (ppm)

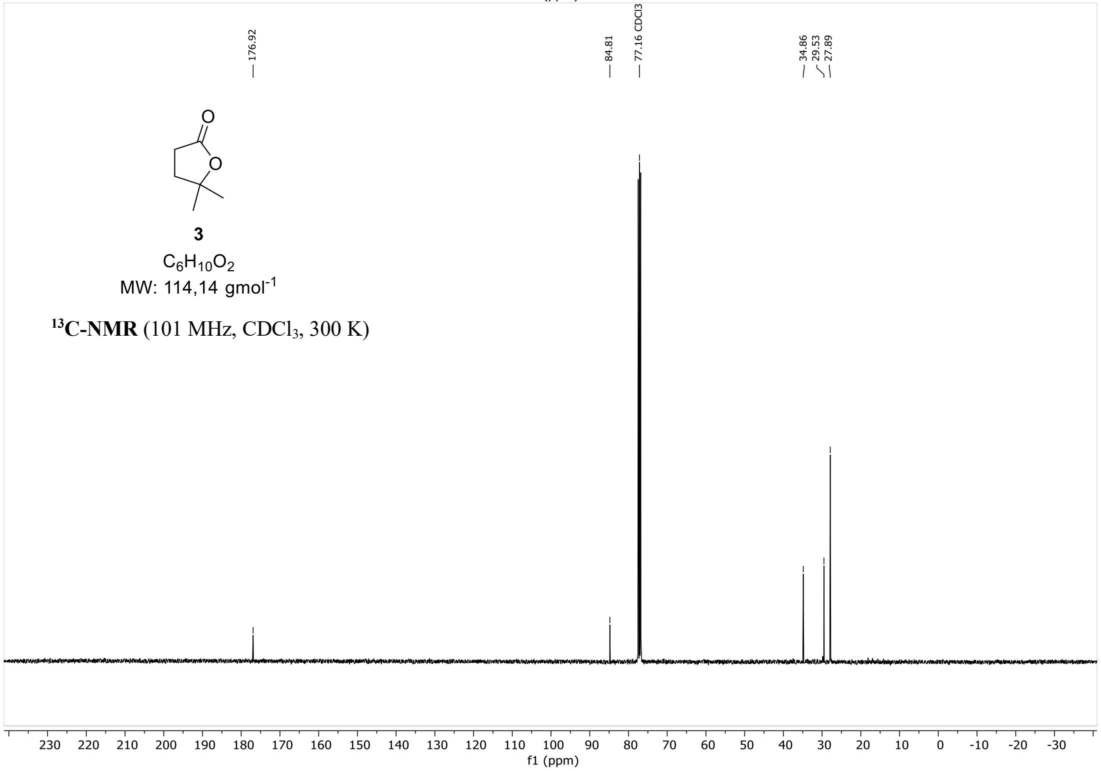
176.92
84.81
77.16 CDCl3
34.86
29.53
27.89
3
C6H10O2
MW: 114,14 gmol-1
13C-NMR (101 MHz, CDCl3, 300 K)
230 220 210 200 190 180 170 160 150 140 130 120 110 100 90 80 70 60 50 40 30 20 10 0 -10 -20 -30
f1 (ppm)

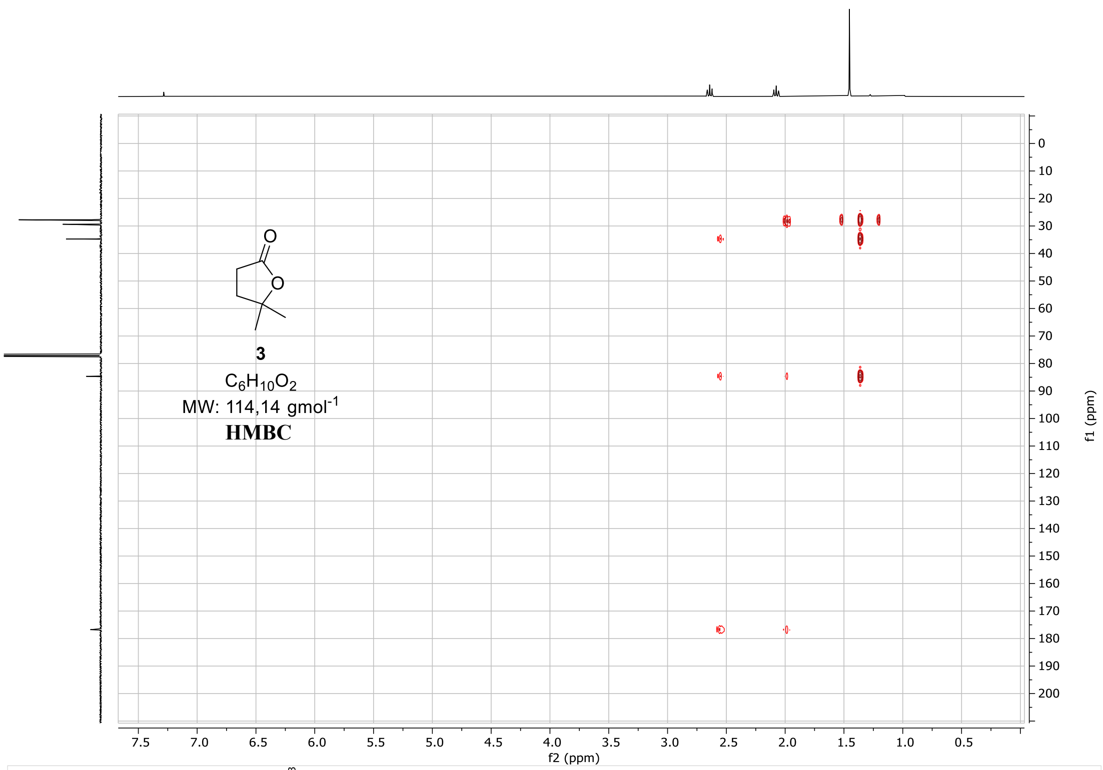
3
$C_6H_{10}O_2$
MW: 114,14 gmol^{-1}
HMBC
f2 (ppm)
f1 (ppm)

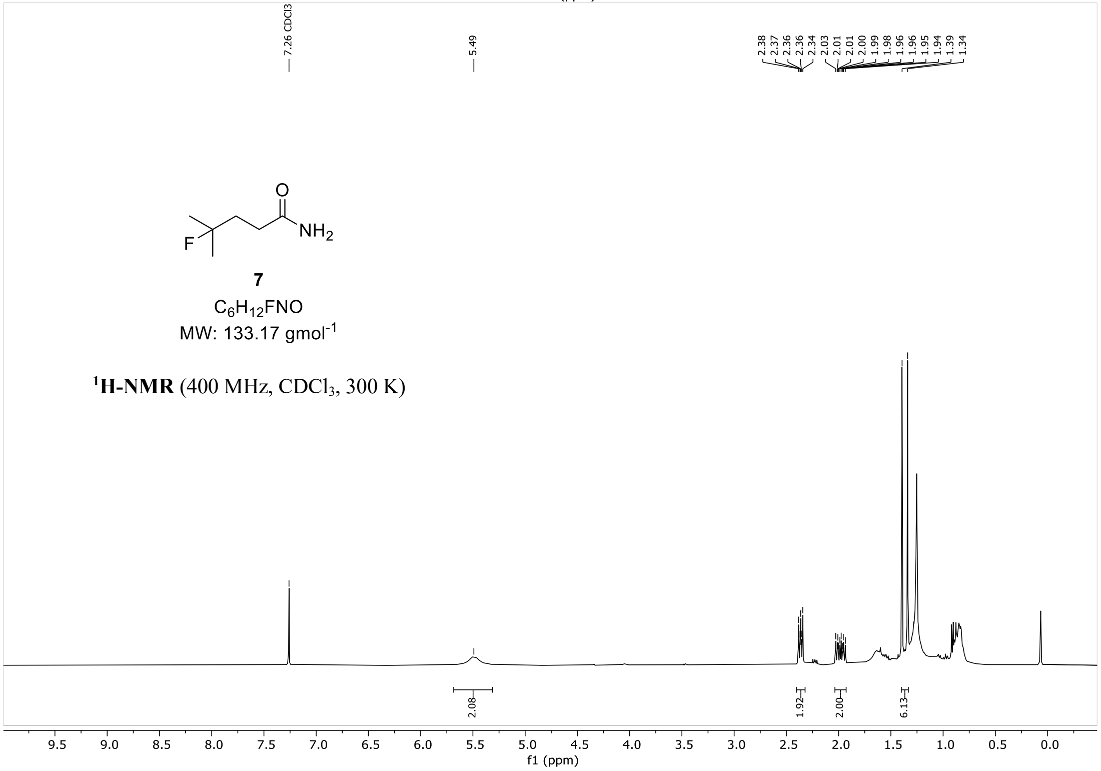
7.26 CDCl3
5.49
2.38 2.37 2.36 2.36 2.34 2.03 2.01 2.01 2.00 1.99 1.98 1.96 1.96 1.95 1.94 1.39 1.34
7
$C_6H_{12}FNO$
MW: 133.17 gmol^{-1}
^{1}H-NMR (400 MHz, $CDCl_3$, 300 K)
2.08
1.92
2.00
6.13
f1 (ppm)

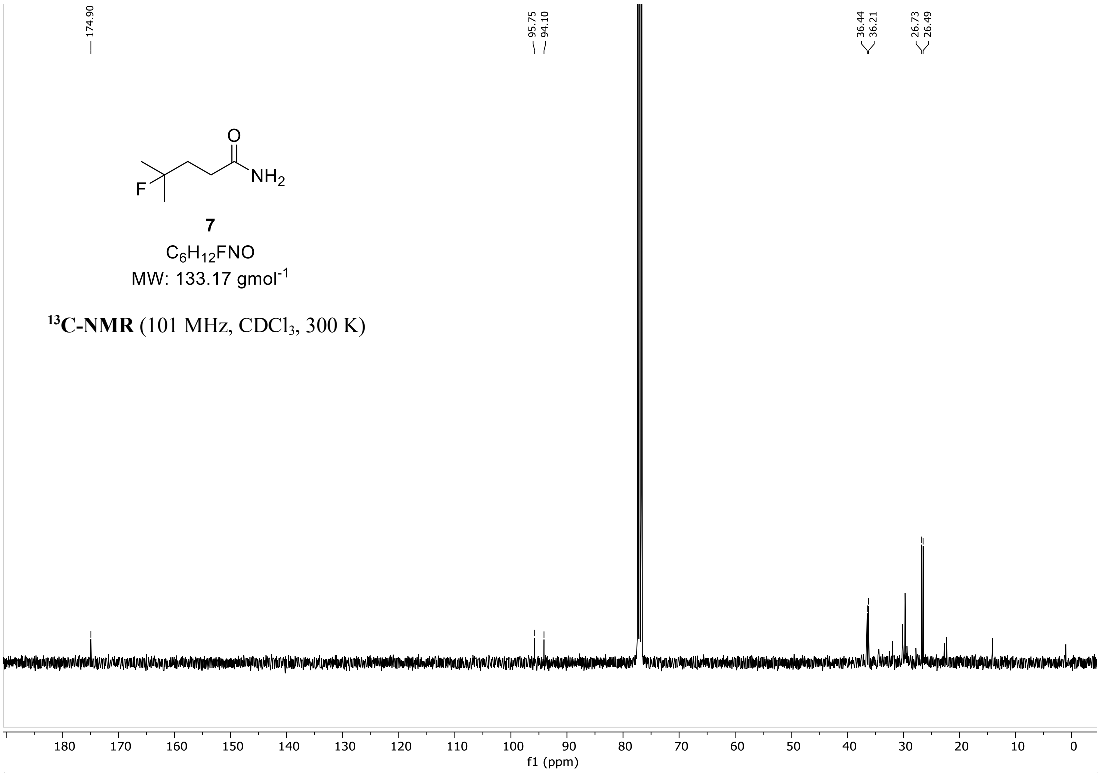
174.90
95.75
94.10
36.44
36.21
26.73
26.49
O
NH2
F
7
C6H12FNO
MW: 133.17 gmol-1
13C-NMR (101 MHz, CDCl3, 300 K)
180 170 160 150 140 130 120 110 100 90 80 70 60 50 40 30 20 10 0
f1 (ppm)

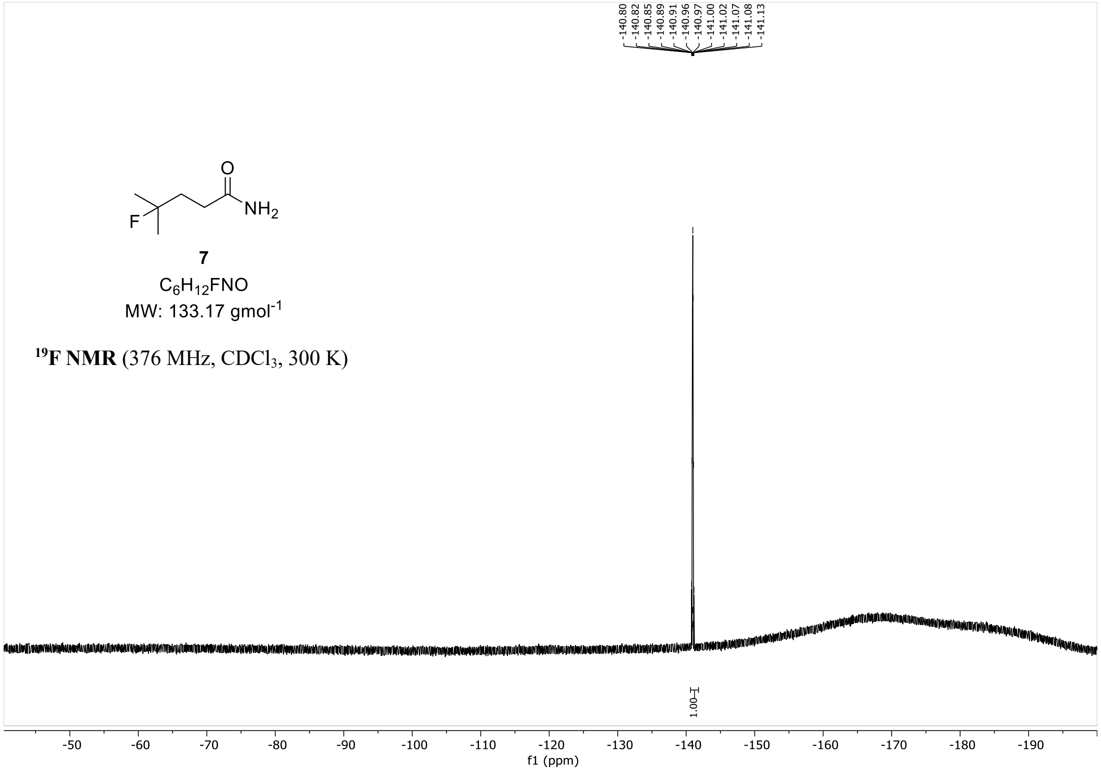
-140.80
-140.82
-140.85
-140.89
-140.91
-140.96
-140.97
-141.00
-141.02
-141.07
-141.08
-141.13
O
NH2
F
7
C6H12FNO
MW: 133.17 gmol-1
19F NMR (376 MHz, CDCl3, 300 K)
1.00
-50 -60 -70 -80 -90 -100 -110 -120 -130 -140 -150 -160 -170 -180 -190
f1 (ppm)

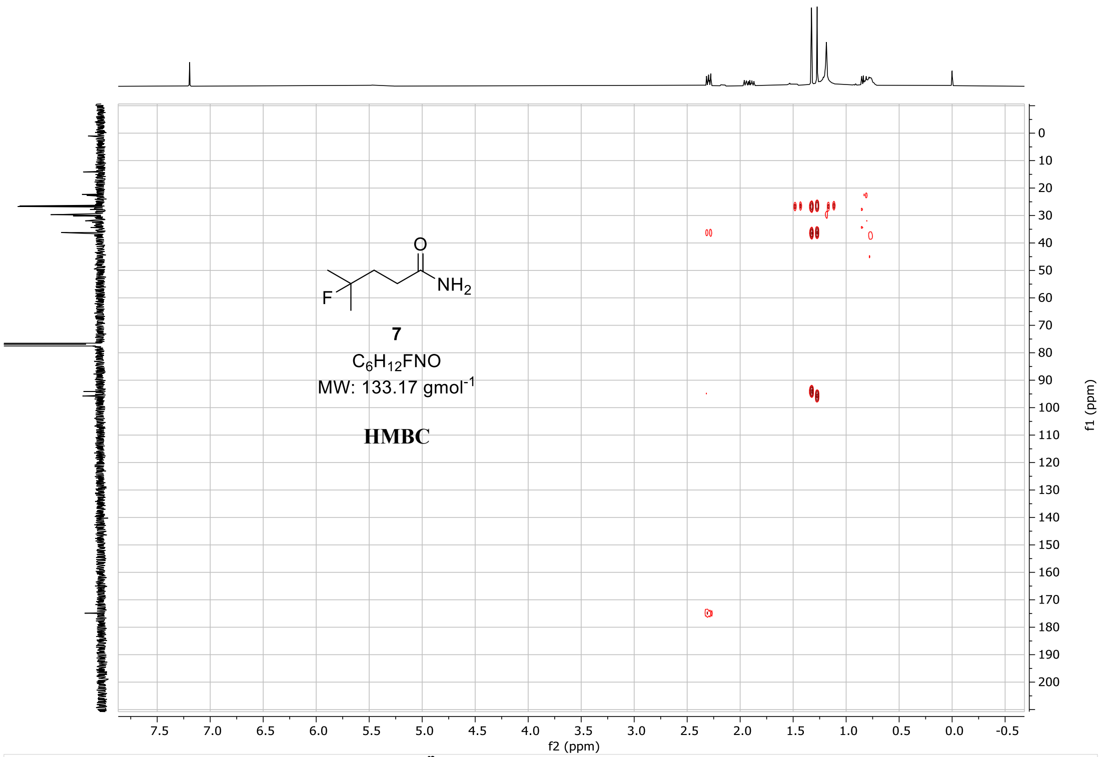

O
NH2
F
7
C6H12FNO
MW: 133.17 gmol-1
HMBC
f2 (ppm)
f1 (ppm)

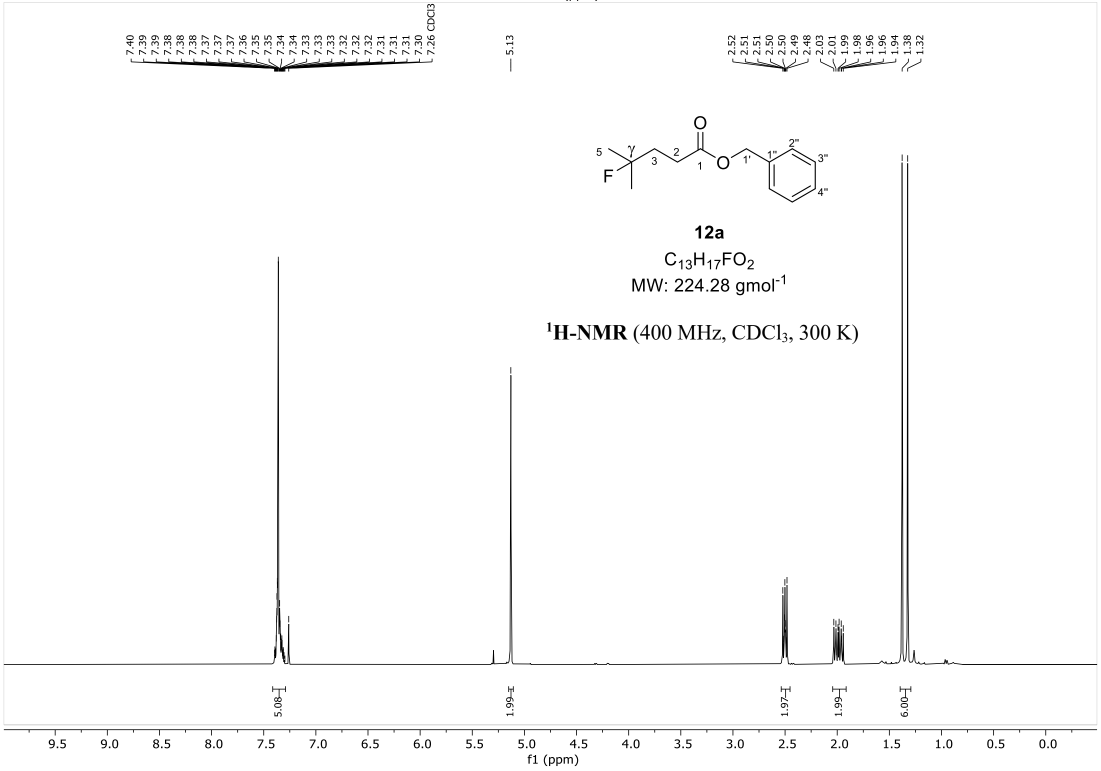

7.40 7.39 7.39 7.38 7.38 7.37 7.37 7.37 7.36 7.35 7.35 7.34 7.34 7.33 7.33 7.33 7.32 7.32 7.32 7.31 7.31 7.31 7.30 7.26 CDCl3
5.13
2.52 2.51 2.51 2.50 2.50 2.49 2.48 2.03 2.01 1.99 1.98 1.96 1.96 1.94 1.38 1.32
12a
C13H17FO2
MW: 224.28 gmol-1
1H-NMR (400 MHz, CDCl3, 300 K)
5.08
1.99
1.97
1.99
6.00
f1 (ppm)

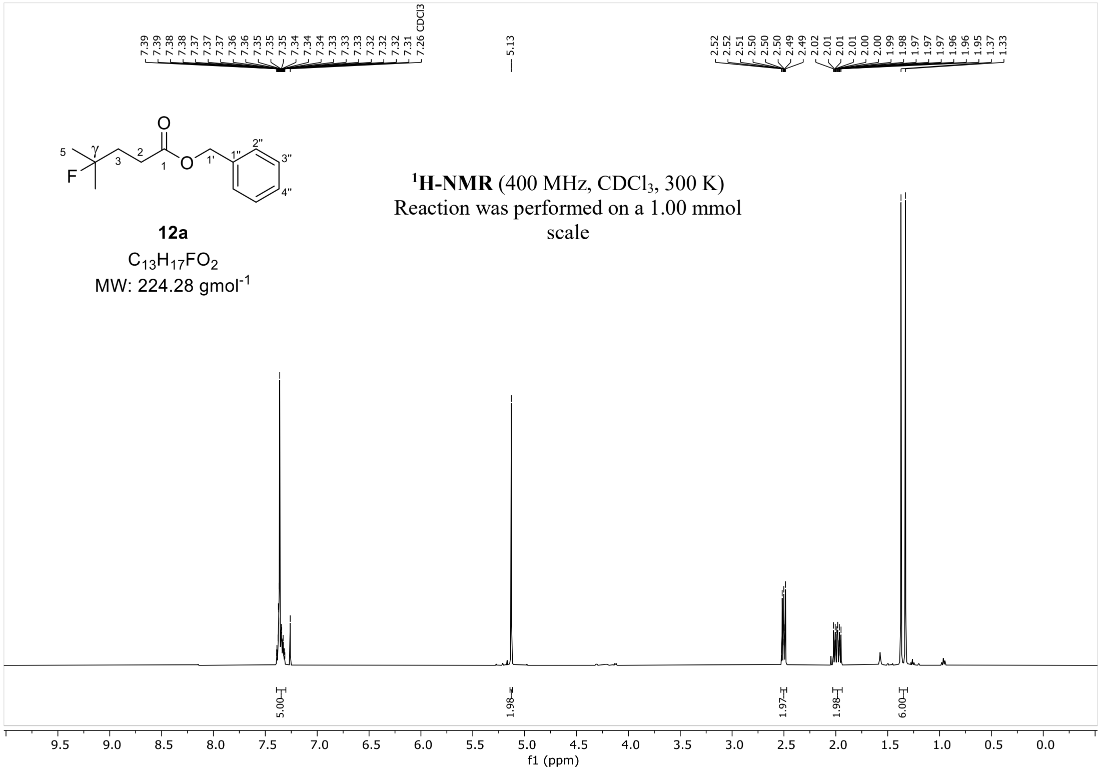
12a
$C_{13}H_{17}FO_2$
MW: 224.28 gmol-1
1H-NMR (400 MHz, CDCl3, 300 K)
Reaction was performed on a 1.00 mmol scale
f1 (ppm)

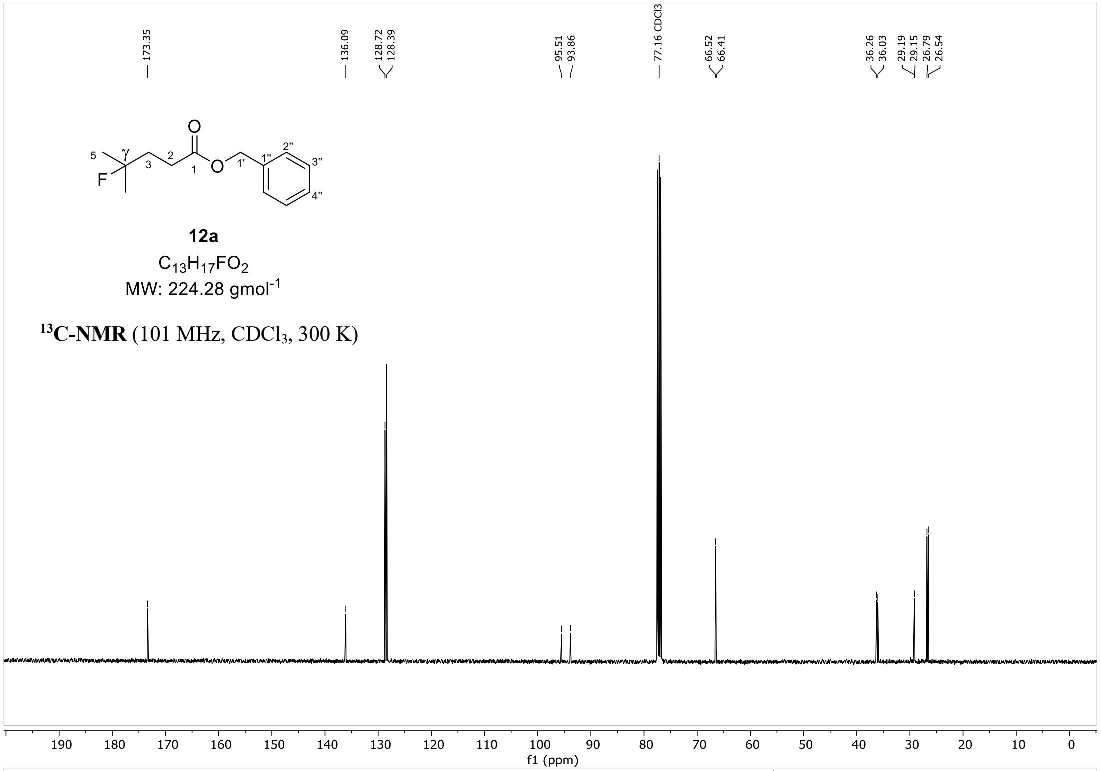
173.35
136.09
128.72
128.39
95.51
93.86
77.16 CDCl3
66.52
66.41
36.26
36.03
29.19
29.15
26.79
26.54
12a
$C_{13}H_{17}FO_2$
MW: 224.28 gmol^{-1}
^{13}C-NMR (101 MHz, $CDCl_3$, 300 K)
f1 (ppm)

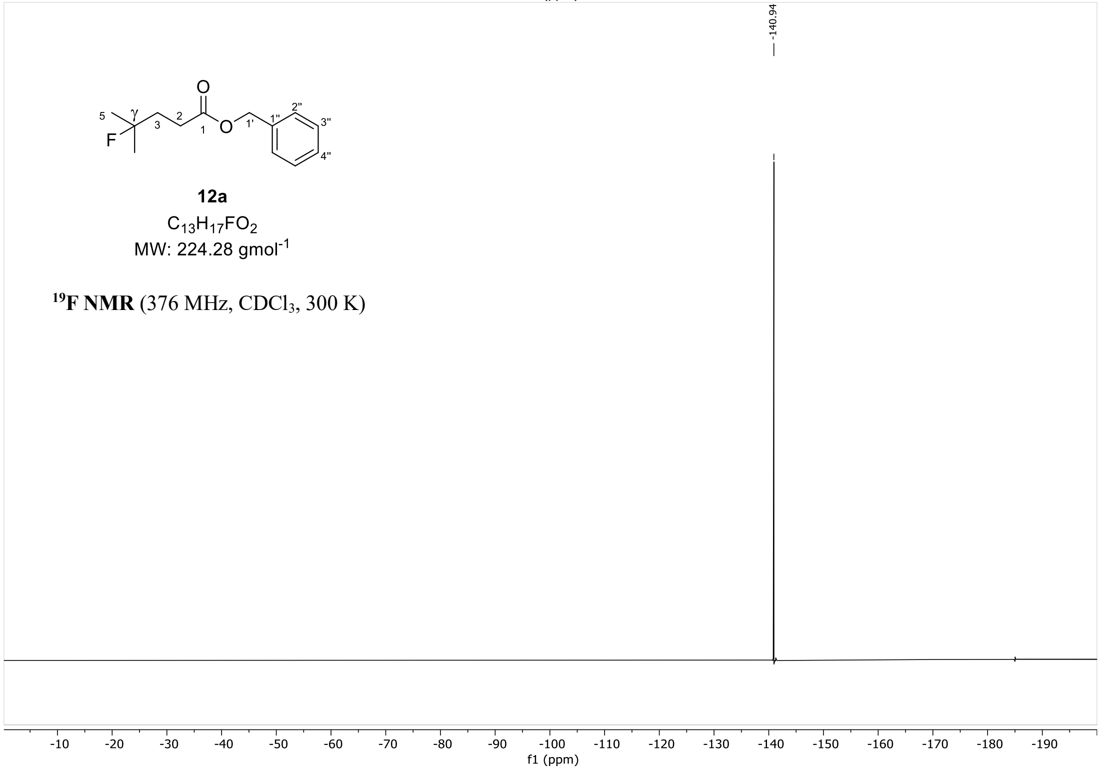
-140.94
12a
$C_{13}H_{17}FO_2$
MW: 224.28 gmol^{-1}
^{19}F NMR (376 MHz, $CDCl_3$, 300 K)
f1 (ppm)

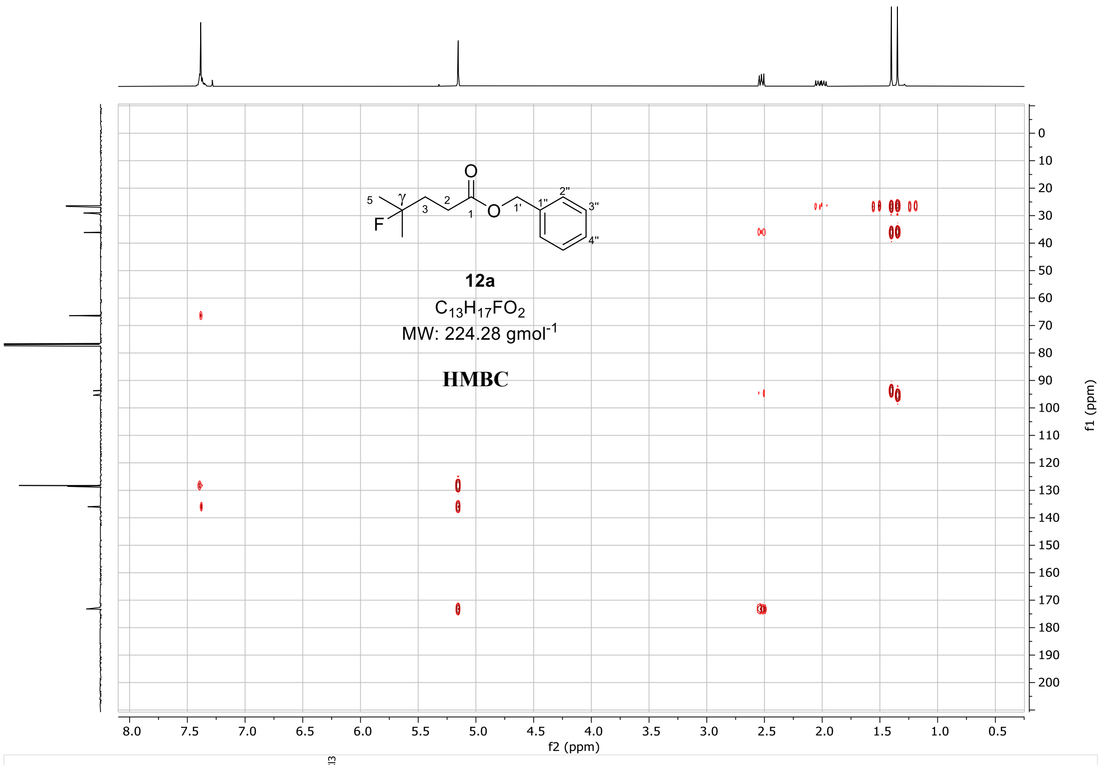
12a
$C_{13}H_{17}FO_2$
MW: 224.28 gmol^{-1}
HMBC
f2 (ppm)
f1 (ppm)

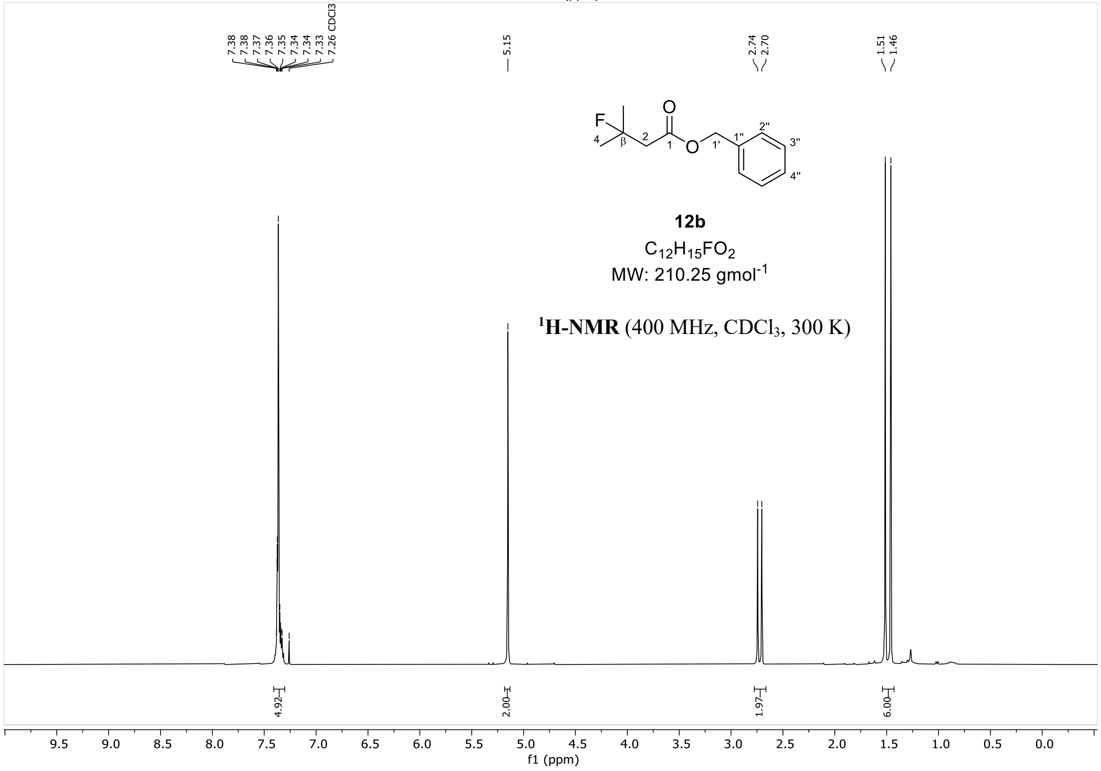
7.38
7.38
7.37
7.36
7.35
7.34
7.34
7.33
7.26 CDCl3
5.15
2.74
2.70
1.51
1.46
12b
$C_{12}H_{15}FO_2$
MW: 210.25 gmol^{-1}
^{1}H-NMR (400 MHz, $CDCl_3$, 300 K)
4.92
2.00
1.97
6.00
f1 (ppm)

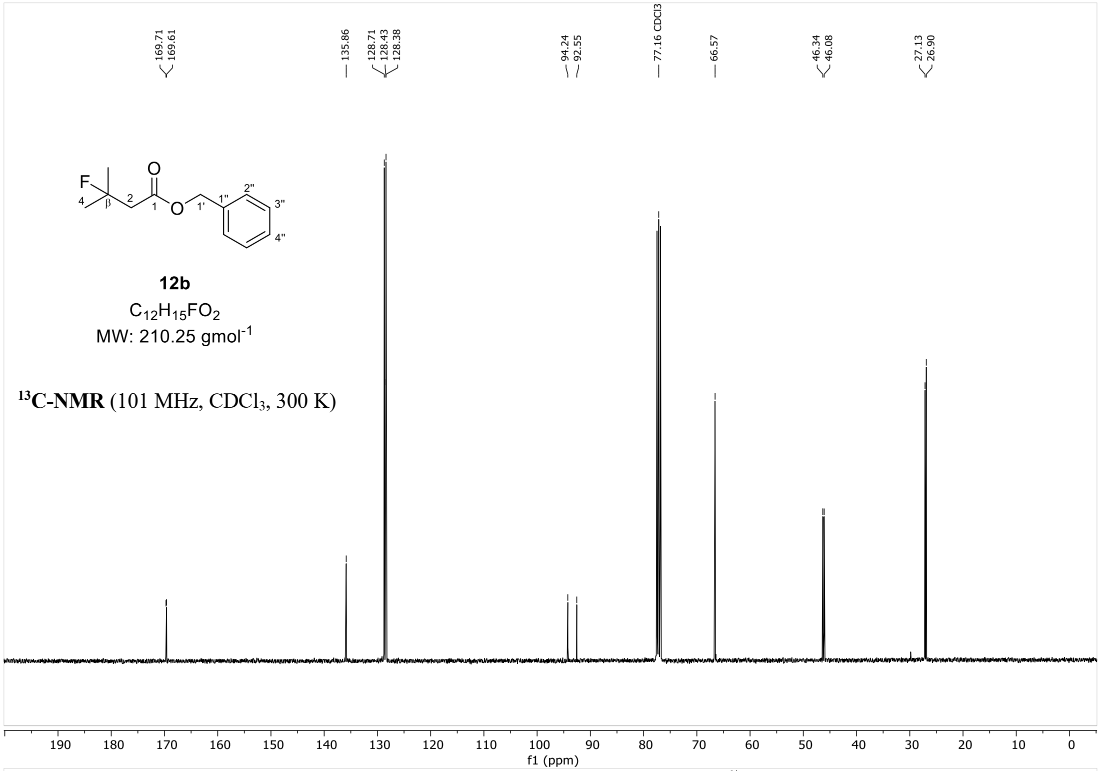
169.71
169.61
135.86
128.71
128.43
128.38
94.24
92.55
77.16 CDCl3
66.57
46.34
46.08
27.13
26.90
F
4
β
2
O
1
O
1'
1''
2''
3''
4''
12b
$C_{12}H_{15}FO_2$
MW: 210.25 gmol^{-1}
^{13}C-NMR (101 MHz, $CDCl_3$, 300 K)
190 180 170 160 150 140 130 120 110 100 90 80 70 60 50 40 30 20 10 0
f1 (ppm)

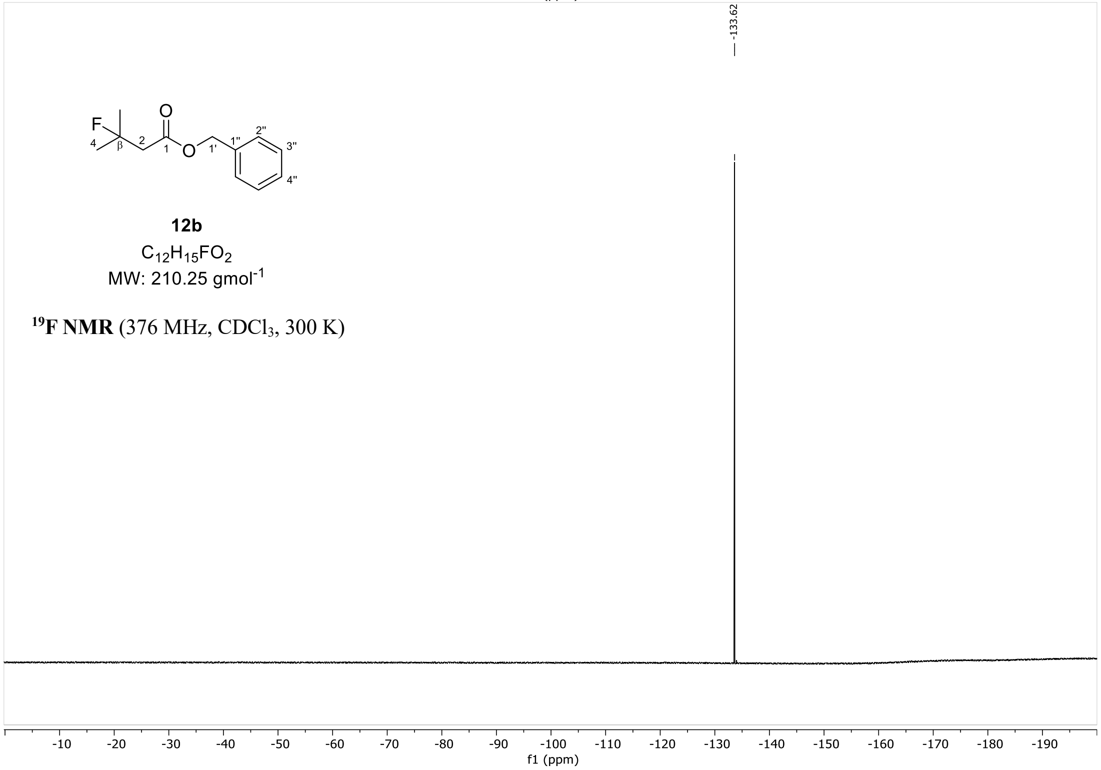
-133.62
F
4
β
2
O
1
O
1'
1''
2''
3''
4''
12b
$C_{12}H_{15}FO_2$
MW: 210.25 gmol^{-1}
^{19}F NMR (376 MHz, $CDCl_3$, 300 K)
-10 -20 -30 -40 -50 -60 -70 -80 -90 -100 -110 -120 -130 -140 -150 -160 -170 -180 -190
f1 (ppm)

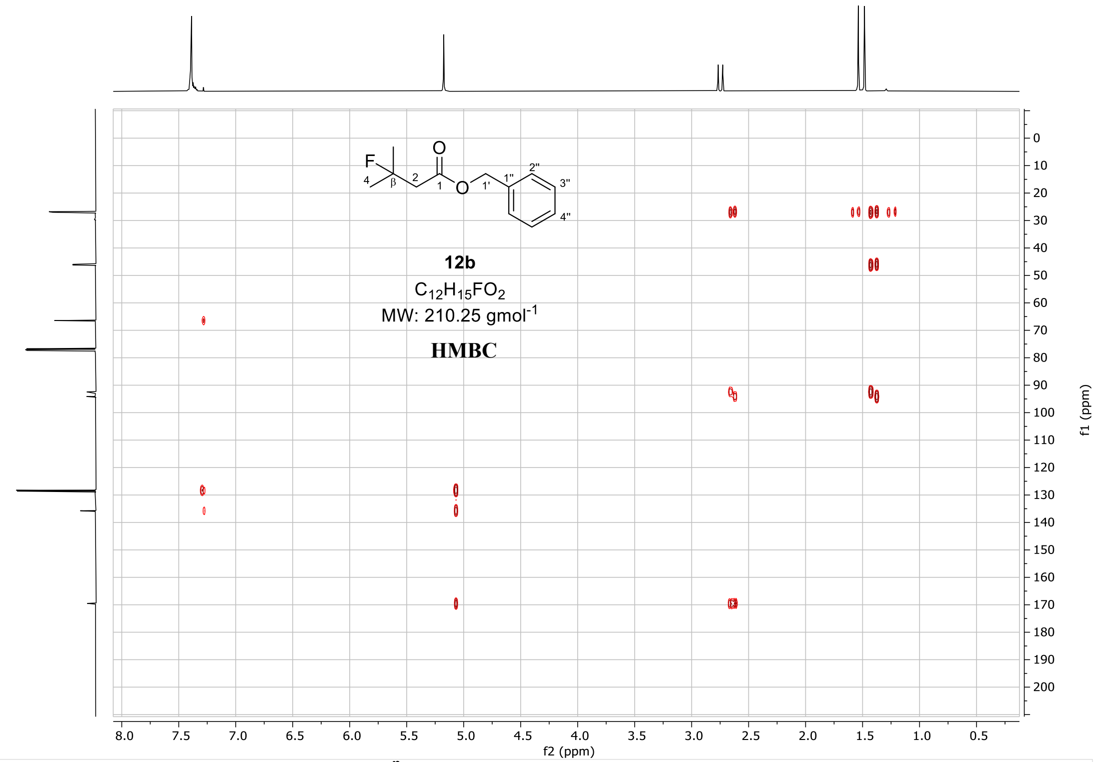

12b
$C_{12}H_{15}FO_2$
MW: 210.25 gmol-1
HMBC
f2 (ppm)
f1 (ppm)

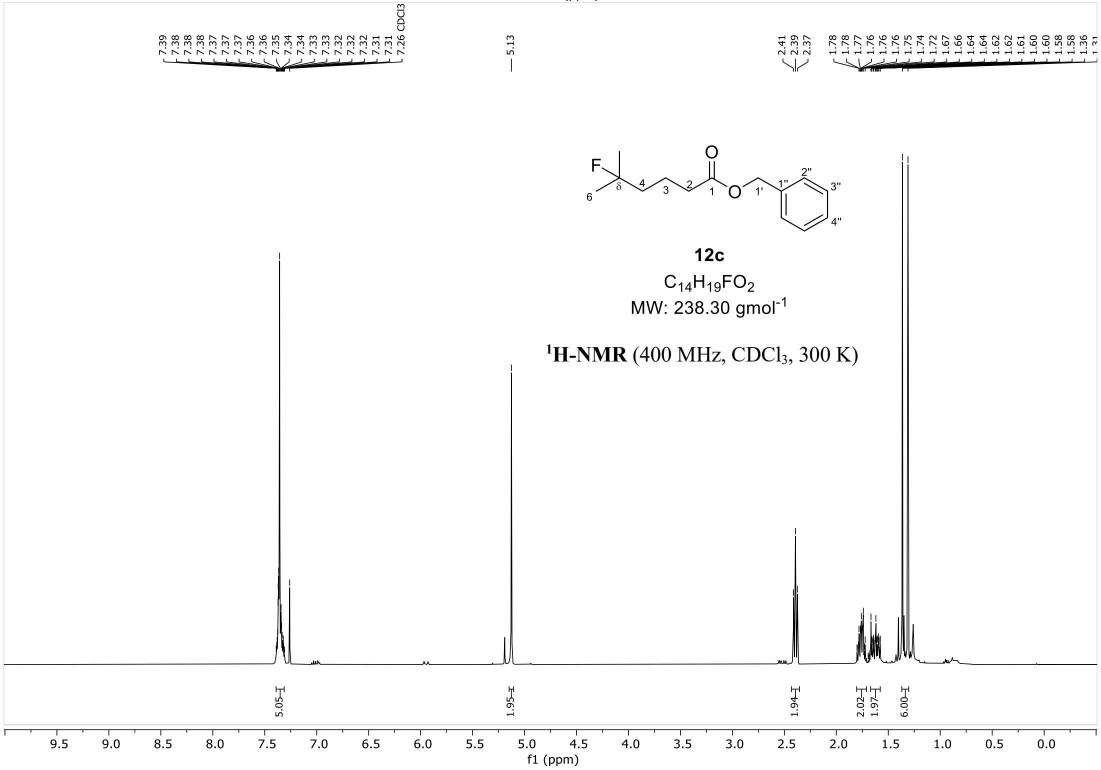

7.39 7.38 7.38 7.38 7.37 7.37 7.37 7.36 7.36 7.35 7.34 7.34 7.33 7.33 7.32 7.32 7.32 7.31 7.31 7.26 CDCl3
5.13
2.41 2.39 2.37
1.78 1.78 1.77 1.76 1.76 1.76 1.75 1.74 1.72 1.67 1.66 1.64 1.64 1.62 1.62 1.61 1.60 1.58 1.58 1.36
12c
$C_{14}H_{19}FO_2$
MW: 238.30 gmol-1
1H-NMR (400 MHz, CDCl3, 300 K)
5.05
1.95
1.94
2.02
1.97
6.00
f1 (ppm)

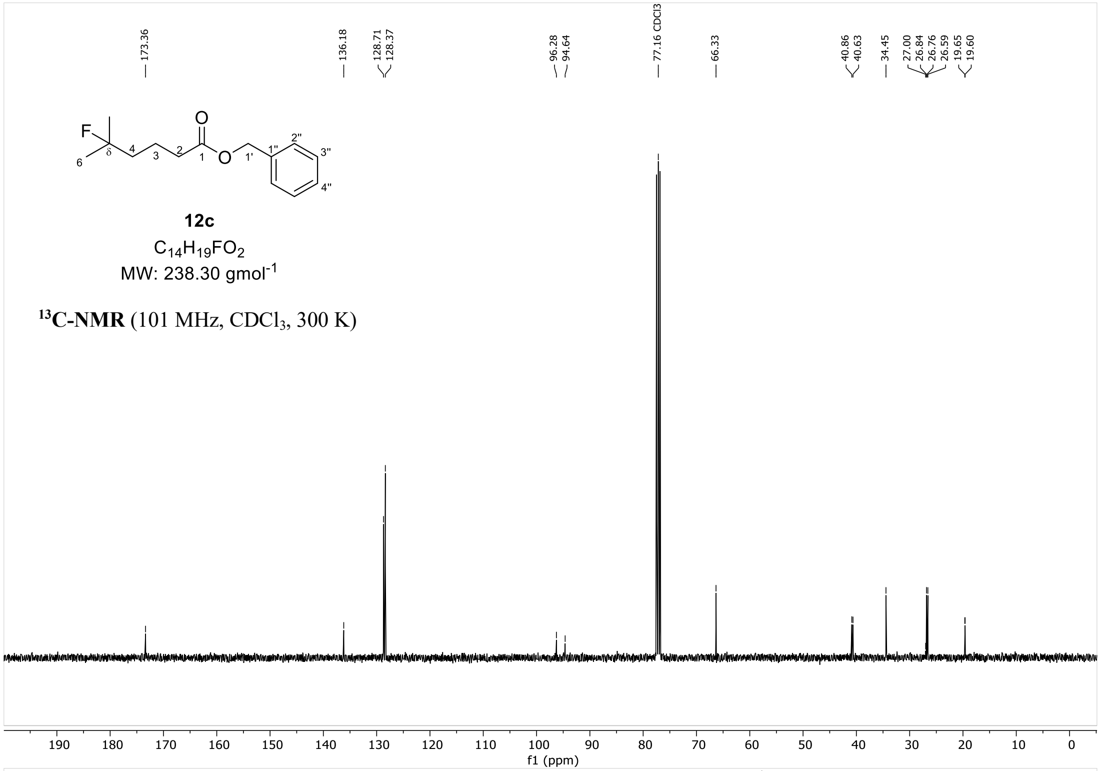

^{13}C-NMR (101 MHz, $CDCl_3$, 300 K)

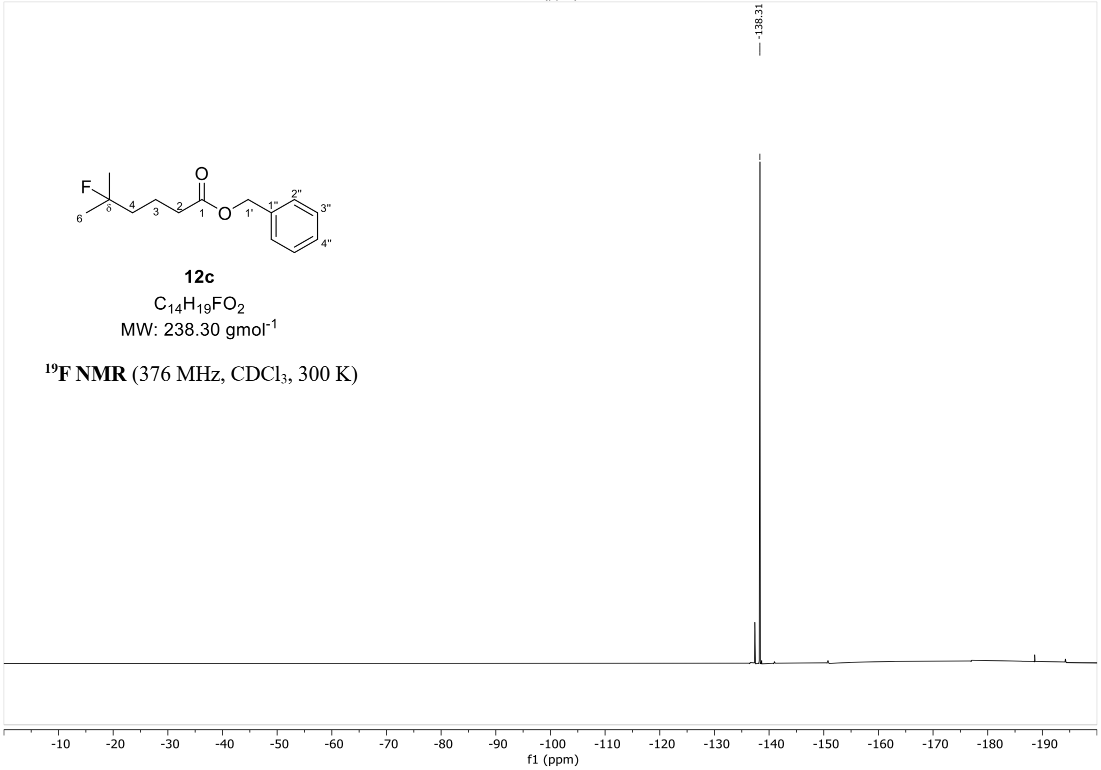

^{19}F NMR (376 MHz, $CDCl_3$, 300 K)

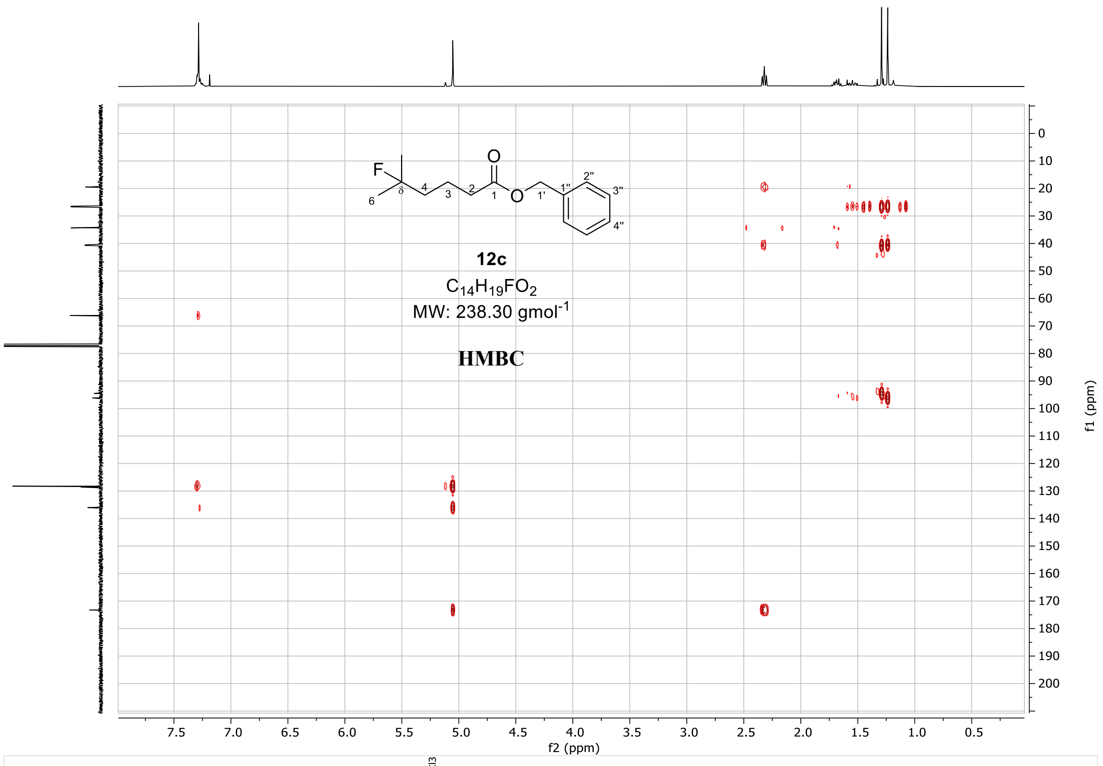
F
6
4
3
2
1
O
1'
1''
2''
3''
4''
12c
C14H19FO2
MW: 238.30 gmol-1
HMBC
f2 (ppm)
f1 (ppm)

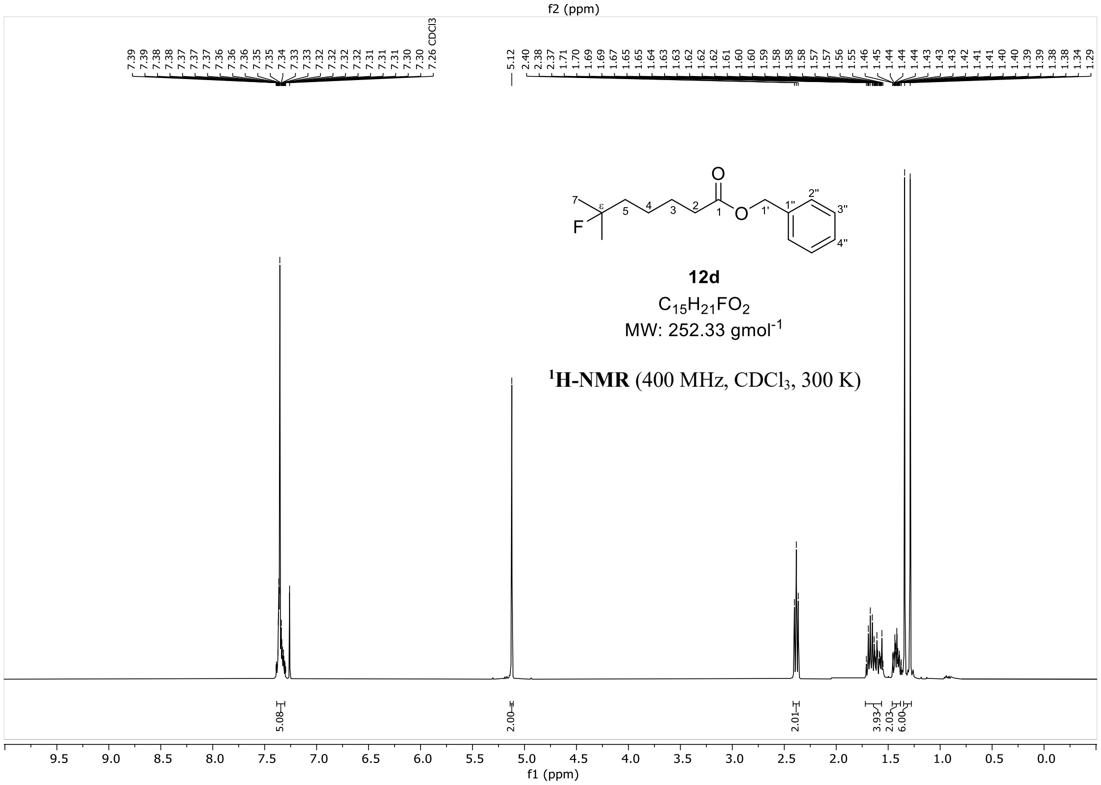
7
F
5
4
3
2
1
O
1'
1''
2''
3''
4''
12d
C15H21FO2
MW: 252.33 gmol-1
1H-NMR (400 MHz, CDCl3, 300 K)
f1 (ppm)

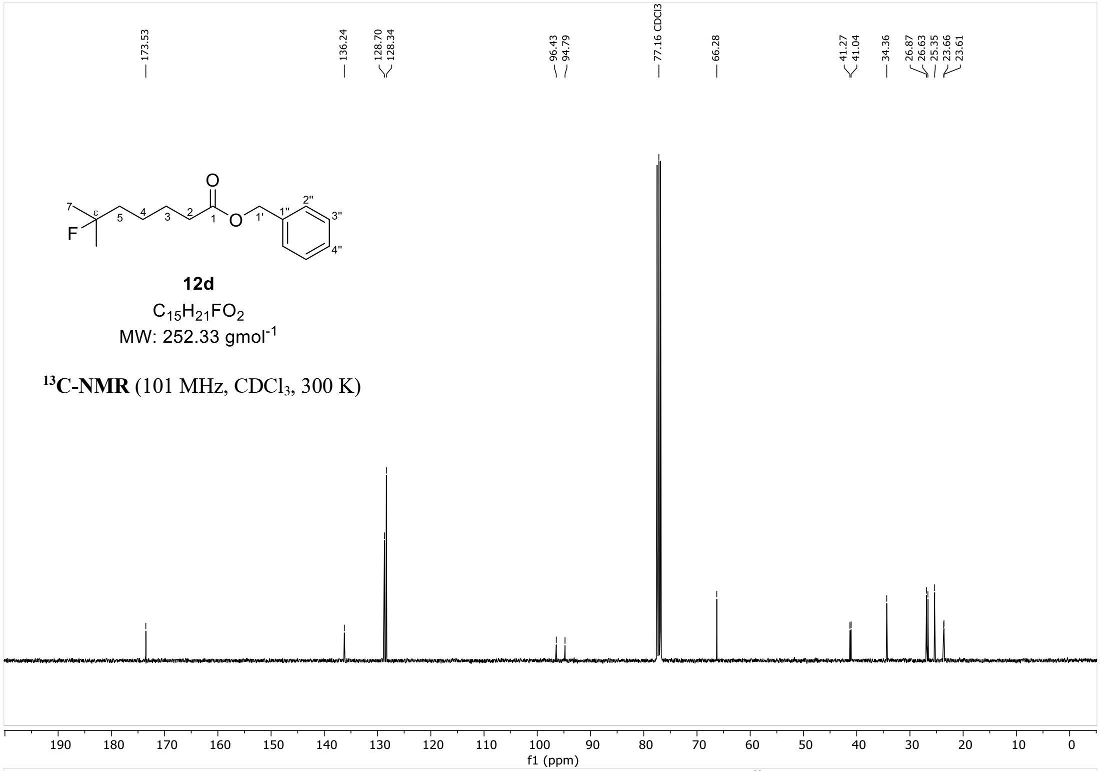
173.53
136.24
128.70
128.34
96.43
94.79
77.16 CDCl3
66.28
41.27
41.04
34.36
26.87
26.63
25.35
23.66
23.61
12d
C15H21FO2
MW: 252.33 gmol-1
13C-NMR (101 MHz, CDCl3, 300 K)
190 180 170 160 150 140 130 120 110 100 90 80 70 60 50 40 30 20 10 0
f1 (ppm)

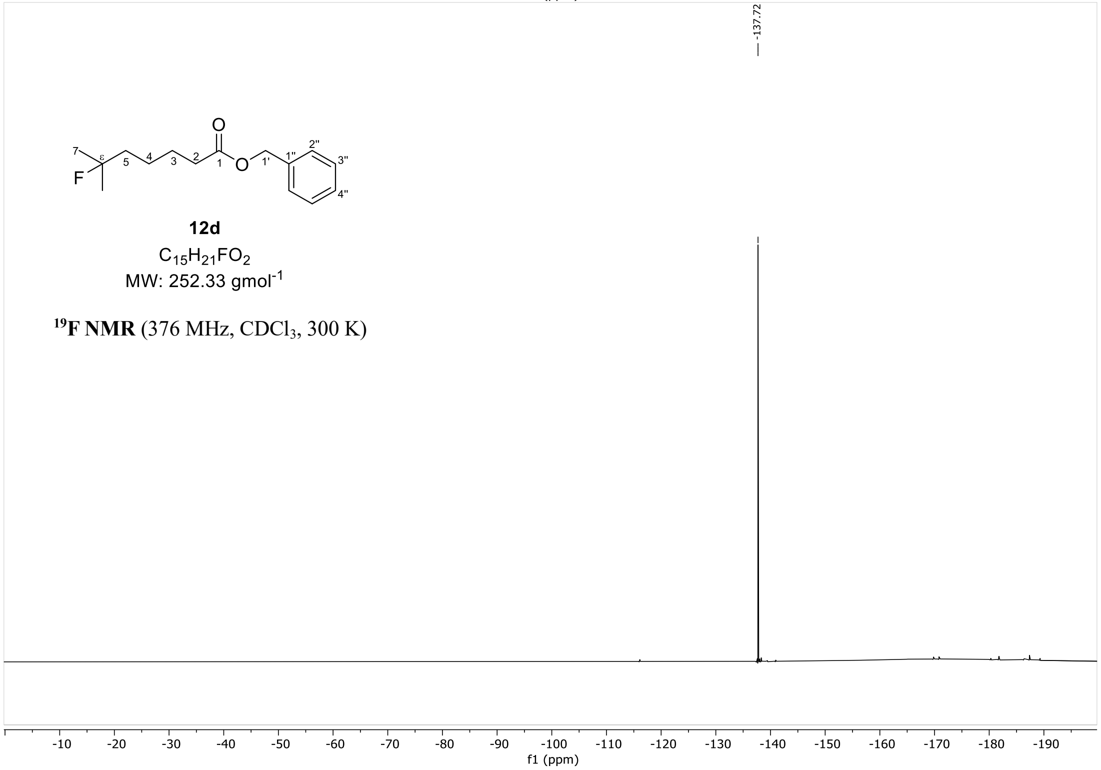
-137.72
12d
C15H21FO2
MW: 252.33 gmol-1
19F NMR (376 MHz, CDCl3, 300 K)
-10 -20 -30 -40 -50 -60 -70 -80 -90 -100 -110 -120 -130 -140 -150 -160 -170 -180 -190
f1 (ppm)

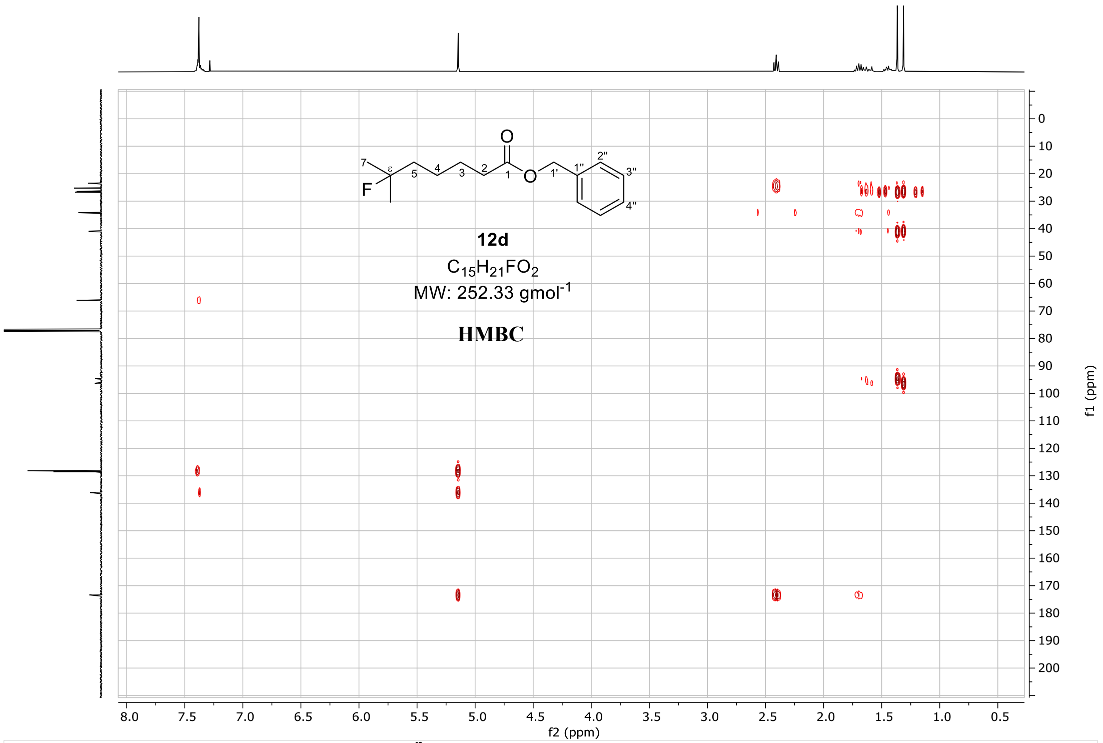
12d
$C_{15}H_{21}FO_2$
MW: 252.33 gmol^{-1}
HMBC
f1 (ppm)
f2 (ppm)

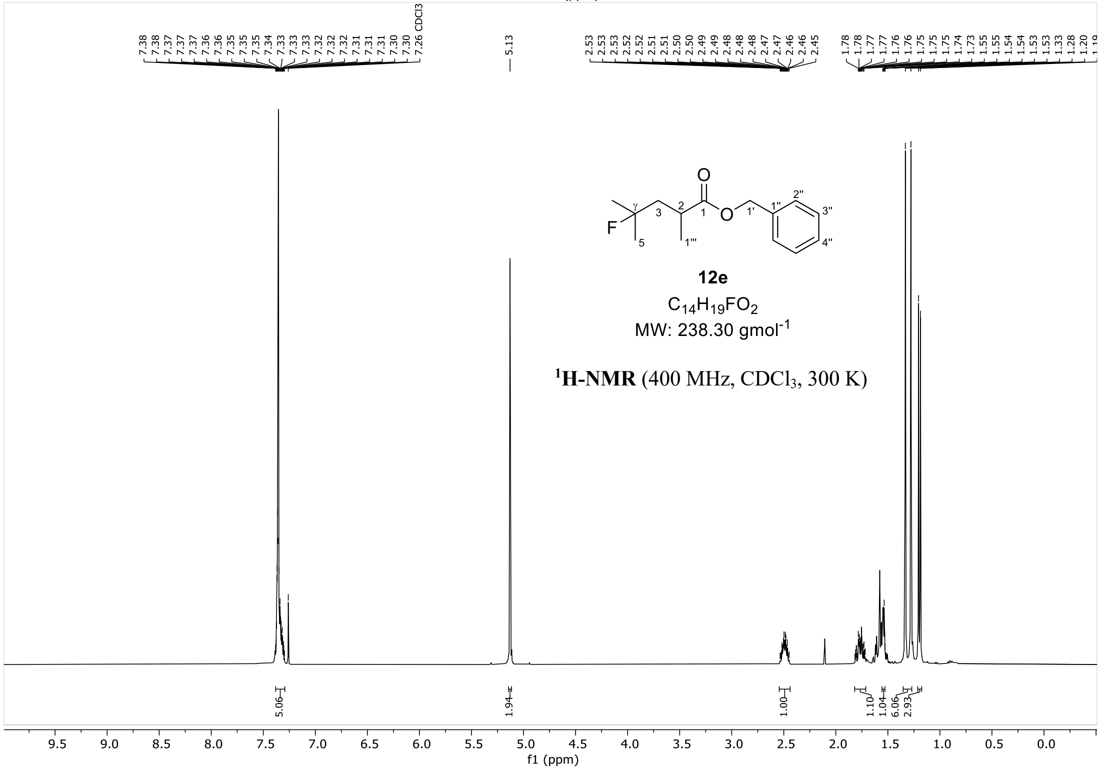
12e
$C_{14}H_{19}FO_2$
MW: 238.30 gmol^{-1}
^{1}H-NMR (400 MHz, CDCl$_3$, 300 K)
f1 (ppm)

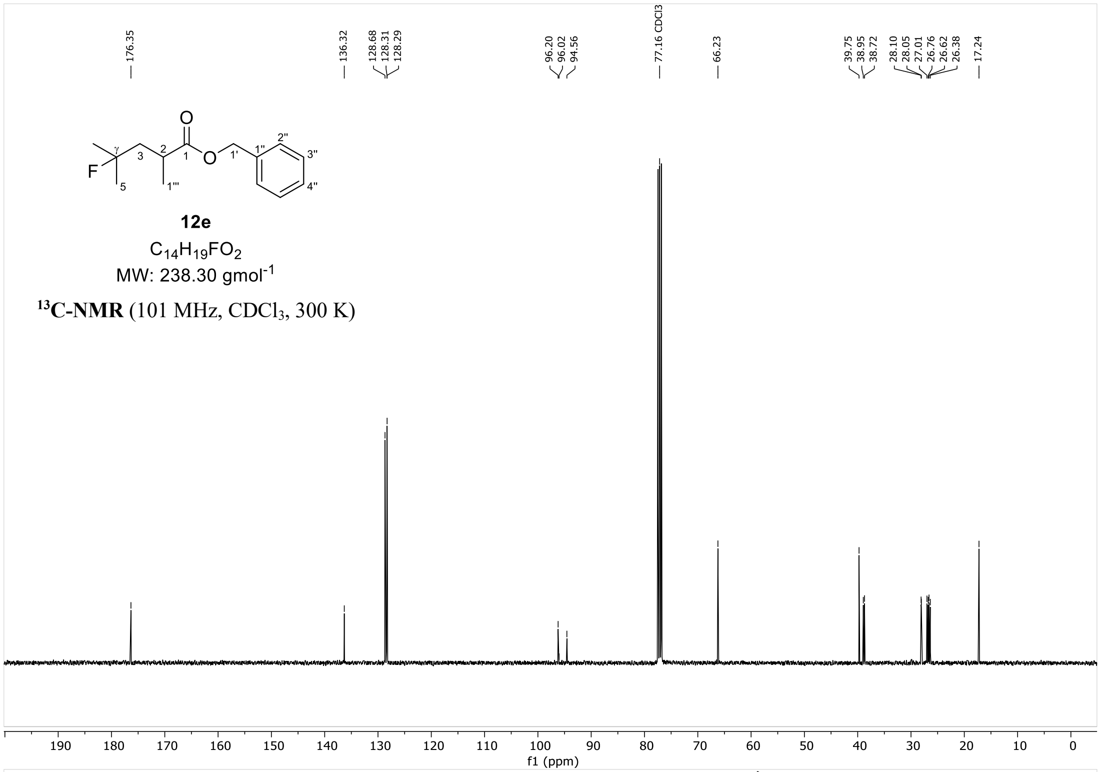
176.35
136.32
128.68
128.31
128.29
96.20
96.02
94.56
77.16 CDCl3
66.23
39.75
38.95
38.72
28.10
28.05
27.01
26.76
26.62
26.38
17.24
12e
$C_{14}H_{19}FO_2$
MW: 238.30 gmol^{-1}
^{13}C-NMR (101 MHz, CDCl$_3$, 300 K)
f1 (ppm)

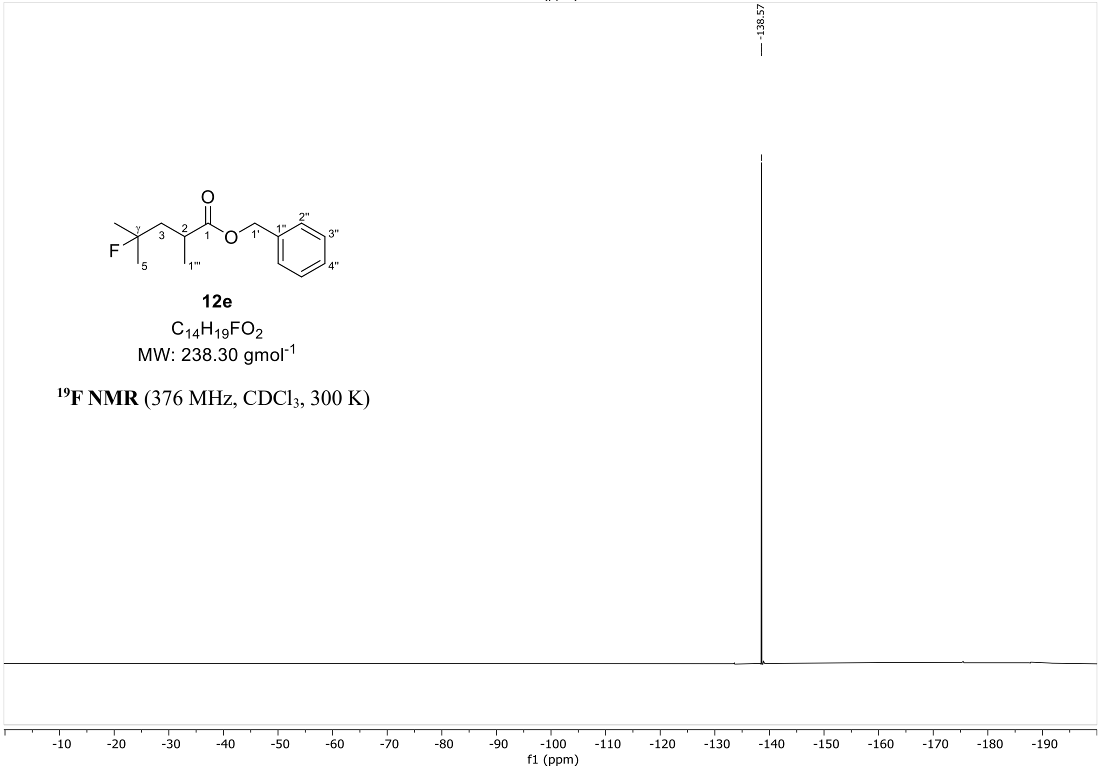
-138.57
12e
$C_{14}H_{19}FO_2$
MW: 238.30 gmol^{-1}
^{19}F NMR (376 MHz, CDCl$_3$, 300 K)
f1 (ppm)

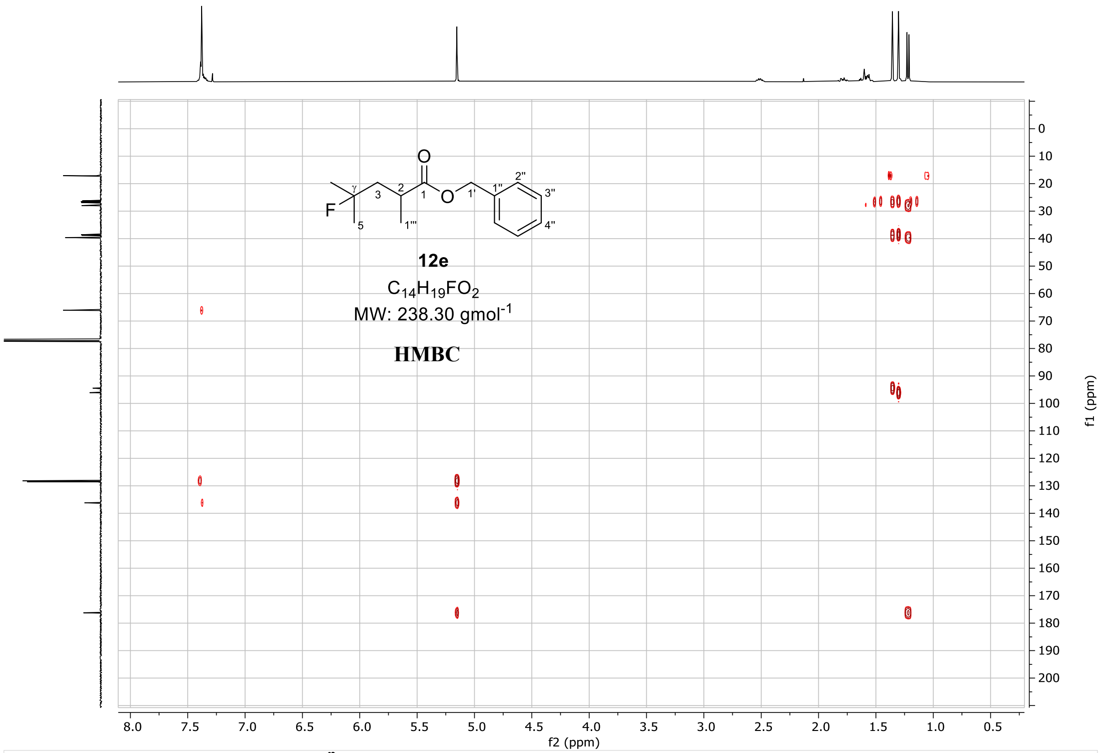
12e
$C_{14}H_{19}FO_2$
MW: 238.30 gmol^{-1}
HMBC
f1 (ppm)
f2 (ppm)

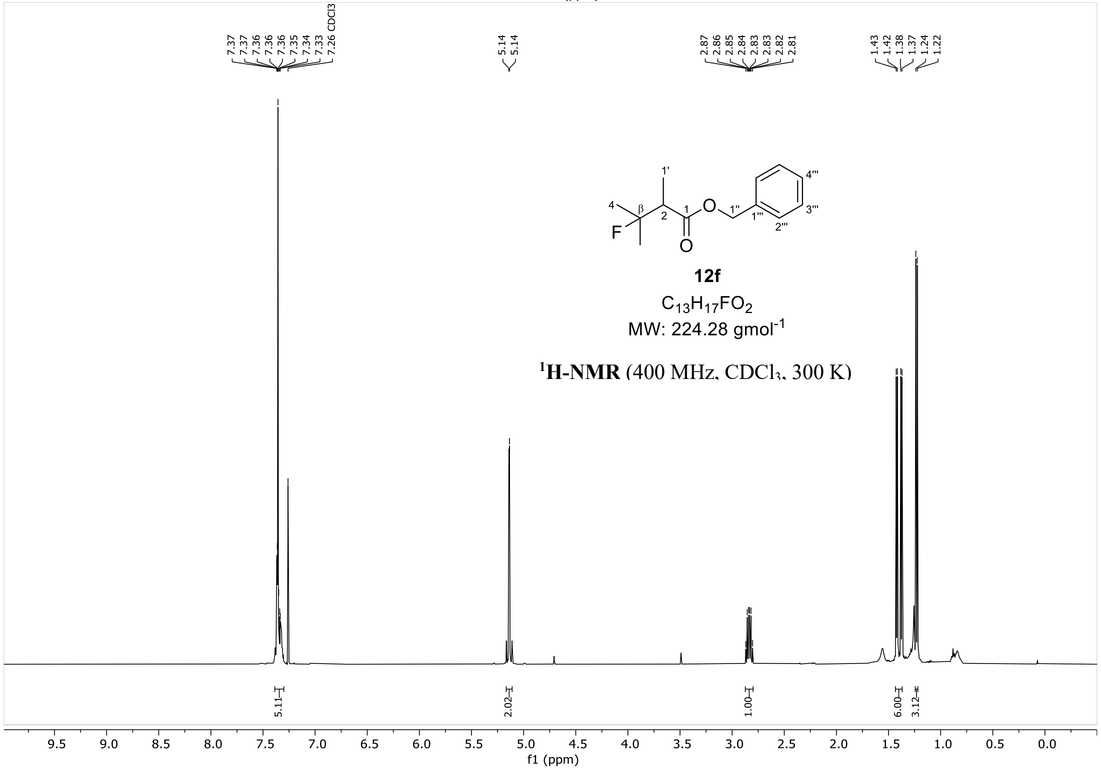
12f
$C_{13}H_{17}FO_2$
MW: 224.28 gmol^{-1}
^{1}H-NMR (400 MHz, $CDCl_3$, 300 K)
f1 (ppm)

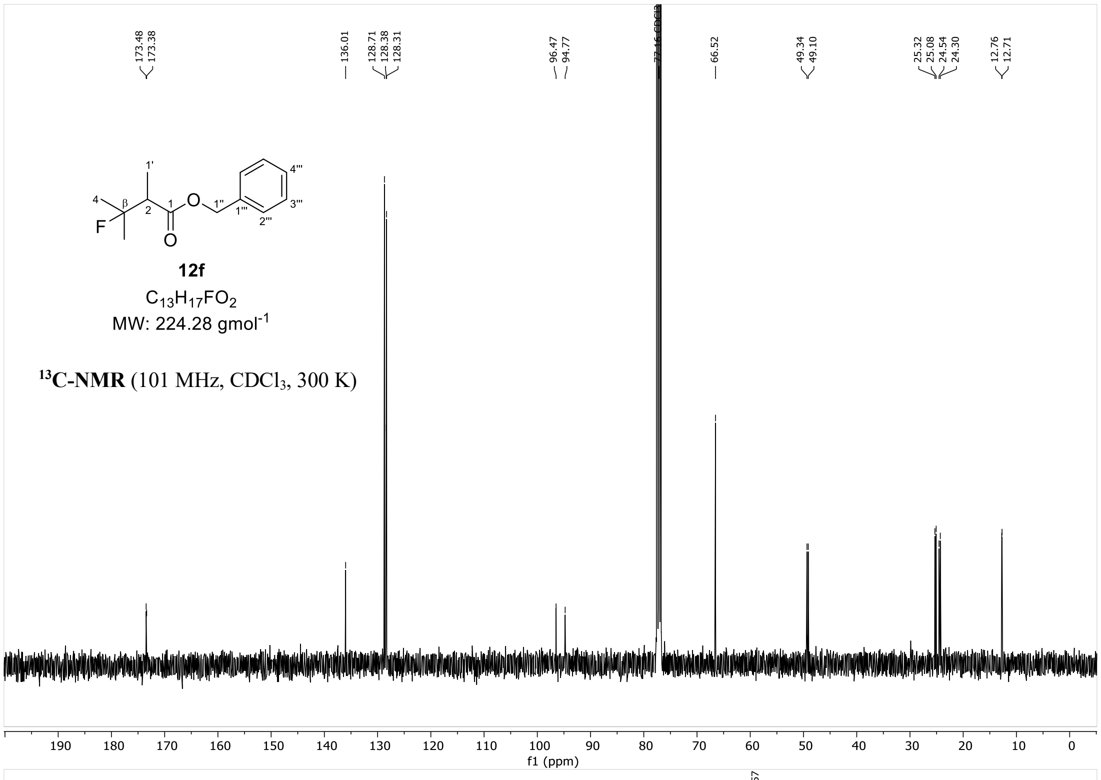
173.48
173.38
136.01
128.71
128.38
128.31
96.47
94.77
66.52
49.34
49.10
25.32
25.08
24.54
24.30
12.76
12.71
12f
$C_{13}H_{17}FO_2$
MW: 224.28 $gmol^{-1}$
^{13}C-NMR (101 MHz, $CDCl_3$, 300 K)
f1 (ppm)

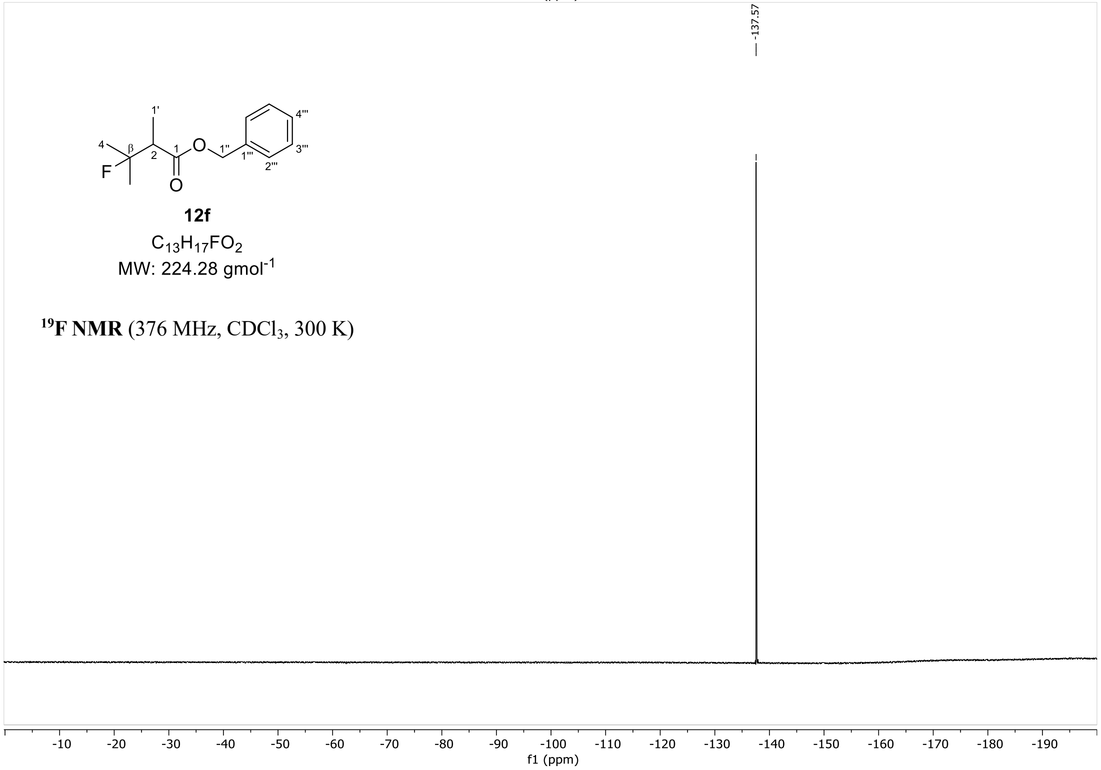
-137.57
12f
$C_{13}H_{17}FO_2$
MW: 224.28 $gmol^{-1}$
^{19}F NMR (376 MHz, $CDCl_3$, 300 K)
f1 (ppm)

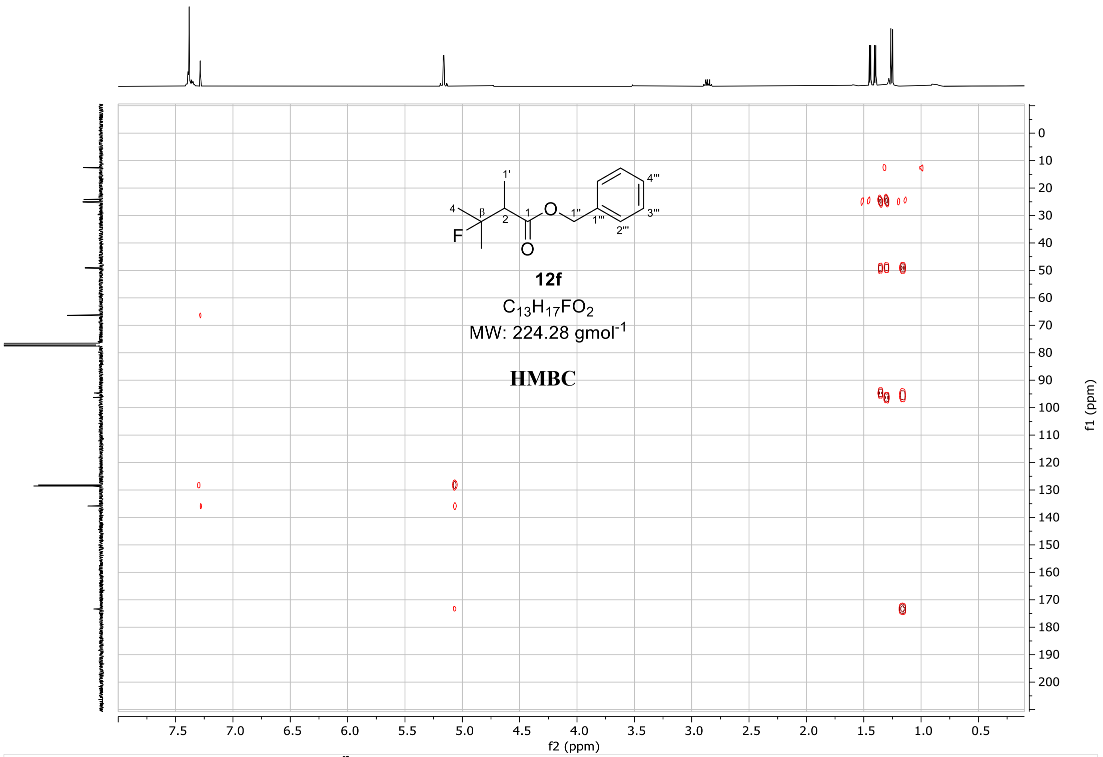
12f
$C_{13}H_{17}FO_2$
MW: 224.28 gmol^{-1}
HMBC
f2 (ppm)
f1 (ppm)

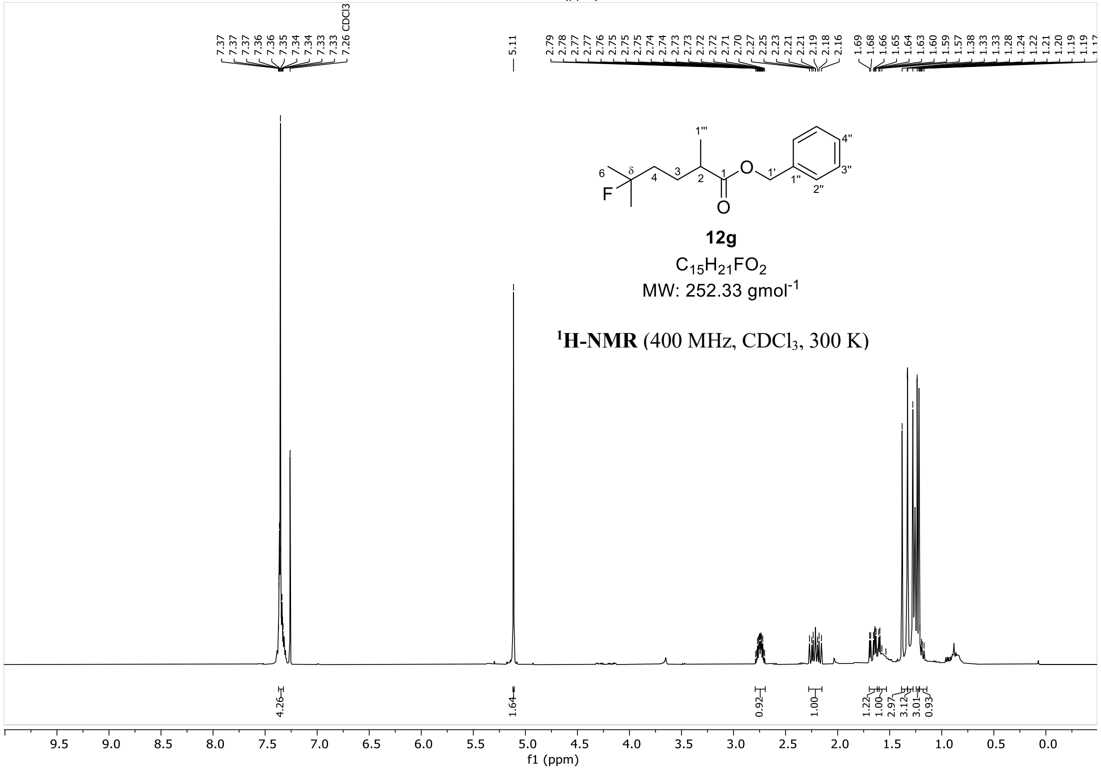
12g
$C_{15}H_{21}FO_2$
MW: 252.33 gmol^{-1}
^{1}H-NMR (400 MHz, $CDCl_3$, 300 K)
f1 (ppm)

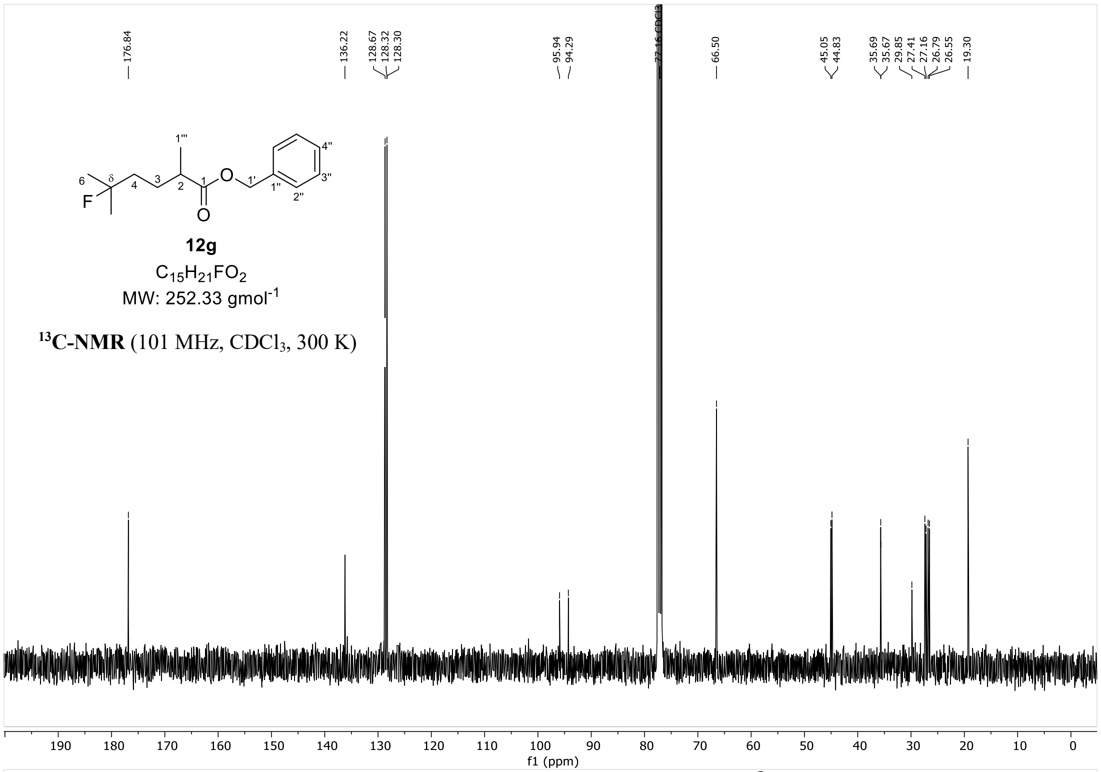
176.84
136.22
128.67
128.32
128.30
95.94
94.29
77.16 CDCl3
66.50
45.05
44.83
35.69
35.67
29.85
27.41
27.16
26.79
26.55
19.30
12g
$C_{15}H_{21}FO_2$
MW: 252.33 gmol^{-1}
^{13}C-NMR (101 MHz, $CDCl_3$, 300 K)
f1 (ppm)

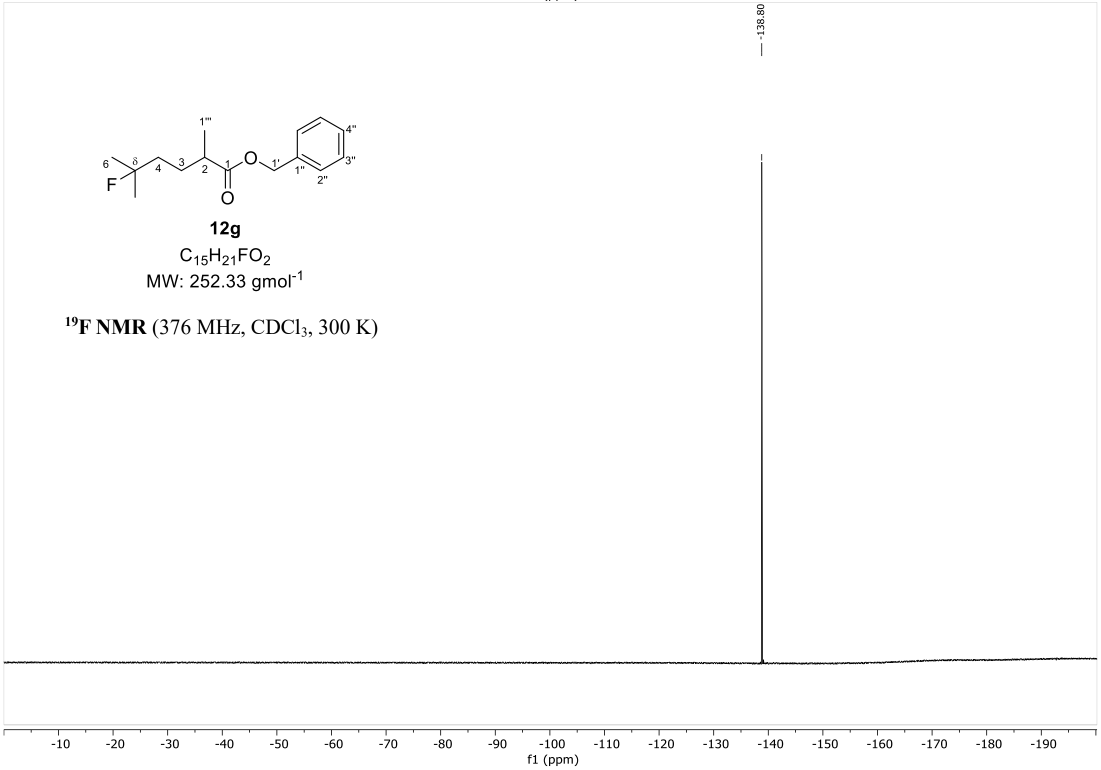
-138.80
12g
$C_{15}H_{21}FO_2$
MW: 252.33 gmol^{-1}
^{19}F NMR (376 MHz, $CDCl_3$, 300 K)
f1 (ppm)

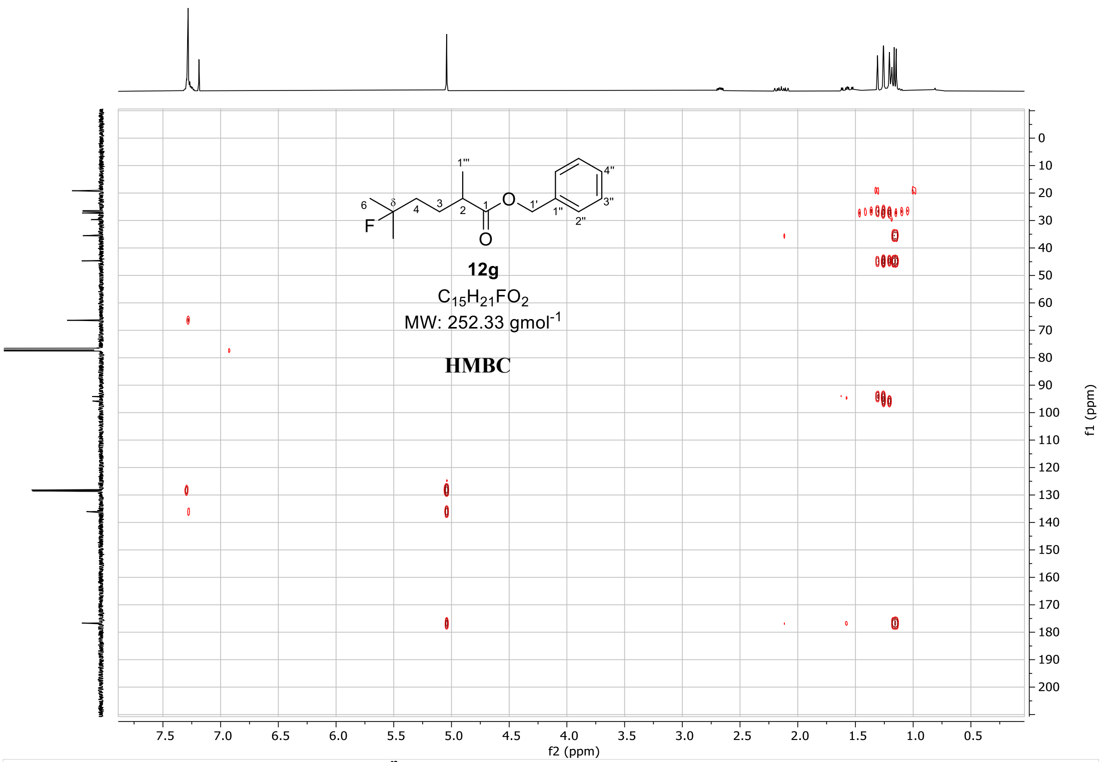

12g
$C_{15}H_{21}FO_2$
MW: 252.33 gmol-1
HMBC
f2 (ppm)
f1 (ppm)

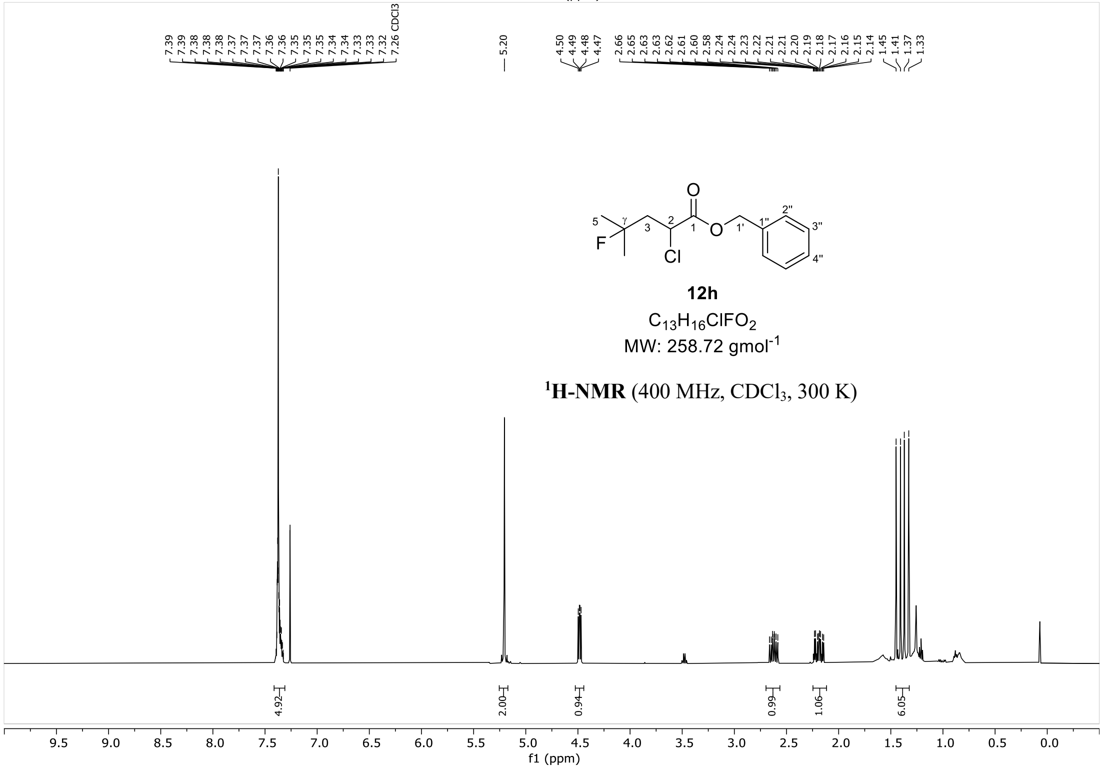

12h
$C_{13}H_{16}ClFO_2$
MW: 258.72 gmol-1
1H-NMR (400 MHz, CDCl3, 300 K)
f1 (ppm)

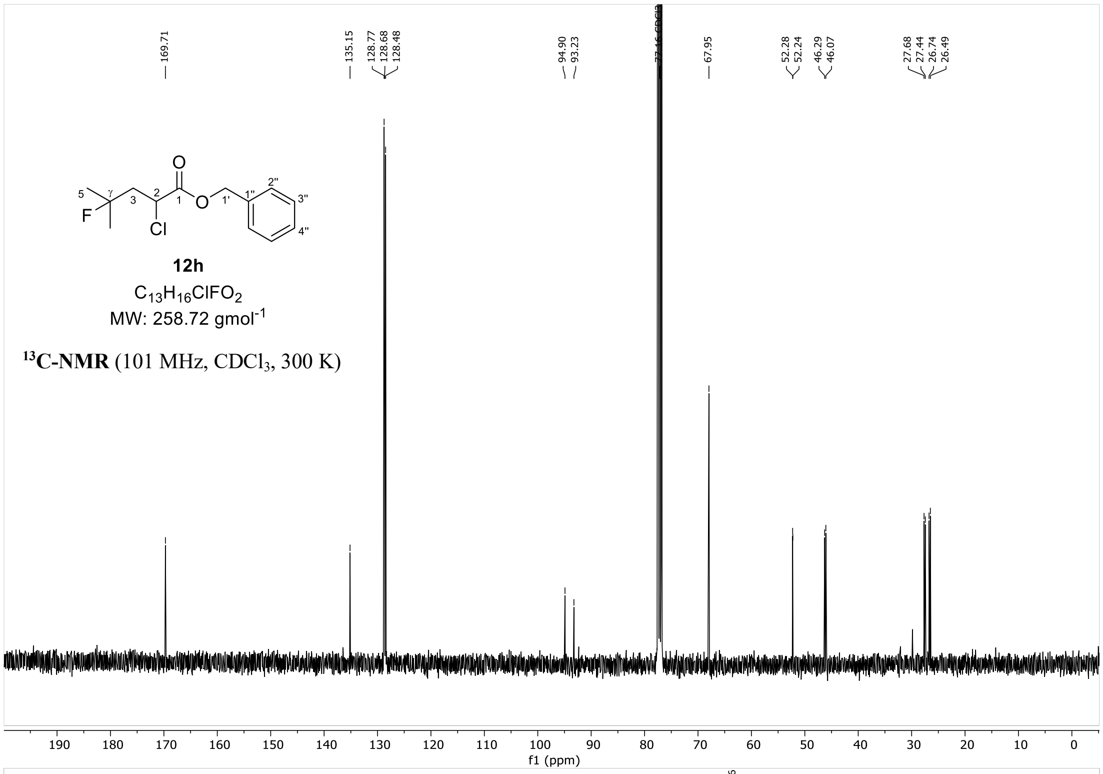
169.71
135.15
128.77
128.68
128.48
94.90
93.23
67.95
52.28
52.24
46.29
46.07
27.68
27.44
26.74
26.49
12h
C13H16ClFO2
MW: 258.72 gmol-1
13C-NMR (101 MHz, CDCl3, 300 K)
f1 (ppm)

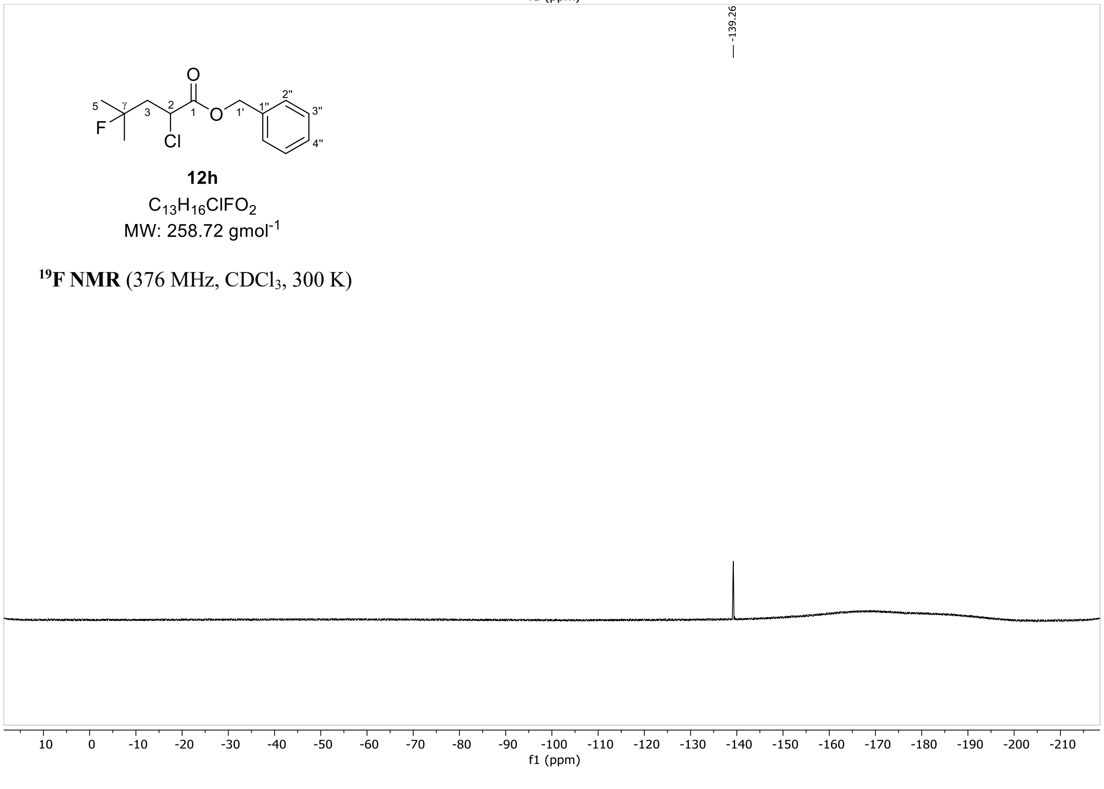
-139.26
12h
C13H16ClFO2
MW: 258.72 gmol-1
19F NMR (376 MHz, CDCl3, 300 K)
f1 (ppm)

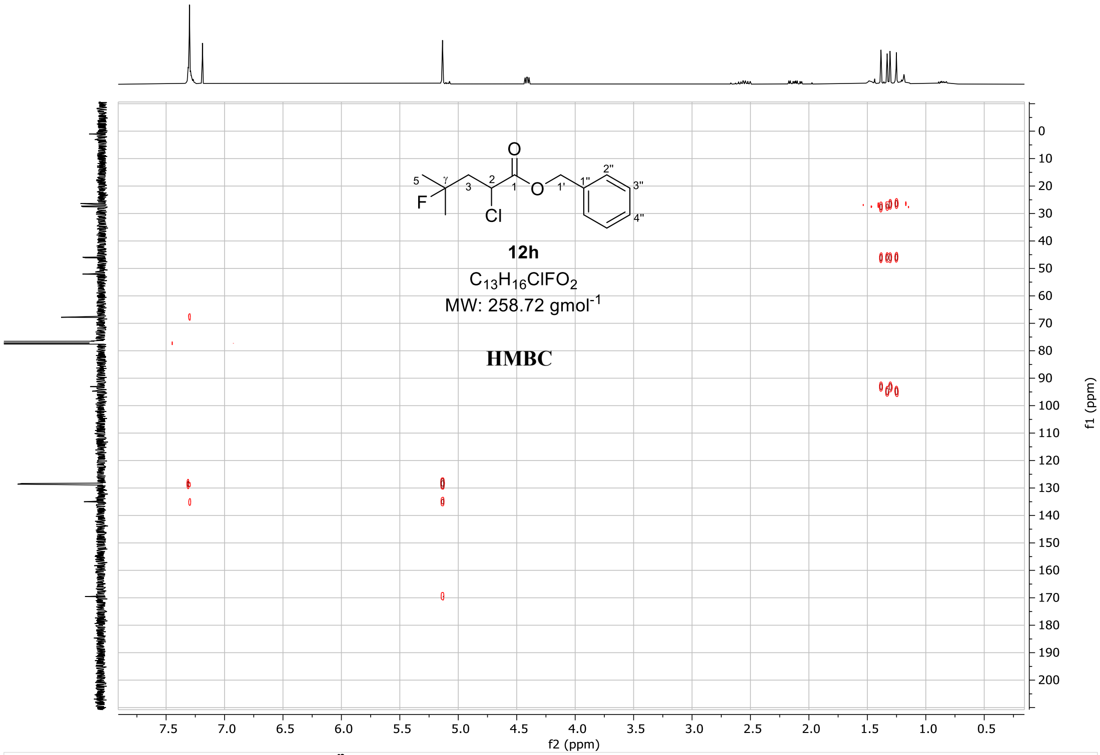
12h
$C_{13}H_{16}ClFO_2$
MW: 258.72 gmol[-1]
HMBC
f2 (ppm)
f1 (ppm)

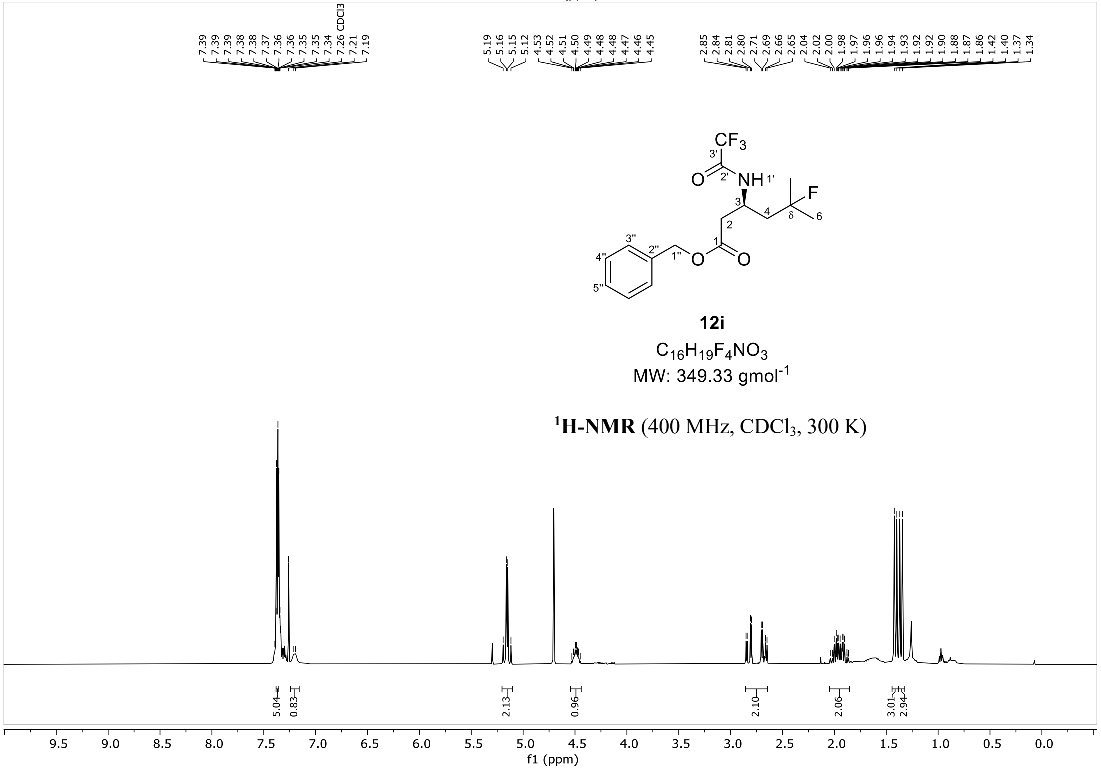
7.39 7.39 7.39 7.38 7.37 7.36 7.36 7.35 7.35 7.34 7.26 CDCl3 7.21 7.19
5.19 5.16 5.15 5.12 4.53 4.52 4.51 4.50 4.49 4.48 4.47 4.46 4.45
2.85 2.84 2.81 2.80 2.71 2.69 2.66 2.65 2.04 2.02 2.00 1.98 1.97 1.96 1.94 1.93 1.92 1.92 1.90 1.88 1.87 1.86 1.42 1.40 1.37 1.34
12i
$C_{16}H_{19}F_4NO_3$
MW: 349.33 gmol[-1]
1H-NMR (400 MHz, CDCl3, 300 K)
5.04 0.83 2.13 0.96 2.10 2.06 3.01 2.94
f1 (ppm)

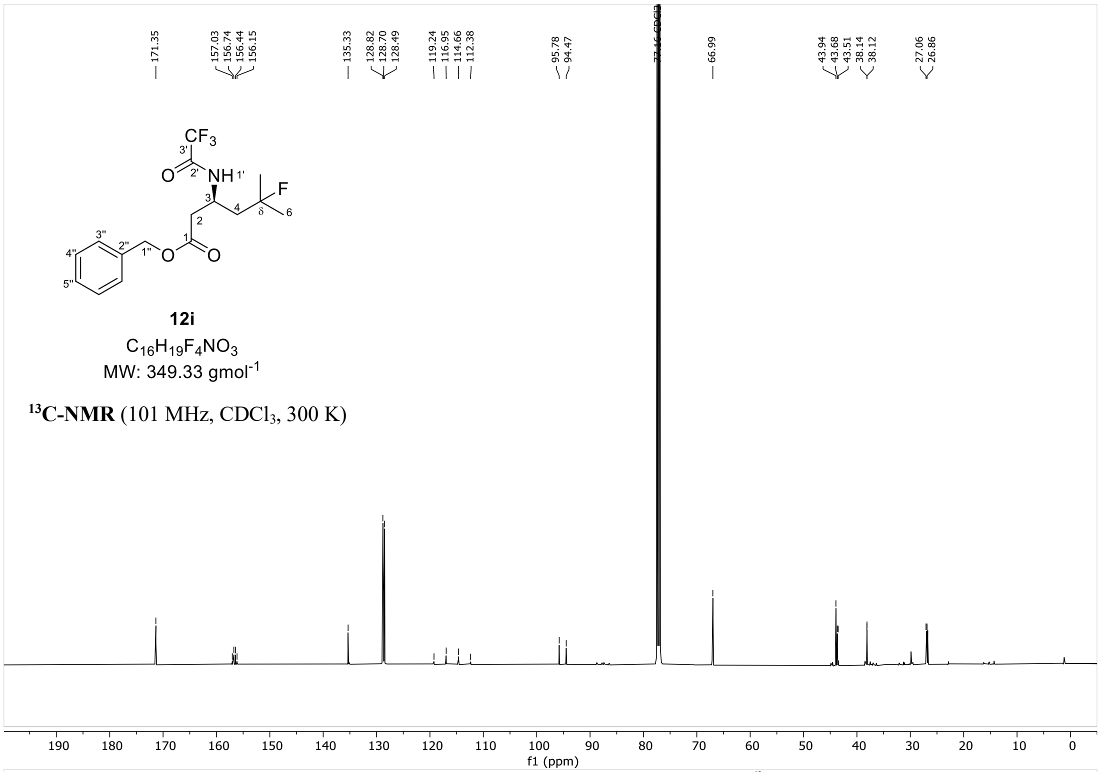
171.35
157.03
156.74
156.44
156.15
135.33
128.82
128.70
128.49
119.24
116.95
114.66
112.38
95.78
94.47
66.99
43.94
43.68
43.51
38.14
38.12
27.06
26.86
CF3
NH
F
O
O
O
12i
C16H19F4NO3
MW: 349.33 gmol-1
13C-NMR (101 MHz, CDCl3, 300 K)
f1 (ppm)

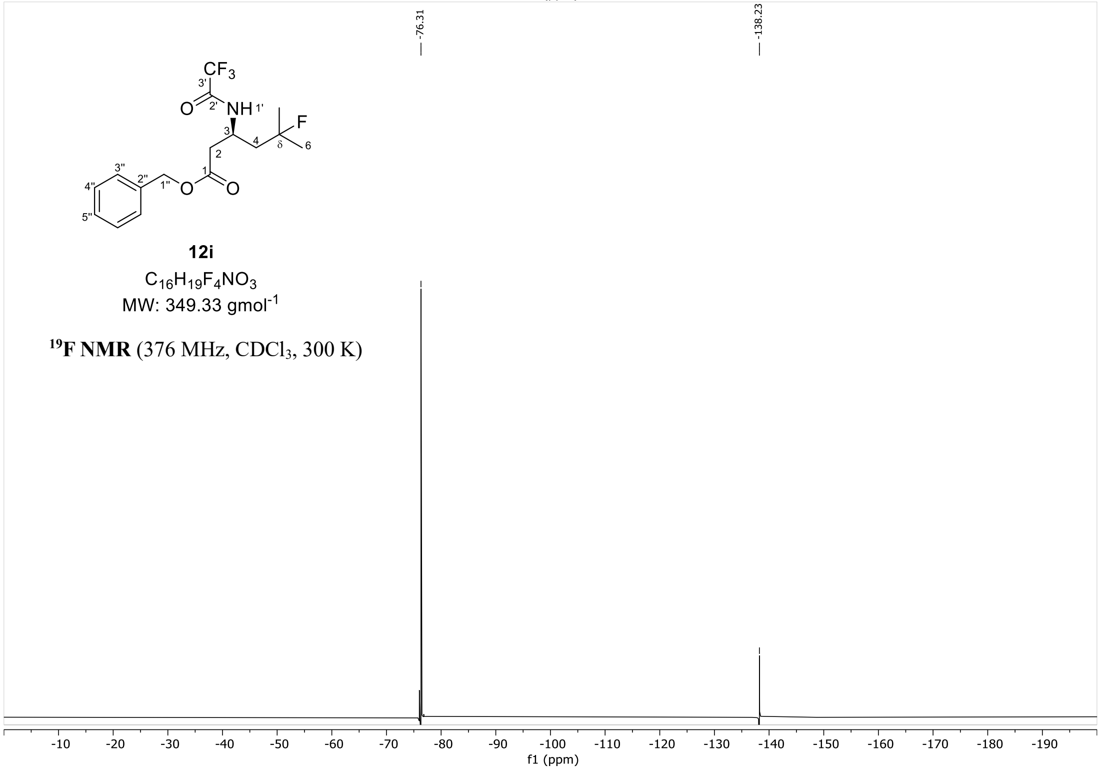
-76.31
-138.23
CF3
NH
F
O
O
O
12i
C16H19F4NO3
MW: 349.33 gmol-1
19F NMR (376 MHz, CDCl3, 300 K)
f1 (ppm)

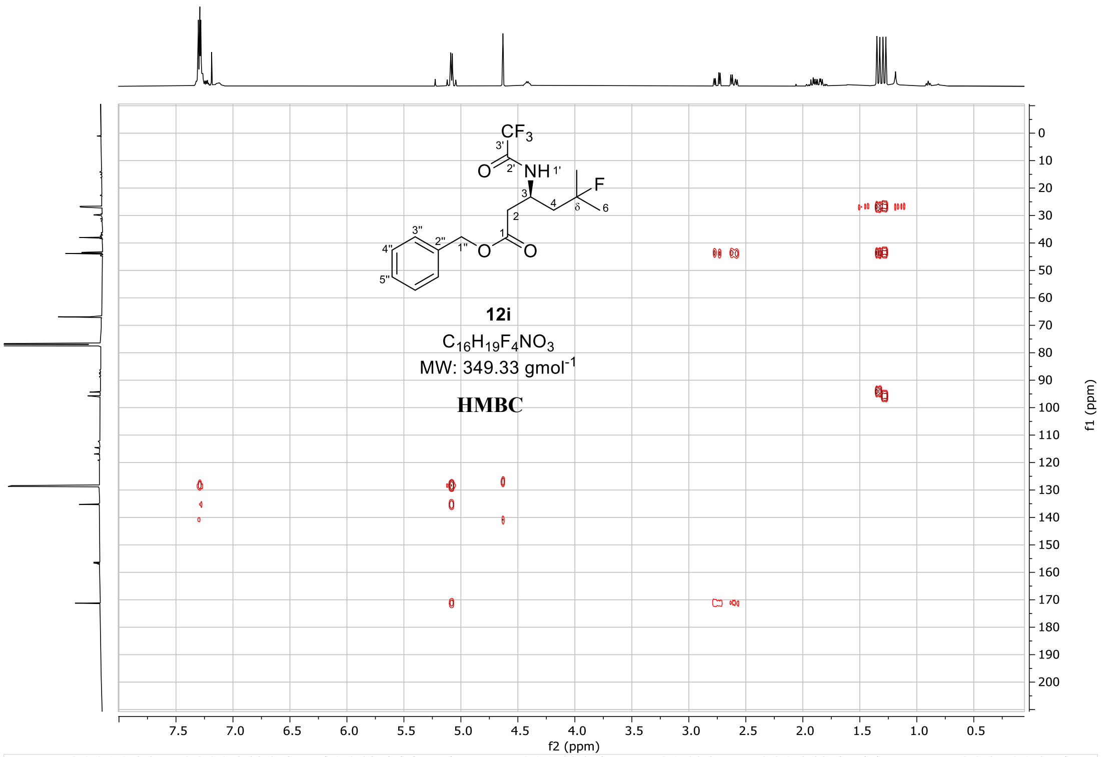
12i
C16H19F4NO3
MW: 349.33 gmol-1
HMBC
f2 (ppm)
f1 (ppm)

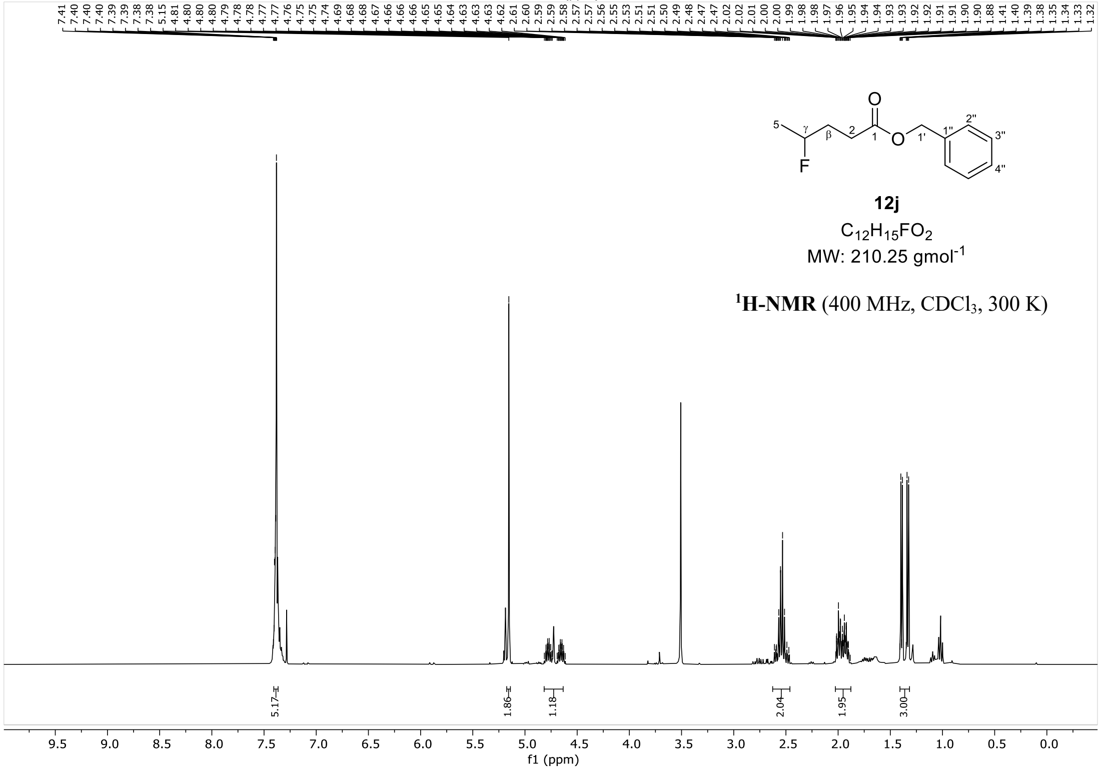
12j
C12H15FO2
MW: 210.25 gmol-1
1H-NMR (400 MHz, CDCl3, 300 K)
f1 (ppm)

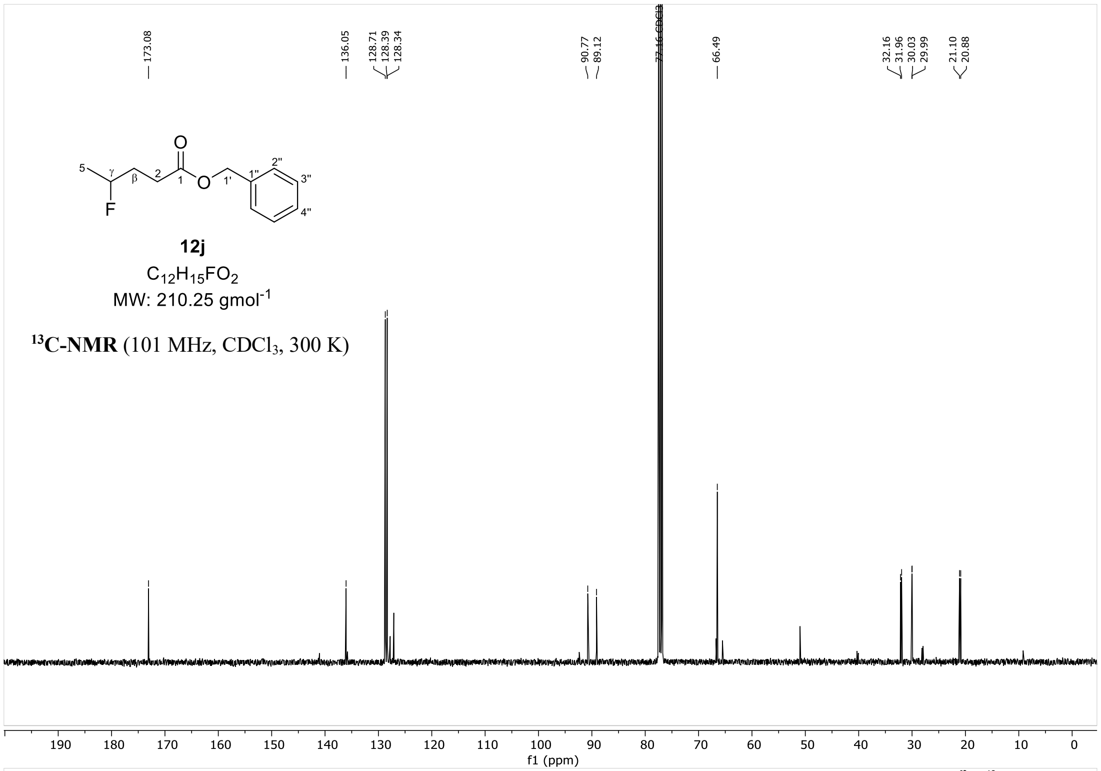
173.08
136.05
128.71
128.39
128.34
90.77
89.12
77.16 CDCl3
66.49
32.16
31.96
30.03
29.99
21.10
20.88
5
γ
β
2
1
O
O
1'
1''
2''
3''
4''
F
12j
$C_{12}H_{15}FO_2$
MW: 210.25 gmol^{-1}
^{13}C-NMR (101 MHz, $CDCl_3$, 300 K)
190 180 170 160 150 140 130 120 110 100 90 80 70 60 50 40 30 20 10 0
f1 (ppm)

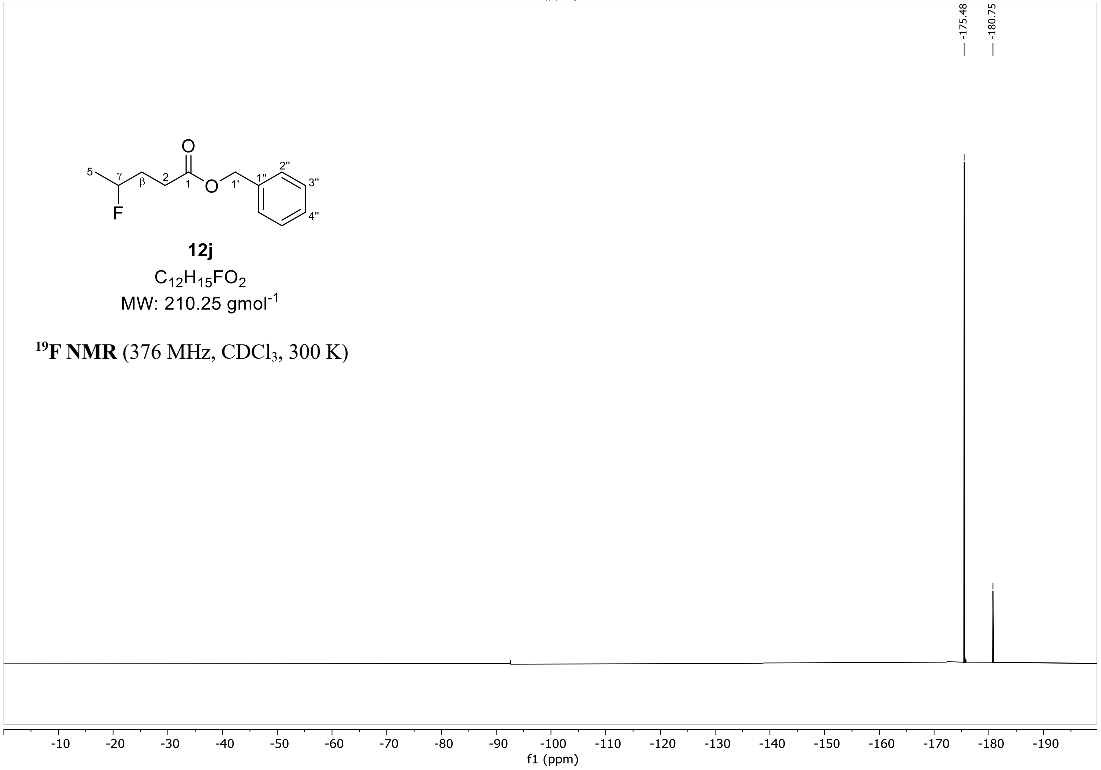
-175.48
-180.75
5
γ
β
2
1
O
O
1'
1''
2''
3''
4''
F
12j
$C_{12}H_{15}FO_2$
MW: 210.25 gmol^{-1}
^{19}F NMR (376 MHz, $CDCl_3$, 300 K)
-10 -20 -30 -40 -50 -60 -70 -80 -90 -100 -110 -120 -130 -140 -150 -160 -170 -180 -190
f1 (ppm)

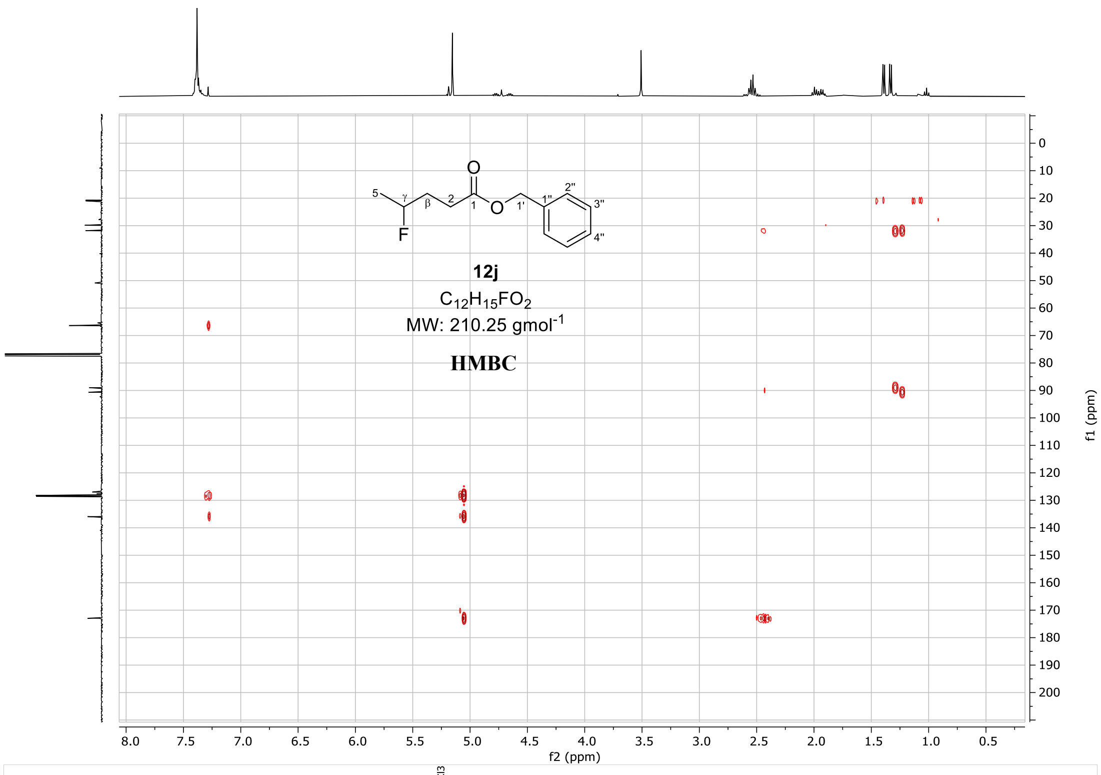
12j
$C_{12}H_{15}FO_2$
MW: 210.25 gmol^{-1}
HMBC
f2 (ppm)
f1 (ppm)

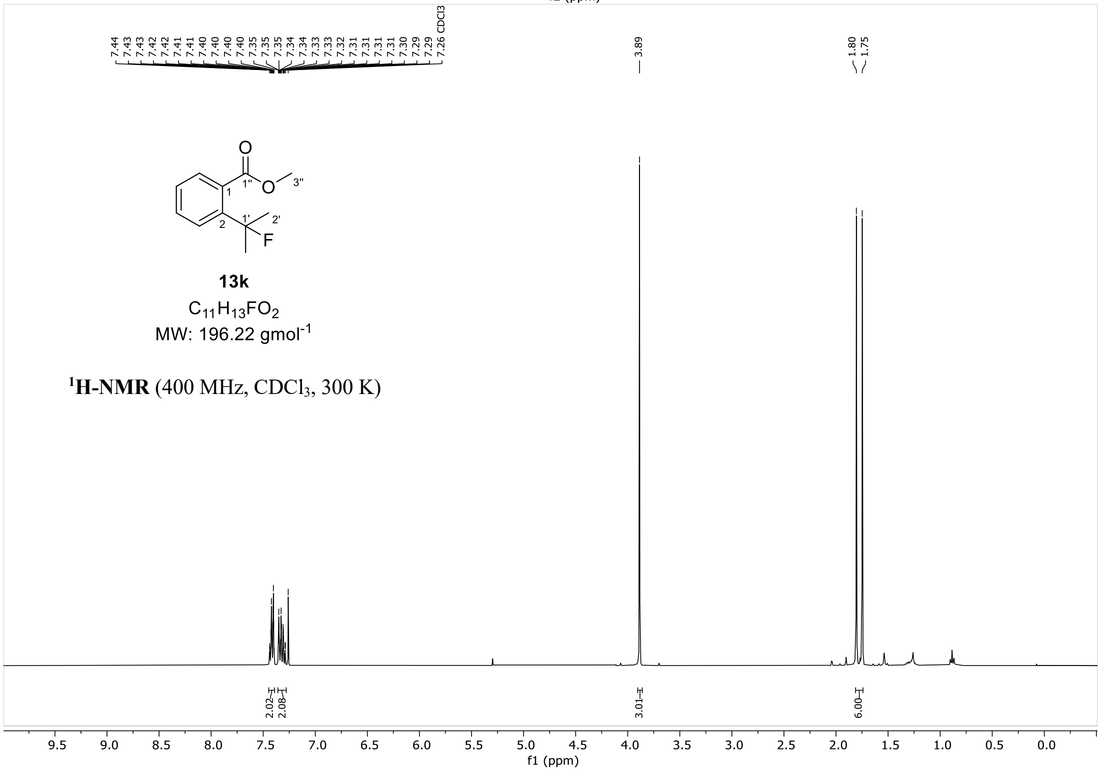
7.44
7.43
7.43
7.42
7.42
7.41
7.41
7.40
7.40
7.40
7.40
7.35
7.35
7.35
7.34
7.34
7.33
7.33
7.32
7.31
7.31
7.31
7.31
7.30
7.29
7.29
7.26 CDCl3
3.89
1.80
1.75
13k
$C_{11}H_{13}FO_2$
MW: 196.22 gmol^{-1}
^{1}H-NMR (400 MHz, $CDCl_3$, 300 K)
2.02
2.08
3.01
6.00
f1 (ppm)

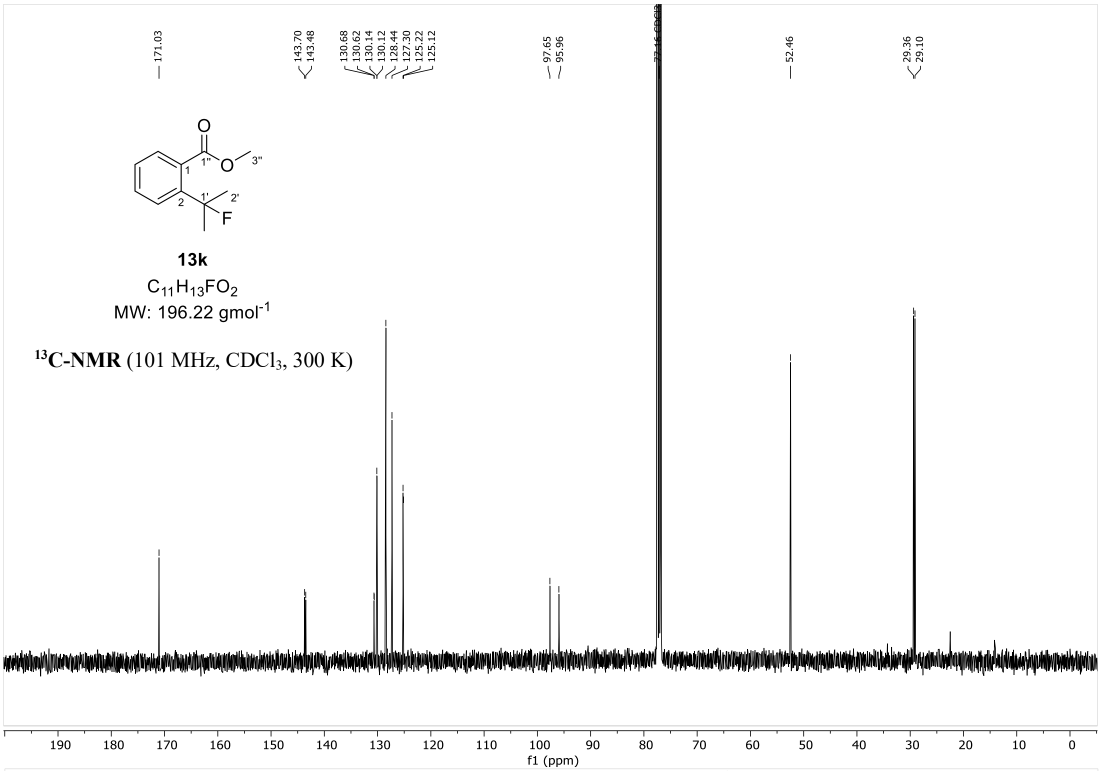
171.03
143.70
143.48
130.68
130.62
130.14
130.12
128.44
127.30
125.22
125.12
97.65
95.96
52.46
29.36
29.10
13k
C11H13FO2
MW: 196.22 gmol-1
13C-NMR (101 MHz, CDCl3, 300 K)
190 180 170 160 150 140 130 120 110 100 90 80 70 60 50 40 30 20 10 0
f1 (ppm)

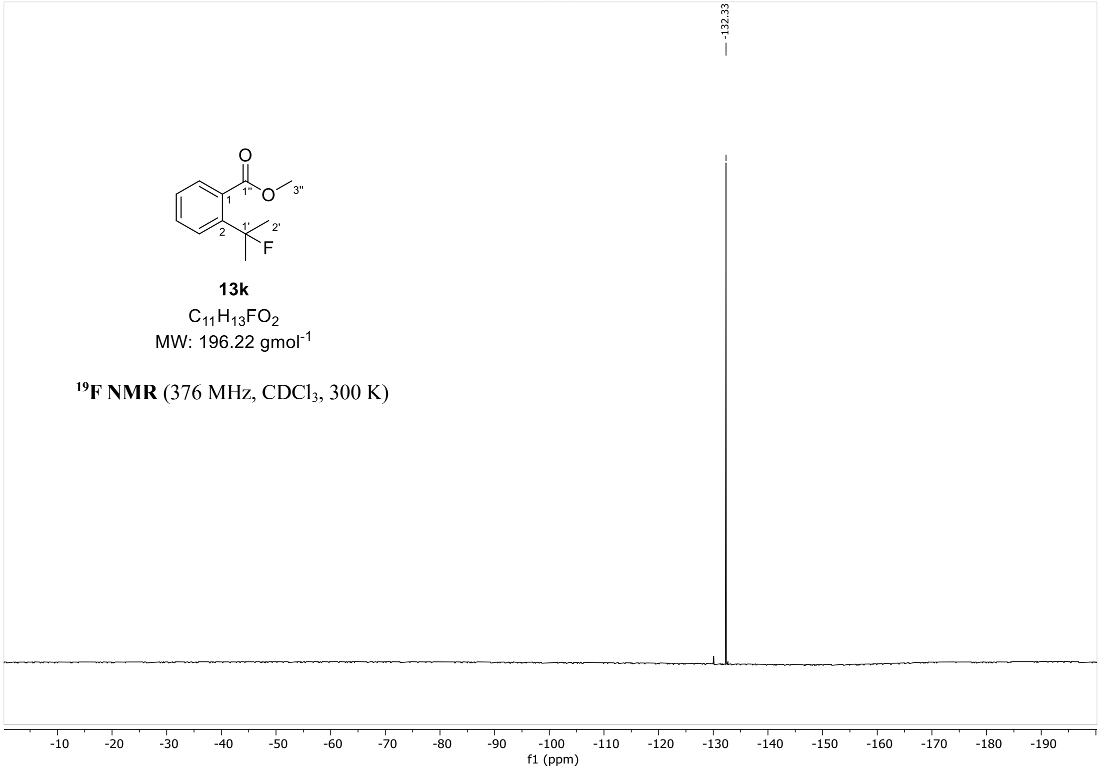
-132.33
13k
C11H13FO2
MW: 196.22 gmol-1
19F NMR (376 MHz, CDCl3, 300 K)
-10 -20 -30 -40 -50 -60 -70 -80 -90 -100 -110 -120 -130 -140 -150 -160 -170 -180 -190
f1 (ppm)

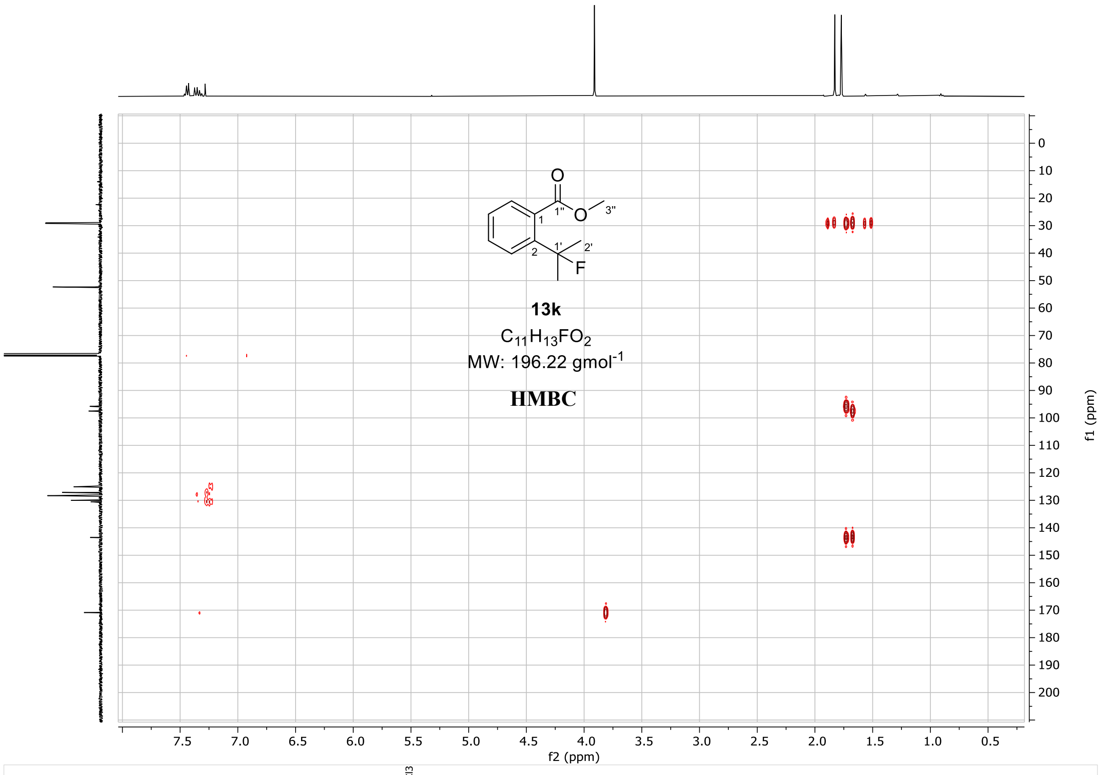

13k
C11H13FO2
MW: 196.22 gmol-1
HMBC
f2 (ppm)
f1 (ppm)

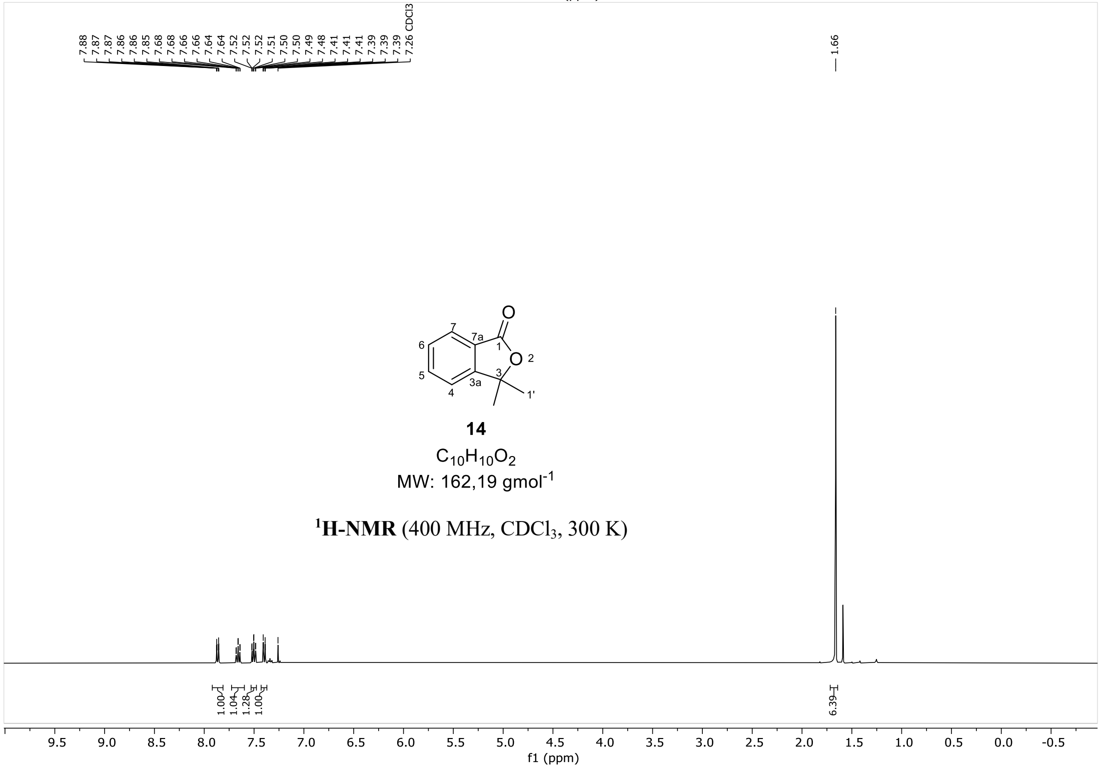

7.88
7.87
7.87
7.86
7.86
7.85
7.68
7.68
7.66
7.66
7.64
7.64
7.52
7.52
7.52
7.52
7.51
7.50
7.49
7.48
7.41
7.41
7.39
7.39
7.26 CDCl3
1.66
14
C10H10O2
MW: 162,19 gmol-1
1H-NMR (400 MHz, CDCl3, 300 K)
1.00
1.04
1.28
1.00
6.39
f1 (ppm)

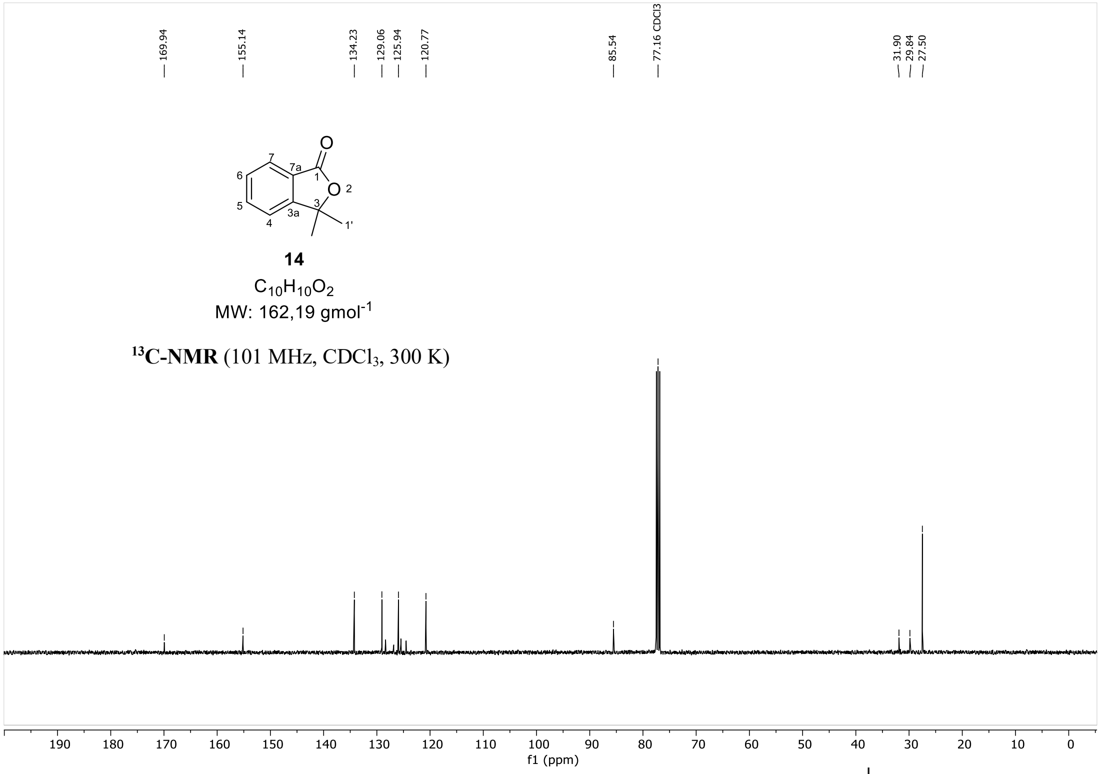
169.94
155.14
134.23
129.06
125.94
120.77
85.54
77.16 CDCl3
31.90
29.84
27.50
14
C10H10O2
MW: 162,19 gmol-1
13C-NMR (101 MHz, CDCl3, 300 K)
f1 (ppm)

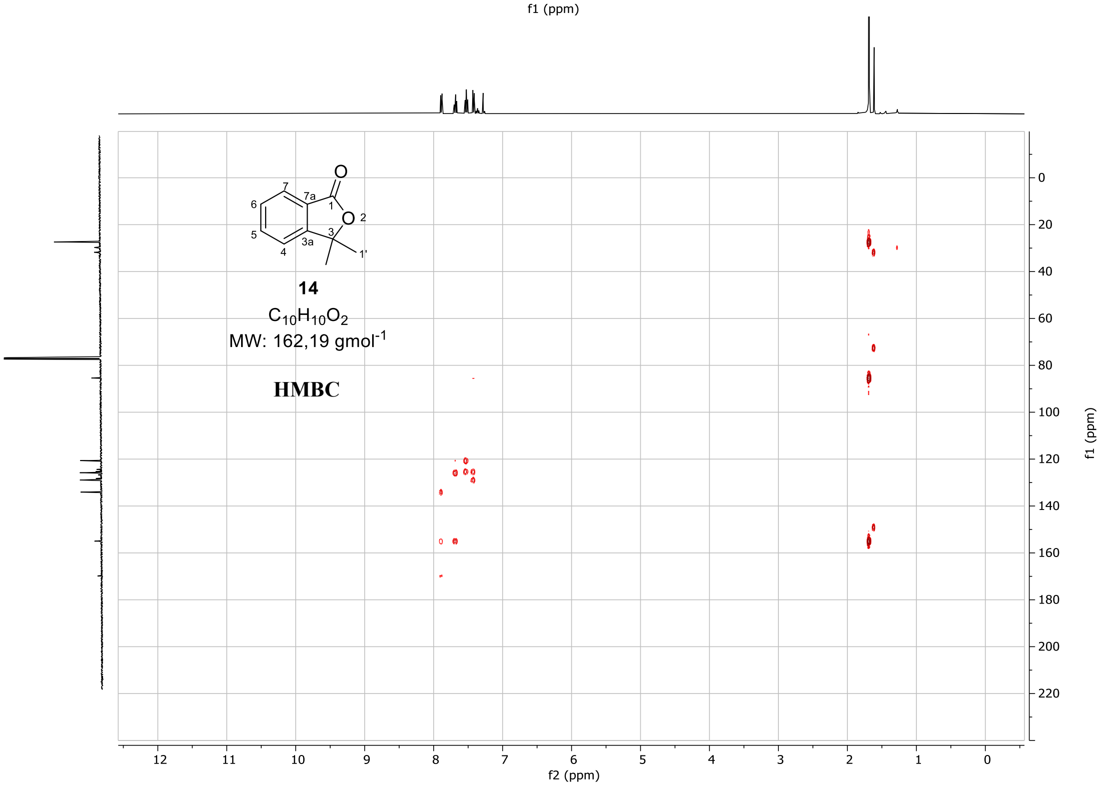
14
C10H10O2
MW: 162,19 gmol-1
HMBC
f2 (ppm)
f1 (ppm)

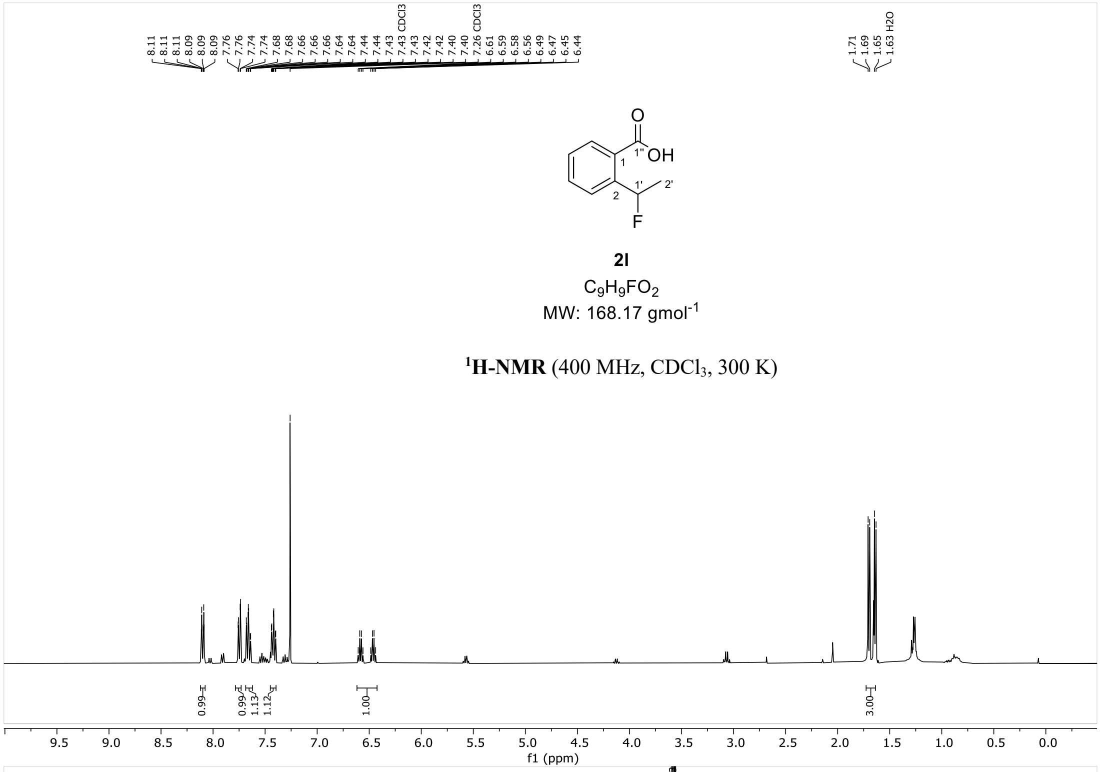
8.11 8.11 8.11 8.09 8.09 8.09 7.76 7.76 7.74 7.74 7.68 7.68 7.66 7.66 7.64 7.64 7.44 7.44 7.43 CDCl3 7.43 7.43 7.42 7.40 7.40 7.26 CDCl3 6.61 6.59 6.58 6.56 6.49 6.47 6.45 6.44
1.71 1.69 1.65 1.63 H2O
O
OH
1''
1
2
1'
2'
F
2l
$C_9H_9FO_2$
MW: 168.17 gmol^{-1}
^{1}H-NMR (400 MHz, $CDCl_3$, 300 K)
0.99
0.99
1.13
1.12
1.00
3.00
9.5 9.0 8.5 8.0 7.5 7.0 6.5 6.0 5.5 5.0 4.5 4.0 3.5 3.0 2.5 2.0 1.5 1.0 0.5 0.0
f1 (ppm)

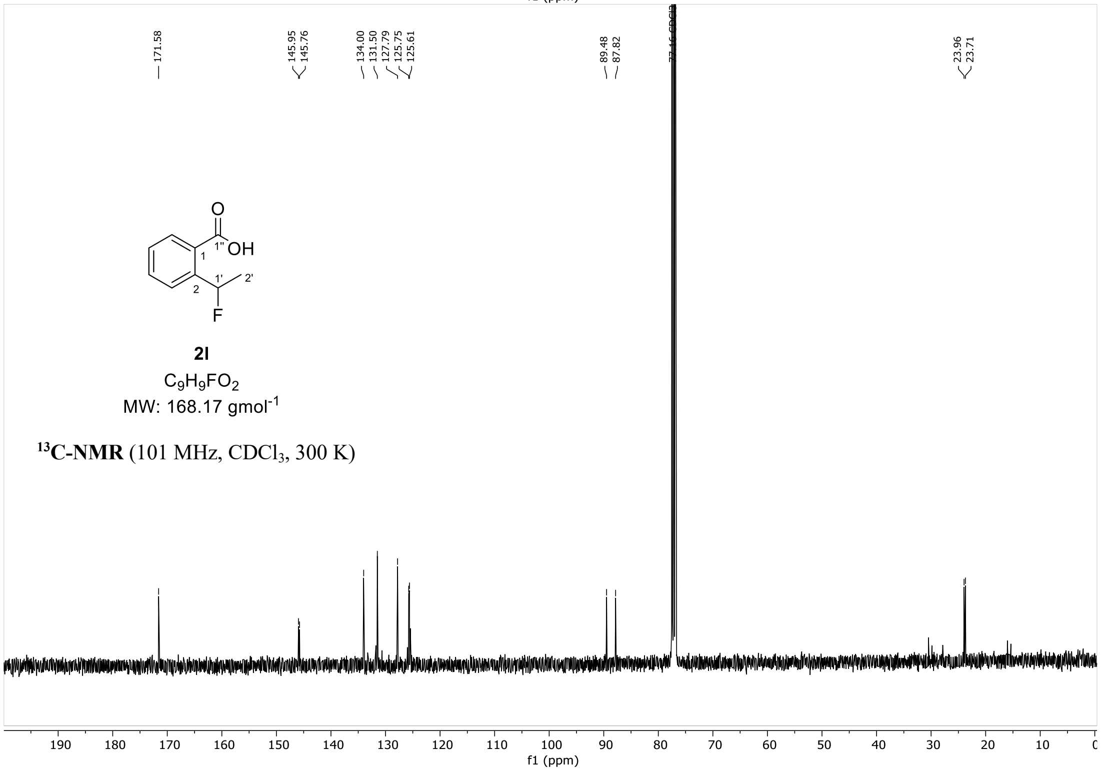
171.58
145.95 145.76
134.00 131.50 127.79 125.75 125.61
89.48 87.82
23.96 23.71
O
OH
1''
1
2
1'
2'
F
2l
$C_9H_9FO_2$
MW: 168.17 gmol^{-1}
^{13}C-NMR (101 MHz, $CDCl_3$, 300 K)
190 180 170 160 150 140 130 120 110 100 90 80 70 60 50 40 30 20 10
f1 (ppm)

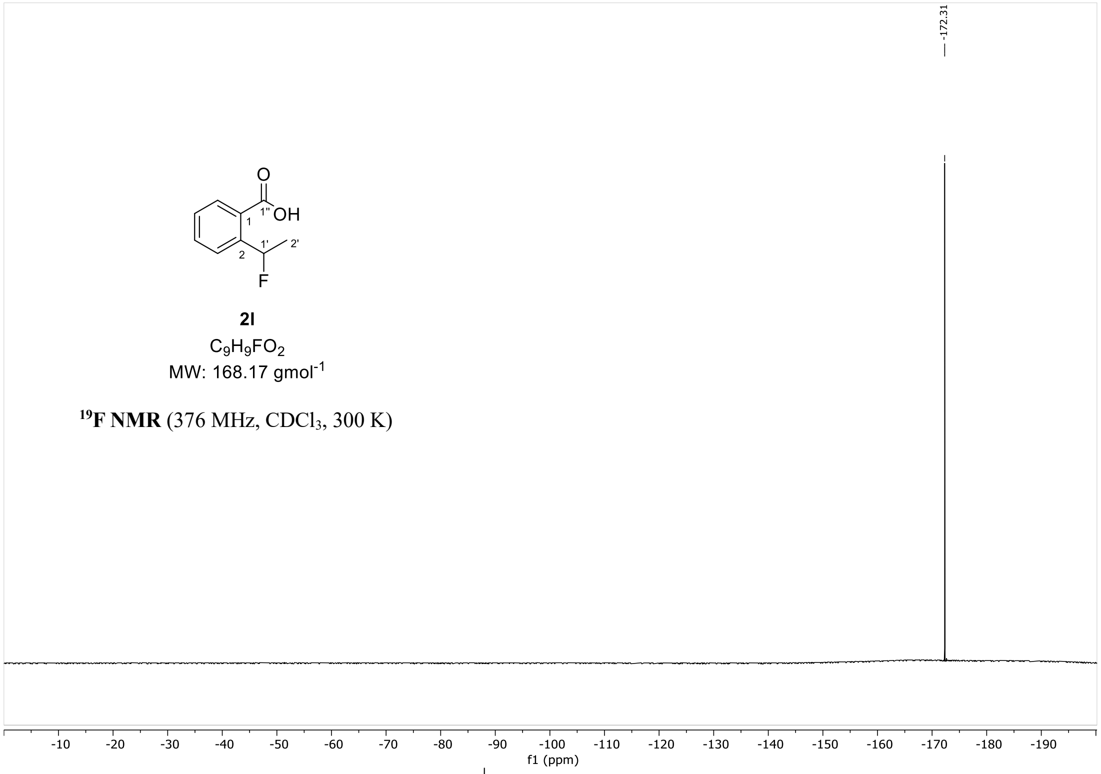
-172.31
O
1''
OH
1
1'
2'
2
F
2l
C9H9FO2
MW: 168.17 gmol-1
19F NMR (376 MHz, CDCl3, 300 K)
-10 -20 -30 -40 -50 -60 -70 -80 -90 -100 -110 -120 -130 -140 -150 -160 -170 -180 -190
f1 (ppm)

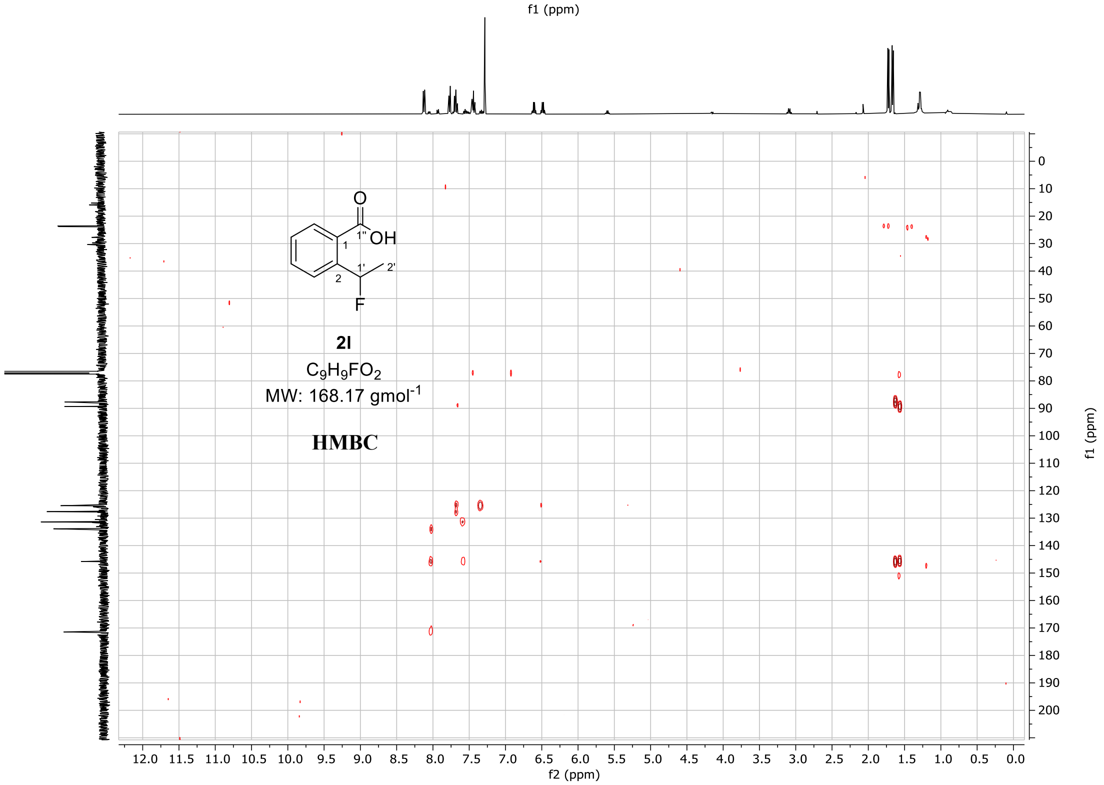
O
1''
OH
1
1'
2'
2
F
2l
C9H9FO2
MW: 168.17 gmol-1
HMBC
12.0 11.5 11.0 10.5 10.0 9.5 9.0 8.5 8.0 7.5 7.0 6.5 6.0 5.5 5.0 4.5 4.0 3.5 3.0 2.5 2.0 1.5 1.0 0.5 0.0
f2 (ppm)
0 10 20 30 40 50 60 70 80 90 100 110 120 130 140 150 160 170 180 190 200
f1 (ppm)

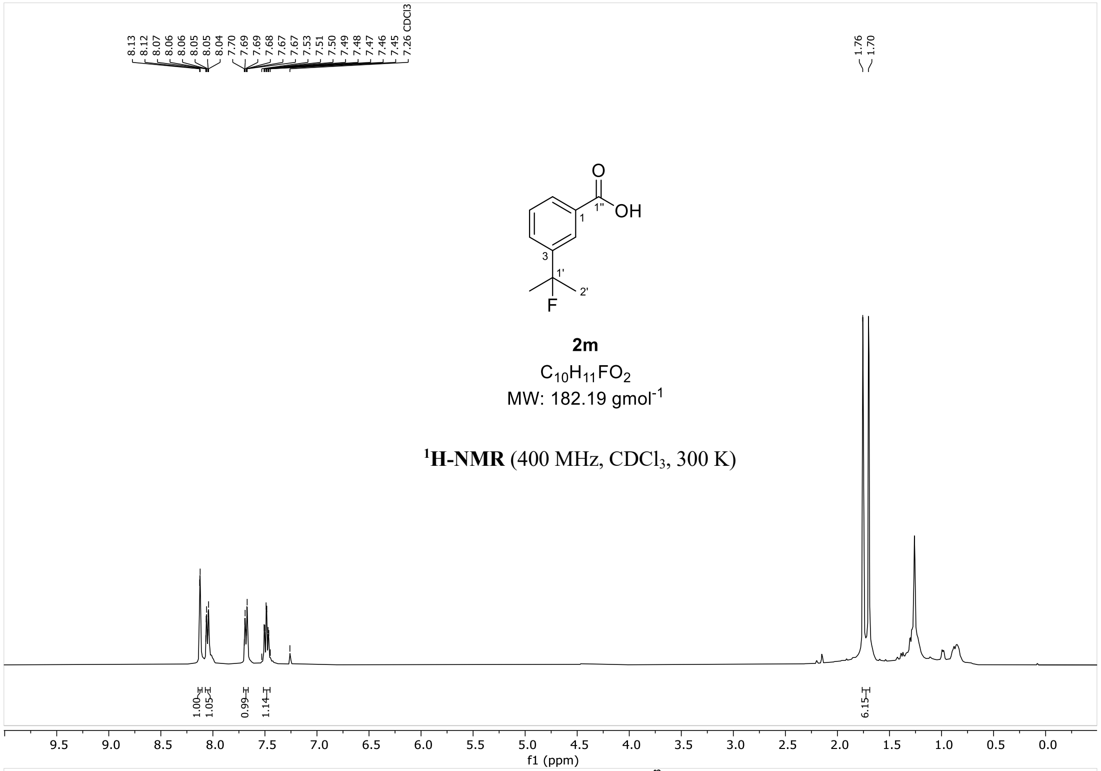
8.13 8.12 8.07 8.06 8.06 8.05 8.05 8.04 7.70 7.69 7.69 7.68 7.67 7.67 7.53 7.51 7.50 7.49 7.48 7.47 7.46 7.45 7.26 CDCl3
1.76 1.70
O
OH
1''
1
3
1'
2'
F
2m
$C_{10}H_{11}FO_2$
MW: 182.19 gmol^{-1}
^{1}H-NMR (400 MHz, $CDCl_3$, 300 K)
1.00 1.05 0.99 1.14 6.15
9.5 9.0 8.5 8.0 7.5 7.0 6.5 6.0 5.5 5.0 4.5 4.0 3.5 3.0 2.5 2.0 1.5 1.0 0.5 0.0
f1 (ppm)

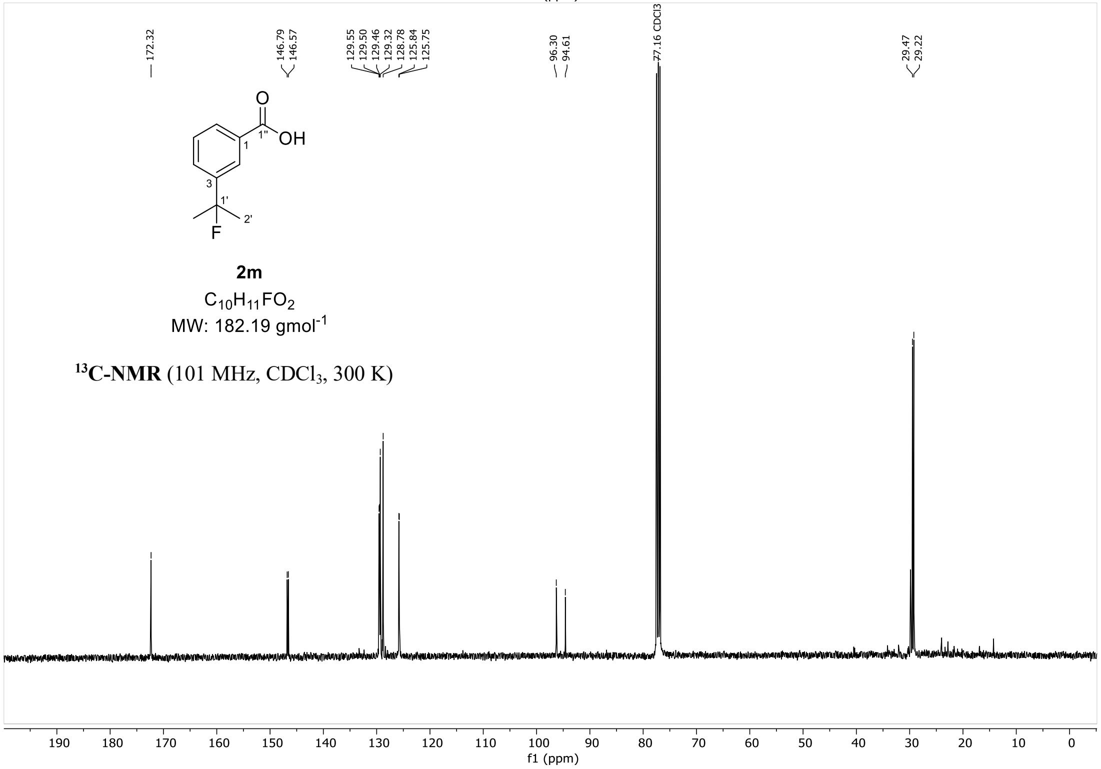
172.32
146.79 146.57
129.55 129.50 129.46 129.32 128.78 125.84 125.75
96.30 94.61
77.16 CDCl3
29.47 29.22
O
OH
1''
1
3
1'
2'
F
2m
$C_{10}H_{11}FO_2$
MW: 182.19 gmol^{-1}
^{13}C-NMR (101 MHz, $CDCl_3$, 300 K)
190 180 170 160 150 140 130 120 110 100 90 80 70 60 50 40 30 20 10 0
f1 (ppm)

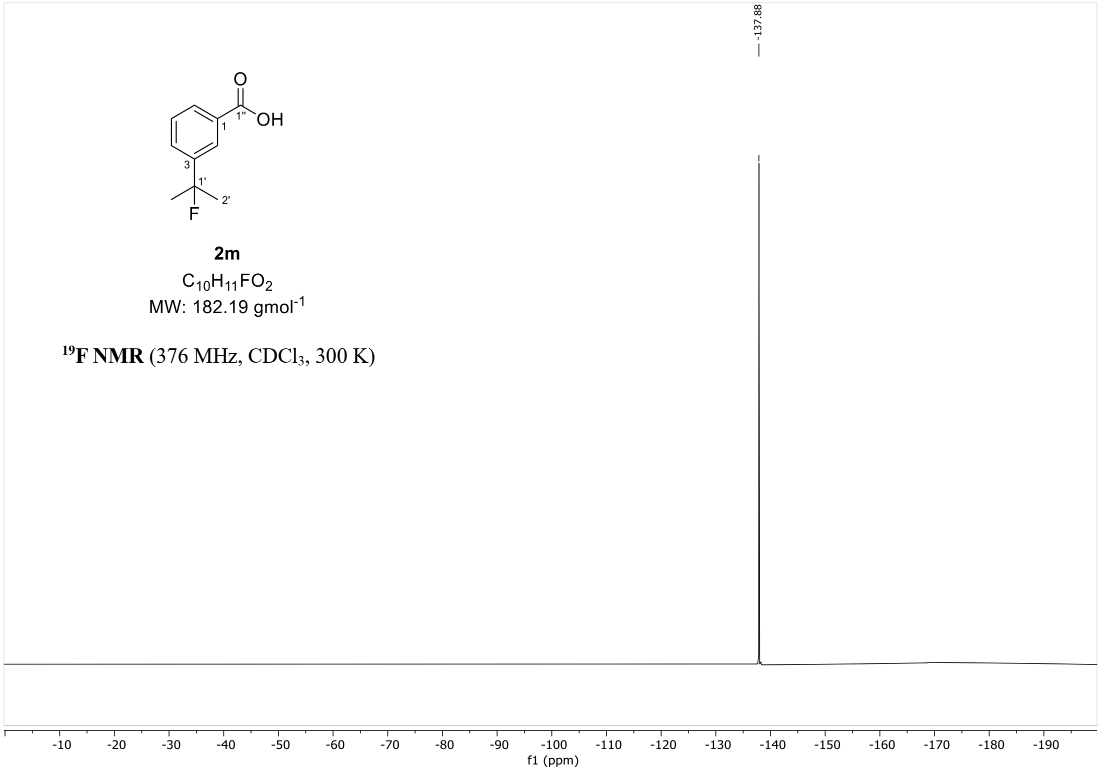
-137.88
O
1''
OH
1
3
1'
2'
F
2m
$C_{10}H_{11}FO_2$
MW: 182.19 gmol-1
19F NMR (376 MHz, CDCl3, 300 K)
f1 (ppm)

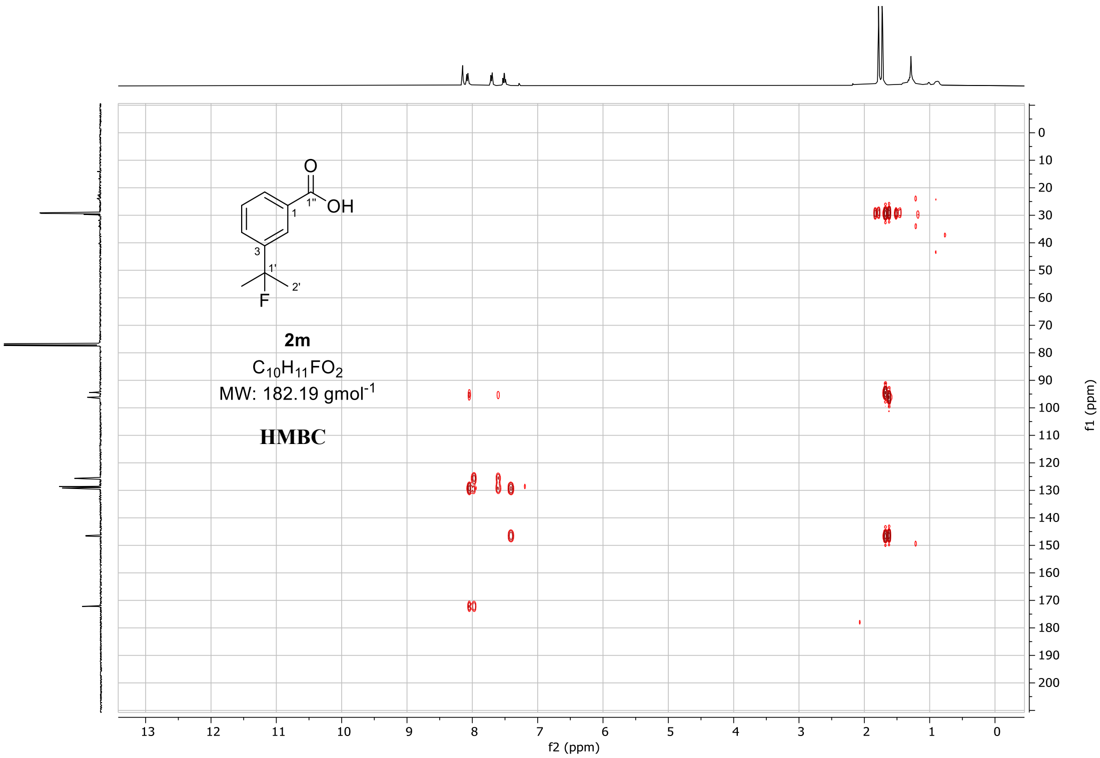
O
1''
OH
1
3
1'
2'
F
2m
$C_{10}H_{11}FO_2$
MW: 182.19 gmol-1
HMBC
f2 (ppm)
f1 (ppm)

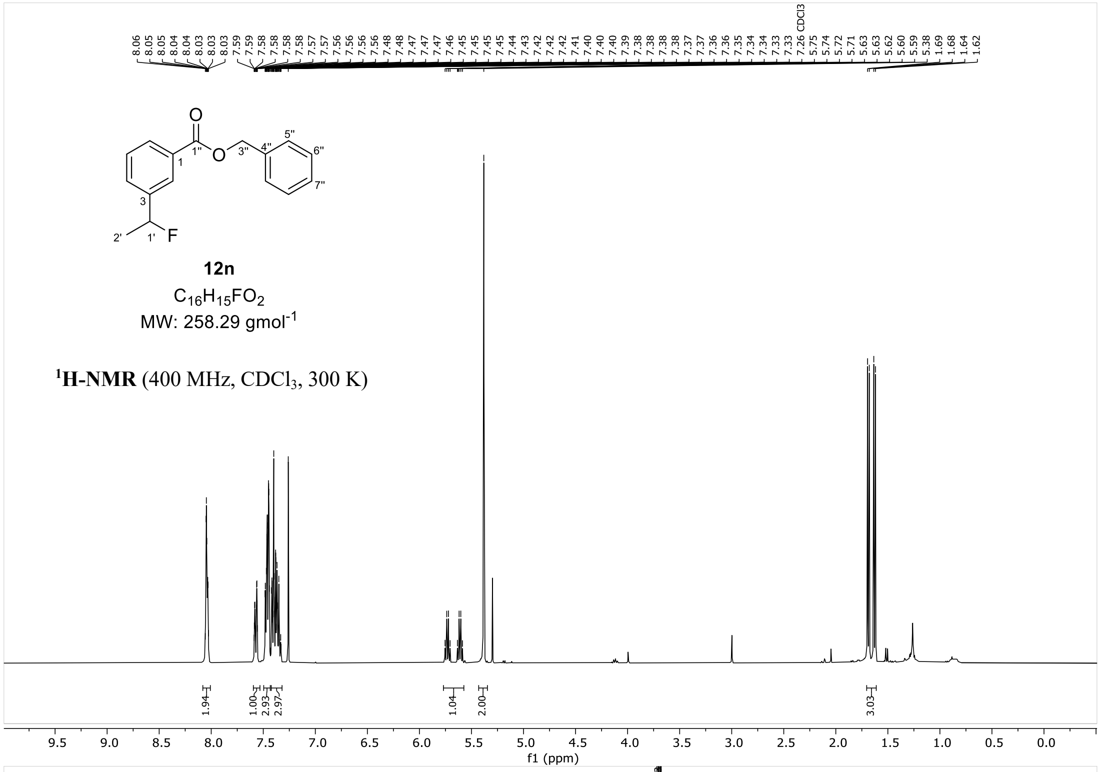

12n
$C_{16}H_{15}FO_2$
MW: 258.29 gmol^{-1}
^{1}**H-NMR** (400 MHz, $CDCl_3$, 300 K)
f1 (ppm)

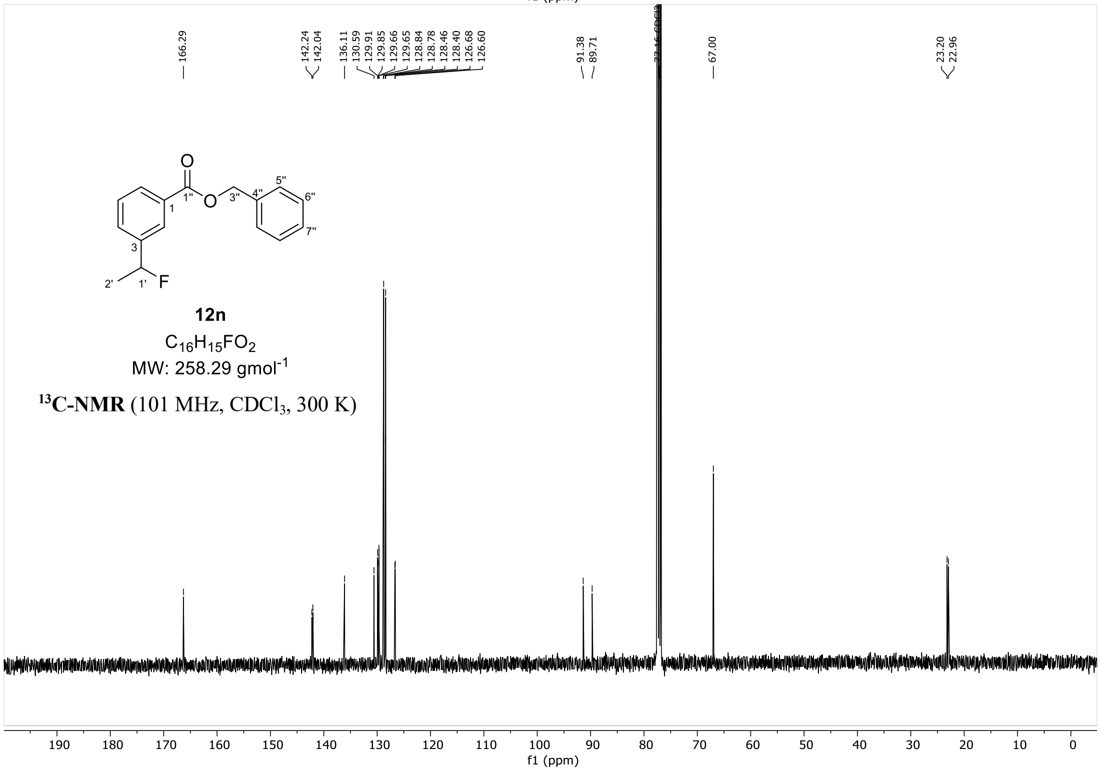

12n
$C_{16}H_{15}FO_2$
MW: 258.29 gmol^{-1}
^{13}**C-NMR** (101 MHz, $CDCl_3$, 300 K)
f1 (ppm)

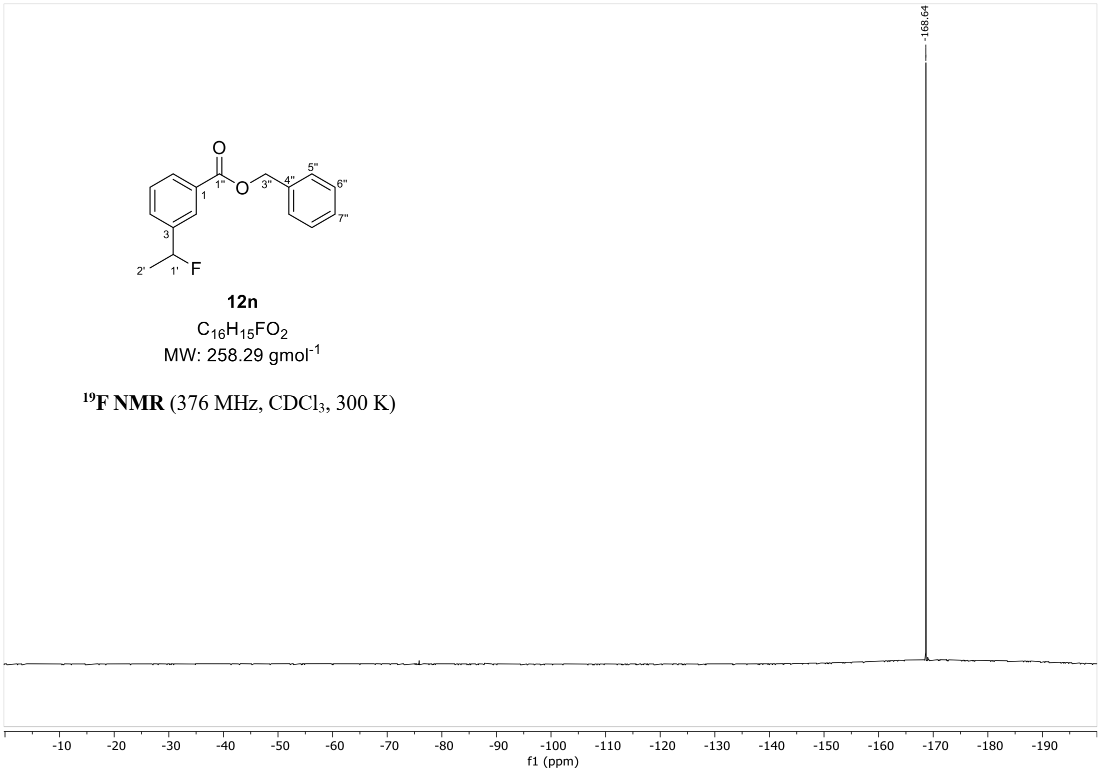
-168.64
12n
$C_{16}H_{15}FO_2$
MW: 258.29 gmol^{-1}
^{19}F NMR (376 MHz, $CDCl_3$, 300 K)
f1 (ppm)

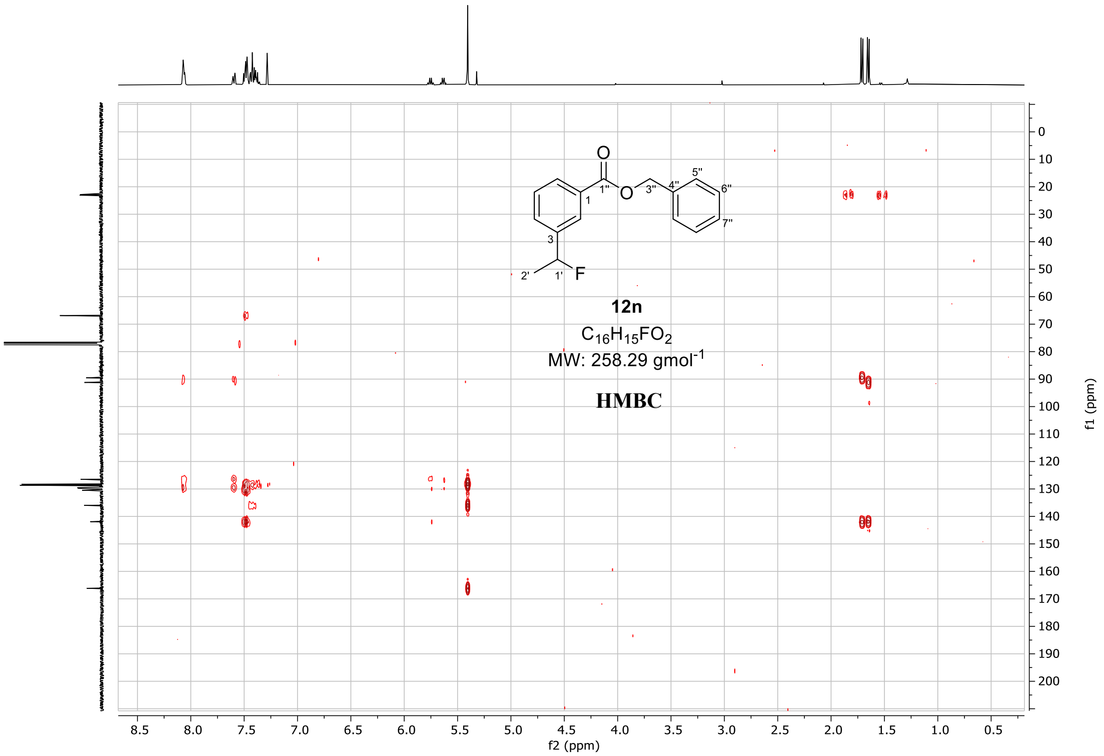
12n
$C_{16}H_{15}FO_2$
MW: 258.29 gmol^{-1}
HMBC
f2 (ppm)
f1 (ppm)

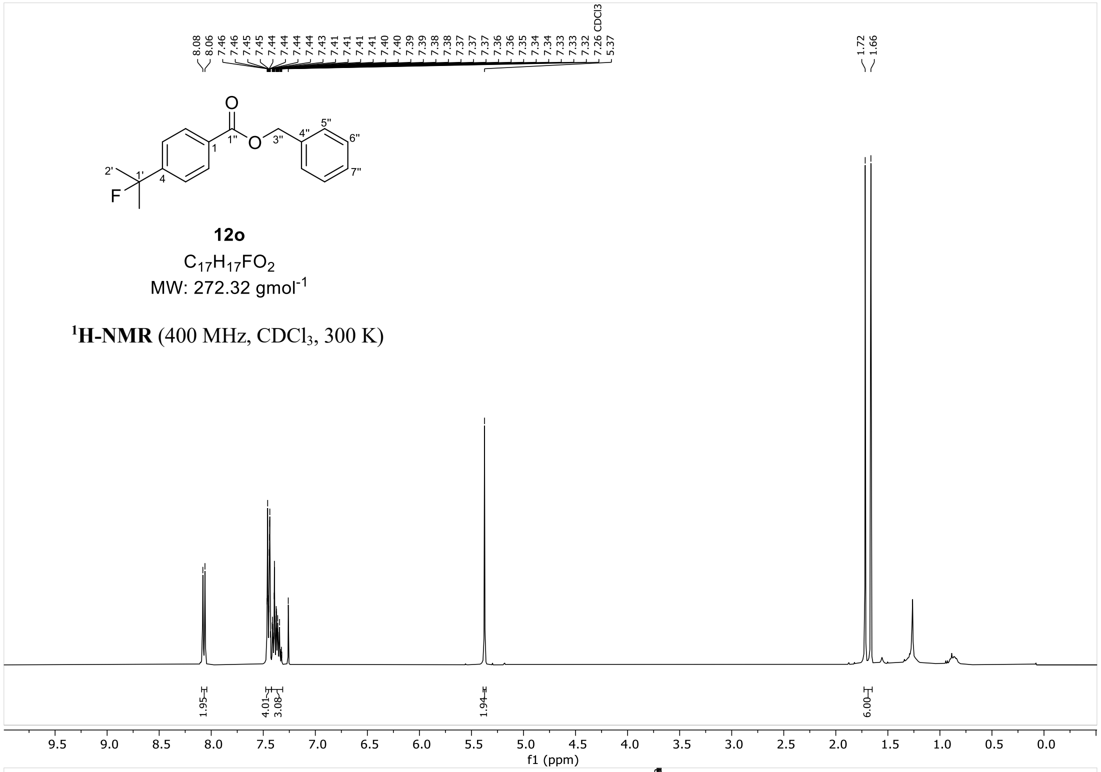
12o
$C_{17}H_{17}FO_2$
MW: 272.32 gmol^{-1}
^{1}H-NMR (400 MHz, $CDCl_3$, 300 K)
8.08 8.06 7.46 7.45 7.45 7.44 7.44 7.44 7.44 7.43 7.41 7.41 7.41 7.41 7.40 7.40 7.39 7.39 7.38 7.38 7.37 7.37 7.37 7.36 7.36 7.35 7.34 7.34 7.33 7.33 7.32 7.26 CDCl3 5.37
1.72 1.66
1.95 4.01 3.08 1.94 6.00
f1 (ppm)

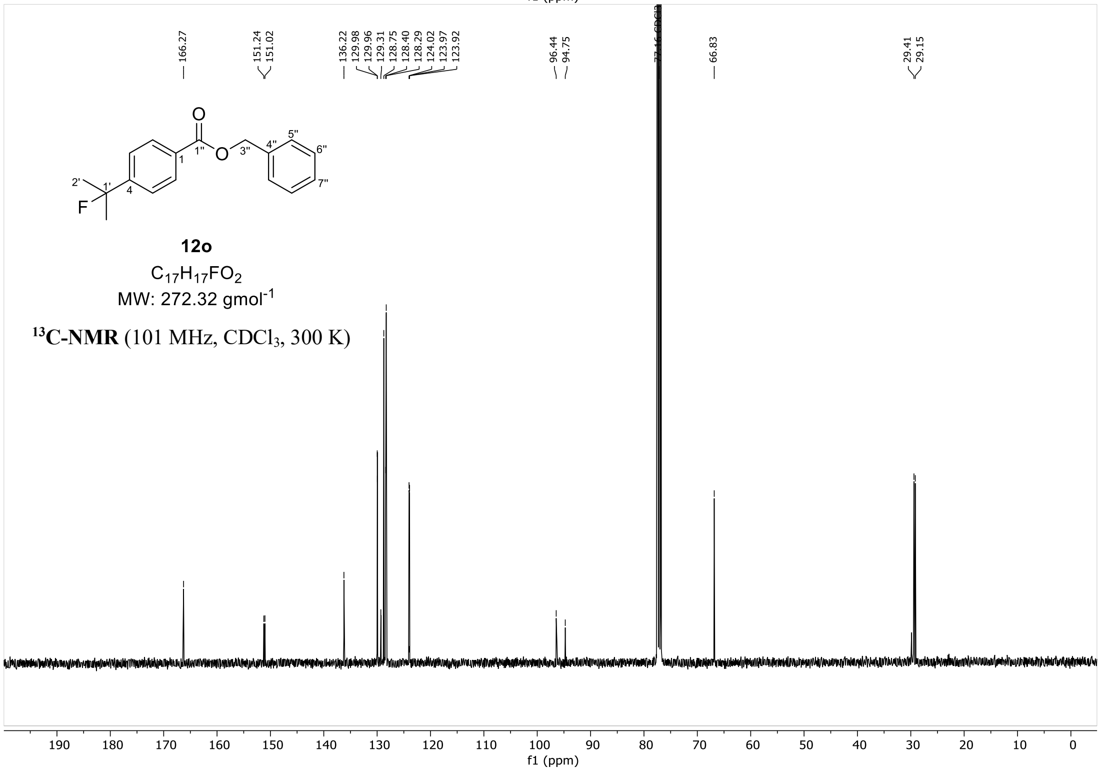
12o
$C_{17}H_{17}FO_2$
MW: 272.32 gmol^{-1}
^{13}C-NMR (101 MHz, $CDCl_3$, 300 K)
166.27
151.24 151.02
136.22 129.98 129.96 129.31 128.75 128.40 128.29 124.02 123.97 123.92
96.44 94.75
66.83
29.41 29.15
f1 (ppm)

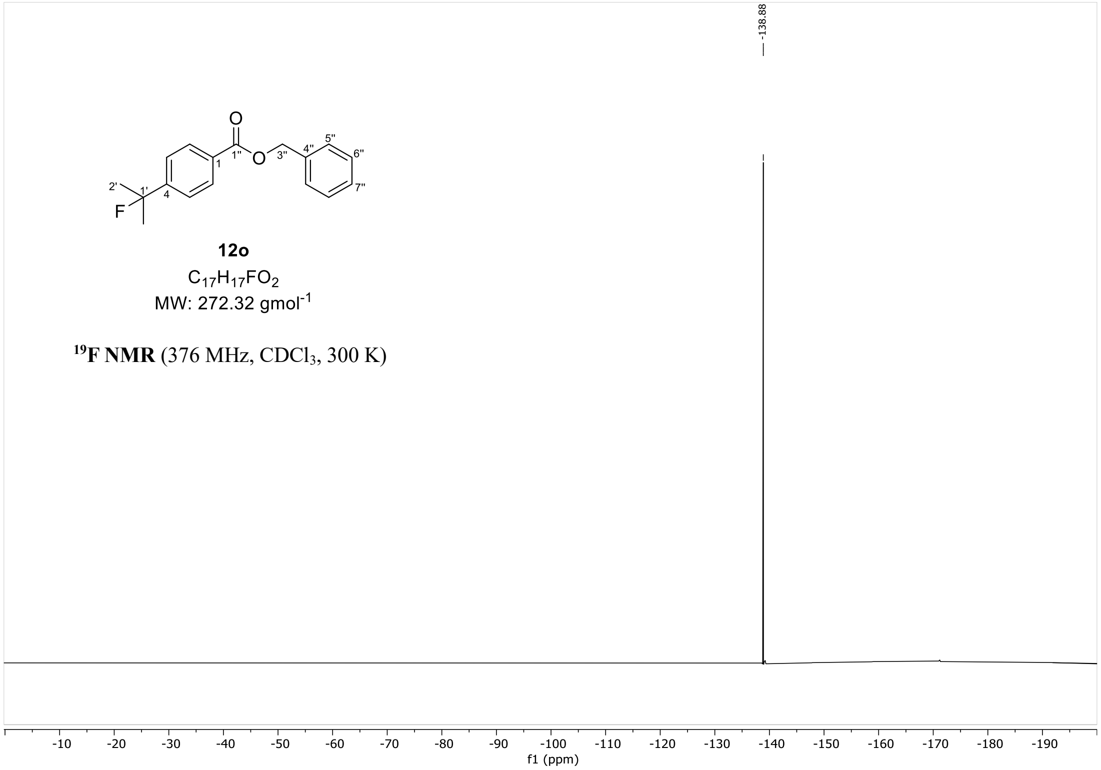
-138.88
1''
3''
4''
5''
6''
7''
2'
1'
4
1
F
O
O
12o
C17H17FO2
MW: 272.32 gmol-1
19F NMR (376 MHz, CDCl3, 300 K)
-10 -20 -30 -40 -50 -60 -70 -80 -90 -100 -110 -120 -130 -140 -150 -160 -170 -180 -190
f1 (ppm)

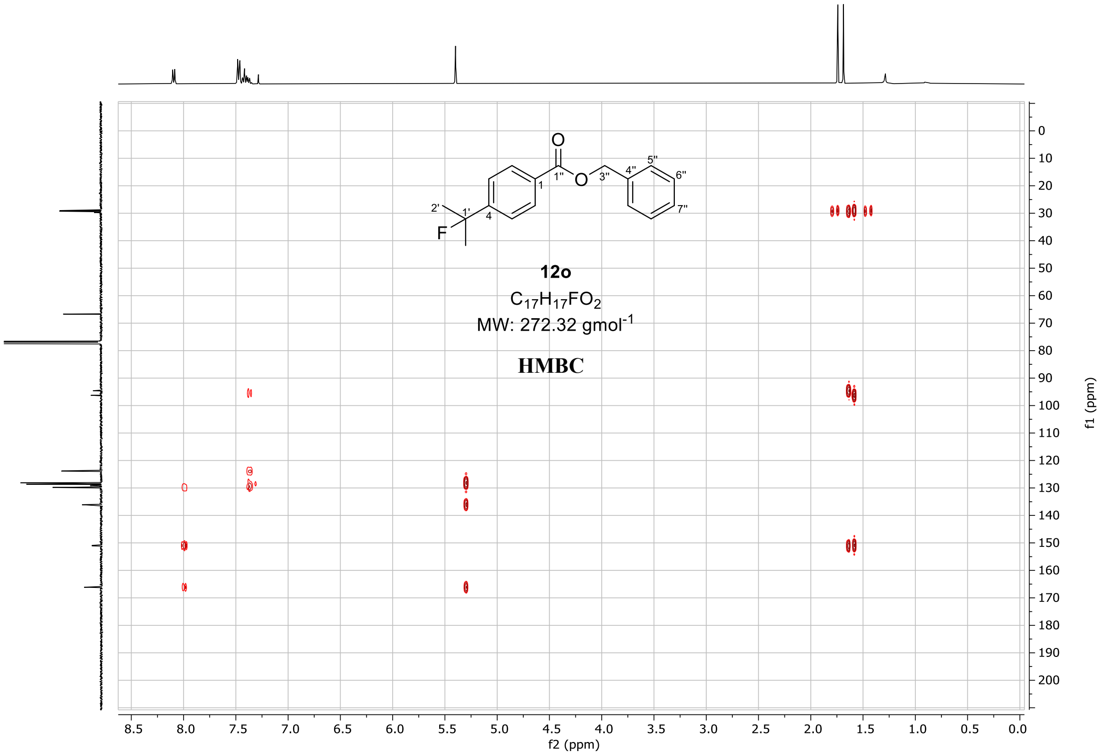
12o
C17H17FO2
MW: 272.32 gmol-1
HMBC
0 10 20 30 40 50 60 70 80 90 100 110 120 130 140 150 160 170 180 190 200
f1 (ppm)
8.5 8.0 7.5 7.0 6.5 6.0 5.5 5.0 4.5 4.0 3.5 3.0 2.5 2.0 1.5 1.0 0.5 0.0
f2 (ppm)

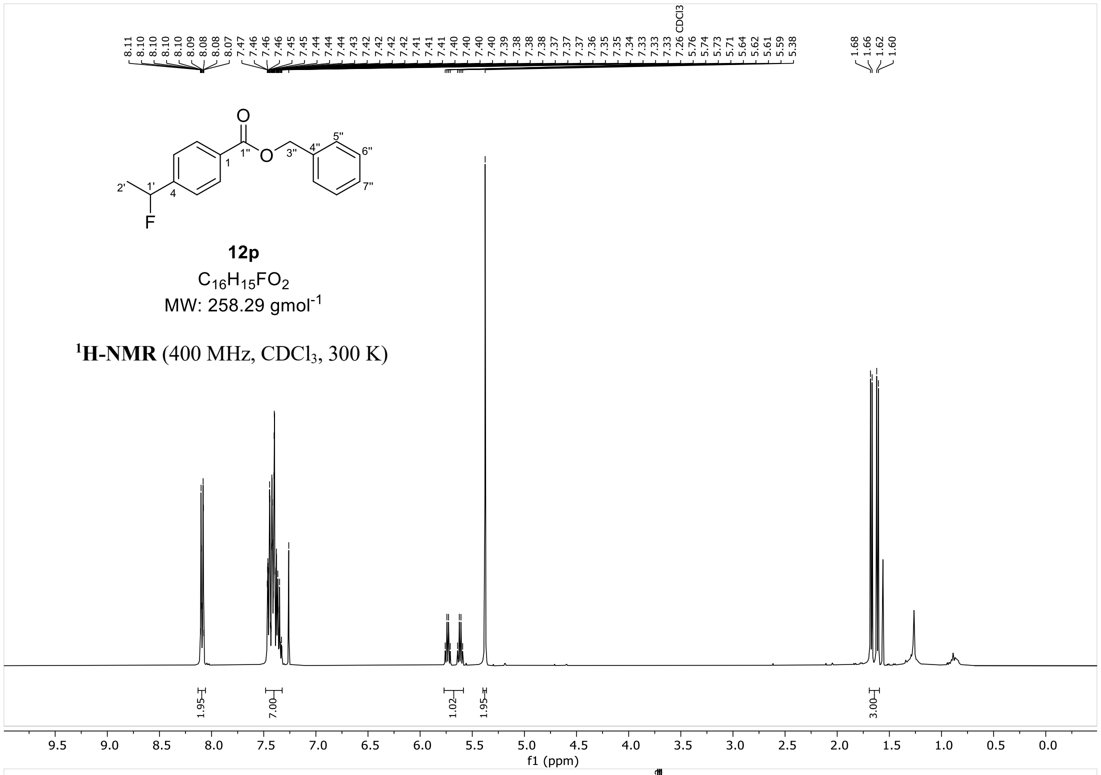
8.11 8.10 8.10 8.10 8.09 8.08 8.08 8.07 7.47 7.46 7.46 7.45 7.45 7.44 7.44 7.43 7.42 7.42 7.42 7.42 7.41 7.41 7.41 7.40 7.40 7.40 7.40 7.39 7.38 7.38 7.38 7.37 7.37 7.36 7.35 7.35 7.34 7.33 7.33 7.26 CDCl3 5.76 5.74 5.73 5.71 5.64 5.62 5.61 5.59 5.38
1.68 1.66 1.62 1.60
12p
$C_{16}H_{15}FO_2$
MW: 258.29 gmol^{-1}
^{1}H-NMR (400 MHz, $CDCl_3$, 300 K)
1.95 7.00 1.02 1.95 3.00
f1 (ppm)

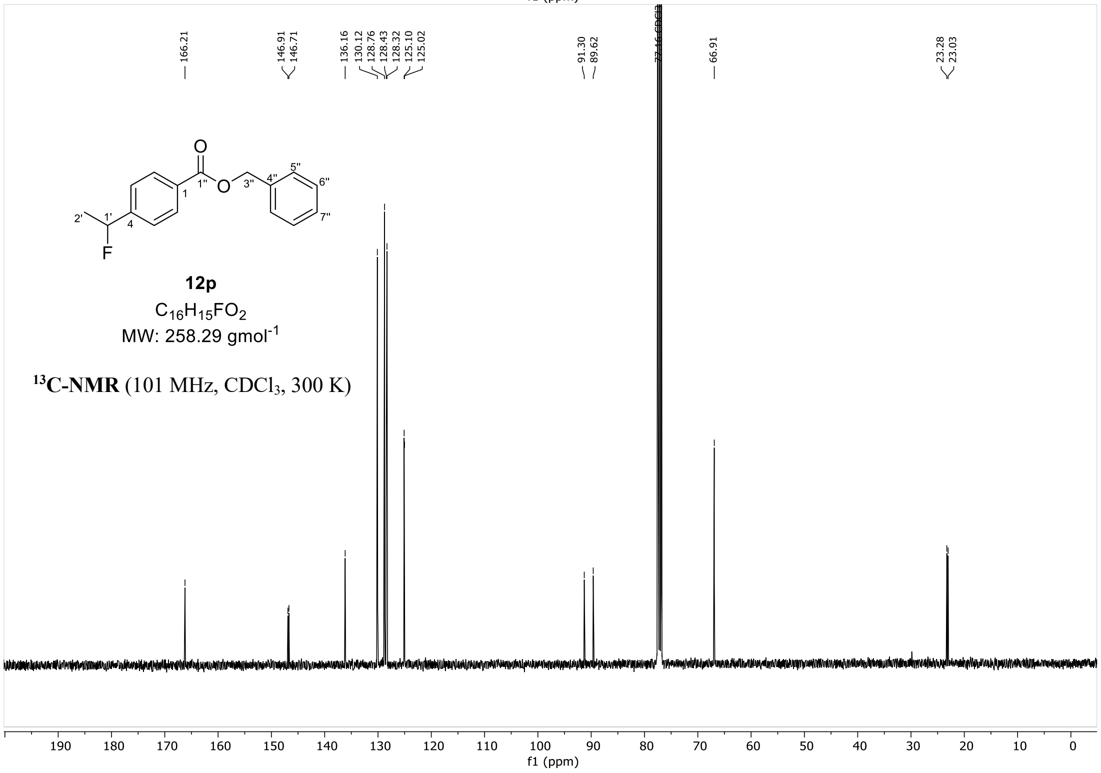
166.21
146.91 146.71
136.16 130.12 128.76 128.43 128.32 125.10 125.02
91.30 89.62
77.16 CDCl3
66.91
23.28 23.03
12p
$C_{16}H_{15}FO_2$
MW: 258.29 gmol^{-1}
^{13}C-NMR (101 MHz, $CDCl_3$, 300 K)
f1 (ppm)

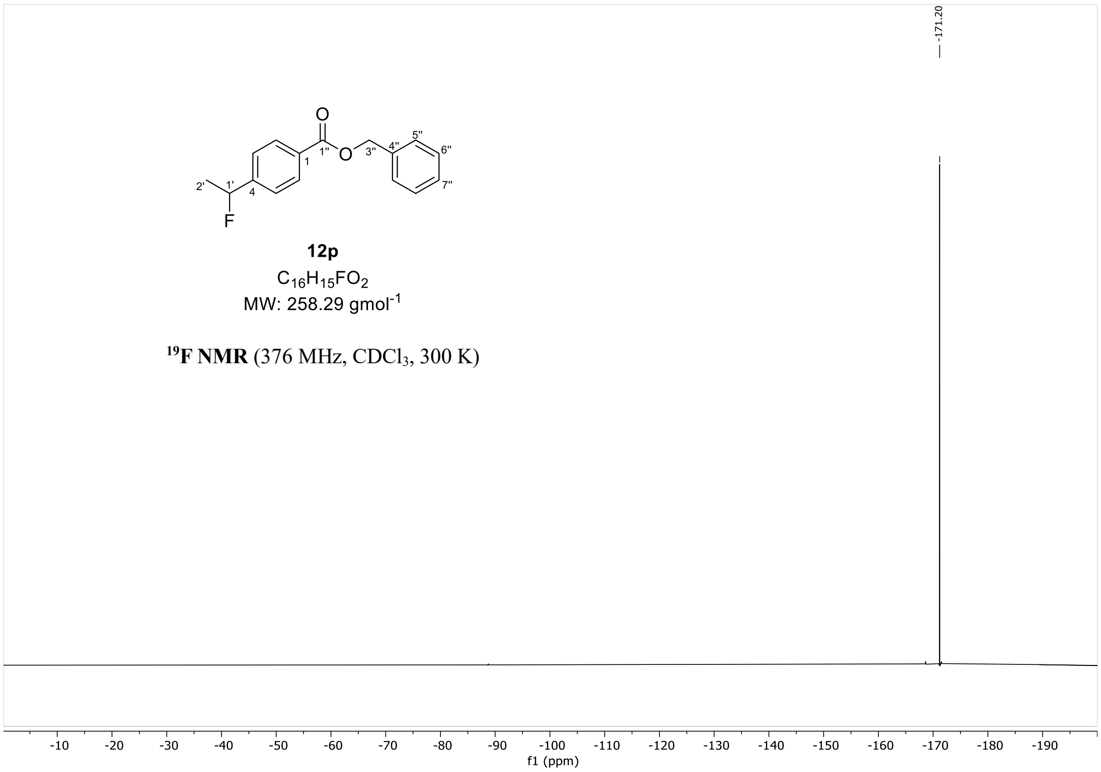
-171.20
12p
$C_{16}H_{15}FO_2$
MW: 258.29 gmol^{-1}
^{19}F NMR (376 MHz, CDCl$_3$, 300 K)
f1 (ppm)

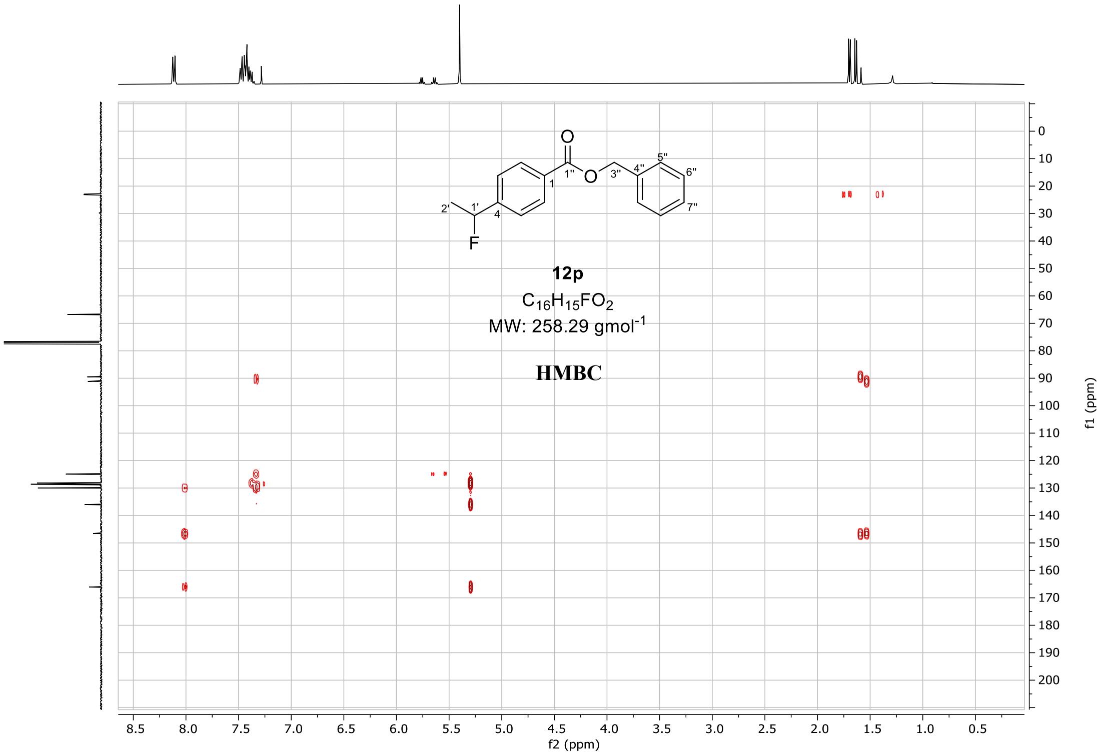
12p
$C_{16}H_{15}FO_2$
MW: 258.29 gmol^{-1}
HMBC
f2 (ppm)
f1 (ppm)

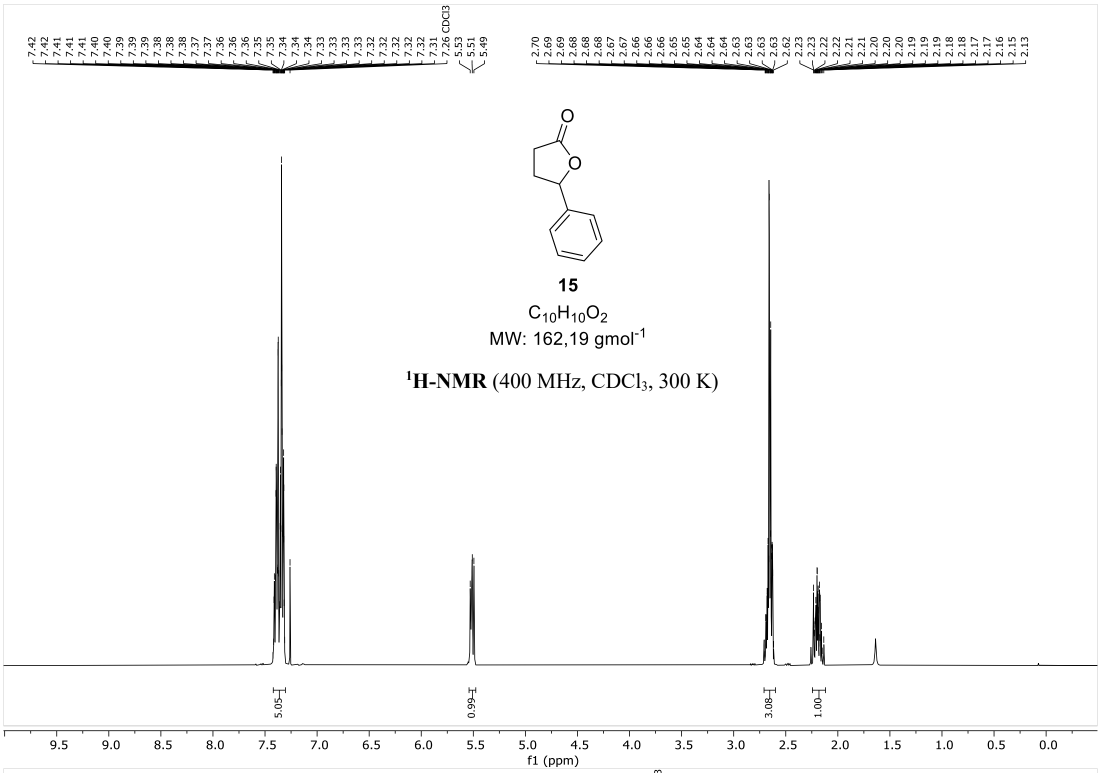
7.42 7.42 7.41 7.41 7.40 7.39 7.39 7.39 7.38 7.38 7.38 7.37 7.37 7.36 7.36 7.36 7.35 7.35 7.34 7.34 7.34 7.33 7.33 7.33 7.33 7.32 7.32 7.32 7.32 7.31 7.26 CDCl3
5.53 5.51 5.49
2.70 2.69 2.69 2.68 2.68 2.67 2.67 2.66 2.66 2.66 2.65 2.65 2.64 2.64 2.64 2.63 2.63 2.63 2.63 2.62 2.23 2.23 2.22 2.22 2.21 2.21 2.20 2.20 2.20 2.19 2.19 2.19 2.18 2.18 2.17 2.17 2.16 2.15 2.13
O
O
15
$C_{10}H_{10}O_2$
MW: 162,19 gmol^{-1}
^{1}H-NMR (400 MHz, $CDCl_3$, 300 K)
5.05
0.99
3.08
1.00
9.5 9.0 8.5 8.0 7.5 7.0 6.5 6.0 5.5 5.0 4.5 4.0 3.5 3.0 2.5 2.0 1.5 1.0 0.5 0.0
f1 (ppm)

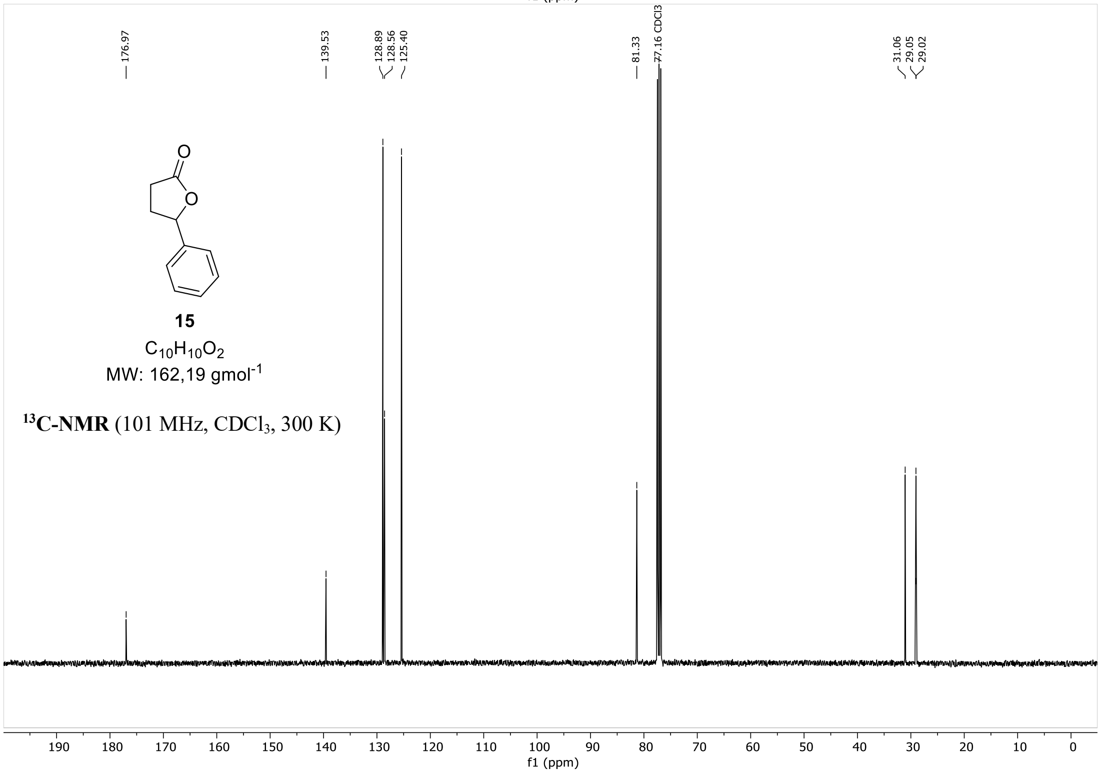
176.97
139.53
128.89 128.56 125.40
81.33
77.16 CDCl3
31.06 29.05 29.02
O
O
15
$C_{10}H_{10}O_2$
MW: 162,19 gmol^{-1}
^{13}C-NMR (101 MHz, $CDCl_3$, 300 K)
190 180 170 160 150 140 130 120 110 100 90 80 70 60 50 40 30 20 10 0
f1 (ppm)

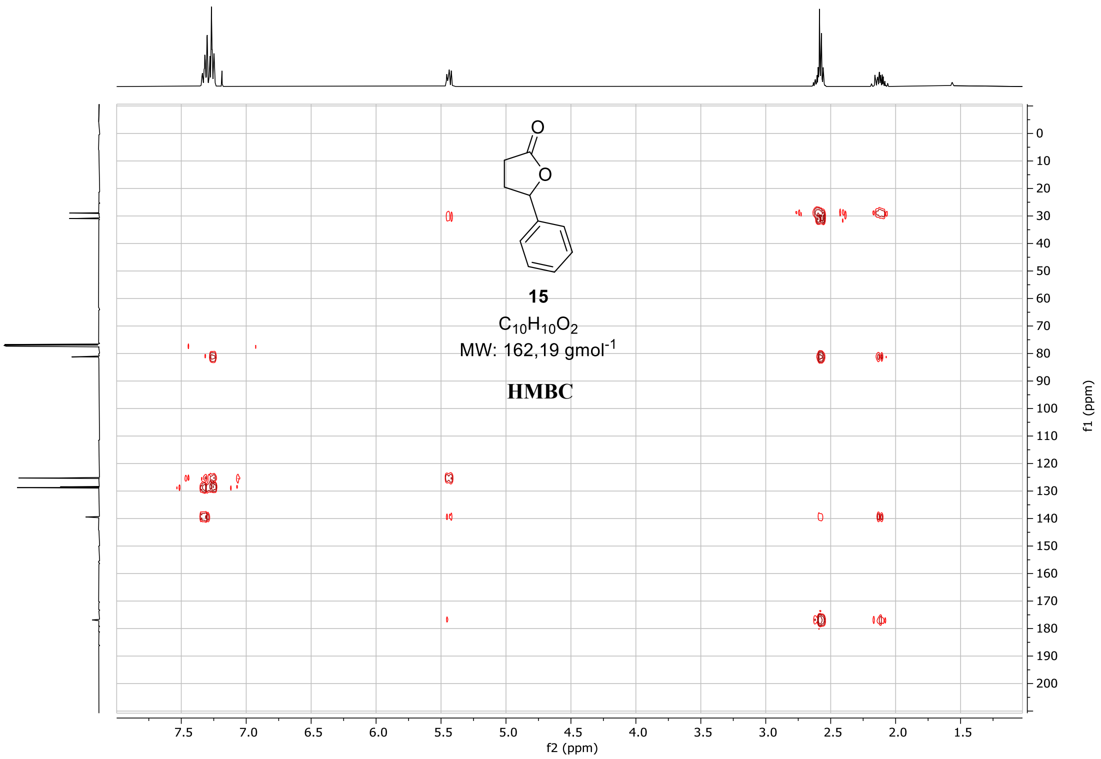

15
$C_{10}H_{10}O_2$
MW: 162,19 gmol-1
HMBC
f1 (ppm)
f2 (ppm)

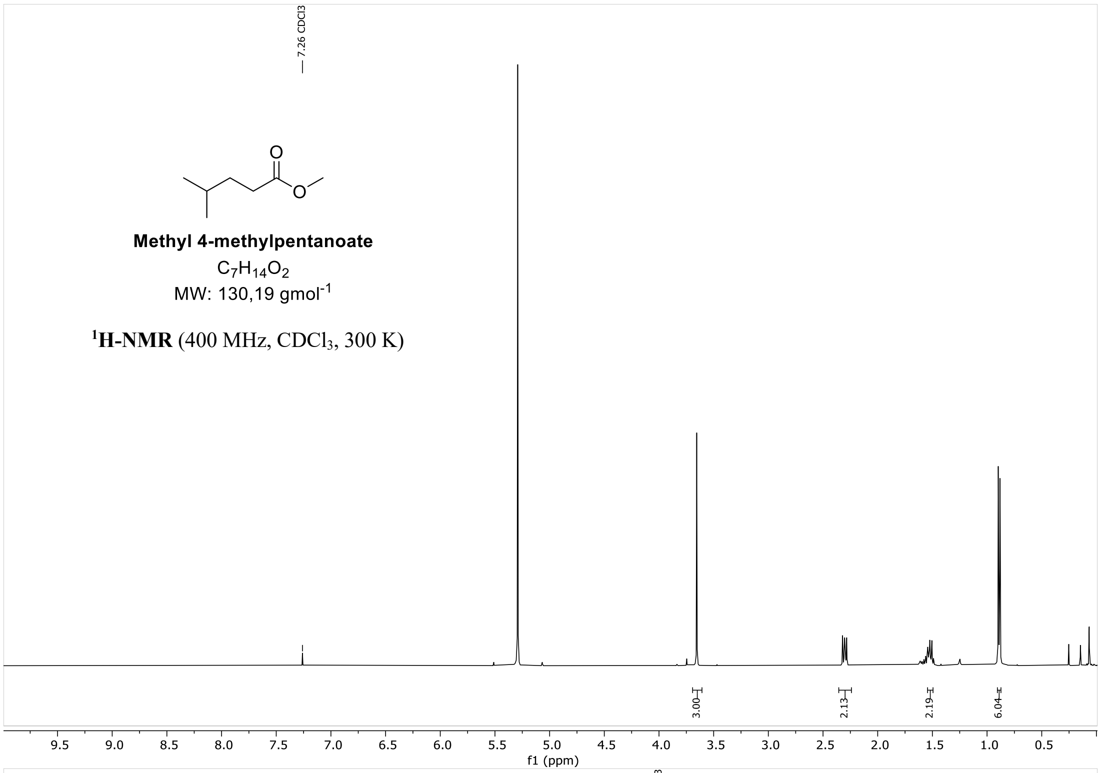
7.26 CDCl3
Methyl 4-methylpentanoate
$C_7H_{14}O_2$
MW: 130,19 gmol-1
1H-NMR (400 MHz, $CDCl_3$, 300 K)
3.00
2.13
2.19
6.04
9.5 9.0 8.5 8.0 7.5 7.0 6.5 6.0 5.5 5.0 4.5 4.0 3.5 3.0 2.5 2.0 1.5 1.0 0.5
f1 (ppm)

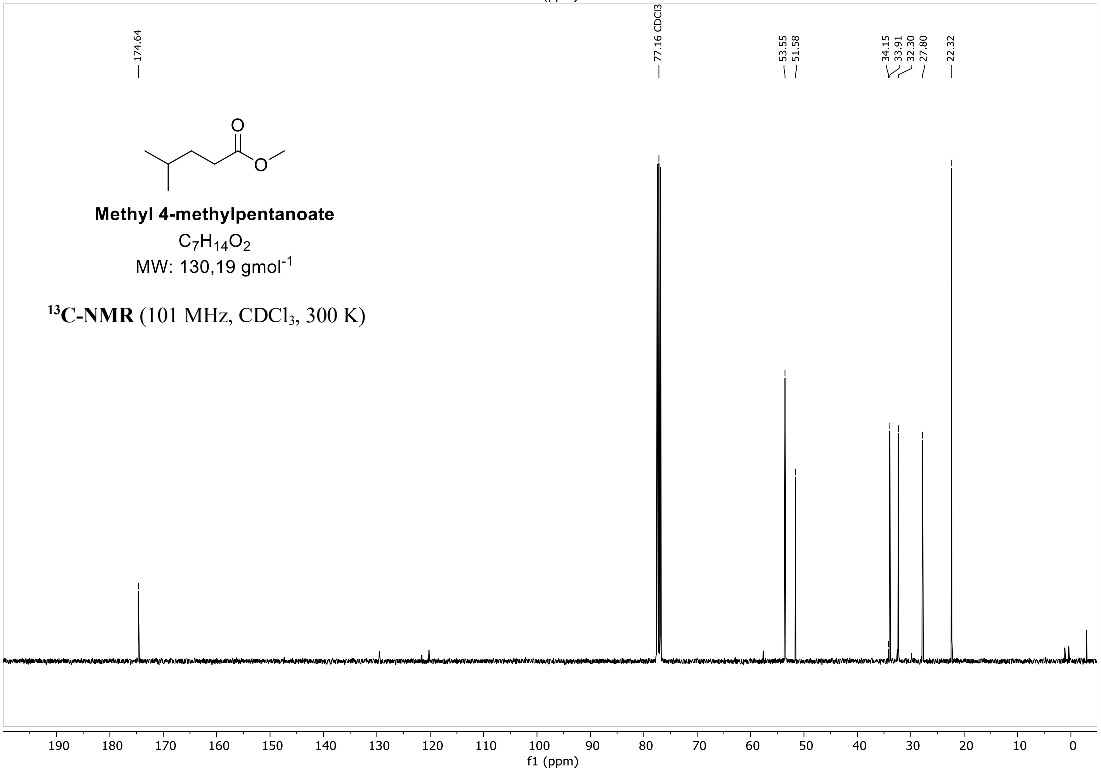
174.64
77.16 CDCl3
53.55
51.58
34.15
33.91
32.30
27.80
22.32
Methyl 4-methylpentanoate
$C_7H_{14}O_2$
MW: 130,19 gmol-1
13C-NMR (101 MHz, $CDCl_3$, 300 K)
190 180 170 160 150 140 130 120 110 100 90 80 70 60 50 40 30 20 10 0
f1 (ppm)

S9. List of References

(1) Yin, F.; Wang, Z.; Li, Z.; Li, C. Silver-Catalyzed Decarboxylative Fluorination of Aliphatic Carboxylic Acids in Aqueous Solution. *J. Am. Chem. Soc.* **2012**, *134*, 10401-10404.

(2) Zhuang, Z.; Sheng, T.; Qiao, J. X.; Yeung, K.-S.; Yu, J.-Q. Versatile Copper-Catalyzed γ-C(sp^3)–H Lactonization of Aliphatic Acids. *J. Am. Chem. Soc.* **2024**, *146*, 17311-17317.

(3) Halperin, S. D.; Fan, H.; Chang, S.; Martin, R. E.; Britton, R. A Convenient Photocatalytic Fluorination of Unactivated C-H Bonds. *Angew. Chem. Int. Ed.* **2014**, *53*, 4690-4693.

(4) Morcillo, S. P.; Dauncey, E. M.; Kim, J. H.; Douglas, J. J.; Sheikh, N. S.; Leonori, D. Photoinduced Remote Functionalization of Amides and Amines Using Electrophilic Nitrogen Radicals. *Angew. Chem. Int. Ed.* **2018**, *57*, 12945-12949.

(5) Romero, N. A.; Nicewicz, D. A. Mechanistic Insight into the Photoredox Catalysis of Anti-Markovnikov Alkene Hydrofunctionalization Reactions. *J. Am. Chem. Soc.* **2014**, *136*, 17024-17035.
